# Methylated Cytochrome P450 and the Solute Carrier Family of Genes Correlate With Perturbations in Bile Acid Metabolism in Parkinson’s Disease

**DOI:** 10.3389/fnins.2022.804261

**Published:** 2022-03-31

**Authors:** Sangeetha Vishweswaraiah, Sumeyya Akyol, Ali Yilmaz, Zafer Ugur, Juozas Gordevičius, Kyung Joon Oh, Patrik Brundin, Uppala Radhakrishna, Viviane Labrie, Stewart F. Graham

**Affiliations:** ^1^Beaumont Health, Royal Oak, MI, United States; ^2^Van Andel Institute, Grand Rapids, MI, United States

**Keywords:** metabolomics, epigenetics, integrative omics, epimetabolomics, Parkinson’s disease, etiopathophysiology

## Abstract

Parkinson’s disease (PD) is second most prevalent neurodegenerative disorder following Alzheimer’s disease. Parkinson’s disease is hypothesized to be caused by a multifaceted interplay between genetic and environmental factors. Herein, and for the first time, we describe the integration of metabolomics and epigenetics (genome-wide DNA methylation; epimetabolomics) to profile the frontal lobe from people who died from PD and compared them with age-, and sex-matched controls. We identified 48 metabolites to be at significantly different concentrations (FDR *q* < 0.05), 4,313 differentially methylated sites [5’-C-phosphate-G-3’ (CpGs)] (FDR *q* < 0.05) and increased DNA methylation age in the primary motor cortex of people who died from PD. We identified Primary bile acid biosynthesis as the major biochemical pathway to be perturbed in the frontal lobe of PD sufferers, and the metabolite taurine (*p*-value = 5.91E-06) as being positively correlated with CpG cg14286187 (*SLC25A27; CYP39A1*) (FDR *q* = 0.002), highlighting previously unreported biochemical changes associated with PD pathogenesis. In this novel multi-omics study, we identify regulatory mechanisms which we believe warrant future translational investigation and central biomarkers of PD which require further validation in more accessible biomatrices.

## Introduction

Parkinson’s disease (PD) is the most common degenerative movement disorder of the central nervous system, characterized by bradycardia, muscular rigidity, rest tremor, postural and gait impairment (Gibb and Lees, 1988; Poewe et al., 2017) frequently accompanied by non-motor symptoms such as cognitive impairment, sleep disorders, anxiety, depression and autonomic dysfunction (Martinez-Martin et al., 2011). The key pathological finding of PD is the loss of dopaminergic neurons in the *substantia nigra pars compacta* (Fearnley and Lees, 1991) and the presence of Lewy body pathology due to the abnormal aggregation of α-synuclein (Daniel and Lees, 1993; Dickson et al., 2009). Since *SNCA* mutations were identified in familial PD (Polymeropoulos et al., 1997), across various Braak stages, a growing list of genes associated with the pathogenesis of PD have been identified (Corti et al., 2011; Keo et al., 2020).

Parkinson’s disease is also characterized by a range of motor symptoms and speech deficits (Moustafa et al., 2016). The precentral gyrus region is the site of primary motor cortex (Brodmann area 4) and responsible for performing voluntary actions including orofacial movements (Zhi et al., 2019). It plays a significant role in creating neuronal impulses that control movements. As PD severity progresses, the primary motor cortex region continues to deteriorate (Burciu and Vaillancourt, 2018).

Epigenetic modulation by environmental factors is regarded as an important mechanism in the pathogenesis of PD (Pavlou and Outeiro, 2017). A complicated interplay of genetic and environmental factors plays a key role in the etiopathogenesis of the disease (Kalia and Lang, 2015; Ascherio and Schwarzschild, 2016). The epigenome, specifically DNA methylation, has been reported to be influenced by multiple factors such as genome, metabolome, environmental factors, and lifestyle, which modulate the phenotype (Tzika et al., 2018; Czamara et al., 2019). Among them, metabolomic changes are considered to be a direct reflection of the pathological changes in PD (Shao and Le, 2019).

Most tissues, including the brain, undergo expeditious alterations in DNA methylation in early life followed by a steady decline in later life (Prasad and Jho, 2019). This mechanism controls long-term memory formation, aging and the onset of neurodegenerative diseases (Miranda-Morales et al., 2017; Prasad and Jho, 2019). Few studies have demonstrated the molecular link between metabolomics and DNA methylation related to disease pathogenesis (Lu and Thompson, 2012; Kaelin and McKnight, 2013; Tzika et al., 2018), to include neurodegenerative diseases (Wang et al., 2018), and as such the molecular mechanism underlying the impact of metabolites on the epigenome is poorly understood (Gibney and Nolan, 2010; Tzika et al., 2018).

In this study, we hypothesized that the complex regulation of DNA methylation and metabolome leads to deficits that cause irregular motor function among PD subjects and the metabolomic disparities may reflect the effect of these complex interactions. Herein, we aim to integrate quantitative metabolomics and DNA methylation of brain tissue from primary motor cortex (Brodmann area 4) from PD sufferers to better understand the biochemistry associated with the onset of the disease. We believe that understanding this complex relationship may reveal potential therapeutic targets for the treatment of the disease, while testing biomarker panels for the early diagnosis of PD, when potential treatments are believed to be most efficient.

## Results

### Metabolic Dysregulation in Parkinson’s Disease Brain

**FIGURE 1:**
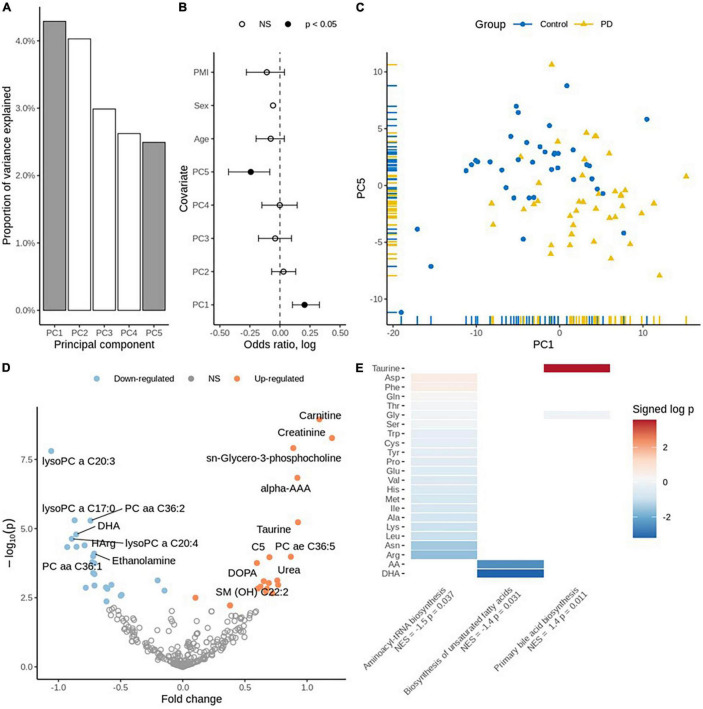
**(A)** Principal component variance: the highlighted two components (PC1 and PC5) were associated with the diagnosis of PD. **(B)** Association of principal components with the diagnosis: binomial regression with five principal components, age, sex, and PMI as covariates. **(C)** Scatter plot displaying PC1 vs. PC5. **(D)** Volcano plot for individual metabolites: Up-regulated metabolites are coded using orange and down-regulated are coded using blue color. **(E)** Enriched pathways of metabolites: Taurine is significantly perturbed on “Primary bile acid biosynthesis,” DHA and Arachidonic acid are significantly perturbed on “Biosynthesis of unsaturated fatty acids”. The other metabolites provided on plot are perturbed to be significant on Aminoacyl *t*-RNA biosynthesis. Normalized Enrichment Score (NES) and the significance (*p*-values) are depicted.

Using ^1^H Nucleic magnetic resonance (NMR) spectroscopy and mass spectrometry (MS), we metabolically profiled post-mortem (PM) brain from people who died from PD and compared them with age-, and sex-matched controls (*N* = 38 controls, 40 PD cases). The demographic information for said samples is available as Table 1 and our analysis shows that there was a slight difference in the post-mortem interval (PMI) between control and PD brain (*P* = 0.03). Of the 78 samples analyzed, none were identified as outliers based on sample distance from the center of either of the first three principal components. Principal component analysis (PCA) highlighted principal components (PC) 1 and 5 as being the most informative, explaining the maximum amount of variation between the two sample groups (binomial regression, *p* = 0.0003 and *p* = 0.005, respectively; Figures 1A–C).

**TABLE 1:** Demographic characteristics of Parkinson’s disease cases vs. cognitively healthy control subjects.

	Cases	Controls	*p*-value
Number of subjects	40	38	n/a
Age in years-Mean (SD)	78 (5.4)	78 (6)	0.46
**Gender**			
Males	20	20	0.09
Females	20	18	
Post-Mortem Interval (PMI) in hours-Mean (SD)	14.6 (4.8)	17.36 (4.3)	0.03

We performed targeted metabolomic profiling and investigated each metabolite using robust regression of diagnosis effect adjusted for age, sex and PMI and identified 48 statistically significantly differentially expressed metabolites in PD PM brain as compared to controls (FDR adjusted *q* < 0.05). While there was no significant trend toward up- or down-regulation (*p* = 0.54, Fisher’s exact test), 27 metabolites were down-regulated, and 21 metabolites were up-regulated Supplementary Table 1. Carnitine, creatinine and taurine were identified among the significantly up-regulated metabolites. Conversely, docosahexaenoic acid (DHA), L-homoarginine and gamma-aminobutyric acid (GABA) were identified among the significantly down-regulated metabolites (Figure 1D and Supplementary Table 1).

Finally, we performed pathway enrichment analysis using the metabolite concentration data and identified three significantly perturbed biochemical pathways. These include Primary bile acid synthesis [normalized enrichment score (NES) = 1.4, *p* = 0.011], Biosynthesis of unsaturated fatty acids (NES = −1.4, *p* = 0.031) and Aminoacyl-tRNA biosynthesis (NES = −1.5, *p* = 0.037) (Figure 1E and Supplementary Table 2).

### Widespread Epigenetic Dysregulation in Parkinson’s Disease Brain

**FIGURE 2:**
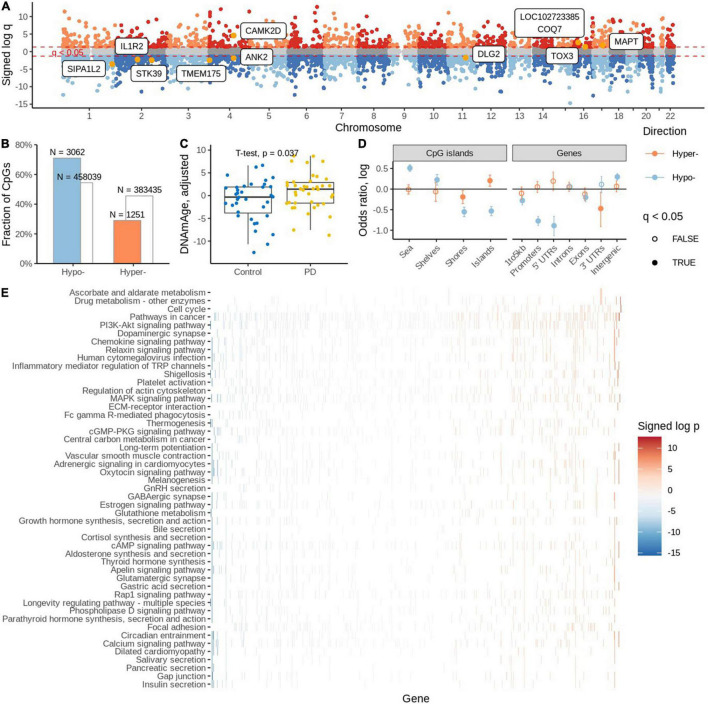
**(A)** Differentially methylated sites with known PD GWAS genes. **(B)** Significantly differentially methylated (hyper and hypo) probes. **(C)** Estimation of DNA methylation age. **(D)** Distribution of significantly differentially methylated cytosines in various genomic areas. **(E)** Estimation of gene set enrichment of KEGG pathways.

Detection of outliers-Two samples had an excess number of missing probe values. On further investigation, one was also more than three standard deviations away from the mean of the first three principal components and, therefore, both were deemed as outliers and removed from further analysis (Supplementary Figures 1a–f). Next, we estimated the proportion of NeuN negative and positive cells in the samples. To identify differentially methylated CpG sites, we fitted a robust linear regression model that accounted for possible batch effects, sample age, sex, and estimated proportion of NeuN positive cells Supplementary Figures 2a–c. We identified statistically significant differential methylation changes (FDR adjusted *q*-value < 0.05) in 4,313 CpGs, among which 3,062 were found to be hypomethylated and the remaining 1,251 hypermethylated (Supplementary Table 3). Some of the differentially methylated sites were in known PD GWAS genes (Figure 2A; Chang et al., 2017).

There was a strong preference toward hypo-methylation in PD PM brain (OR = 1/0.488, *p* = 2.05 × 10e-109, Fisher’s exact test; Figure 2B). We found that among the significant probes, 2001 had an associated SNP. We also confirmed the trend toward hypo-methylation among the non-SNP probes (OR = 1/0.4, *p* = 9.79e-92, Fisher’s exact test).

We employed a DNA methylation clock (Horvath, 2013) to estimate the biological age of cases and controls. We found higher DNA methylation ages associated with the PD group (OR = 1.13, *p* = 0.036, binomial regression adjusted for age, sex, PMI, neuronal proportion). Similarly, we report 2.07 years of aging acceleration among PD cases when compared to controls (*p* = 0.037, *t*-test; Figure 2C).

Subsequently, we evaluated the distribution of significant differentially methylated CpG sites (DMCs) in various genomic areas. We observed that CpG islands are enriched with hyper-modified DMCs but depleted of hypo-modified DMCs (OR = 1.22, *p* = 0.003 and OR = 0.58, *p* = 3.33e-24, respectively, Fisher’s exact test); (Figure 2D and Supplementary Table 4). Interestingly, island shores are depleted of both hyper- and hypo modified DMCs. Going further from the islands, shelves and shores are enriched with hypo-modified DMCs (OR = 1.25, *p* = 0.0006 and OR = 0.57, *p* = 1.01e-24, respectively, Fisher’s exact test). When we analyzed the enrichment of DMCs in relation to gene regions we found no significant enrichment of hyper-modified DMCs, but significant depletion of hypo modified DMCs.

Mapping CpGs on genomic regions and genes showed hypermethylated CpGs are enriched in TsSA (Active TSS, OR = 1.83, *p* = 2.88e-21; Fisher’s exact test) and PromBiv (Bivalent Promoter, OR = 1.54, *p* = 1.68e-05; Fisher’s exact test) followed by enrichment of hypomethylated CpGs in the Tx (Strong transcription) region [OR = 1.65, *p* = 1.16e-03, Fisher’s exact test (Supplementary Table 5)].

We investigated which KEGG pathways were affected by differential methylation. We assigned zero, one or more gene names to each CpG using the EPIC array annotation file from Illumina. For each gene, we determined the signed log *p*-value of the most affected CpGs assigned to that gene which we used to rank the genes and subsequently gene set enrichment analysis to estimate the enrichment of KEGG pathways. We found cell cycle, drug metabolism and ascorbate and aldarate metabolic pathways to be enriched with hyper-modified genes and 47 pathways were enriched with hypo-modified genes (Figure 2E). Among them, the bile acid secretion pathway, the longevity regulating pathway, glutamatergic synapse, glutathione metabolism, GABAergic synapse, the oxytocin signaling pathway, and the dopaminergic synapse pathways were some of the interesting pathways identified.

### Establishing Epigenome-Metabolome Interactions

**FIGURE 3:**
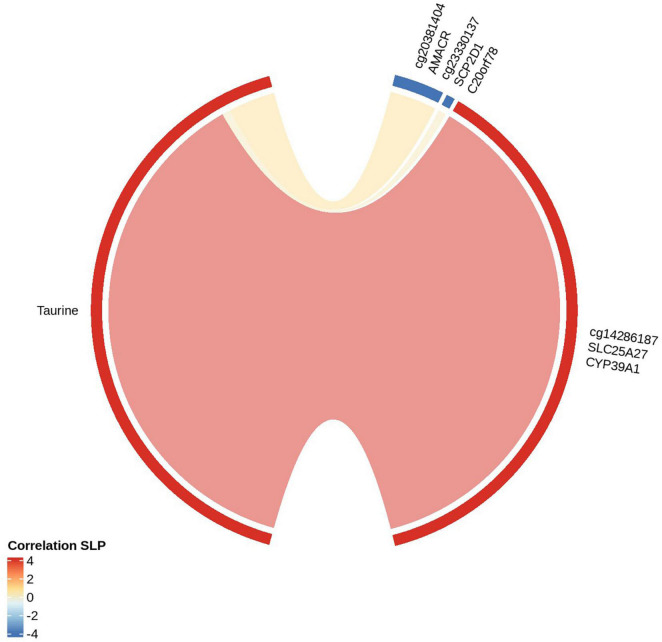
Correlation between significantly differentially methylated cytosines (genes) with metabolites of Primary Bile acid biosynthesis: The red strip around the “circos plot” shows the positive correlation and the blue strip shows the negative correlation. The metabolite correlated with CpGs and the genes encompassed under CpGs are shown.

We established linear relationships between DMCs and differentially expressed metabolites. We further explored the top three metabolic pathways, primary bile acid biosynthesis (Figure 3), biosynthesis of unsaturated fatty acids (Supplementary Figure 3), and aminoacyl-tRNA biosynthesis (Supplementary Figure 4), by mapping the differentially methylated CpGs (genes) to the biochemical pathways. On the top metabolic pathway (Primary bile acid biosynthesis) the significantly perturbed metabolites include taurine (HMDB0000251) and glycine (HMDB0000123). The cg14286187 (methylated on transcription start site 1,500 of *SLC25A27;* 1st exon and 5’UTR of *CYP39A1* transcripts) is correlated positively with taurine and cg23330137 (*SCP2D1*; *C20orf78*) is negatively correlated with taurine. However, glycine is not significant among the differentially expressed metabolites from Brodmann area 4.

### Diagnostic Models

We randomly separated the samples training and validation sets. We trained our Random Forest (RF), LASSO and Ensemble models on a training set and assessed the model using a test set. For the methylome, the Ensemble model achieved an AUC = 0.94 with a sensitivity of 57% and specificity of 88%. The LASSO model had an AUC = 0.91 (57% sensitivity and 100% specificity) and the RF model had an AUC = 0.83 (57% sensitivity and 88% specificity) (Supplementary Figure 5a). For the metabolome, the Ensemble model had an AUC = 0.85 with sensitivity = 86 and 62% specificity. Our LASSO model had an AUC = 0.87 (100% sensitivity and 62% specificity) and our RF had an AUC = 0.89 (71% sensitivity and 75% specificity) (Supplementary Figure 5b).

## Discussion

In the present study, we integrated quantitative metabolomic and genome-wide DNA methylation data from post-mortem (PM) PD brain tissue acquired from the primary motor cortex (Brodmann area 4) and compared them with age-, and sex-matched controls. Among 14,966 samples available on NIH Brain Bank, we studied 78 samples. None of these 78 samples were reported with any neuropsychiatric disorders and a “Genetic diagnosis” was not reported as well. However, using our DNA methylation profile, we checked for SNPs of 290 CpGs encompassing the Parkinson’s disease candidate genes *SNCA, PARK2, PARK7, PINK1*, and *LRRK2.* It is well-known that the SNPs nearby or on CpG sites can influence DNA methylation (Vohra et al., 2020). The 289 out of 290 CpGs were not significant in our study and one CpG (cg20054739) encompassing gene *PARK2* was found to be significant but the SNPs around this CpG has a very low minor allele frequency and would not influence the methylation and probably the metabolome too. Human PM brain tissue is considered to be one of the gold standard biomatrices for understanding the etiopathogenesis of PD and other neurodegenerative diseases (Hartmann, 2004; Tran et al., 2020). However, as previously noted, we did identify a slight difference in the PMI between control and PD brain (*P* = 0.03) and this may have an effect on the DNA methylation pattern (Sjöholm et al., 2018). Fortunately, we have controlled for said effect when conducting our statistical analysis, further information available in the Supplementary Information. While considering the epigenome alone, the results suggest that differential methylation of the PD epigenome may act indirectly on gene expression, for example, through the differential methylation of enhancers (Flam et al., 2019). Overall, the enrichment of gene results recapitulates previously reported epigenome wide studies of PD brain (Masliah et al., 2013; Young et al., 2019; Kia et al., 2021) and indicate widespread epigenetic dysregulation in the prefrontal cortex of PD patients.

### Epigenetic Age

The brain demonstrates a shift in DNA methylation as it ages (Levine et al., 2018; Prasad and Jho, 2019). We observed increased epigenetic age among the PD cases compared to controls though the biological age between the groups was not statistically significantly different. Our results corroborate and confirms the results of an earlier study by Horvath and Ritz (2015) who also report increased epigenetic age in the blood of PD subjects (Horvath and Ritz, 2015).

Our central aim in the study was to understand the interplay and regulatory mechanism between altered metabolism and the differentially methylated genes. Our hypothesis is that by exploring the relationship between metabolites and epigenetic variants we will enhance our understanding of the cellular processes underlying the pathogenesis of PD (Chan et al., 2010; Katada et al., 2012). Pathway analysis using the recorded/detected metabolite concentrations revealed biologically relevant metabolic pathways associated with PD to include, Primary bile acid biosynthesis, Biosynthesis of unsaturated fatty acids and Aminoacyl-tRNA biosynthesis which were found to be perturbed in the brain of PD sufferers. Having assigned the metabolomic data to their respective metabolic pathways, the epigenetic data were subsequently correlated to said pathways.

### Primary Bile Acid Biosynthesis

Our group previously reported perturbed bile acid metabolism in a prodromal mouse model of PD (Graham et al., 2018b). Further, we identified a panel of bile acids in the blood of said model capable of discriminating between prodromal PD and control mice with ∼90% accuracy (Graham et al., 2018a). Another recent study by our group demonstrated disruption to the gut’s microbiome and in particular bile acid metabolism in PD sufferers (Li et al., 2021). In this study, we identified bile acid metabolism using both metabolomics and epigenetics approaches as the top perturbed metabolic pathway in the brain of PD sufferers. In brief, cholesterol metabolism produces bile acids as an end product using the Cytochrome P450 family of enzymes (McMillin and DeMorrow, 2016). The essential bile acids in human brain are, chenodeoxycholic acid and cholic acid (Mano et al., 2004; Kiriyama and Nochi, 2019). The chenodeoxycholic acid is converted to α- and β-muricholic acid and further conjugates with taurine or glycine and freely passes through the blood-brain barrier (BBB) with the help of transporters (Kiriyama and Nochi, 2019). Herein, we were not able to directly measure the concentrations of chenodeoxycholic acid and cholic acid due to their relatively low concentrations in PM brain, however we were able to accurately quantify the expression of taurine (*q* = 0.00026) and glycine (NS). We found taurine to be positively correlated with the methylation locus cg14286187 which is hypermethylated. cg14286187 is located on promoter region of both *CYP39A1* and *SLC25A27*, possibly suppressing their expression.

We recorded taurine at higher concentrations in the PM brain of PD sufferers compared to controls, however it was previously reported to be decreased in the blood plasma of PD patients (Zhang et al., 2016), with neuroprotective properties (Che et al., 2018). Interestingly, the gene correlated with taurine, *CYP39A1* is directly involved in the neural cholesterol clearance pathway of bile acids as identified in PM brain tissues of Alzheimer’s disease cohorts (Baloni et al., 2020). The intronic variants of *CYP39A1* have been reported to be associated with levodopa-induced dyskinesia (Ryu et al., 2020). The abnormal lower expression of *CYP39A1* gene results in buildup of 24S-hydroxycholesterol inducing amyloid-β peptide accumulation in neurodegenerative disease (Matsuoka et al., 2020). Increasing the expression of the CYP39A1 protein has the potential to suppress 24S-hydroxycholesterol aggregation in the brain and act as a therapeutic target for neurodegenerative diseases involving abnormal amyloid-β accumulation such as Alzheimer’s disease (Matsuoka et al., 2020).

*SLC25A27 (UCP4)* was also positively correlated with increased taurine concentration, and it belongs to Solute Carrier (SLC) family of genes. The SLC family of genes generally contribute to cellular influx and efflux of neurotransmitters, metabolites, nutrients, drugs and toxins. However, *SLC25A27* is one of the mitochondrial carriers that protects against oxidative stress and may play a role in calcium regulation, neuronal cell survival with a probable association with organizing the brain’s neuroanatomy (Dahlin et al., 2009; Xu et al., 2018). Studies also observed that the loss of function of one of the PD candidate genes PARK7 (DJ-1) decreases the expression of SLC25A27 (Xu et al., 2018), highlighting the significance of *SLC25A27* and the SLC family of genes as a potential therapeutic targets for the treatment of PD.

Taurine was also found to be negatively correlated with hypomethylated loci at cg23330137 and cg20381404. cg23330137 influences the genes *SCP2D1* on TSS1500, and *C20orf78* on the gene body. The locus cg20381404 influences *AMACR* on 5’UTR and 1st Exons. *SCP2D1* and *C20orf78* have not been reported previously to be involved with neurodegenerative disease. However, *AMACR* genomic variants are associated with relapsing encephalopathy (Smith et al., 2010) and cerebellar ataxia (Dick et al., 2011). Further studies are required to understand their specific role and how they relate to PD pathogenesis.

In this study, regulation of differentially methylated genes involved in bile acid metabolism and how they correlate with taurine were identified by mapping the significantly hypo/hypermethylated genes to the altered biochemical pathway. Our findings support the hypothesis that interactions between the metabolome and the epigenome have significant impact on the pathogenesis of PD. However, our study is not without limitations, as the accurate role of these interactions are unclear, and further functional studies are necessary to better understand their specific biological mechanisms and how they relate to PD pathogenesis.

### Diagnostic Models

Area Under the Receiver Operating Characteristic (AU-ROC) analysis was subsequently performed to determine how well the brain tissue-based methylation and metabolomic markers could discriminate between PD cases and neurologically normal controls. Both sets of data performed well with the Ensemble model created using the methylome data performing best with an AUC = 0.94 and using metabolomics data, the RF model with an AUC = 0.89 outperformed the other models we created using said data. Interestingly, the models created using the methylome data surpassed the diagnostic accuracy of previously reported models (Picca et al., 2019; Wang et al., 2019) while our metabolomics model performed similarly to previous models developed by our group using CSF (Yilmaz et al., 2020).

### Conclusion

Our previous studies demonstrated perturbations in a prodromal mouse model of PD to include both brain (Graham et al., 2018b) and blood (Graham et al., 2018a), while our most recent study reported disturbances in the gut microbiome of PD sufferers (Li et al., 2021). The gut-brain axis has been receiving a lot of attention in recent years, specifically how it relates to neurodegenerative disease (Peterson, 2020; Agus et al., 2021; Varma et al., 2021); herein and for the first time, combining metabolomics and epigenetics approaches, we highlight bile acid metabolism as being the major biochemical pathway to be perturbed in the brain of those people who died from PD as compared with controls. The interplay between these specific methylation changes and their correlating metabolites may have a direct impact on the pathogenesis of PD. However, future translational studies are required to elucidate the specific metabolite-gene interactions and how they relate to the etiopathogenesis of PD. In particular those metabolites and methylation changes related to bile acid metabolism in PD sufferers needs to be further evaluated. Importantly, we highlight several metabolites and CpG sites capable of discriminating between PD and controls with a high degree of accuracy. Our aim is to determine how useful these central biomarkers are in more accessible biomatrices such as blood for diagnosis/prediction of those at greatest risk of developing PD.

## Materials and Methods

### Study Subjects

The present study was approved by the Beaumont Institutional Review board (IRB# 2018-358). A total of 78 brain tissue samples were acquired from the NIH NeuroBioBank among which 40 brain tissue samples were from PD cases and 38 were from cognitively healthy subjects. The post-mortem brain specimens were classified based on diagnosis and pathological findings as laid out in the NIH Brain Bank’s (NBB) webpage.^1^ In brief, subjects contained within the NBB inventory have undergone extensive neuropathological evaluation and have been characterized using all available donor records (e.g., medical records, autopsy reports, family interviews). In general, diagnoses contained within the NBB inventory are classified based on the International Classification of Diseases (ICD-10) coding schema. Parkinson’s disease affected cases were selected considering the clinical brain diagnosis stating, “Parkinson’s disease (Confirmed Diagnosis).” A subject with the absence of a clinical brain, neuropathology, or genetic diagnosis were considered as “unaffected control”. The frozen brain tissues were obtained from the primary motor cortex (Brodmann area 4) (Table 1 and Supplementary Table 6) and No information on an individual patient’s polypharmacy was available from the NIH Neurobiobank.

### Metabolomics ^1^H Nucleic Magnetic Resonance Analysis

Samples were stored at −80°C prior to preparation. Subsequently, samples were lyophilized and milled to a fine powder under liquid nitrogen to limit the amount of heat produced. Using previously optimized methods for ^1^H NMR analysis (Graham et al., 2013, 2014, 2016), 50 mg samples of lyophilized and milled tissue were extracted in 50% methanol/water (1 mg per 10 ul; 0.1 g/mL) in a sterile 2 mL Eppendorf tube. The samples were mixed for 20 min and sonicated for 20 min and centrifuged at 13,000 × *g* at 4°C for 30 min to remove any macro molecules which may affect the NMR signal. Supernatants were collected, dried under vacuum using a Savant DNA Speedvac (Thermo Scientific, Waltham, MA, United States) and reconstituted in 285 μL of 50 mM potassium phosphate buffer (pH 7.0), 30 μL of Sodium 2,2-dimethyl-2-silapentane-5-sulfonate (DSS) and 35 μL of D_2_O. Two hundred microliters of the reconstituted sample was transferred to a 3 mm Bruker NMR tube for analysis. All samples were housed at 4°C in a thermostatically controlled SampleJet autosampler (Bruker-Biospin, Billerica, MA, United States) and heated to room temperature over 3 min prior to analysis by NMR.

Using a randomized running order all 1D ^1^H NMR data were recorded at 300 (±0.5) K on a Bruker ASCEND HD 600 MHz spectrometer (Bruker-Biospin, Billerica, MA, United States) coupled with a 5 mm TCI cryoprobe. For each sample, 256 transients were collected as 64k data points with a spectral width of 12 kHz (20 ppm), using a pulse sequence called CPP WaterSupp (Bruker pulse program: pusenoesypr1d) developed by Mercier et al. (2011) and an inter-pulse delay of 9.65 s. The data collection protocol included a 180-s temperature equilibration period, fast 3D shimming using the z-axis profile of the ^2^H NMR solvent signal, receiver gain adjustment, and acquisition. The free induction decay signal was zero filled to 128k points and exponentially multiplied with a 0.1 Hz line broadening factor. The zero and first order phase constants were manually optimized after Fourier transformation and a polynomial baseline correction of the FID (degree 5) was applied for precise quantitation. All spectra were processed and analyzed using Chenomx NMR Suite (ver. 8.1, Chenomx, Edmonton, AB, Canada).

### Direct Injection/Liquid Chromatography-Mass Spectral Analysis

Brain samples were analyzed using the MXP Quant 500 Kit (BIOCRATES, Life Science AG, Innsbruck, Austria) on an Acquity UPLC I-Class (Waters, Milford, MA, United States) coupled with a Xevo TQ-S (Waters, Milford, MA, United States). Sample preparation and quantification steps were performed according to the manufacturer’s instructions. Briefly, 25 mg lyophilized brain tissue were extracted using 85% ethanol and 15% phosphate-buffered saline solution. 10 μL of the extraction solvent plus seven calibration standards, three quality controls were applied to each spot on a 96-well plate and subsequently dried under nitrogen (Waters, MA, United States). Metabolites were derivatized by incubating the sample for 60 min in 50 μL of phenylisothiocyanate (PITC). Samples were then dried under nitrogen. Next, we extracted the metabolites using 300 μL of methanol containing 5 mM ammonium acetate. Sample extracts were centrifuged at 500 × *g* for 2 min and the supernatant collected. All samples were diluted for both the LC (Liquid Chromatography) and FIA (Flow Injection Analysis) steps. Polar metabolites were separated using a chromatographic method as detailed by the producer (BIOCRATES Life Sciences AG, Innsbruck, Austria) prior to the quantification on a triple-quadrupole mass spectrometer (Xevo TQ-MS, Waters, Milford, MA, United States). All non-polar metabolites were analyzed via direct injection onto the same mass spectrometer. MetIQ software (BIOCRATES Life Sciences AG, Innsbruck, Austria) was used for assay workflow, sample registration, and calculation of metabolite concentrations.

### Genome-Wide DNA Methylation Assay

Genomic DNA was extracted from the lyophilized and milled brain tissue using the QIAamp DNA Mini Kit (Qiagen, Hilden, Germany) and subsequent DNA samples were bisulfite converted using the EZ DNA Methylation-Direct Kit (Zymo Research, Orange, CA, United States) per the manufacturer’s protocol. DNA methylation profiling was performed using the Illumina Infinium MethylationEPIC BeadChip array constituting >850,000 cytosine or “CpG” sites per assay, mapped at single nucleotide resolution covering the various regions of the genome. The BeadChips were processed according to the manufacturer’s recommendations and the fluorescently stained BeadChips were imaged using the Illumina iScan (Illumina, CA, United States).

### Metabolomic Data Analysis

Raw metabolomic data were subjected to sum normalization, autoscaling and subsequently log transformation. Principal Component Analysis (PCA) was performed to identify outliers. A sample that deviates by more than three standard deviations away from the center (i.e., 99.7% of confidence) of any of the first three principal components was considered an outlier. No such outlier samples were identified. A logistic regression model was used to estimate the sample diagnosis based on the first five principal components, sample age, sex and post-mortem interval. Differentially expressed metabolites were identified by fitting a robust linear regression model to each one implemented in R package limma with sample diagnosis, age, sex, and PMI as covariates. *P*-value estimates for diagnosis were obtained after empirical Bayes treatment of fitted models. Metabolites with FDR *q* < 0.05 were deemed significantly differentially expressed in disease.

To estimate metabolite pathway enrichment, the metabolites were ranked by their log transformed *p*-value multiplied by the sign of fold change. That way the metabolites with significant increase in abundance were at the top of the ranked list whereas those with the most significant decrease in abundance were at the bottom of the ranked list. Metabolites without HMDB ID were removed from this ranked list. R package *multiGSEA* was used to download metabolomic pathways from KEGG database and package *fgsea* (fgsea_1.16.0) was then used to compute metabolite set enrichment with 10,000 permutations.

### Methylome EPIC Array Statistical Analysis

Bioconductor *minfi* package was used to read raw *.idat files and mark the failed probes. Two samples had more than 1% of failed probes and one of them was also a PCA outlier defined as above for metabolomics data. The two samples were removed from further analyses. Sex chromosome probes were then removed, and missing values were imputed using Bioconductor *impute* package. Probes that had missing values in more than half of the samples or zero variance were removed. The samples were normalized using Noob normalization. Methylation beta values were extracted for the remaining probes. Sample position effect on the EPIC array was adjusted using *empiricalBayesLM* function from *WGCNA* package. The effect of sample position was modeled as polynomial of second degree. Proportion of neuronal cells in each sample was then estimated using flow sorted PFC samples (Guintivano et al., 2013) and *estimateCellCount* function from *minfi* package. Sample DNA methylation age was estimated using *ENmix* package. Age acceleration was defined as the residuals of a linear model where DNAmAge was the dependent variable and sample chronological age, sex, post-mortem interval, proportion of neuronal cells were the independent variables.

Differentially methylated CpGs were identified through robust linear regression implemented in R package *limma* with sample diagnosis, age, sex, post-mortem interval, proportion of neuronal nuclei as covariates. *P*-value estimates for diagnosis were obtained after empirical Bayes treatment of fitted models. Cytosines with FDR *q* < 0.05 were deemed significantly altered in disease.

### Epigenome Enrichment Analysis

Cytosines were mapped to gene names using *UCSC_RefGene_Name* column specified in Bioconductor EPIC array annotation package *IlluminaHumanMethylationEPICanno. ilm10b4.hg19*. When a CpG locus was annotated with multiple genes and multiple times, the one that is most frequently associated with the locus was chosen. The genes were ranked in the order of significance of affected cytosines multiplied by the sign of fold change. For the genes that mapped to multiple cytosines, the one with the smallest *p*-value was used. In the same fashion as for metabolite set enrichment analysis, the KEGG pathways were downloaded and *fgsea* function was used to estimate the pathway enrichment.

### Establishing Epigenome-Metabolome Interactions

For a selected metabolite pathway, the corresponding metabolites with FDR *q* < 0.05 were chosen. Similarly, for the pathway we identified affected proteins and CpG sites associated with those proteins. The pathway—protein associations were downloaded from SMPDB^2^ (Frolkis et al., 2010). Only differentially modified CpGs were further considered. The concordance of each metabolite – CpG pair was established by fitting a robust linear regression model without intercept where standardized methylation value was a response variable and standardized metabolite abundance as well diagnosis, age, sex, PMI, proportion of neuronal nuclei were the independent variables.

### Evaluation of Diagnostic Models

The same classifier training, testing and validation strategy was chosen for both metabolomic and methylome data. First, the full data was split into 80% of training/testing samples and 20% of validation samples. The training data was subject to preprocessing that removed probes/metabolites of zero and near-zero variance. Then, the data was transformed using principal component analysis. R package *caret* was used to train *glmnet* (LASSO) and *rf* (Random forest) models with ten automatically selected model tuning parameters and area under ROC as the model quality metric. The models were then combined into an ensemble model using *caretEnsemble* package. The accuracy of the models was then evaluated on the validation samples which were processed and transformed in the same way as the training data.

## Data Availability Statement

The datasets presented in this study can be found in online repositories. The names of the repository/repositories and accession number(s) can be found below: GEO, accession number GSE195834.

## Ethics Statement

The studies involving human participants were reviewed and approved by the Beaumont Institutional Review Board (IRB# 2018-358). The patients/participants provided their written informed consent prior to their passing, and/or from next-of-kin who authorize a postmortem donation.

## Author Contributions

SV and UR were involved in the methylation array process and methylation data generation. SA, AY, and ZU were involved in the sample preparation and metabolomic data collection through mass spectrometry. AY performed sample preparation and metabolomic data collection using NMR spectroscopy. JG performed the data analysis. KO and PB contributed for manuscript draft. VL contributed to the data analysis and SG designed the experiment, oversaw the experiments, and provided guidance throughout the project. The manuscript was written by SV and SG and commented on by all authors.

## Conflict of Interest

SG had received commercial support as a consultant from Biogen and Coleman Research. The remaining authors declare that the research was conducted in the absence of any commercial or financial relationships that could be construed as a potential conflict of interest.

## Publisher’s Note

All claims expressed in this article are solely those of the authors and do not necessarily represent those of their affiliated organizations, or those of the publisher, the editors and the reviewers. Any product that may be evaluated in this article, or claim that may be made by its manufacturer, is not guaranteed or endorsed by the publisher.

## Footnotes

1
https://neurobiobank.nih.gov/subjects/

2
https://smpdb.ca
