# Supplementary material for: Methylated Cytochrome P450 and the Solute Carrier Family of Genes Correlate With Perturbations in Bile Acid Metabolism in Parkinson’s Disease
Source: Front Neurosci. 2022 Mar 31;16:804261. doi: 10.3389/fnins.2022.804261 (PMC9009246; doi:10.3389/fnins.2022.804261)
Supplement: Supplementary file 1 [file Data_Sheet_1.PDF]

Supplementary Table ST1: List of all the identified and quantified metabolites in association with PD in brain tissue. Common names are provided for all the metabolites and HMDB IDs are provided for those available on "The Human Metabolome database". The logFC provides the expression status of each metabolite. Positive number being the up-regulated and the negative being the down-regulated metabolite.

Name	HMDB	logFC	AveExpr	t	P.Value	adj.P.Val	B	Contrast
Carnitine	HMDB00062	1.09853471	8.97E-10	6.965131915	1.12E-09	4.42E-07	11.86406874	PD
Creatinine	HMDB0000562	1.200335577	3.85E-10	6.59913777	5.34E-09	1.06E-06	10.3829199	PD
sn-Glycero-3-phosphocholine	HMDB00049	0.888785984	-1.54E-09	6.401540698	1.23E-08	1.56E-06	9.582481789	PD
lysoPC a C20:3	HMDB0010393	-1.058467044	1.28E-10	-6.344049861	1.57E-08	1.56E-06	9.354951076	PD
alpha-AAA	HMDB0000510	0.922049244	-5.13E-10	5.808388782	1.46E-07	1.16E-05	7.233864359	PD
lysoPC a C17:0	HMDB0012108	-0.870261012	2.56E-10	-4.921984473	5.01E-06	0.000260176	3.886654901	PD
PC aa C36:2	HMDB0007979	-0.742105657	1.28E-10	-4.914377626	5.16E-06	0.000260176	3.864616307	PD
SM C16:1		0.997947743	3.85E-10	4.890284999	5.66E-06	0.000260176	3.776974036	PD
Taurine	HMDB0000251	0.926597102	1.28E-09	4.878698093	5.91E-06	0.000260176	3.736805939	PD
DHA	HMDB0002183	-0.857454768	-5.13E-10	-4.61191799	1.62E-05	0.000643431	2.775683287	PD
lysoPC a C20:4	HMDB0010395	-0.892483282	-3.85E-10	-4.513514801	2.34E-05	0.000843457	2.433635378	PD
HArg	HMDB0000670	-0.789749049	-5.13E-10	-4.365039537	4.04E-05	0.001316989	1.934262527	PD
lysoPC a C16:1	HMDB0010383	-0.854703755	-2.56E-10	-4.332006774	4.55E-05	0.001316989	1.818587624	PD
4-Aminobutyrate	HMDB00112	-0.92952772	-7.69E-10	-4.32593944	4.66E-05	0.001316989	1.785490411	PD
Ethanolamine	HMDB00149	-0.712681605	1.28E-10	-4.174351573	8.02E-05	0.002117728	1.284797949	PD
PC aa C36:1	HMDB0007978	-0.720971584	8.97E-09	-4.11207026	0.000100009	0.002378043	1.086403052	PD
PC ae C36:5	HMDB11220	0.869402548	1.28E-10	4.100759715	0.000104075	0.002378043	1.035537144	PD
C5	HMDB0000378	0.69704627	-6.41E-11	4.089995045	0.000108093	0.002378043	1.021440384	PD

AA	HMDB0001043	-0.728978087	3.27E-18	-3.968832608	0.000164939	0.00340959	0.614190195	PD
DOPA	HMDB0000181	0.596253574	2.56E-11	3.950181745	0.000175915	0.00340959	0.56144183	PD
Acetic acid	HMDB00042	-0.710683936	1.41E-09	-3.942218941	0.000180812	0.00340959	0.541082093	PD
PC aa C36:0	HMDB0007886	-0.720511812	-1.28E-10	-3.706585635	0.000401669	0.00723005	-0.216340739	PD
Met-SO	HMDB0002005	-0.710397362	2.56E-11	-3.678377607	0.000441104	0.007594658	-0.290776683	PD
SDMA		0.315522902	-5.13E-11	3.644901088	0.00049269	0.008129388	-0.391187456	PD
TG(18:3_38:6)	HMDB0053582	-0.2019798	1.28E-09	-3.517181483	0.000747209	0.011432639	-0.725184124	PD
Urea	HMDB00294	0.7601706	-1.21E-17	3.5157662	0.000750628	0.011432639	-0.791868452	PD
SM (OH) C22:2	HMDB0013467	0.651419225	4.80E-18	3.496944883	0.000797529	0.011697099	-0.830340352	PD
C18:2	HMDB0006461	0.692004138	-5.13E-10	3.449105863	0.000929541	0.01314636	-0.981500726	PD
Cystine	HMDB0000192	-0.57409442	1.28E-11	-3.402287467	0.001078535	0.014347702	-1.105088844	PD
AMP	HMDB00045	0.767080081	1.28E-11	3.39983	0.001086947	0.014347702	-1.133764125	PD
3-Met-His	HMDB0000479	-0.710332857	-1.28E-11	-3.3836759	0.001143805	0.014611185	-1.178803878	PD
C14:1	HMDB0002014	0.62552956	-2.56E-10	3.353704628	0.001256797	0.015552867	-1.253531046	PD
Putrescine	HMDB0001414	-0.618382915	2.56E-11	-3.335022274	0.001332464	0.015961756	-1.310175583	PD
Glycerol	HMDB00131	-0.780513407	-8.97E-10	-3.326016112	0.001370454	0.015961756	-1.345000297	PD
Cortisol	HMDB00063	0.605004584	-3.85E-10	3.305657662	0.001460117	0.016117868	-1.385761735	PD
C18:1	HMDB0005065	0.677411323	2.56E-10	3.29737222	0.001498164	0.016117868	-1.425835077	PD
PC aa C42:6	HMDB0008288	-0.60674122	-1.15E-09	-3.295698444	0.001505962	0.016117868	-1.430816867	PD
3-Hydroxyisovaleric acid	HMDB00754	-0.147149863	1.28E-11	-3.247965343	0.001745088	0.018185658	-1.535763654	PD
ADP	HMDB01341	0.667962992	-3.85E-11	3.219806505	0.001902405	0.019316726	-1.642731904	PD

C16:1	HMDB0006317	0.721401965	1.28E-11	3.173353614	0.002191277	0.021693644	-1.766489942	PD
Arg	HMDB0000517	-0.492029886	-3.85E-10	-3.132090449	0.002481786	0.023970423	-1.845913768	PD
ADMA	HMDB01539	-0.499526254	-1.82E-18	-3.106741183	0.002677683	0.025246721	-1.937738207	PD
TG(18:0_36:1)	HMDB0045559	0.102206745	3.85E-11	3.051309337	0.003157328	0.02907679	-2.076151203	PD
PC ae C42:3		-0.623309627	2.56E-10	-3.02548242	0.003407084	0.030663757	-2.16370306	PD
DG(17:0_18:1)		-0.706382979	-5.13E-10	-2.984469279	0.00384167	0.033806695	-2.287342422	PD
Xanthine		-0.532934299	-2.56E-10	-2.9636705	0.004081191	0.035133729	-2.320083574	PD
Asn	HMDB0000168	-0.613812574	-1.28E-10	-2.946161183	0.004293472	0.036174782	-2.362071905	PD
TG(20:4_36:3)	HMDB05462	0.380134667	1.15E-09	2.828824922	0.006000915	0.049507551	-2.627109576	PD
lysoPC a C18:0	HMDB0010384	-0.55438953	-2.56E-11	-2.759220626	0.00728901	0.058907103	-2.837220241	PD
lysoPC a C18:1	HMDB0002815	-0.569935836	3.85E-10	-2.718513054	0.008155051	0.064588007	-2.960252371	PD
SM C26:0	HMDB0011698	-0.591371094	1.28E-11	-2.691910792	0.008770728	0.068102124	-3.003792734	PD
PC ae C32:1		0.637305327	5.13E-10	2.683390723	0.008976696	0.068360995	-3.035649386	PD
Hypoxanthine	HMDB0000157	-0.519300196	-1.28E-10	-2.663441247	0.009476311	0.069799036	-3.068570517	PD
TG(16:1_34:1)	HMDB0044889	-0.08810916	-1.28E-10	-2.660879368	0.009542272	0.069799036	-3.035954078	PD
TG(16:0_36:2)	HMDB05369	0.231467345	-1.28E-11	2.649877932	0.009830301	0.069799036	-3.083414425	PD
SM C18:1	HMDB0012100	0.588963205	7.69E-10	2.648363206	0.009870571	0.069799036	-3.119448168	PD
C16	HMDB0000222	0.581298621	-2.56E-10	2.613802888	0.010831083	0.074624945	-3.208450254	PD
TG(17:1_34:3)		-0.097405783	3.85E-11	-2.610406314	0.010929916	0.074624945	-3.142742515	PD
PC ae C42:1		-0.549057768	-2.56E-10	-2.600249057	0.011230368	0.075376708	-3.239027321	PD

p-Cresol-SO4	HMDB0011635	0.543640256	1.28E-10	2.592452353	0.011466049	0.075675926	-3.262930028	PD
Hex2Cer(d18:1/18:0)		-0.496124339	-1.03E-09	-2.57962772	0.011863473	0.077015335	-3.274109923	PD
Propylene glycol	HMDB01881	-0.515487825	1.28E-11	-2.53862404	0.013219134	0.084431891	-3.368361429	PD
PC aa C36:5	HMDB0007984	-0.578093914	1.03E-09	-2.529342458	0.013544768	0.084941943	-3.399953301	PD
C14	HMDB0005066	0.538710396	2.56E-11	2.52420812	0.013727991	0.084941943	-3.403324002	PD
C2	HMDB0000201	0.391734385	-6.41E-11	2.511541088	0.014189609	0.085294774	-3.419790869	PD
TG(20:4_34:1)	HMDB05385	0.423971921	8.97E-11	2.510833697	0.014215796	0.085294774	-3.40349137	PD
lysoPC a C26:0		-0.534629692	1.28E-10	-2.504203264	0.014463365	0.085484965	-3.457729274	PD
TG(18:1_34:1)	HMDB0045740	0.472996301	-3.85E-10	2.460048263	0.016213309	0.094418681	-3.56518074	PD
GTP	HMDB01273	0.461008517	-1.03E-09	2.442196324	0.016973123	0.097122618	-3.57788539	PD
PC ae C38:6	HMDB0013409	0.535497926	2.56E-10	2.429221886	0.017545184	0.097122618	-3.618869898	PD
Acetone	HMDB01659	-0.399094598	1.54E-10	-2.428471779	0.017578779	0.097122618	-3.613468646	PD
Leu	HMDB0000687	-0.48773852	-3.85E-10	-2.423051338	0.017823255	0.097122618	-3.638154298	PD
Serotonin	HMDB0000259	-0.454679654	-5.13E-10	-2.421277505	0.017903917	0.097122618	-3.625758368	PD
C12	HMDB0002250	0.467463119	-3.85E-10	2.410918654	0.018381517	0.098236023	-3.655570095	PD
Cer(d18:0/20:0)	HMDB0011764	-0.095561945	-5.13E-10	-2.406147266	0.018605307	0.098236023	-3.658318576	PD
Carnosine	HMDB0000033	-0.527531196	1.03E-09	-2.39557157	0.019110024	0.099573284	-3.709089796	PD
C5-OH (C3-DC-M)		-0.431798814	5.13E-10	-2.382240439	0.019763619	0.101266386	-3.728508412	PD
Pantothenate	HMDB00210	-0.392299269	1.28E-11	-2.378582457	0.019946409	0.101266386	-3.710426437	PD
SM C24:1	HMDB0012107	0.495804278	-1.67E-10	2.366297374	0.020571366	0.103117226	-3.756675718	PD
Niacinamide	HMDB01406	0.416513161	-3.85E-10	2.341297861	0.021897154	0.106772587	-3.804613147	PD
Lys	HMDB0000182	-0.477282081	-5.13E-11	-2.339639331	0.021987735	0.106772587	-3.805725826	PD

TG(20:4_34:2)	HMDB05391	0.112099546	3.85E-10	2.337419825	0.022109475	0.106772587	-3.787854415	PD
TG(18:3_38:5)	HMDB0050761	-0.085612452	-5.13E-11	-2.327519194	0.022659839	0.108112003	-3.8389692	PD
Cer(d16:1/20:0)		-0.332029685	-3.85E-10	-2.317789055	0.023212525	0.108328043	-3.832332539	PD
Dimethyl sulfone	HMDB04983	0.359354734	1.28E-10	2.317097947	0.023252231	0.108328043	-3.848412965	PD
TG(22:6_34:1)		0.107588914	-2.56E-10	2.300643174	0.024215546	0.111504142	-3.887714461	PD
PC aa C24:0		-0.48415991	-6.41E-10	-2.276217721	0.025710575	0.117027445	-3.948111361	PD
Isoleucine	HMDB00172	-0.454169457	6.05E-18	-2.222336264	0.029299534	0.130807952	-4.055162963	PD
TG(20:5_36:2)	HMDB10464	-0.069777852	-2.56E-10	-2.220930894	0.029398757	0.130807952	-3.992855374	PD
Hex2Cer(d18:1/24:1)		-0.520737901	-7.69E-10	-2.17956013	0.032454754	0.142800919	-4.166454306	PD
PC ae C34:2	HMDB0011151	0.490824515	1.78E-18	2.171553636	0.033077245	0.14394054	-4.176419493	PD
PC ae C40:1		-0.465980273	-1.28E-10	-2.155522557	0.034355034	0.147876017	-4.189743019	PD
Ala	HMDB0000161	-0.437209437	-2.56E-11	-2.142062209	0.035460941	0.150994973	-4.226772825	PD
ATP	HMDB00538	0.457084954	2.56E-11	2.109707636	0.038246752	0.160103598	-4.289246589	PD
PC aa C38:5	HMDB0007989	-0.348563337	3.85E-10	-2.106348976	0.038546539	0.160103598	-4.2723441	PD
CE(16:1)	HMDB0000658	0.200449726	-3.85E-10	2.101534877	0.038979788	0.160103598	-4.233383696	PD
PC ae C40:3		0.481124044	-5.13E-11	2.098915426	0.039217295	0.160103598	-4.317554659	PD
PC aa C38:4	HMDB0007988	-0.402705762	-1.54E-09	-2.081747103	0.040805211	0.163686673	-4.351006885	PD
DG(16:0_16:0)	HMDB0007098	-0.463349987	1.15E-09	-2.080510862	0.040921668	0.163686673	-4.349669808	PD
Cer(d18:1/25:0)	HMDB0004957	0.441010502	7.69E-10	2.072857931	0.04164901	0.164930079	-4.36206233	PD
lysoPC a C18:2	HMDB0010386	-0.471053384	-5.13E-11	-2.05452178	0.043437264	0.170308481	-4.403045763	PD
TG(18:1_36:3)	HMDB05455	0.455007243	-1.28E-10	2.023078587	0.046657964	0.179474576	-4.46588136	PD

DG(14:0_14:0)	HMDB0007008	-0.20469193	7.98E-18	-2.018955148	0.04709517	0.179474576	-4.461402659	PD
lysoPC a C24:0	HMDB0010405	-0.404758051	-3.85E-10	-2.013882854	0.0476378	0.179474576	-4.475160583	PD
PC aa C42:1	HMDB0008059	-0.432501483	2.56E-10	-2.010858307	0.047963908	0.179474576	-4.482786542	PD
PC aa C40:2	HMDB0008276	0.424529406	3.85E-10	2.010144277	0.048041174	0.179474576	-4.464504473	PD
PC ae C30:0	HMDB0013341	0.359313417	1.03E-10	1.997606015	0.049415437	0.181668843	-4.481649028	PD
PC ae C36:3		0.449114616	-5.13E-11	1.996429961	0.049546048	0.181668843	-4.49849221	PD
TG(16:0_32:2)	HMDB05376	-0.112668665	2.56E-09	-1.985841875	0.050735309	0.184322775	-4.431945912	PD
SM C16:0	HMDB0061712	0.486915335	-1.28E-10	1.966455182	0.052976115	0.189391031	-4.581138088	PD
Cer(d18:1/18:0)	HMDB0004950	0.468119671	5.13E-10	1.961821727	0.053523996	0.189391031	-4.583318472	PD
Ile	HMDB0000172	-0.450952598	1.28E-10	-1.9614754	0.05356514	0.189391031	-4.560648508	PD
PC aa C34:1	HMDB0007971	-0.402960716	8.97E-10	-1.95697934	0.054101732	0.189595452	-4.559629658	PD
C3-DC (C4-OH)	HMDB02095	0.38128857	5.13E-11	1.950365266	0.054899418	0.189961805	-4.574270885	PD
Met	HMDB0000696	-0.387053515	-5.13E-10	-1.94817577	0.055165676	0.189961805	-4.578981133	PD
OH-GlutAcid	HMDB0000428	0.337559748	-1.28E-11	1.921993578	0.058435595	0.19948703	-4.640323704	PD
Cer(d16:1/24:0)		-0.431744749	-3.85E-10	-1.915174329	0.059313739	0.200754192	-4.65700964	PD
HexCer(d18:2/18:0)		-0.287317342	-3.85E-10	-1.91100809	0.059855722	0.200871745	-4.640988463	PD
TG(16:0_36:3)	HMDB05384	0.117913451	-5.13E-11	1.905240013	0.060613	0.201703763	-4.672969304	PD
TG(18:1_36:2)	HMDB0005453	0.110755351	1.28E-11	1.875698553	0.064619538	0.213244475	-4.711359717	PD
Cer(d18:2/18:0)		0.301663773	3.85E-10	1.871385451	0.065222754	0.213456287	-4.698469454	PD
lysoPC a C16:0	HMDB0010382	-0.389721018	-2.56E-10	-1.848750586	0.068466583	0.222235794	-4.764943778	PD
CE(17:1)		0.068528332	-6.79E-18	1.838708643	0.069948454	0.2251999	-4.791647244	PD

TG(16:1_30:1)		-0.05777474	9.12E-18	-1.808835467	0.074516359	0.237971599	-4.825380325	PD
Cer(d18:2/18:1)		-0.346426855	-7.47E-18	-1.798740378	0.076115149	0.240262852	-4.853257448	PD
PC aa C26:0		-0.373740482	-1.28E-10	-1.796665472	0.076447271	0.240262852	-4.862066637	PD
His	HMDB0000177	-0.395122534	-1.28E-11	-1.788096737	0.077831628	0.242687597	-4.880350077	PD
TG(18:1_36:4)	HMDB05385	0.329681182	-1.41E-09	1.780219854	0.079122523	0.244785305	-4.894430536	PD
Val	HMDB0000883	-0.406465665	-5.13E-10	-1.77074541	0.080698717	0.247726293	-4.914092669	PD
Asp	HMDB0000191	0.403976427	6.41E-10	1.755021858	0.083371888	0.252412875	-4.948061438	PD
Cer(d18:1/24:1)	HMDB0004953	0.496512582	8.97E-10	1.754277564	0.083500219	0.252412875	-4.954169883	PD
TG(18:2_34:1)	HMDB0045870	0.097819558	1.28E-10	1.744269692	0.085241677	0.25572503	-4.938479552	PD
TG(17:1_32:1)		0.152163433	3.85E-11	1.724288728	0.08880818	0.26175197	-4.937203297	PD
C18	HMDB0000848	0.390399254	7.69E-10	1.723944475	0.088870687	0.26175197	-4.985308516	PD
PC aa C38:6	HMDB0007991	0.424405718	7.69E-10	1.721949522	0.089233626	0.26175197	-4.994138685	PD
O-Acetylcarnitine	HMDB00201	0.220135212	1.28E-10	1.697074258	0.093862248	0.273304781	-5.002720658	PD
Hex2Cer(d18:1/14:0)		-0.353521537	-3.85E-11	-1.681494862	0.096859929	0.279974686	-5.058012956	PD
Cer(d18:0/18:0)	HMDB0011761	-0.372257	-6.41E-10	-1.667168575	0.099685005	0.286052622	-5.082478928	PD
Phe	HMDB0000159	0.279605271	-1.03E-09	1.655855743	0.101962917	0.28859898	-5.070836879	PD
Dimethylamine	HMDB00087	0.362594367	6.41E-10	1.655525994	0.102029943	0.28859898	-5.079366279	PD
Cer(d18:0/24:0)	HMDB0011768	-0.376618702	-3.85E-10	-1.62463116	0.108470135	0.304639529	-5.16263135	PD
Glu	HMDB0000148	-0.253626557	-1.28E-09	-1.610251511	0.111577611	0.309576775	-5.165272219	PD
TG(18:0_36:4)	HMDB05370	0.315537142	2.56E-10	1.60674491	0.112346165	0.309576775	-5.174358223	PD

Cer(d18:1/20:0(OH))		0.377163008	-7.69E-10	1.605711953	0.112573373	0.309576775	-5.183354411	PD
TG(22:5_32:0)		-0.380496321	3.85E-11	-1.599468732	0.113954497	0.31121366	-5.195881081	PD
PC aa C42:0	HMDB0008058	-0.409774571	2.14E-18	-1.582513454	0.117774093	0.319442062	-5.212268568	PD
PC aa C42:2		0.361481603	3.85E-10	1.576045474	0.119257904	0.32126619	-5.226897695	PD
Cer(d18:1/18:1)	HMDB0004948	0.342804917	-2.56E-10	1.569516058	0.12077093	0.32132466	-5.232386235	PD
Cit	HMDB0000904	-0.407535511	1.28E-11	-1.568951534	0.12090246	0.32132466	-5.237846505	PD
Cer(d18:1/14:0)	HMDB0011773	0.275252901	-2.56E-10	1.555871214	0.123982238	0.327233774	-5.24729938	PD
CE(16:0)	HMDB0000885	0.044006134	-1.28E-10	1.552531375	0.124778535	0.327233774	-5.224302224	PD
lysoPC a C28:0		-0.401031238	-5.13E-10	-1.546588357	0.126205549	0.327580948	-5.27630985	PD
PC ae C30:2	HMDB0013410	-0.363597821	-3.85E-09	-1.540736507	0.127623313	0.327580948	-5.276856404	PD
beta-Ala	HMDB0000056	0.341898835	3.85E-11	1.538664039	0.128128442	0.327580948	-5.279018652	PD
TG(16:0_34:2)	HMDB05362	0.191226126	-6.41E-09	1.538289842	0.128219816	0.327580948	-5.269745191	PD
Pyroglutamic acid	HMDB00267	0.245588409	-7.69E-10	1.534579196	0.129128698	0.327788232	-5.257225092	PD
PC ae C34:3	HMDB11211	0.405538776	-2.56E-10	1.530712673	0.130081184	0.32810286	-5.299721593	PD
PC ae C36:2	HMDB11243	0.324754376	5.13E-10	1.521134577	0.132464635	0.331999971	-5.304506876	PD
TG(18:2_36:3)	HMDB05461	0.050381738	-1.49E-17	1.505238439	0.136496301	0.339953051	-5.211005117	PD
TG(14:0_34:0)	HMDB0068866	-0.290765854	-3.85E-11	-1.497594443	0.138469113	0.342711054	-5.333378345	PD
Pro	HMDB0000162	-0.302864513	-5.13E-10	-1.492934559	0.139682719	0.343567434	-5.330346169	PD
PC ae C40:6	HMDB0013422	0.323122595	5.13E-10	1.485870337	0.141538412	0.345982785	-5.345371484	PD
TG(18:1_34:2)	HMDB05384	0.088065381	3.85E-11	1.480937425	0.142845653	0.347036065	-5.370126251	PD
DG(16:0_18:2)	HMDB0007103	0.04181325	-1.15E-09	1.45087007	0.151019033	0.362907295	-5.30192638	PD

Cer(d16:1/23:0)		-0.105604804	-1.03E-10	-1.446695383	0.152182057	0.362907295	-5.390848844	PD
TG(18:1_35:3)		0.378797455	-2.56E-10	1.444795053	0.152713768	0.362907295	-5.421583909	PD
TG(18:1_31:0)		-0.055224443	1.28E-10	-1.443616543	0.153044238	0.362907295	-5.333593568	PD
DG-O(18:2_18:2)		-0.393185717	6.41E-09	-1.422293133	0.159119922	0.373074993	-5.454619507	PD
DG(18:1_18:2)	HMDB0007219	0.049598375	-7.69E-10	1.421959825	0.159216348	0.373074993	-5.385927467	PD
C3	HMDB0000824	0.288017564	3.85E-11	1.411862306	0.162159014	0.376704077	-5.429994055	PD
Cer(d18:2/24:1)		0.308017209	-3.85E-10	1.408011505	0.163292199	0.376704077	-5.462149141	PD
PC ae C42:0	HMDB13443	-0.291820964	2.56E-10	-1.406904982	0.163618943	0.376704077	-5.448094124	PD
Fumaric acid	HMDB00134	-0.291744962	8.90E-20	-1.401347656	0.165267565	0.37753132	-5.454504571	PD
Hex2Cer(d18:1/26:1)		-0.141399287	-2.56E-10	-1.399277393	0.165884974	0.37753132	-5.452796121	PD
PC ae C34:1	HMDB0013426	0.306734271	1.28E-10	1.384902336	0.17022093	0.384658349	-5.483920395	PD
DG(18:0_20:4)	HMDB0007170	0.114507584	4.00E-18	1.379348847	0.171919048	0.384658349	-5.50153325	PD
CE(18:1)	HMDB0005189	0.034786761	2.56E-09	1.379311129	0.171930626	0.384658349	-5.40186697	PD
TG(16:0_32:0)	HMDB0005356	0.327617243	-6.41E-09	1.369119043	0.175080877	0.388408075	-5.508488231	PD
DG(16:0_18:1)	HMDB0007101	0.245778541	-1.28E-10	1.367554593	0.175568296	0.388408075	-5.491514383	PD
HexCer(d18:2/24:0)		-0.347176571	-7.69E-10	-1.35992846	0.177959086	0.39150999	-5.531428734	PD
DG(14:1_18:1)	HMDB0007044	0.062671597	1.28E-09	1.336164833	0.185567564	0.402254446	-5.518921798	PD
SM C26:1	HMDB0013461	-0.294071246	7.47E-18	-1.334213076	0.18620321	0.402254446	-5.535639616	PD
PC aa C36:4	HMDB0007982	-0.280780998	2.56E-10	-1.332342703	0.18681389	0.402254446	-5.539532765	PD
TG(16:0_38:7)	HMDB0063700	-0.042659145	6.41E-11	-1.332060666	0.186906106	0.402254446	-5.518419512	PD
TG(22:5_34:1)		-0.106293891	1.03E-10	-1.316199855	0.192147418	0.411299337	-5.46696136	PD

TG(20:4_36:2)	HMDB05412	0.054493439	-7.69E-10	1.309574456	0.194369189	0.411823367	-5.553739129	PD
PC ae C38:2		0.276651329	-3.85E-11	1.30749929	0.195069019	0.411823367	-5.582833581	PD
PC ae C44:4		-0.30118954	-3.85E-10	-1.306188315	0.195512103	0.411823367	-5.594672962	PD
Cer(d16:1/18:0)		-0.243721818	-1.28E-11	-1.302399285	0.196796955	0.412336477	-5.560526738	PD
TG(20:3_36:3)		-0.088950614	4.85E-18	-1.278799555	0.204941855	0.427141972	-5.59302358	PD
PC aa C40:6	HMDB0008057	0.311940144	1.28E-10	1.269438609	0.208240937	0.431038464	-5.645546428	PD
TG(18:1_32:2)	HMDB05433	0.080734319	5.13E-09	1.265993752	0.209464846	0.431038464	-5.56422866	PD
TG(22:6_32:0)	HMDB10418	0.319795777	-7.69E-09	1.262631004	0.210664701	0.431038464	-5.648533317	PD
PC aa C34:4	HMDB0007883	-0.246226587	3.85E-10	-1.261232168	0.211165308	0.431038464	-5.628867233	PD
TG(14:0_35:2)		-0.352531361	-1.41E-08	-1.250785092	0.214931823	0.436476933	-5.681132498	PD
TG(18:1_33:1)		0.11942529	-2.56E-09	1.241848283	0.218192849	0.438611393	-5.681544852	PD
PC ae C42:4		-0.307807127	-3.85E-10	-1.241833993	0.218198092	0.438611393	-5.688238683	PD
HexCer(d18:2/22:0)		-0.254423064	6.41E-10	-1.237428304	0.219819036	0.439638072	-5.674650145	PD
TG(17:0_32:1)		-0.044078747	8.97E-10	-1.22410952	0.224772869	0.445163724	-5.668585707	PD
O-Phosphocholine	HMDB00284	0.255226562	-5.13E-10	1.221774628	0.225649637	0.445163724	-5.676619445	PD
HCys	HMDB0000742	0.258026424	1.93E-18	1.219764745	0.226406354	0.445163724	-5.689942638	PD
CE(18:3)	HMDB0010370	-0.090257841	-1.28E-10	-1.217983678	0.227078465	0.445163724	-5.598300354	PD
Pyruvic acid	HMDB00243	0.063962387	6.41E-11	1.199543571	0.234122534	0.456711939	-5.707126815	PD
Tyr	HMDB0000158	-0.225218617	1.15E-17	-1.193409519	0.236500385	0.458881208	-5.706509255	PD
Cer(d18:0/24:1)	HMDB0011769	-0.259083858	2.56E-10	-1.190710538	0.23755214	0.458881208	-5.73764519	PD
Cys	HMDB0000574	-0.177228584	6.41E-10	-1.182704336	0.240691864	0.462689215	-5.709178658	PD

DG(18:2_18:2)	HMDB0007248	0.105066939	-5.13E-09	1.173237082	0.244442904	0.467629904	-5.641274138	PD
TG(17:1_36:3)		0.042653421	1.28E-10	1.162600141	0.248707125	0.471114965	-5.704797613	PD
CE(15:1)		0.256665583	1.28E-11	1.160058702	0.249733767	0.471114965	-5.774075525	PD
Acetoacetate	HMDB00060	-0.231275591	3.85E-10	-1.159811734	0.249833694	0.471114965	-5.765506693	PD
Cer(d18:1/26:0)	HMDB0004955	0.305796015	2.56E-10	1.156758547	0.251071404	0.4712051	-5.780604445	PD
Sarcosine	HMDB0000271	-0.239975513	3.85E-10	-1.14103557	0.257514356	0.481017381	-5.770651494	PD
lysoPC a C26:1		-0.284258528	-3.85E-10	-1.135475854	0.259820385	0.48304635	-5.80256167	PD
Spermine	HMDB0001256	0.188973471	7.69E-10	1.116249075	0.267907451	0.494403044	-5.822634548	PD
SM (OH) C16:1	HMDB0013463	0.240614471	-1.28E-10	1.115030477	0.268425895	0.494403044	-5.821446085	PD
Trp	HMDB0000929	-0.281736815	-6.41E-10	-1.10189188	0.274060291	0.502443866	-5.838652048	PD
TG(16:0_35:1)		-0.043324436	8.97E-09	-1.097526114	0.275950642	0.50357813	-5.687052997	PD
PC ae C38:3		0.29547802	2.56E-10	1.091152764	0.278726526	0.50631057	-5.859593358	PD
SM (OH) C14:1	HMDB0013462	0.23350018	1.17E-17	1.07772662	0.284637478	0.514686947	-5.846795137	PD
DG(18:1_18:4)	HMDB0007334	0.13823291	-1.28E-09	1.06191706	0.291707971	0.522703601	-5.819665755	PD
TG(20:3_34:1)		0.067032686	8.97E-11	1.061910681	0.291710848	0.522703601	-5.873079771	PD
lysoPC a C28:1		-0.291888289	5.13E-11	-1.055732148	0.294506547	0.524068203	-5.894997664	PD
Gln	HMDB0000641	0.173248682	-1.28E-10	1.054383508	0.295119216	0.524068203	-5.873231011	PD
PC aa C40:3		0.200681514	-2.56E-10	1.043603857	0.300047565	0.528124834	-5.876170413	PD
HexCer(d18:2/23:0)		-0.248458469	3.85E-10	-1.043553043	0.300070929	0.528124834	-5.899406578	PD
TG(14:0_35:1)		0.091824356	8.97E-10	1.020959998	0.31058154	0.542891372	-5.868107879	PD

CE(22:5)	HMDB0010374	-0.035278517	-5.25E-18	-1.019640667	0.311202882	0.542891372	-5.799479149	PD
Choline	HMDB00097	-0.190526222	2.56E-11	-0.997895687	0.321564227	0.558506289	-5.934334749	PD
Histamine	HMDB0000870	-0.201833359	6.41E-10	-0.984904279	0.327863008	0.566959612	-5.921697408	PD
SM C20:2		0.186800535	1.28E-10	0.969591854	0.335391298	0.577456322	-5.948167298	PD
CE(18:0)	HMDB0062461	-0.040807609	1.03E-10	-0.963176211	0.338579026	0.580421187	-5.971404225	PD
TG(16:0_33:2)		-0.055999737	-8.97E-10	-0.958276038	0.341027088	0.58209796	-5.927958974	PD
PC ae C32:2	HMDB0013411	0.234097119	1.28E-10	0.949566681	0.345406636	0.587043037	-5.987828975	PD
PC ae C42:2		-0.180988326	-8.97E-11	-0.933892064	0.353380434	0.598028426	-5.993229796	PD
DG(18:1_22:5)	HMDB0007236	-0.058735085	-1.56E-19	-0.925630025	0.357630812	0.598389865	-5.9851725	PD
TG(18:1_30:0)		-0.089826791	-3.85E-11	-0.925508571	0.357693538	0.598389865	-5.980106804	PD
DG(16:0_20:3)	HMDB0007111	0.042924651	-1.03E-08	0.924669123	0.358127268	0.598389865	-5.878639123	PD
TG(17:0_36:3)		0.268098127	2.56E-09	0.901978427	0.369978915	0.61559517	-6.04638501	PD
AABA	HMDB0000452	0.196218701	2.56E-11	0.88997725	0.37634668	0.619678641	-6.034227911	PD
PC aa C30:0	HMDB0007869	0.197648769	-2.56E-10	0.888848417	0.376949164	0.619678641	-6.028392892	PD
PC ae C40:2		0.207737735	5.13E-10	0.888514202	0.377127658	0.619678641	-6.030124629	PD
PC aa C40:4	HMDB0008054	0.170383066	2.56E-10	0.881147068	0.381075725	0.620762524	-6.018264285	PD
Cer(d18:1/23:0)	HMDB0000950	0.236545301	-1.28E-11	0.881128775	0.38108556	0.620762524	-6.060613821	PD
CE(20:0)	HMDB0062459	0.037731123	5.13E-11	0.878519587	0.38249004	0.620762524	-6.03137831	PD
PC aa C34:3	HMDB0007974	-0.183629063	1.28E-11	-0.867860711	0.388261082	0.627556688	-6.052108167	PD
Uracil	HMDB00300	-0.178623356	-1.28E-10	-0.862219774	0.391337041	0.629957189	-6.070737964	PD
HexCer(d18:2/20:0)		-0.17995346	1.28E-11	-0.857872583	0.3937178	0.631223679	-6.061614707	PD

Adenine	HMDB00034	-0.114222647	-7.69E-10	-0.847884258	0.399221748	0.63572893	-6.033395217	PD
DG(18:1_20:4)	HMDB0007228	-0.023217458	-1.28E-09	-0.846512212	0.399981472	0.63572893	-6.05479823	PD
TG(16:1_32:1)	HMDB0044888	-0.074868066	-6.41E-11	-0.844055472	0.401344021	0.63572893	-6.050536175	PD
TG(18:1_38:6)	HMDB05462	-0.038995797	7.69E-10	-0.840440961	0.403353851	0.636367031	-6.089208917	PD
CE(15:0)	HMDB0060057	0.187529161	5.13E-10	0.836687396	0.405447498	0.637131783	-6.082302429	PD
TG(18:0_38:6)	HMDB05412	-0.181637561	2.56E-09	-0.830261073	0.40904729	0.640247932	-6.074249383	PD
PC ae C44:6		-0.189476336	8.97E-10	-0.820650351	0.414466939	0.646176802	-6.100390477	PD
C0	HMDB0000062	-0.186933796	1.28E-10	-0.812789936	0.418931608	0.650576144	-6.096927515	PD
TG(18:1_28:1)		0.067753911	-1.28E-10	0.784908813	0.434999096	0.672000722	-6.035193694	PD
Cer(d18:0/26:1(OH))		-0.173727294	-2.56E-09	-0.78298382	0.436121681	0.672000722	-6.099356852	PD
PC aa C42:5	HMDB0008287	-0.164449119	-5.13E-10	-0.778035358	0.43901525	0.673837361	-6.134953379	PD
Orn	HMDB0000214	0.175343574	2.56E-10	0.770099188	0.443679271	0.676279983	-6.129484278	PD
Anserine	HMDB00194	-0.110279993	-1.28E-10	-0.767340096	0.445307507	0.676279983	-6.11564652	PD
TG(18:1_30:1)		0.054258817	3.85E-10	0.763876224	0.44735657	0.676279983	-6.119679492	PD
TG(20:4_32:1)	HMDB05380	0.090593835	8.97E-10	0.761647949	0.448677598	0.676279983	-6.092904399	PD
Cer(d18:2/20:0)		-0.162044869	2.56E-10	-0.760859543	0.449145544	0.676279983	-6.149775655	PD
TG(18:1_26:0)		-0.053834247	7.69E-09	-0.756790672	0.451565042	0.677347563	-6.106212987	PD
PC aa C28:1		0.198031171	1.28E-09	0.749085541	0.456167339	0.678287559	-6.156662268	PD
DG(16:1_18:0)	HMDB0007129	-0.174935996	-5.13E-10	-0.748231842	0.456678907	0.678287559	-6.149537258	PD
TG(18:1_34:4)		0.028189476	2.56E-11	0.747145685	0.457330248	0.678287559	-6.158027971	PD

TG(18:1_38:5)	HMDB05456	-0.193130119	-1.28E-10	-0.739383676	0.46200039	0.682657292	-6.168575967	PD
Cer(d18:1/24:0)	HMDB0004956	0.16803625	3.85E-11	0.724143624	0.471248375	0.693733667	-6.175705475	PD
TG(16:0_33:1)		0.023643293	-5.13E-11	0.718465636	0.474720359	0.696256526	-6.117934584	PD
TG(16:0_36:4)	HMDB05363	0.064127696	-3.85E-10	0.70953768	0.480208506	0.699245943	-6.09204287	PD
D-Galactose	HMDB00143	-0.043298918	2.56E-11	-0.709405306	0.480290143	0.699245943	-6.113226802	PD
GABA	HMDB0000112	0.160989873	3.85E-10	0.696396258	0.488350542	0.70837661	-6.16623497	PD
PC aa C36:3	HMDB0007980	-0.152922965	-7.69E-11	-0.67513086	0.501685302	0.72506343	-6.20205439	PD
TG(18:3_36:3)		-0.033787606	7.69E-10	-0.651819563	0.51652601	0.741044719	-6.153924693	PD
CE(17:0)	HMDB0060059	0.165074881	-1.47E-18	0.649421047	0.51806605	0.741044719	-6.232913435	PD
Ascorbic acid	HMDB00044	0.166881865	-1.28E-11	0.647255419	0.519458636	0.741044719	-6.23211851	PD
SM (OH) C22:1	HMDB0013466	0.177722167	2.31E-18	0.646059719	0.520228363	0.741044719	-6.233708472	PD
PC aa C36:6	HMDB0007892	0.167333984	-2.56E-11	0.637529694	0.525736888	0.746207196	-6.230810833	PD
C6 (C4:1-DC)	HMDB0000705	0.141076891	-3.85E-10	0.620709965	0.536687225	0.759029076	-6.228094203	PD
Cer(d16:1/22:0)		-0.049121466	2.56E-10	-0.612399873	0.542140321	0.761325757	-6.249298615	PD
PC ae C38:4	HMDB0013420	-0.128798277	2.49E-18	-0.612375706	0.542156221	0.761325757	-6.24275283	PD
CE(20:5)	HMDB0006731	-0.070991276	-8.97E-10	-0.603666025	0.54790172	0.766675199	-6.111422298	PD
TG(16:1_32:0)	HMDB05359	-0.199899857	-2.56E-09	-0.598002857	0.551653938	0.768126981	-6.269925965	PD
Trigonelline	HMDB0000875	-0.078610844	-5.13E-10	-0.596248892	0.552818661	0.768126981	-6.218760708	PD
C5-DC (C6-OH)		0.121712465	-8.97E-10	0.585951653	0.559681275	0.774943304	-6.2565448	PD
TG(18:1_36:1)	HMDB0045741	-0.066372746	-2.56E-10	-0.579570027	0.56395538	0.778140525	-6.254332169	PD
TG(20:4_36:4)	HMDB05392	0.017513253	5.13E-10	0.569746504	0.570565892	0.784528101	-6.172031551	PD
TG(20:4_32:0)	HMDB05363	-0.018943331	-8.97E-09	-0.553539724	0.581553495	0.796869149	-6.225303332	PD

TG(18:1_32:0)	HMDB05360	0.0116371	-3.85E-10	0.548703707	0.584851587	0.797870856	-6.257437841	PD
PC ae C40:4		-0.123620659	-1.25E-18	-0.546563228	0.586314189	0.797870856	-6.285392427	PD
DG(14:1_20:2)	HMDB0007051	-0.098798578	3.85E-10	-0.542577808	0.589042052	0.798837851	-6.260961164	PD
CE(18:2)		0.066896842	-1.28E-10	0.533843969	0.595040822	0.802480278	-6.203428725	PD
TG(16:0_28:1)		0.019373872	6.41E-10	0.532769445	0.595780813	0.802480278	-6.164123025	PD
PC ae C44:5		-0.107687158	1.28E-10	-0.524786193	0.601291979	0.804449973	-6.281566797	PD
SM C18:0	HMDB0001348	0.111281322	-7.69E-10	0.524765868	0.60130604	0.804449973	-6.264249984	PD
CE(14:0)	HMDB0006725	-0.014609524	-6.41E-11	-0.518573855	0.605596788	0.805058369	-6.235556332	PD
Cer(d18:1/22:0)	HMDB0004952	0.126621357	2.67E-19	0.51824256	0.605826752	0.805058369	-6.306633924	PD
TG(18:1_35:2)		0.105482898	1.28E-10	0.507986773	0.612965309	0.810434761	-6.301301385	PD
TG(16:1_32:2)	HMDB0005432	-0.022268458	-8.01E-19	-0.506553822	0.613965728	0.810434761	-6.278102535	PD
Methanol	HMDB01875	0.117385923	-3.85E-11	0.500886885	0.61792929	0.812956807	-6.309260487	PD
TG(18:0_36:3)	HMDB05405	0.024075774	-2.56E-11	0.492754928	0.62363679	0.817748903	-6.211401853	PD
N-Acetylaspartate	HMDB00812	-0.102451643	1.15E-09	-0.484618716	0.629370444	0.82254355	-6.312411944	PD
TG(16:0_34:0)	HMDB0063117	0.119012509	2.56E-10	0.472109103	0.638230609	0.828903186	-6.318775026	PD
TG(18:3_34:3)		-0.021609608	-3.85E-10	-0.471838429	0.638422908	0.828903186	-6.252792214	PD
3-Hydroxybutyric acid	HMDB00357	0.062396101	-1.28E-11	0.4649128	0.64335162	0.832572684	-6.260855426	PD
Malonate	HMDB00691	0.043502962	5.13E-10	0.4615854	0.64572533	0.832922576	-6.272314195	PD
TG(16:0_40:7)	HMDB0063773	0.038231544	2.56E-10	0.450060121	0.653975638	0.84082582	-6.277571746	PD
Cer(d18:1/20:0)	HMDB0004951	0.101323225	1.28E-10	0.426099807	0.671265267	0.860262284	-6.345450757	PD

TG(18:3_36:4)	HMDB10490	-0.065576679	2.56E-10	-0.410931963	0.682303533	0.865902497	-6.349790687	PD
PC aa C40:5	HMDB0008055	0.090014599	-5.13E-10	0.410765059	0.682425388	0.865902497	-6.337422221	PD
Cer(d18:2/16:0)		0.064776893	-5.13E-10	0.406495542	0.685545386	0.865902497	-6.337869295	PD
TMAO	HMDB0000925	-0.055959354	2.56E-10	-0.405940253	0.685951573	0.865902497	-6.281822164	PD
TG(18:0_32:0)	HMDB0044753	0.085986241	-1.28E-10	0.405054812	0.686599454	0.865902497	-6.346835999	PD
GLCAS		-0.093465321	1.15E-10	-0.395387106	0.693688559	0.872065617	-6.338612467	PD
DG(16:0_16:1)	HMDB0007099	-0.075855148	8.97E-10	-0.387641885	0.699387787	0.876275068	-6.341316055	PD
Thr	HMDB0000167	0.084095173	1.15E-09	0.383568553	0.702392073	0.876275068	-6.347589382	PD
TG(18:1_33:2)		-0.083707888	-5.13E-10	-0.381830484	0.703675434	0.876275068	-6.344806623	PD
Isobutyric acid	HMDB01873	-0.034180935	-7.69E-10	-0.372794061	0.71036159	0.879356168	-6.291108213	PD
PC ae C36:4		0.082135035	3.85E-10	0.369002085	0.713174144	0.879356168	-6.363385165	PD
TG(18:0_30:0)	HMDB0108000	0.076521709	6.41E-10	0.364764406	0.716321993	0.879356168	-6.365539185	PD
Formate	HMDB00142	-0.066947538	1.79E-10	-0.363333402	0.717386092	0.879356168	-6.351766968	PD
PC ae C38:1		0.075481474	5.13E-11	0.362829117	0.717761213	0.879356168	-6.339506597	PD
PC ae C36:0	HMDB0013406	-0.060631284	-5.13E-10	-0.357575907	0.721673021	0.879356168	-6.302577082	PD
Cer(d18:2/22:0)		0.081395283	-1.28E-10	0.357547995	0.721693825	0.879356168	-6.372879267	PD
Cer(d18:2/24:0)		0.077598653	2.56E-10	0.334927839	0.738621913	0.894629401	-6.37864522	PD
TG(18:2_34:2)	HMDB05390	0.01571015	1.28E-09	0.334761341	0.738747006	0.894629401	-6.314811134	PD
TG(16:0_32:1)	HMDB0044726	-0.027317117	6.41E-11	-0.324844847	0.74621009	0.900912182	-6.347226267	PD
TG(18:0_34:2)	HMDB05369	-0.031525906	3.85E-10	-0.319212343	0.750459969	0.903289202	-6.298725787	PD
PC aa C40:1	HMDB0007993	-0.071960191	3.85E-10	-0.312814422	0.755296757	0.905782941	-6.376669923	PD

TG(18:0_30:1)	HMDB0044726	0.008612246	7.69E-10	0.308197038	0.758793574	0.905782941	-6.282716172	PD
PC aa C38:3	HMDB0008020	-0.067283061	9.96E-18	-0.306685644	0.759939274	0.905782941	-6.377110694	PD
PC aa C32:3	HMDB0007876	0.061287646	1.15E-10	0.304389193	0.76168111	0.905782941	-6.375520846	PD
PC ae C34:0	HMDB0013405	0.07591733	-2.56E-10	0.294958941	0.768846696	0.90651473	-6.371911482	PD
DG(16:1_18:2)	HMDB0007132	0.00854387	-5.13E-09	0.294640468	0.769089044	0.90651473	-6.309004099	PD
Citric acid	HMDB00094	-0.078488261	-1.28E-11	-0.294541956	0.769164013	0.90651473	-6.396054291	PD
DG(18:1_18:1)	HMDB0007218	-0.070195234	-7.96E-18	-0.286178818	0.775536456	0.911312868	-6.390728167	PD
TG(18:1_36:5)	HMDB05441	0.010044174	6.05E-18	0.275449097	0.78373474	0.912531988	-6.360185622	PD
1-Methylhistidine	HMDB00001	-0.067769353	-1.15E-09	-0.272740832	0.785807968	0.912531988	-6.400611797	PD
TG(18:2_34:0)	HMDB05369	-0.054312044	-5.13E-09	-0.272318669	0.786131282	0.912531988	-6.37821213	PD
TG(20:3_36:4)	HMDB10492	-0.066094863	-3.85E-10	-0.271712309	0.78659573	0.912531988	-6.398455033	PD
DG(16:0_20:4)	HMDB0007112	-0.006524698	-1.28E-10	-0.269754568	0.788095808	0.912531988	-6.310478412	PD
3-Hydroxyisobutyrate	HMDB00023	-0.050367699	1.28E-11	-0.258122318	0.797025139	0.919549295	-6.39745719	PD
TG(16:1_36:2)	HMDB05425	-0.010170581	-1.28E-09	-0.253353163	0.800694037	0.919549295	-6.319718436	PD
TG(18:1_38:7)		-0.043706732	5.13E-10	-0.252124414	0.801640041	0.919549295	-6.389852962	PD
Isopropyl alcohol	HMDB00863	-0.022184127	2.56E-11	-0.249781587	0.803444586	0.919549295	-6.37026818	PD
TG(18:1_32:1)	HMDB0062639	-0.007514404	2.56E-10	-0.244310571	0.807662744	0.920060959	-6.38172202	PD
PC ae C44:3		-0.043355597	-7.69E-11	-0.243175741	0.808538419	0.920060959	-6.384135144	PD
Inosine	HMDB00195	0.056055024	6.23E-19	0.230415655	0.81840114	0.925820527	-6.41319823	PD
DG(16:0_20:0)	HMDB0007107	-0.035866173	1.28E-11	-0.228827712	0.819630606	0.925820527	-6.393640078	PD
CE(14:1)	HMDB0062458	0.023157801	-1.28E-11	0.222711289	0.824370455	0.925820527	-6.372983293	PD

TG(18:1_34:3)	HMDB05440	-0.058228234	1.28E-10	-0.219350725	0.826977479	0.925820527	-6.418746354	PD
2-Hydroxyisovalerate	HMDB00407	0.045324627	-3.85E-10	0.214627848	0.83064463	0.925820527	-6.406663944	PD
SM C24:0	HMDB0011697	0.047325563	1.15E-09	0.214409961	0.830813904	0.925820527	-6.389913488	PD
Succinate	HMDB00254	0.028946302	1.28E-11	0.214142894	0.831021395	0.925820527	-6.401780808	PD
t4-OH-Pro	HMDB0000725	-0.046284976	7.07E-18	-0.212493264	0.832303302	0.925820527	-6.410952372	PD
DG-O(16:0_20:4)		0.021384555	-0.000152073	0.200447313	0.841677642	0.93362562	-6.374862669	PD
Cer(d18:1/16:0)	HMDB0004949	0.044166736	-3.85E-10	0.195413195	0.845602146	0.934986176	-6.402071628	PD
DG(18:1_18:3)	HMDB0007221	0.040401076	-3.85E-10	0.189507043	0.850211451	0.934986176	-6.390108586	PD
TG(17:1_34:1)		0.043088636	-2.56E-09	0.187250643	0.85197379	0.934986176	-6.406506127	PD
TG(17:0_34:2)		-0.019524977	6.41E-11	-0.186770999	0.852348509	0.934986176	-6.365438904	PD
CE(22:1)	HMDB0062456	0.01724728	-6.41E-11	0.177220619	0.859816648	0.935595898	-6.331657844	PD
TG(20:1_24:3)		0.036928093	-2.56E-09	0.176202931	0.86061322	0.935595898	-6.409049823	PD
Cer(d18:1/18:0(OH))		-0.006957639	5.13E-10	-0.172384867	0.863603006	0.935595898	-6.383668823	PD
TG(18:2_32:1)	HMDB05379	-0.020516643	-1.78E-18	-0.170597838	0.86500305	0.935595898	-6.352390798	PD
Spermidine	HMDB0001257	0.031333068	-1.28E-11	0.168373254	0.866746499	0.935595898	-6.404580359	PD
TG(16:1_33:1)		-0.025152358	-1.28E-11	-0.167795773	0.867199191	0.935595898	-6.381024934	PD
DG(18:1_22:6)	HMDB0007237	-0.046295998	-1.28E-10	-0.164934708	0.869442653	0.935595898	-6.430059432	PD
PC ae C42:5		0.041091202	3.85E-10	0.155668935	0.876715544	0.939616506	-6.429425212	PD
TG(16:0_35:2)		0.007613246	-2.56E-10	0.154130022	0.877924513	0.939616506	-6.223656154	PD
TG(17:0_34:1)		-0.028756946	-8.97E-10	-0.148274156	0.882527514	0.941997023	-6.401630597	PD

TG(22:5_34:3)		0.005029965	-2.56E-10	0.124798412	0.901019705	0.959150009	-6.368935759	PD
Hex2Cer(d18:1/16:0)		-0.024879536	3.85E-11	-0.119066908	0.905543234	0.959518057	-6.406328906	PD
IMP	HMDB00175	-0.021430686	2.56E-10	-0.118220529	0.906211498	0.959518057	-6.415238939	PD
PC ae C40:5		-0.02547963	-1.03E-17	-0.100372247	0.920318766	0.971636411	-6.433980524	PD
PC ae C30:1	HMDB0013402	-0.019847667	1.28E-10	-0.097218227	0.922814495	0.971636411	-6.40999278	PD
TG(18:1_36:0)	HMDB05381	0.011867848	1.28E-11	0.094434966	0.925017492	0.971636411	-6.392146388	PD
Cer(d18:1/26:1)	HMDB04954	-0.023007969	-8.01E-19	-0.090189444	0.928379019	0.972587543	-6.42819768	PD
Ser	HMDB0000187	-0.018103037	-1.28E-10	-0.081735069	0.935076862	0.976930918	-6.425742393	PD
PC aa C38:0	HMDB0007893	0.017379987	-2.56E-10	0.078729714	0.937458962	0.976930918	-6.408638138	PD
Gly	HMDB0000123	-0.014480719	1.41E-09	-0.070995184	0.943592068	0.980741362	-6.420904998	PD
TG(18:0_34:3)	HMDB05425	-0.002001246	-2.56E-10	-0.055916775	0.955557989	0.98894478	-6.38360338	PD
TG(18:0_38:7)	HMDB0063278	-0.002564787	5.13E-11	-0.052530954	0.958246412	0.98894478	-6.377642704	PD
TG(20:5_34:1)		0.013844992	1.28E-09	0.05161126	0.958976757	0.98894478	-6.441500064	PD
PC ae C38:5	HMDB11253	-0.008070421	1.28E-11	-0.045558406	0.963784275	0.991320969	-6.40016009	PD
Cer(d18:2/23:0)		0.007717434	-1.03E-09	0.035181969	0.972028855	0.997210949	-6.425044144	PD
TG(18:2_32:0)	HMDB05362	-0.002367564	-5.13E-09	-0.028139959	0.977625864	0.99865931	-6.401215937	PD
PC aa C42:4	HMDB0008191	0.005717302	-5.13E-11	0.027059938	0.978484374	0.99865931	-6.417419452	PD
TG(16:1_34:2)	HMDB05379	-0.001595695	-1.28E-11	-0.017411101	0.986155257	0.99997421	-6.369293183	PD
CE(20:4)	HMDB0006726	0.000608612	8.97E-10	0.016706584	0.986715412	0.99997421	-6.361139542	PD
CE(22:6)	HMDB0006733	0.000392842	7.69E-10	0.01478203	0.988245641	0.99997421	-6.355974095	PD

PC aa C32:2	HMDB0007874	-0.002604908	-5.13E-10	-0.012582261	0.989994749	0.99997421	-6.42724556	PD
PC ae C38:0	HMDB0013408	-0.00067945	-3.85E-11	-0.003121703	0.9975176	0.99997421	-6.386273743	PD
Myo-inositol	HMDB00211	-1.91E-05	2.05E-08	-0.001471416	0.998829918	0.99997421	-6.368487242	PD
L-Lactic acid	HMDB00190	-2.57E-06	-3.85E-09	-0.000212094	0.999831341	0.99997421	-6.43536292	PD
Creatine	HMDB00064	-4.01E-07	-4.74E-09	-3.24E-05	0.99997421	0.99997421	-6.41443109	PD

Supplementary Table ST2: Enrichment analysis of the metabolites and associated KEGG pathways: The KEGG pathways and their significance values are provided along with Enrichment Score (ES), Normalized Enrichment Score (NES), observed hits (number of metabolites perturbed) and leadingEdge (HMDB IDs of metabolites) are detailed.

Pathway	P.Value	adj.P.Val	ES	NES	Hits	leadingEdge	Contrast
(KEGG) Primary bile acid biosynthesis	0.01	0.65	0.98	1.38	2	HMDB0000251,HMDB0000123	PD
(KEGG) Biosynthesis of unsaturated fatty acids	0.03	0.65	-0.97	-1.36	2	HMDB0002183,HMDB0001043	PD
(KEGG) Aminoacyl-tRNA biosynthesis	0.04	0.65	-0.64	-1.48	20	HMDB0000517,HMDB0000168,HMDB0000687,HMDB0000182, HMDB0000161,HMDB0000172,HMDB0000696,HMDB0000177, HMDB0000883,HMDB0000148,HMDB0000162,HMDB0000158, HMDB0000574,HMDB0000929	PD
(KEGG) Biosynthesis of amino acids	0.06	0.65	-0.63	-1.42	18	HMDB0000517,HMDB0000168,HMDB0000687,HMDB0000161, HMDB0000172,HMDB0000696,HMDB0000883,HMDB0000148, HMDB0000904,HMDB0000162,HMDB0000158,HMDB0000574	PD
(KEGG) Arachidonic acid metabolism	0.09	0.65	-0.96	-1.28	1	HMDB0001043	PD
(KEGG) Fc gamma R-mediated phagocytosis	0.09	0.65	-0.96	-1.28	1	HMDB0001043	PD

(KEGG) Long-term depression	0.09	0.65	-0.96	-1.28	1	HMDB0001043	PD
(KEGG) Platelet activation	0.09	0.65	-0.96	-1.28	1	HMDB0001043	PD
(KEGG) Vascular smooth muscle contraction	0.09	0.65	-0.96	-1.28	1	HMDB0001043	PD
(KEGG) Glycerolipid metabolism	0.13	0.68	0.94	1.25	1	HMDB0045559	PD
(KEGG) Taurine and hypotaurine metabolism	0.14	0.68	0.90	1.26	2	HMDB0000251,HMDB0000574	PD
(KEGG) Fatty acid degradation	0.16	0.68	0.93	1.23	1	HMDB0000222	PD
(KEGG) Fatty acid metabolism	0.16	0.68	0.93	1.23	1	HMDB0000222	PD
(KEGG) Arginine and proline metabolism	0.18	0.68	-0.68	-1.28	8	HMDB0001414,HMDB0000517,HMDB0000148,HMDB0000162	PD
(KEGG) Inflammatory mediator regulation of TRP channels	0.19	0.68	-0.83	-1.25	3	HMDB0001043,HMDB0000259	PD
(KEGG) Valine, leucine and isoleucine biosynthesis	0.19	0.68	-0.79	-1.26	4	HMDB0000687,HMDB0000172,HMDB0000883	PD
(KEGG) Valine, leucine and isoleucine degradation	0.22	0.68	-0.82	-1.23	3	HMDB0000687,HMDB0000172,HMDB0000883	PD
(KEGG) Serotonergic synapse	0.22	0.68	-0.89	-1.19	1	HMDB0000259	PD
(KEGG) Tyrosine metabolism	0.23	0.68	0.86	1.20	2	HMDB0000181,HMDB0000158	PD
(KEGG) Biotin metabolism	0.27	0.68	-0.87	-1.16	1	HMDB0000182	PD
(KEGG) AGE-RAGE signaling pathway in diabetic complications	0.27	0.68	0.87	1.16	1	HMDB0004957	PD
(KEGG) Insulin resistance	0.27	0.68	0.87	1.16	1	HMDB0004957	PD
(KEGG) Neurotrophin signaling pathway	0.27	0.68	0.87	1.16	1	HMDB0004957	PD
(KEGG) Sphingolipid signaling pathway	0.27	0.68	0.87	1.16	1	HMDB0004957	PD
(KEGG) Lysine degradation	0.29	0.68	0.83	1.16	2	HMDB0000510	PD
(KEGG) Selenocompound metabolism	0.30	0.68	-0.86	-1.14	1	HMDB0000161	PD
(KEGG) Cysteine and methionine metabolism	0.34	0.68	-0.68	-1.14	5	HMDB0000192,HMDB0000696,HMDB0000574	PD
(KEGG) Nicotinate and nicotinamide metabolism	0.35	0.68	0.83	1.11	1	HMDB0000191	PD
(KEGG) Glutathione metabolism	0.37	0.68	-0.63	-1.11	6	HMDB0001414,HMDB0000148,HMDB0000574	PD
(KEGG) Histidine metabolism	0.37	0.68	-0.63	-1.10	6	HMDB0000479,HMDB0000033,HMDB0000177,HMDB0000148	PD

(KEGG) Porphyrin and chlorophyll metabolism	0.40	0.68	-0.78	-1.10	2	HMDB0000148	PD
(KEGG) Metabolic pathways	0.41	0.68	-0.38	-1.03	42	HMDB0001043,HMDB0000192,HMDB0000479,HMDB0001414,HMDB0000517,HMDB0000168,HMDB0011698,HMDB0000157,HMDB0000259,HMDB0000687,HMDB0000033,HMDB0000182,HMDB0000161,HMDB0000172,HMDB0000696,HMDB0000177,HMDB0000883,HMDB0000148,HMDB0000904,HMDB0000162	PD
(KEGG) Circadian entrainment	0.42	0.68	-0.80	-1.06	1	HMDB0000148	PD
(KEGG) Cocaine addiction	0.42	0.68	-0.80	-1.06	1	HMDB0000148	PD
(KEGG) FoxO signaling pathway	0.42	0.68	-0.80	-1.06	1	HMDB0000148	PD
(KEGG) Huntington disease	0.42	0.68	-0.80	-1.06	1	HMDB0000148	PD
(KEGG) Long-term potentiation	0.42	0.68	-0.80	-1.06	1	HMDB0000148	PD
(KEGG) Phospholipase D signaling pathway	0.42	0.68	-0.80	-1.06	1	HMDB0000148	PD
(KEGG) Retrograde endocannabinoid signaling	0.42	0.68	-0.80	-1.06	1	HMDB0000148	PD
(KEGG) Spinocerebellar ataxia	0.42	0.68	-0.80	-1.06	1	HMDB0000148	PD
(KEGG) Taste transduction	0.42	0.68	-0.80	-1.06	1	HMDB0000148	PD
(KEGG) Propanoate metabolism	0.43	0.68	0.79	1.05	1	HMDB0000056	PD
(KEGG) Glycine, serine and threonine metabolism	0.44	0.68	-0.64	-1.07	5	HMDB0000574,HMDB0000271	PD
(KEGG) cAMP signaling pathway	0.44	0.68	-0.76	-1.08	2	HMDB0000259	PD
(KEGG) 2-Oxocarboxylic acid metabolism	0.48	0.69	0.58	1.02	6	HMDB0000510,HMDB0000191	PD
(KEGG) Tryptophan metabolism	0.49	0.69	-0.74	-1.04	2	HMDB0000259,HMDB0000929	PD
(KEGG) Steroid biosynthesis	0.49	0.69	-0.76	-1.01	1	HMDB0010370	PD
(KEGG) Melanogenesis	0.50	0.69	-0.75	-1.00	1	HMDB0000158	PD
(KEGG) Ubiquinone and other terpenoid-quinone biosynthesis	0.50	0.69	-0.75	-1.00	1	HMDB0000158	PD
(KEGG) Thiamine metabolism	0.51	0.70	-0.75	-0.99	1	HMDB0000574	PD
(KEGG) Gastric acid secretion	0.56	0.73	-0.73	-0.97	1	HMDB0000870	PD
(KEGG) Purine metabolism	0.56	0.73	-0.71	-1.00	2	HMDB0000157	PD
(KEGG) Alanine, aspartate and glutamate metabolism	0.59	0.74	-0.53	-0.93	6	HMDB0000168,HMDB0000161,HMDB0000148	PD

(KEGG) Pyrimidine metabolism	0.59	0.74	0.69	0.97	2	HMDB0000056,HMDB0000641	PD
(KEGG) Arginine biosynthesis	0.61	0.76	-0.55	-0.92	5	HMDB0000517,HMDB0000148,HMDB0000904	PD
(KEGG) GABAergic synapse	0.72	0.85	0.64	0.86	1	HMDB0000112	PD
(KEGG) Morphine addiction	0.72	0.85	0.64	0.86	1	HMDB0000112	PD
(KEGG) Carbon metabolism	0.72	0.85	-0.48	-0.81	5	HMDB0000161,HMDB0000148	PD
(KEGG) Sphingolipid metabolism	0.78	0.89	-0.50	-0.76	3	HMDB0011698	PD
(KEGG) Butanoate metabolism	0.78	0.89	-0.57	-0.81	2	HMDB0000148	PD
(KEGG) Glyoxylate and dicarboxylate metabolism	0.84	0.94	-0.43	-0.68	4	HMDB0000148	PD
(KEGG) D-Glutamine and D-glutamate metabolism	0.91	0.95	-0.44	-0.62	2	HMDB0000148	PD
(KEGG) Glutamatergic synapse	0.91	0.95	-0.44	-0.62	2	HMDB0000148	PD
(KEGG) Nitrogen metabolism	0.91	0.95	-0.44	-0.62	2	HMDB0000148	PD
(KEGG) beta-Alanine metabolism	0.91	0.95	-0.35	-0.61	6	HMDB0000033,HMDB0000177	PD
(KEGG) Phenylalanine metabolism	0.95	0.96	0.42	0.59	2	HMDB0000159,HMDB0000158	PD
(KEGG) Phenylalanine, tyrosine and tryptophan biosynthesis	0.95	0.96	0.42	0.59	2	HMDB0000159,HMDB0000158	PD
(KEGG) Pantothenate and CoA biosynthesis	0.96	0.96	0.33	0.52	4	HMDB0000191,HMDB0000056	PD

Supplementary Table ST3: All measured cytosine loci with the influence on genes in association with PD in brain tissue. The logFC defines the methylation status. The positive value shows the hypermethylation and the negative value shows the hypomethylation status of a CpG.

ID	logFC	AveExpr	t	P.Value	adj.P.Val	B	Contrast
cg27265939	-3.24843	-7.64452	-14.0697	2.40E-21	2.03E-15	34.33648	PD
cg05106893	0.542311	3.948114	12.6215	4.24E-19	1.79E-13	30.18427	PD
cg16769976	-1.08189	5.074266	-12.3968	9.68E-19	2.73E-13	29.36459	PD
cg26074202	-1.91845	-6.38791	-12.1143	2.76E-18	4.69E-13	28.74336	PD
cg18148498	-2.56157	5.867556	-12.113	2.78E-18	4.69E-13	28.60728	PD
cg05077452	0.462608	4.906854	11.91507	5.82E-18	8.20E-13	28.2741	PD
cg03182576	-0.93069	4.690859	-11.3726	4.53E-17	3.85E-12	26.3544	PD
cg23963229	1.299261	4.635983	11.37091	4.56E-17	3.85E-12	25.82707	PD
cg08380311	-1.52833	-6.24517	-11.3657	4.66E-17	3.85E-12	26.11459	PD
cg16719517	-0.63385	-6.1985	-11.3551	4.85E-17	3.85E-12	26.58782	PD
cg27475132	0.527874	-5.04601	11.34577	5.02E-17	3.85E-12	26.33702	PD
cg07397185	-0.82594	4.128012	-11.3239	5.46E-17	3.85E-12	26.2574	PD
cg09175811	-0.45446	4.332521	-11.2718	6.67E-17	4.34E-12	26.03219	PD
cg11392855	0.490734	-4.29625	11.17454	9.68E-17	5.85E-12	25.88011	PD
cg01299494	-1.62103	4.616841	-11.1243	1.17E-16	6.62E-12	25.23226	PD
cg07146321	1.50112	4.162969	11.03851	1.63E-16	8.34E-12	24.86762	PD
cg03718736	0.723969	3.545999	11.03192	1.68E-16	8.34E-12	24.85045	PD
cg12088417	-0.85995	4.778443	-10.9292	2.49E-16	1.17E-11	24.74672	PD
cg11179579	1.125408	4.150722	10.91397	2.64E-16	1.18E-11	24.55286	PD
cg18967534	0.918302	-6.4328	10.82546	3.72E-16	1.58E-11	24.38132	PD
cg20832559	-0.52469	4.168949	-10.6579	7.15E-16	2.86E-11	23.98579	PD
cg12482334	-0.61065	5.616095	-10.6364	7.77E-16	2.86E-11	24.06532	PD
cg27307781	-1.08991	-6.29925	-10.6264	8.08E-16	2.86E-11	23.91098	PD
cg23975459	-1.48187	4.790814	-10.6248	8.13E-16	2.86E-11	23.62041	PD

cg10691647	-1.57141	3.541253	-10.5676	1.02E-15	3.44E-11	23.90074	PD
cg10336025	1.122975	4.551051	10.50068	1.32E-15	4.30E-11	23.24467	PD
cg15329552	-0.4667	2.734155	-10.4623	1.53E-15	4.70E-11	23.4272	PD
cg23096297	-0.88846	-5.26715	-10.4586	1.56E-15	4.70E-11	23.04963	PD
cg02655824	-1.32942	4.842537	-10.3986	1.97E-15	5.75E-11	23.02511	PD
cg17329859	-0.34824	4.286122	-10.3415	2.47E-15	6.95E-11	22.98499	PD
cg11133658	-1.55845	4.784071	-10.329	2.59E-15	7.06E-11	22.65356	PD
cg07817055	-0.68368	3.655114	-10.2896	3.02E-15	7.78E-11	22.65438	PD
cg16083558	-0.72597	3.739041	-10.2884	3.04E-15	7.78E-11	22.70285	PD
cg25047753	1.178653	-6.26781	10.26429	3.34E-15	8.31E-11	22.60682	PD
cg10387963	3.916182	5.970849	10.22547	3.89E-15	9.41E-11	22.77563	PD
cg07019285	-0.43232	4.137752	-10.1993	4.32E-15	1.01E-10	22.56628	PD
cg00501815	-0.53135	4.259337	-10.067	7.28E-15	1.66E-10	21.85236	PD
cg19432688	-3.96845	5.389745	-10.039	8.13E-15	1.81E-10	21.94474	PD
cg03796735	1.307073	-5.37087	10.01734	8.86E-15	1.92E-10	21.95494	PD
cg23419210	-0.68363	3.979735	-9.99922	9.52E-15	2.01E-10	21.77041	PD
cg21890736	1.040737	-7.05129	9.93111	1.25E-14	2.57E-10	21.18429	PD
cg00794381	-0.76589	4.384191	-9.91939	1.31E-14	2.63E-10	21.48474	PD
cg17333139	0.832642	3.425929	9.872743	1.57E-14	3.09E-10	21.24777	PD
cg10240127	-1.76303	4.030662	-9.82577	1.90E-14	3.64E-10	21.02387	PD
cg05226593	0.887053	3.956627	9.816619	1.97E-14	3.69E-10	21.1244	PD
cg01228656	0.769189	3.365132	9.810085	2.02E-14	3.71E-10	21.18531	PD
cg20381404	-1.85385	-5.67507	-9.70086	3.12E-14	5.54E-10	20.65329	PD
cg14276772	-2.30217	-6.70487	-9.69602	3.18E-14	5.54E-10	20.88605	PD
cg00269440	-0.86584	4.113356	-9.69341	3.21E-14	5.54E-10	20.8886	PD
cg19477942	0.500958	4.179889	9.665339	3.59E-14	6.08E-10	20.66059	PD
cg09917014	0.638238	4.247105	9.637728	4.01E-14	6.65E-10	20.68352	PD
cg18821137	0.534328	4.720836	9.631198	4.12E-14	6.70E-10	20.56518	PD
cg16138163	-0.58584	3.818238	-9.54008	5.93E-14	9.36E-10	19.8349	PD
cg01751027	-0.44442	4.107858	-9.53806	5.98E-14	9.36E-10	20.18442	PD
cg18985334	-0.60428	3.436391	-9.50847	6.73E-14	1.03E-09	19.93884	PD

cg01918128	1.493733	4.343966	9.488845	7.28E-14	1.08E-09	20.16774	PD
cg26113982	-0.83238	4.087955	-9.48666	7.34E-14	1.08E-09	20.13869	PD
cg09940138	-0.60773	4.591881	-9.48384	7.42E-14	1.08E-09	19.92122	PD
cg12700148	-0.41183	4.67017	-9.47013	7.84E-14	1.12E-09	19.96793	PD
cg00361682	-0.28161	4.222263	-9.43339	9.09E-14	1.28E-09	19.90863	PD
cg00913770	1.106344	3.537441	9.423572	9.45E-14	1.31E-09	19.48026	PD
cg08227826	-0.75262	4.018216	-9.41091	9.95E-14	1.36E-09	19.25717	PD
cg15371675	-0.46258	3.981839	-9.38696	1.09E-13	1.47E-09	19.65049	PD
cg08742404	0.587101	4.34076	9.349723	1.27E-13	1.68E-09	19.55864	PD
cg13283094	-0.83273	4.42784	-9.34549	1.29E-13	1.68E-09	19.5781	PD
cg04852694	-0.45964	3.663451	-9.33674	1.34E-13	1.72E-09	19.53322	PD
cg13236475	-0.72795	5.283057	-9.32475	1.41E-13	1.77E-09	19.07782	PD
cg22179510	1.640339	-6.04364	9.286442	1.64E-13	2.04E-09	19.32387	PD
cg03761891	-0.61414	3.404251	-9.2809	1.68E-13	2.04E-09	19.0811	PD
cg01275521	1.132552	3.276705	9.279057	1.69E-13	2.04E-09	19.24488	PD
cg17208729	0.395971	4.393841	9.270833	1.75E-13	2.08E-09	19.297	PD
cg16515500	1.137966	6.496269	9.229926	2.06E-13	2.42E-09	19.16992	PD
cg20795417	1.172079	4.189238	9.224169	2.11E-13	2.44E-09	18.94764	PD
cg24152907	-0.64346	3.591125	-9.17487	2.57E-13	2.94E-09	18.94041	PD
cg08291129	1.597089	4.167068	9.169351	2.63E-13	2.96E-09	18.98898	PD
cg15060733	-0.70158	4.285909	-9.14587	2.89E-13	3.21E-09	18.69836	PD
cg07007773	0.456382	4.100913	9.137082	2.99E-13	3.29E-09	18.91543	PD
cg24984882	0.770446	3.753751	9.121028	3.19E-13	3.46E-09	18.79131	PD
cg07346920	-0.7638	4.526249	-9.09256	3.58E-13	3.83E-09	18.42021	PD
cg17198361	-0.52247	4.187049	-9.0721	3.89E-13	4.11E-09	18.63192	PD
cg21649277	-1.15355	-4.76199	-9.0637	4.02E-13	4.11E-09	18.55561	PD
cg10195619	0.504326	4.300961	9.063207	4.03E-13	4.11E-09	18.58658	PD
cg17918676	0.675359	4.102967	9.062899	4.04E-13	4.11E-09	18.69552	PD
cg10194279	-0.68747	4.061684	-9.05576	4.15E-13	4.18E-09	18.48699	PD
cg20649848	0.616126	-5.36883	9.052769	4.21E-13	4.18E-09	18.59053	PD
cg24990015	-0.48212	3.699587	-9.0499	4.25E-13	4.18E-09	18.6177	PD

cg22956887	-0.65856	4.54192	-9.02172	4.77E-13	4.63E-09	18.36328	PD
cg27088635	0.669759	-4.95797	9.015501	4.89E-13	4.70E-09	18.19747	PD
cg05373834	-0.85924	3.746191	-9.00029	5.20E-13	4.94E-09	18.34579	PD
cg14042923	-1.51534	4.907916	-8.96016	6.11E-13	5.75E-09	18.05863	PD
cg24020459	-0.4672	4.481754	-8.93528	6.76E-13	6.28E-09	18.16861	PD
cg10032131	-0.82131	-5.18443	-8.93245	6.84E-13	6.29E-09	18.11548	PD
cg01849514	1.245137	5.412029	8.907464	7.57E-13	6.86E-09	17.61001	PD
cg12201190	1.046556	4.434359	8.90573	7.62E-13	6.86E-09	18.00964	PD
cg04287289	1.851432	-5.01307	8.895577	7.94E-13	7.07E-09	17.71521	PD
cg02284441	-0.57282	4.015543	-8.88904	8.15E-13	7.18E-09	18.03118	PD
cg24532093	-0.88266	4.089241	-8.88463	8.30E-13	7.24E-09	17.85516	PD
cg16514866	-0.57405	4.112786	-8.82888	1.04E-12	8.98E-09	17.30014	PD
cg04474990	-0.52825	-3.58458	-8.8233	1.06E-12	9.09E-09	17.77497	PD
cg12578563	-1.37542	6.082369	-8.80592	1.14E-12	9.65E-09	17.56864	PD
cg15038303	-0.68167	4.402186	-8.80113	1.16E-12	9.74E-09	17.52728	PD
cg16697214	-1.11031	-5.94603	-8.79628	1.19E-12	9.84E-09	17.68256	PD
cg04220433	-0.54216	2.891727	-8.79254	1.20E-12	9.89E-09	17.46951	PD
cg08277196	0.644151	-5.36205	8.774285	1.30E-12	1.05E-08	17.55928	PD
cg16313663	0.662174	4.008835	8.774083	1.30E-12	1.05E-08	17.49971	PD
cg26513180	1.244492	-5.24629	8.768644	1.33E-12	1.06E-08	17.29342	PD
cg18118834	-0.61363	4.139326	-8.74914	1.44E-12	1.14E-08	17.46958	PD
cg19139138	-0.5435	3.980605	-8.74173	1.48E-12	1.16E-08	17.44235	PD
cg21305774	0.320193	4.652563	8.735081	1.52E-12	1.18E-08	17.50746	PD
cg16587794	0.352896	-5.34971	8.725635	1.58E-12	1.22E-08	17.40002	PD
cg18085400	0.667336	-5.35854	8.718282	1.63E-12	1.24E-08	17.36967	PD
cg17522207	0.650224	-4.44664	8.696594	1.78E-12	1.34E-08	17.20274	PD
cg05461498	-0.65252	3.882026	-8.69347	1.80E-12	1.34E-08	17.10139	PD
cg27157669	-0.68633	4.333004	-8.6925	1.81E-12	1.34E-08	17.13591	PD
cg05244234	-1.71891	-5.67358	-8.68833	1.84E-12	1.35E-08	17.09108	PD
cg21175760	-0.49324	4.158716	-8.68682	1.85E-12	1.35E-08	17.31584	PD
cg01131790	-0.36822	4.09661	-8.63007	2.33E-12	1.68E-08	17.1215	PD

cg10736303	-0.97366	4.423817	-8.60627	2.57E-12	1.83E-08	16.84129	PD
cg21741679	-0.57731	3.939929	-8.60501	2.58E-12	1.83E-08	16.98211	PD
cg17981101	0.533008	4.052278	8.593869	2.70E-12	1.90E-08	16.82797	PD
cg14366007	-0.80042	2.94633	-8.59118	2.73E-12	1.91E-08	16.91782	PD
cg18757227	0.54005	3.387456	8.589019	2.75E-12	1.91E-08	16.91877	PD
cg25527508	-0.60749	3.770987	-8.54766	3.26E-12	2.24E-08	16.7953	PD
cg17044529	0.99929	3.080981	8.528638	3.52E-12	2.40E-08	16.67047	PD
cg17573813	3.32682	-5.36398	8.525847	3.56E-12	2.41E-08	16.64077	PD
cg12666435	0.931287	4.478922	8.513806	3.74E-12	2.51E-08	16.62672	PD
cg12149758	-0.47455	3.911676	-8.49538	4.03E-12	2.65E-08	16.51753	PD
cg12452792	-0.71513	4.389041	-8.49527	4.03E-12	2.65E-08	16.62135	PD
cg06765813	0.556271	4.41179	8.493983	4.05E-12	2.65E-08	16.53393	PD
cg22069745	-0.62084	3.948843	-8.4766	4.35E-12	2.83E-08	16.42541	PD
cg21423504	-0.52991	4.656118	-8.45459	4.75E-12	3.03E-08	16.43594	PD
cg09397997	0.710645	3.497766	8.45392	4.77E-12	3.03E-08	16.30859	PD
cg17255085	-0.40094	3.728657	-8.45376	4.77E-12	3.03E-08	16.42194	PD
cg26903052	0.795294	3.779328	8.452502	4.79E-12	3.03E-08	16.42515	PD
cg25721040	1.332309	4.118093	8.447156	4.90E-12	3.06E-08	16.2559	PD
cg17246297	-0.48549	3.142137	-8.44439	4.95E-12	3.06E-08	16.21094	PD
cg13887095	-0.83868	5.751392	-8.44358	4.97E-12	3.06E-08	16.41181	PD
cg21445827	-0.57033	3.980775	-8.44281	4.99E-12	3.06E-08	16.33137	PD
cg17717333	1.011103	-4.67901	8.439382	5.06E-12	3.08E-08	16.21792	PD
cg06503981	1.626505	3.945028	8.410713	5.68E-12	3.43E-08	16.08084	PD
cg03132864	-0.40855	3.678682	-8.40942	5.71E-12	3.43E-08	16.2284	PD
cg00156928	-0.51342	4.332486	-8.38142	6.40E-12	3.81E-08	15.88811	PD
cg23237621	0.489372	4.073441	8.37897	6.46E-12	3.82E-08	15.91224	PD
cg05080777	-0.60641	3.015324	-8.36403	6.87E-12	4.04E-08	15.92399	PD
cg14145602	-0.54087	3.882537	-8.34543	7.41E-12	4.32E-08	15.97225	PD
cg03096819	1.036585	3.765462	8.337307	7.66E-12	4.44E-08	16.02068	PD
cg09501102	0.674657	3.959566	8.333407	7.78E-12	4.48E-08	15.82685	PD
cg22877644	-0.47428	4.508938	-8.31903	8.25E-12	4.72E-08	15.87293	PD

cg09397187	-0.59222	3.64236	-8.31474	8.40E-12	4.75E-08	15.91551	PD
cg26303616	0.805038	-4.528	8.312276	8.48E-12	4.75E-08	15.74477	PD
cg25003343	-1.0906	-5.10868	-8.31227	8.48E-12	4.75E-08	15.76452	PD
cg05130075	0.486545	3.613535	8.310369	8.55E-12	4.76E-08	15.92712	PD
cg22554448	0.802942	-4.78291	8.300972	8.88E-12	4.89E-08	15.88295	PD
cg06280307	-0.73673	3.612826	-8.30027	8.91E-12	4.89E-08	15.67544	PD
cg13585334	-0.39259	3.944624	-8.2957	9.07E-12	4.95E-08	15.83436	PD
cg02243946	0.521236	3.69927	8.280844	9.64E-12	5.23E-08	15.79414	PD
cg19913815	-0.66953	4.37554	-8.26902	1.01E-11	5.45E-08	15.47423	PD
cg25744355	2.653971	6.209387	8.267478	1.02E-11	5.45E-08	15.77722	PD
cg10151583	-0.8351	3.01208	-8.25592	1.07E-11	5.67E-08	15.555	PD
cg04662079	-0.59763	3.784506	-8.25475	1.07E-11	5.67E-08	15.56462	PD
cg21992044	0.309903	4.082448	8.233093	1.17E-11	6.15E-08	15.63484	PD
cg24435986	-0.49595	-4.71905	-8.22573	1.21E-11	6.30E-08	15.60621	PD
cg02024240	0.554971	3.543517	8.223396	1.22E-11	6.32E-08	15.58301	PD
cg26787320	0.392632	4.149709	8.204957	1.31E-11	6.75E-08	15.51904	PD
cg11000786	-0.57146	4.156842	-8.20417	1.32E-11	6.75E-08	15.40441	PD
cg22223109	0.4778	4.264219	8.201429	1.33E-11	6.79E-08	15.53309	PD
cg07078747	0.424562	3.812614	8.185886	1.42E-11	7.18E-08	15.49276	PD
cg01347966	0.615376	3.913514	8.184618	1.43E-11	7.18E-08	15.47411	PD
cg21342300	-0.44073	4.052742	-8.17844	1.46E-11	7.27E-08	15.47177	PD
cg23945952	-1.53811	6.599818	-8.17797	1.47E-11	7.27E-08	15.33116	PD
cg05603011	-0.45196	3.90224	-8.17721	1.47E-11	7.27E-08	15.34646	PD
cg21371986	0.617801	4.107645	8.175807	1.48E-11	7.27E-08	15.25398	PD
cg18493115	-0.70199	3.037949	-8.172	1.50E-11	7.32E-08	15.36202	PD
cg21266314	0.658958	-4.96939	8.171237	1.51E-11	7.32E-08	15.32338	PD
cg09245589	0.721026	-6.25876	8.168907	1.52E-11	7.35E-08	15.36853	PD
cg20839522	0.852126	5.818046	8.157337	1.59E-11	7.66E-08	15.40946	PD
cg11154534	-1.36176	3.340585	-8.15608	1.60E-11	7.66E-08	15.22139	PD
cg26146888	-0.55668	4.110926	-8.15089	1.64E-11	7.78E-08	15.29132	PD
cg17669549	-0.47503	3.650991	-8.13373	1.76E-11	8.29E-08	15.24053	PD

cg17466567	-0.69242	-3.25587	-8.11431	1.90E-11	8.93E-08	15.15101	PD
cg16344227	-1.54746	4.397829	-8.10566	1.97E-11	9.20E-08	15.09213	PD
cg18697348	1.283601	-5.87659	8.102549	1.99E-11	9.26E-08	15.20486	PD
cg24413826	-0.92862	4.193413	-8.09425	2.06E-11	9.52E-08	14.90734	PD
cg20033239	-0.31747	3.351137	-8.09322	2.07E-11	9.52E-08	14.99475	PD
cg07397612	-0.72496	3.917644	-8.09096	2.09E-11	9.56E-08	15.17087	PD
cg17018201	-0.65458	3.815994	-8.05909	2.38E-11	1.08E-07	14.94009	PD
cg01934420	-0.46179	3.790601	-8.05464	2.42E-11	1.10E-07	14.96922	PD
cg25446460	-0.4947	3.860735	-8.04685	2.50E-11	1.13E-07	14.92713	PD
cg00221969	0.619035	-4.55791	8.041892	2.55E-11	1.14E-07	14.89482	PD
cg04130508	-0.42199	3.998317	-8.03856	2.59E-11	1.15E-07	14.90873	PD
cg06340512	-0.84104	4.341134	-8.03178	2.66E-11	1.18E-07	14.89671	PD
cg10994425	0.511857	-4.64667	8.018698	2.81E-11	1.24E-07	14.74128	PD
cg05099240	-0.68531	4.172588	-8.01409	2.86E-11	1.25E-07	14.65735	PD
cg11547898	-0.68749	2.807185	-7.99507	3.09E-11	1.35E-07	14.59711	PD
cg00387872	0.867727	3.909646	7.977858	3.31E-11	1.44E-07	14.60852	PD
cg15954263	0.674525	3.704952	7.971512	3.40E-11	1.47E-07	14.53258	PD
cg24265957	-0.64487	3.068652	-7.96852	3.44E-11	1.47E-07	14.62416	PD
cg13210813	-0.56645	3.009179	-7.96722	3.46E-11	1.47E-07	14.63612	PD
cg02593734	-0.28995	3.609212	-7.96722	3.46E-11	1.47E-07	14.65155	PD
cg12513911	-1.13342	-4.55246	-7.95826	3.59E-11	1.52E-07	14.63158	PD
cg22548845	-0.59008	4.166236	-7.94997	3.71E-11	1.56E-07	14.61469	PD
cg01244074	-0.37629	4.383167	-7.94832	3.74E-11	1.57E-07	14.62838	PD
cg02564302	0.301123	4.037201	7.945418	3.78E-11	1.58E-07	14.55749	PD
cg20637688	-0.72038	3.972191	-7.94247	3.83E-11	1.59E-07	14.5013	PD
cg18792536	-0.59614	-4.90295	-7.93057	4.02E-11	1.66E-07	14.51382	PD
cg06588010	0.578496	3.83286	7.922947	4.15E-11	1.70E-07	14.39909	PD
cg23283320	0.522535	-4.99495	7.913047	4.32E-11	1.76E-07	14.33382	PD
cg09948538	1.237329	3.879697	7.902005	4.52E-11	1.84E-07	14.35976	PD
cg05803631	-0.867	-5.70772	-7.90005	4.55E-11	1.84E-07	14.40565	PD
cg05303549	0.389768	3.667309	7.886873	4.80E-11	1.94E-07	14.31234	PD

cg18086678	0.53571	3.864726	7.883872	4.86E-11	1.95E-07	14.28953	PD
cg27210166	-0.74571	5.931235	-7.87923	4.96E-11	1.98E-07	14.17172	PD
cg04549802	-0.47977	3.947621	-7.86798	5.19E-11	2.06E-07	14.20825	PD
cg06136459	-0.49753	4.288916	-7.86272	5.30E-11	2.10E-07	14.21111	PD
cg15654551	0.628193	4.412428	7.861377	5.33E-11	2.10E-07	14.21382	PD
cg26152108	-0.30673	3.754538	-7.85527	5.47E-11	2.14E-07	14.27411	PD
cg26220323	-0.51526	3.89149	-7.84956	5.59E-11	2.18E-07	14.21002	PD
cg17161088	-0.22266	3.64701	-7.84463	5.71E-11	2.21E-07	14.11379	PD
cg00209612	-0.75614	3.813203	-7.84424	5.72E-11	2.21E-07	14.10636	PD
cg25868285	0.758064	4.394762	7.841156	5.79E-11	2.23E-07	14.12847	PD
cg03246849	-0.80684	4.119323	-7.83304	5.98E-11	2.29E-07	14.04462	PD
cg23669159	1.007209	-5.81604	7.820025	6.31E-11	2.40E-07	14.00133	PD
cg07805959	-0.5254	5.631894	-7.81912	6.33E-11	2.40E-07	14.07329	PD
cg02208776	-0.7275	-6.35176	-7.8171	6.39E-11	2.41E-07	14.14701	PD
cg22230293	-0.65769	3.986998	-7.78965	7.14E-11	2.69E-07	13.97719	PD
cg24861013	-0.48874	4.687506	-7.77497	7.58E-11	2.84E-07	13.98458	PD
cg22427279	-0.73846	3.384213	-7.77083	7.71E-11	2.87E-07	13.75338	PD
cg19678070	0.895536	-5.43482	7.761534	8.01E-11	2.96E-07	13.73225	PD
cg22866218	-1.95367	3.746421	-7.76137	8.02E-11	2.96E-07	13.88805	PD
cg11383659	-0.41639	4.229338	-7.76051	8.05E-11	2.96E-07	13.83432	PD
cg11674404	-1.36426	-5.5795	-7.74896	8.43E-11	3.09E-07	13.69621	PD
cg03744954	0.460494	-5.10209	7.742256	8.67E-11	3.16E-07	13.72606	PD
cg02703850	-0.64239	5.420854	-7.74021	8.74E-11	3.17E-07	13.83455	PD
cg17422692	-1.55968	-5.44166	-7.72748	9.21E-11	3.33E-07	13.66495	PD
cg19657945	-0.62345	5.560658	-7.71798	9.57E-11	3.44E-07	13.7734	PD
cg21997403	0.84584	4.191791	7.716341	9.63E-11	3.45E-07	13.66744	PD
cg08597019	-0.3927	4.517192	-7.70293	1.02E-10	3.63E-07	13.66864	PD
cg17869152	-0.26676	4.275952	-7.7003	1.03E-10	3.66E-07	13.67698	PD
cg19876216	-0.33837	4.213611	-7.69795	1.04E-10	3.66E-07	13.71258	PD
cg18434356	-0.82374	3.199985	-7.69763	1.04E-10	3.66E-07	13.61579	PD
cg06176987	-0.65923	3.923987	-7.68737	1.08E-10	3.81E-07	13.52505	PD

cg22785503	0.600672	4.261711	7.669777	1.16E-10	4.07E-07	13.55431	PD
cg04725837	-0.37927	4.577953	-7.66433	1.19E-10	4.15E-07	13.50462	PD
cg00387731	-1.1281	-4.57707	-7.66256	1.20E-10	4.16E-07	13.50039	PD
cg14164492	0.487403	4.562761	7.659118	1.22E-10	4.20E-07	13.47998	PD
cg12263200	0.515381	3.540863	7.646082	1.28E-10	4.40E-07	13.51346	PD
ch.1.194404001R	1.6412	-4.48798	7.644799	1.29E-10	4.40E-07	13.43374	PD
cg18261836	-0.34152	3.441569	-7.64458	1.29E-10	4.40E-07	13.5055	PD
cg14454577	-0.49144	3.877615	-7.63959	1.32E-10	4.48E-07	13.42391	PD
cg16990132	-0.44934	3.454448	-7.63674	1.33E-10	4.51E-07	13.47376	PD
cg10235702	-0.5305	2.616553	-7.62002	1.43E-10	4.81E-07	13.28382	PD
cg06323483	0.74795	4.369833	7.617324	1.44E-10	4.84E-07	13.36668	PD
cg05788548	0.497699	4.347026	7.614193	1.46E-10	4.89E-07	13.384	PD
cg13285893	2.136352	-6.09849	7.607632	1.50E-10	5.00E-07	13.06305	PD
cg23571474	0.992904	-5.28674	7.598045	1.56E-10	5.18E-07	13.16145	PD
cg03954425	1.401759	-5.47512	7.593031	1.59E-10	5.26E-07	13.14653	PD
cg15482025	0.550418	4.204147	7.588498	1.62E-10	5.34E-07	13.22712	PD
cg11563656	-0.54381	3.762197	-7.58763	1.63E-10	5.34E-07	13.19782	PD
cg18212915	-0.26773	3.640364	-7.57741	1.70E-10	5.53E-07	13.24766	PD
cg07520611	-0.323	3.834175	-7.57657	1.70E-10	5.53E-07	13.25939	PD
cg19982221	-0.65671	3.607223	-7.57618	1.71E-10	5.53E-07	13.17667	PD
cg11584684	-0.47508	3.79782	-7.56668	1.77E-10	5.73E-07	12.95842	PD
cg14863544	-0.47054	5.422358	-7.55951	1.83E-10	5.87E-07	13.16086	PD
cg10195889	0.678703	3.846731	7.556118	1.85E-10	5.93E-07	13.11384	PD
cg06636485	0.942058	3.763603	7.550286	1.90E-10	6.04E-07	13.07016	PD
cg12187434	-0.61686	4.011295	-7.54978	1.90E-10	6.04E-07	13.08257	PD
cg02006950	-0.38277	3.32315	-7.54279	1.96E-10	6.19E-07	13.07276	PD
cg04439006	0.502364	4.417243	7.541424	1.97E-10	6.21E-07	13.11971	PD
cg00180722	-0.43228	4.563192	-7.53968	1.98E-10	6.23E-07	12.94031	PD
cg20353088	-0.3293	3.834546	-7.53497	2.02E-10	6.32E-07	12.98942	PD
cg03393996	-0.67452	-5.54293	-7.53255	2.04E-10	6.36E-07	12.94476	PD
cg20925233	-0.68202	3.401323	-7.51772	2.17E-10	6.73E-07	12.91592	PD

cg07569336	-1.68503	4.366957	-7.51167	2.22E-10	6.88E-07	12.91083	PD
cg18785380	-0.25354	4.025246	-7.50423	2.29E-10	7.06E-07	12.9245	PD
cg15691416	0.786204	3.847095	7.500818	2.32E-10	7.14E-07	12.9914	PD
cg00573148	-0.50837	3.917633	-7.49858	2.34E-10	7.16E-07	12.93035	PD
cg15797903	-0.36578	4.379577	-7.49837	2.34E-10	7.16E-07	12.9664	PD
cg26639431	-0.47685	4.245331	-7.49332	2.39E-10	7.28E-07	12.89094	PD
cg25169180	2.184059	6.217188	7.489666	2.43E-10	7.36E-07	12.92787	PD
cg12765935	-0.57115	3.745214	-7.47792	2.55E-10	7.69E-07	12.65333	PD
cg19734801	-0.49521	5.310331	-7.46994	2.63E-10	7.92E-07	12.8335	PD
cg25613667	-1.09264	-4.56686	-7.46758	2.66E-10	7.97E-07	12.7605	PD
cg00686450	-0.33113	4.401791	-7.46511	2.68E-10	8.02E-07	12.66254	PD
cg07530460	0.508936	4.187349	7.461533	2.72E-10	8.11E-07	12.73861	PD
cg23928234	-0.41657	4.359834	-7.45331	2.82E-10	8.36E-07	12.81721	PD
cg22361409	-0.32188	2.991874	-7.44486	2.91E-10	8.62E-07	12.65182	PD
cg00988148	-0.84082	-7.34947	-7.44061	2.97E-10	8.73E-07	12.71073	PD
cg19192106	0.747553	3.965541	7.439999	2.97E-10	8.73E-07	12.57397	PD
cg26939204	0.437802	4.512406	7.423949	3.17E-10	9.29E-07	12.7204	PD
cg23470812	-0.45374	3.932806	-7.42238	3.19E-10	9.32E-07	12.71999	PD
cg01787644	-0.26299	-6.05653	-7.41532	3.29E-10	9.56E-07	12.59106	PD
cg15227302	-1.03032	-5.39311	-7.41395	3.31E-10	9.58E-07	12.51986	PD
cg17373649	-0.71858	5.0329	-7.40966	3.36E-10	9.71E-07	12.59372	PD
cg02001099	-0.83266	6.045509	-7.40773	3.39E-10	9.72E-07	12.56392	PD
cg14168563	-0.63527	4.463602	-7.40772	3.39E-10	9.72E-07	12.48514	PD
cg20696049	-0.27218	3.63128	-7.40444	3.44E-10	9.82E-07	12.60864	PD
cg10966873	-0.45427	5.651915	-7.39909	3.51E-10	1.00E-06	12.62119	PD
cg05906698	-0.76264	3.891377	-7.3913	3.63E-10	1.03E-06	12.58205	PD
cg12466675	0.399066	3.853031	7.391229	3.63E-10	1.03E-06	12.56151	PD
cg23889112	0.957194	-5.41595	7.383864	3.74E-10	1.05E-06	12.53627	PD
cg01714268	0.515603	3.906712	7.3766	3.85E-10	1.08E-06	12.31484	PD
cg26447514	-0.75101	4.295574	-7.36363	4.06E-10	1.14E-06	12.42047	PD
cg13117582	1.261854	4.938697	7.340417	4.46E-10	1.25E-06	12.1976	PD

cg14020943	-1.04731	5.733897	-7.33868	4.49E-10	1.25E-06	12.35251	PD
cg09152224	0.469294	3.936406	7.33729	4.52E-10	1.25E-06	12.34707	PD
cg14574773	-0.62486	4.075227	-7.33385	4.58E-10	1.27E-06	12.37686	PD
cg08669938	-0.61753	-6.16348	-7.33052	4.64E-10	1.28E-06	12.36572	PD
cg25871594	-0.32842	3.698774	-7.32891	4.67E-10	1.28E-06	12.20286	PD
cg22403800	0.396645	4.350497	7.317464	4.90E-10	1.34E-06	12.24095	PD
cg03886486	-0.3455	3.803905	-7.314	4.97E-10	1.36E-06	12.28669	PD
cg23060747	-0.34261	2.78664	-7.31178	5.01E-10	1.36E-06	12.24111	PD
cg00055603	-0.3726	3.680001	-7.30901	5.07E-10	1.37E-06	12.23496	PD
cg14922328	-0.56243	4.194575	-7.30705	5.11E-10	1.38E-06	12.19101	PD
cg18934139	0.789871	-5.77046	7.305864	5.13E-10	1.38E-06	12.20632	PD
cg12715136	-0.32431	3.567456	-7.30561	5.14E-10	1.38E-06	12.24307	PD
cg10528826	-0.51098	3.965216	-7.3053	5.15E-10	1.38E-06	12.2102	PD
cg11106864	-0.99384	3.984744	-7.30101	5.24E-10	1.40E-06	12.11089	PD
cg13326354	0.650386	3.860799	7.279409	5.72E-10	1.52E-06	12.08177	PD
cg04615668	0.493888	3.339901	7.273196	5.87E-10	1.55E-06	12.13005	PD
cg26843567	-0.39743	3.434633	-7.27284	5.87E-10	1.55E-06	12.12374	PD
cg05476733	-0.63437	4.247633	-7.26988	5.95E-10	1.57E-06	12.11874	PD
cg06073459	-0.39033	3.498718	-7.26295	6.12E-10	1.61E-06	12.0205	PD
cg23436303	-0.42958	4.049663	-7.26208	6.14E-10	1.61E-06	12.0258	PD
cg07493237	-0.75091	3.137326	-7.25947	6.20E-10	1.62E-06	12.06423	PD
cg15116775	-1.08917	3.656912	-7.25539	6.31E-10	1.64E-06	11.88501	PD
cg15512314	-0.4939	3.477078	-7.24586	6.56E-10	1.70E-06	12.02582	PD
cg22660197	-0.50235	3.76524	-7.24476	6.58E-10	1.70E-06	11.99967	PD
cg11927033	0.675783	-5.14061	7.238737	6.75E-10	1.74E-06	11.97302	PD
cg09044139	-0.55542	3.830461	-7.23699	6.80E-10	1.75E-06	12.03005	PD
cg02439483	-0.39372	3.931568	-7.23593	6.83E-10	1.75E-06	11.96766	PD
cg00244894	0.489956	-5.39502	7.234961	6.85E-10	1.75E-06	11.85076	PD
cg04950423	-0.24605	4.501116	-7.22958	7.00E-10	1.78E-06	11.92329	PD
cg13384867	-0.31691	6.261061	-7.22193	7.23E-10	1.84E-06	11.8238	PD
cg14039968	-0.31755	3.949086	-7.21731	7.36E-10	1.86E-06	11.92294	PD

cg03114748	0.622153	3.616503	7.207742	7.66E-10	1.93E-06	11.87768	PD
cg12223279	-0.35193	3.226103	-7.20635	7.70E-10	1.94E-06	11.87039	PD
cg01276031	-0.42371	4.20952	-7.19052	8.21E-10	2.06E-06	11.80025	PD
cg19663354	0.631051	5.814214	7.177871	8.64E-10	2.16E-06	11.72172	PD
cg18013847	0.580679	4.82601	7.177851	8.65E-10	2.16E-06	11.81208	PD
cg18717044	-0.48763	4.431515	-7.17647	8.69E-10	2.16E-06	11.73505	PD
cg23007441	-0.26115	4.105432	-7.17625	8.70E-10	2.16E-06	11.73868	PD
cg02879584	0.457618	4.073638	7.168512	8.98E-10	2.22E-06	11.76258	PD
cg09818739	-0.27473	3.115857	-7.16526	9.10E-10	2.24E-06	11.62237	PD
cg15849439	-0.40636	4.28995	-7.16512	9.10E-10	2.24E-06	11.7216	PD
cg00578690	0.548385	4.05472	7.157722	9.38E-10	2.30E-06	11.59394	PD
cg16053651	-0.24323	3.733722	-7.15211	9.60E-10	2.35E-06	11.65056	PD
cg04606663	-0.25509	4.038479	-7.14853	9.74E-10	2.37E-06	11.66457	PD
cg21755610	0.38216	3.737519	7.146231	9.83E-10	2.39E-06	11.64	PD
cg20107632	0.920714	3.339182	7.139754	1.01E-09	2.45E-06	11.66982	PD
cg06982827	0.518322	4.485624	7.137378	1.02E-09	2.46E-06	11.66448	PD
cg01394772	0.772595	2.299275	7.135962	1.03E-09	2.47E-06	11.42317	PD
cg22968863	-0.40617	3.74899	-7.13543	1.03E-09	2.47E-06	11.57357	PD
cg03875091	-0.62261	3.798883	-7.13298	1.04E-09	2.49E-06	11.60016	PD
cg05708441	-1.74405	3.838859	-7.13082	1.05E-09	2.50E-06	11.52753	PD
cg14280627	-0.74465	-5.05477	-7.12568	1.07E-09	2.55E-06	11.46616	PD
cg25207147	-0.28413	3.842643	-7.12078	1.09E-09	2.58E-06	11.49386	PD
cg11843674	0.298893	4.366278	7.120588	1.09E-09	2.58E-06	11.59248	PD
cg15418104	0.276168	1.180981	7.118266	1.10E-09	2.60E-06	11.50744	PD
cg10797866	0.605919	4.519899	7.117785	1.10E-09	2.60E-06	11.53191	PD
cg22336141	0.733691	3.91613	7.110521	1.14E-09	2.67E-06	11.54402	PD
cg06620926	-0.69296	2.988549	-7.1074	1.15E-09	2.70E-06	11.45669	PD
cg16655291	-0.69314	4.418163	-7.1019	1.18E-09	2.75E-06	11.44302	PD
cg21556271	-1.08101	2.807101	-7.09826	1.19E-09	2.78E-06	11.4962	PD
cg19360212	-0.87986	3.691297	-7.08884	1.24E-09	2.88E-06	11.34271	PD
cg03333052	-0.27334	3.79902	-7.08488	1.26E-09	2.92E-06	11.4287	PD

cg11753155	1.450154	-5.98654	7.08365	1.27E-09	2.92E-06	11.34251	PD
cg13422830	-1.2133	4.610431	-7.08343	1.27E-09	2.92E-06	11.31725	PD
cg09578729	0.783293	3.5612	7.083111	1.27E-09	2.92E-06	11.40663	PD
cg08061275	0.337253	3.908476	7.081944	1.28E-09	2.93E-06	11.3649	PD
cg14198352	-2.03491	4.840389	-7.07917	1.29E-09	2.95E-06	11.24673	PD
cg24240654	-0.36427	3.293503	-7.07806	1.30E-09	2.96E-06	11.38638	PD
cg04883592	0.620083	3.195682	7.076286	1.31E-09	2.97E-06	11.28598	PD
cg15016740	0.890613	3.617573	7.074355	1.32E-09	2.99E-06	11.35591	PD
cg12770003	-1.11262	4.782022	-7.07367	1.32E-09	2.99E-06	11.3067	PD
cg05353868	-0.21008	3.395728	-7.07096	1.33E-09	3.01E-06	11.35411	PD
cg03863692	0.277575	4.699108	7.064005	1.37E-09	3.09E-06	11.38279	PD
cg20715026	-0.47496	3.99431	-7.06153	1.39E-09	3.11E-06	11.26448	PD
cg12426776	0.64197	3.585237	7.059946	1.40E-09	3.12E-06	11.31147	PD
cg03513647	-0.48554	4.646815	-7.05211	1.44E-09	3.22E-06	11.23298	PD
cg09496544	-0.29107	4.027659	-7.05042	1.45E-09	3.23E-06	11.30643	PD
cg19157240	-0.39714	4.275182	-7.04465	1.49E-09	3.30E-06	11.23176	PD
cg22708327	-0.51867	2.85482	-7.04236	1.50E-09	3.32E-06	11.30789	PD
cg19874475	-1.52543	-7.32801	-7.04194	1.50E-09	3.32E-06	11.26678	PD
cg10546252	-1.90278	-5.56749	-7.04108	1.51E-09	3.32E-06	11.19215	PD
cg13474262	1.007777	3.249427	7.038176	1.52E-09	3.35E-06	11.07894	PD
cg23668222	-0.53994	4.257145	-7.03025	1.57E-09	3.45E-06	11.25328	PD
cg08172037	-0.39585	4.492235	-7.02996	1.58E-09	3.45E-06	11.22387	PD
cg25148674	-0.57178	4.183089	-7.02767	1.59E-09	3.47E-06	11.17173	PD
cg06899226	-0.79731	3.908804	-7.02384	1.62E-09	3.51E-06	11.03948	PD
cg21485543	-0.50323	3.732033	-7.01925	1.65E-09	3.56E-06	11.10904	PD
cg11063328	0.759837	-5.0353	7.019096	1.65E-09	3.56E-06	11.07502	PD
cg12989560	-0.47323	-4.41161	-7.01846	1.65E-09	3.56E-06	11.11764	PD
cg11304899	1.189546	3.657131	7.017961	1.66E-09	3.56E-06	11.19405	PD
cg20270863	0.864362	-4.60676	7.017553	1.66E-09	3.56E-06	11.19782	PD
cg23075597	1.21604	3.225759	7.012237	1.69E-09	3.63E-06	11.12704	PD
cg21397098	-0.3723	3.456036	-7.00085	1.77E-09	3.79E-06	11.15086	PD

cg16109817	0.527362	-5.3259	7.00039	1.78E-09	3.79E-06	11.07168	PD
cg00244610	-0.43581	4.198885	-6.99746	1.80E-09	3.82E-06	11.11865	PD
cg16022038	0.711841	3.388679	6.996187	1.81E-09	3.83E-06	11.09784	PD
cg25279778	-0.27092	3.957605	-6.99468	1.82E-09	3.85E-06	11.0743	PD
cg02504993	-0.95083	2.715991	-6.99349	1.83E-09	3.86E-06	11.08583	PD
cg17776040	-0.62164	3.575645	-6.98284	1.91E-09	4.02E-06	10.94089	PD
cg17146617	-0.40489	3.782736	-6.98053	1.93E-09	4.04E-06	11.06357	PD
cg24492374	-2.3777	-5.03071	-6.97726	1.95E-09	4.09E-06	10.97823	PD
cg05673539	0.603465	3.742688	6.975323	1.97E-09	4.11E-06	10.98497	PD
cg07063294	1.08018	6.508094	6.973788	1.98E-09	4.12E-06	11.0537	PD
cg20910935	0.643921	3.913061	6.973604	1.98E-09	4.12E-06	11.04032	PD
cg01052274	-0.40817	3.511992	-6.97223	1.99E-09	4.13E-06	11.01321	PD
cg01442214	1.498204	4.066344	6.966293	2.04E-09	4.22E-06	10.92945	PD
cg09232487	-0.35799	2.73873	-6.95755	2.11E-09	4.36E-06	10.96408	PD
cg08267278	-0.97957	3.946038	-6.94184	2.25E-09	4.64E-06	10.82913	PD
cg22759624	-0.4812	4.173365	-6.94131	2.26E-09	4.64E-06	10.93644	PD
cg15032304	-0.86674	3.665181	-6.93836	2.29E-09	4.68E-06	10.81938	PD
cg03289221	0.724129	4.026818	6.935978	2.31E-09	4.72E-06	10.77412	PD
cg18419070	0.564139	4.405092	6.931765	2.35E-09	4.79E-06	10.84012	PD
cg10848257	-0.30702	4.118104	-6.92966	2.37E-09	4.81E-06	10.86903	PD
cg22975300	0.830275	4.097863	6.927087	2.39E-09	4.85E-06	10.81181	PD
cg22845224	-0.42212	4.369184	-6.9235	2.43E-09	4.91E-06	10.79252	PD
cg16022555	0.468706	-4.27197	6.920061	2.46E-09	4.97E-06	10.79837	PD
cg12938223	-0.32576	3.58606	-6.9146	2.52E-09	5.07E-06	10.75899	PD
cg07600373	-0.36394	4.33673	-6.90991	2.57E-09	5.15E-06	10.80212	PD
cg01173558	-0.47532	3.71568	-6.90682	2.60E-09	5.20E-06	10.73976	PD
cg00436496	-0.90861	-4.4497	-6.90678	2.60E-09	5.20E-06	10.77662	PD
cg11505805	-0.58798	2.536743	-6.9058	2.61E-09	5.20E-06	10.79294	PD
cg17508167	-0.38576	3.471521	-6.90525	2.61E-09	5.20E-06	10.77012	PD
cg25470611	-0.31367	1.727482	-6.90125	2.66E-09	5.28E-06	10.68836	PD
cg21604803	0.747762	-5.08898	6.897071	2.70E-09	5.34E-06	10.72197	PD

cg22830202	-0.48059	2.257547	-6.89668	2.71E-09	5.34E-06	10.66629	PD
cg26041493	-0.5842	5.672125	-6.89641	2.71E-09	5.34E-06	10.65609	PD
cg14841443	-0.29057	3.7674	-6.8938	2.74E-09	5.39E-06	10.48365	PD
cg03505857	-0.44737	4.51231	-6.88922	2.79E-09	5.47E-06	10.76	PD
cg26217085	-0.39112	3.204316	-6.88882	2.79E-09	5.47E-06	10.68791	PD
cg21927055	-0.36333	3.916869	-6.88462	2.84E-09	5.55E-06	10.67973	PD
cg16729283	0.611231	4.119168	6.882727	2.86E-09	5.57E-06	10.56713	PD
cg05331692	0.713575	3.204977	6.882552	2.87E-09	5.57E-06	10.67104	PD
cg19855227	-0.81051	-5.25696	-6.87906	2.91E-09	5.63E-06	10.54225	PD
cg13508672	-0.37018	4.027894	-6.87903	2.91E-09	5.63E-06	10.65972	PD
cg25655482	0.788914	3.820736	6.87409	2.97E-09	5.73E-06	10.64192	PD
cg25911398	-0.74089	3.566198	-6.86043	3.13E-09	6.04E-06	10.46611	PD
cg18915099	-0.53598	4.282416	-6.85976	3.14E-09	6.04E-06	10.59038	PD
cg08931987	0.431947	3.972866	6.837659	3.44E-09	6.59E-06	10.53956	PD
cg00183480	-0.56146	3.78684	-6.83712	3.44E-09	6.59E-06	10.51357	PD
cg18399629	-0.26425	4.017566	-6.82266	3.65E-09	6.97E-06	10.48832	PD
cg23628949	0.494411	4.249095	6.821666	3.67E-09	6.99E-06	10.49366	PD
cg08119527	-1.01031	3.639913	-6.8194	3.70E-09	7.03E-06	10.4411	PD
cg20823529	-0.62272	3.193303	-6.81586	3.75E-09	7.12E-06	10.2479	PD
cg01233215	-0.52003	-5.60599	-6.8121	3.81E-09	7.21E-06	10.45527	PD
cg21425790	0.626412	3.340957	6.80736	3.89E-09	7.32E-06	10.41446	PD
cg25928062	-0.364	4.309216	-6.80673	3.90E-09	7.32E-06	10.43628	PD
cg21003441	-0.50627	4.613905	-6.80668	3.90E-09	7.32E-06	10.43217	PD
cg24575376	0.426711	4.171297	6.802279	3.97E-09	7.44E-06	10.39877	PD
cg15702478	-0.44978	4.208256	-6.80058	3.99E-09	7.47E-06	10.37404	PD
cg25722644	0.497054	4.241257	6.79557	4.08E-09	7.60E-06	10.37605	PD
cg05023707	0.403412	3.97813	6.795371	4.08E-09	7.60E-06	10.38718	PD
cg09844794	-0.40544	4.573448	-6.79065	4.16E-09	7.73E-06	10.30452	PD
cg02118823	-0.64051	-5.14063	-6.78905	4.18E-09	7.76E-06	10.27368	PD
cg16878920	-0.32556	4.17154	-6.78719	4.22E-09	7.80E-06	10.34248	PD
cg03141489	-0.47386	3.432343	-6.78609	4.23E-09	7.82E-06	10.32435	PD

cg00850175	0.425395	3.137281	6.783431	4.28E-09	7.89E-06	10.27813	PD
cg25956966	-0.71668	5.654583	-6.78174	4.31E-09	7.92E-06	10.2392	PD
cg02820548	-0.5859	3.912899	-6.77919	4.35E-09	7.99E-06	10.25341	PD
cg02109711	-0.73084	-3.77403	-6.77364	4.45E-09	8.14E-06	10.31285	PD
cg07116861	-0.33844	3.382904	-6.77344	4.46E-09	8.14E-06	10.29836	PD
cg18532869	0.771417	-5.23294	6.769233	4.53E-09	8.26E-06	10.25863	PD
cg07157757	0.316382	4.502848	6.768802	4.54E-09	8.26E-06	10.28397	PD
cg18146506	0.966081	-4.04561	6.761733	4.67E-09	8.48E-06	10.11172	PD
cg08214342	-1.07297	4.014279	-6.75774	4.75E-09	8.60E-06	10.01461	PD
cg05674046	-0.38977	3.395046	-6.75561	4.79E-09	8.66E-06	10.20974	PD
cg26500754	-0.53485	3.685081	-6.75336	4.83E-09	8.72E-06	10.21466	PD
cg23494018	0.550753	4.250611	6.752136	4.86E-09	8.74E-06	10.06763	PD
cg00615835	-0.47866	3.91999	-6.74854	4.93E-09	8.85E-06	10.17798	PD
cg03886254	-0.3373	4.241195	-6.74764	4.95E-09	8.86E-06	10.20916	PD
cg13130965	0.39916	-6.07656	6.746233	4.97E-09	8.90E-06	10.14958	PD
cg11327317	-0.33326	4.161466	-6.74426	5.01E-09	8.95E-06	10.16646	PD
cg18782604	-0.47409	-5.17505	-6.74109	5.08E-09	9.04E-06	10.14336	PD
cg14078231	0.721501	-5.10617	6.734324	5.22E-09	9.27E-06	10.14808	PD
cg26673784	-0.42988	3.479395	-6.73392	5.23E-09	9.27E-06	10.15703	PD
cg10733507	-0.60422	3.127562	-6.73327	5.24E-09	9.28E-06	10.1169	PD
cg01346252	-0.34721	4.708177	-6.7264	5.39E-09	9.52E-06	10.04233	PD
cg25128170	-0.31422	4.278429	-6.72523	5.42E-09	9.54E-06	10.11008	PD
cg20189053	-0.35728	3.936564	-6.72406	5.44E-09	9.57E-06	10.07689	PD
cg02284293	-0.49353	3.741922	-6.72075	5.51E-09	9.68E-06	10.05646	PD
cg22593669	-2.25781	-5.77905	-6.71924	5.55E-09	9.71E-06	10.10652	PD
cg11868634	-0.45167	3.387507	-6.71621	5.62E-09	9.81E-06	10.02449	PD
cg00052487	0.744552	3.891747	6.71467	5.65E-09	9.85E-06	10.09492	PD
cg08012663	-0.36067	4.431302	-6.70913	5.78E-09	1.01E-05	9.93862	PD
cg21886364	-0.44102	3.319825	-6.70405	5.90E-09	1.02E-05	9.968952	PD
cg26204322	-1.06607	-5.80856	-6.69791	6.05E-09	1.05E-05	10.05445	PD
cg15482420	-0.35796	3.862303	-6.69733	6.06E-09	1.05E-05	9.996517	PD

cg05304538	-0.3401	3.955172	-6.69361	6.15E-09	1.06E-05	9.979211	PD
cg25919177	-0.33736	3.789379	-6.69188	6.20E-09	1.07E-05	9.909635	PD
cg00978344	-0.39386	4.452864	-6.68475	6.38E-09	1.10E-05	9.9346	PD
cg12904922	-0.58834	4.177284	-6.68325	6.41E-09	1.10E-05	9.87562	PD
cg01504714	0.838896	3.723096	6.676258	6.60E-09	1.13E-05	9.817403	PD
cg18202492	0.36476	3.82866	6.674232	6.65E-09	1.14E-05	9.8982	PD
cg19878076	-0.54173	3.189561	-6.67387	6.66E-09	1.14E-05	9.84133	PD
cg13787154	-0.60849	4.030716	-6.67327	6.68E-09	1.14E-05	9.887474	PD
cg16857912	-0.48538	3.416442	-6.67257	6.70E-09	1.14E-05	9.888963	PD
cg23148596	-0.26844	3.80294	-6.67028	6.76E-09	1.15E-05	9.882752	PD
cg17176698	-1.87774	3.851073	-6.66852	6.81E-09	1.15E-05	9.700594	PD
cg07722774	0.352226	5.359671	6.665826	6.88E-09	1.16E-05	9.878697	PD
cg25619350	-0.64855	3.994062	-6.66542	6.89E-09	1.16E-05	9.874786	PD
cg09145778	-1.91601	6.501452	-6.66205	6.99E-09	1.17E-05	9.798998	PD
cg04989813	-0.49339	4.47622	-6.65561	7.17E-09	1.20E-05	9.876565	PD
cg11483424	0.348374	4.39653	6.652691	7.26E-09	1.22E-05	9.80724	PD
cg12727358	-0.47836	3.613854	-6.64643	7.44E-09	1.24E-05	9.625182	PD
cg13144628	-0.27626	4.309182	-6.64459	7.50E-09	1.25E-05	9.806439	PD
cg12562660	0.423354	4.328914	6.636801	7.74E-09	1.29E-05	9.77251	PD
cg25704068	-0.30519	3.499522	-6.63227	7.88E-09	1.31E-05	9.683516	PD
cg15390391	-0.67962	-5.23148	-6.63178	7.89E-09	1.31E-05	9.807262	PD
cg03108651	0.632248	3.491683	6.630392	7.94E-09	1.31E-05	9.688957	PD
cg01565659	0.605863	2.873859	6.629649	7.96E-09	1.32E-05	9.799089	PD
cg06296752	-0.37329	4.424975	-6.62726	8.04E-09	1.33E-05	9.743679	PD
cg17755535	-0.39353	4.150252	-6.62589	8.08E-09	1.33E-05	9.7242	PD
cg24996821	-0.34733	4.191977	-6.6239	8.15E-09	1.34E-05	9.766185	PD
cg19492498	0.472568	3.356844	6.620643	8.26E-09	1.35E-05	9.714245	PD
cg08061367	-0.69432	4.587028	-6.61959	8.29E-09	1.36E-05	9.709458	PD
cg27531470	-0.67404	-4.31712	-6.61245	8.53E-09	1.39E-05	9.684099	PD
cg11976616	-0.47463	3.693682	-6.6017	8.91E-09	1.45E-05	9.644471	PD
cg03619081	0.431479	3.531028	6.597571	9.06E-09	1.47E-05	9.590018	PD

cg27659734	0.681548	-4.24353	6.591878	9.27E-09	1.51E-05	9.594117	PD
cg23698649	-0.52437	3.059337	-6.59088	9.31E-09	1.51E-05	9.580912	PD
cg05505627	0.826997	-6.59243	6.587175	9.45E-09	1.53E-05	9.60973	PD
cg15199040	-0.3169	3.689761	-6.5837	9.58E-09	1.55E-05	9.625003	PD
cg00422068	0.355779	4.154111	6.583107	9.60E-09	1.55E-05	9.591781	PD
cg01033336	0.569573	3.924451	6.582742	9.62E-09	1.55E-05	9.58047	PD
cg06764387	-0.37474	3.023152	-6.57668	9.86E-09	1.58E-05	9.557216	PD
cg24364568	0.76725	4.32825	6.576008	9.88E-09	1.58E-05	9.585212	PD
cg07950798	-0.48738	5.257141	-6.57364	9.98E-09	1.60E-05	9.562365	PD
cg08916385	1.067092	3.644904	6.569871	1.01E-08	1.62E-05	9.539068	PD
cg00037940	0.325279	4.075008	6.567564	1.02E-08	1.63E-05	9.538958	PD
cg13868773	-0.30154	4.228161	-6.56706	1.02E-08	1.63E-05	9.557262	PD
cg12178185	-0.39031	3.903995	-6.567	1.02E-08	1.63E-05	9.545174	PD
cg02552490	0.75006	3.737172	6.565711	1.03E-08	1.63E-05	9.397875	PD
cg09238196	-0.41021	4.521959	-6.56271	1.04E-08	1.65E-05	9.466261	PD
cg07920089	-0.27908	4.030455	-6.56024	1.05E-08	1.66E-05	9.516464	PD
cg08848269	0.257923	4.329027	6.557811	1.06E-08	1.67E-05	9.536141	PD
cg16987213	-0.26905	3.15331	-6.55778	1.06E-08	1.67E-05	9.517003	PD
cg25809434	-0.7004	5.271452	-6.5535	1.08E-08	1.70E-05	9.44482	PD
cg24510437	-0.45843	3.544725	-6.54719	1.11E-08	1.74E-05	9.503165	PD
cg14495124	-0.50362	4.288751	-6.54603	1.11E-08	1.74E-05	9.4355	PD
cg11643867	-0.32665	3.826176	-6.54562	1.12E-08	1.74E-05	9.418165	PD
cg23057687	-0.32416	3.58874	-6.54392	1.12E-08	1.75E-05	9.451626	PD
cg19374462	-1.1044	5.450683	-6.54231	1.13E-08	1.76E-05	9.389229	PD
cg04470905	0.836671	-5.64771	6.534588	1.17E-08	1.81E-05	9.401776	PD
cg22673500	0.430134	3.944263	6.533263	1.17E-08	1.82E-05	9.411004	PD
cg07203362	-0.55762	2.734459	-6.52857	1.20E-08	1.85E-05	9.379945	PD
cg06839896	0.545159	3.703334	6.521234	1.23E-08	1.90E-05	9.374226	PD
cg11471805	-0.19784	4.126644	-6.52114	1.23E-08	1.90E-05	9.356409	PD
cg02617107	-0.48364	4.008706	-6.521	1.23E-08	1.90E-05	9.329657	PD
cg09134179	-0.37585	4.029264	-6.51609	1.26E-08	1.93E-05	9.366754	PD

cg18281723	-0.36787	4.293023	-6.50917	1.29E-08	1.98E-05	9.328426	PD
cg15575098	-0.51915	4.347036	-6.50905	1.29E-08	1.98E-05	9.344583	PD
cg26173420	0.450501	4.494707	6.508626	1.30E-08	1.98E-05	9.335244	PD
cg02590715	0.767879	3.629452	6.506441	1.31E-08	1.99E-05	9.242051	PD
cg20146868	-0.36245	-4.3309	-6.50303	1.33E-08	2.02E-05	9.315738	PD
cg21450627	0.383832	3.597531	6.501153	1.34E-08	2.03E-05	9.302995	PD
cg24330042	-1.94978	-5.24786	-6.50042	1.34E-08	2.03E-05	9.229798	PD
cg10180142	-0.32944	4.040394	-6.49782	1.35E-08	2.05E-05	9.291252	PD
cg09658126	-0.97276	-5.02854	-6.49713	1.36E-08	2.05E-05	9.259377	PD
cg13133503	-0.34913	3.523089	-6.49565	1.37E-08	2.06E-05	9.257222	PD
cg27231166	-0.65226	3.905444	-6.49046	1.39E-08	2.10E-05	9.191864	PD
cg15794909	-0.66209	4.328916	-6.49033	1.39E-08	2.10E-05	9.092757	PD
cg14937732	-0.46921	4.53441	-6.48701	1.41E-08	2.12E-05	9.212072	PD
cg27291337	-0.34222	3.502593	-6.48079	1.45E-08	2.17E-05	9.227088	PD
cg04481295	0.571452	3.676098	6.470538	1.51E-08	2.25E-05	9.163303	PD
cg00699919	0.567189	4.228495	6.470454	1.51E-08	2.25E-05	9.169516	PD
cg05276512	-0.3562	3.778533	-6.46749	1.53E-08	2.28E-05	9.166322	PD
cg16372051	-0.40121	3.21034	-6.46718	1.53E-08	2.28E-05	9.187368	PD
cg22888387	-0.41898	4.140203	-6.46234	1.56E-08	2.32E-05	9.144295	PD
cg14737164	-0.32492	3.22431	-6.4603	1.57E-08	2.33E-05	9.107736	PD
cg08223534	-0.93925	-5.92069	-6.4599	1.58E-08	2.33E-05	9.095216	PD
cg03447327	-0.3221	4.44879	-6.45894	1.58E-08	2.34E-05	9.115719	PD
cg00954566	0.826718	-5.6931	6.457828	1.59E-08	2.34E-05	9.079987	PD
cg01203147	-0.30349	3.907069	-6.45395	1.61E-08	2.37E-05	9.108754	PD
cg15031658	-0.6943	3.504488	-6.4537	1.62E-08	2.37E-05	9.123265	PD
cg15999674	-0.51041	3.000752	-6.45064	1.64E-08	2.40E-05	9.08984	PD
cg03453277	-0.30473	2.535966	-6.44956	1.64E-08	2.40E-05	9.113673	PD
cg20986381	0.386143	4.187402	6.448218	1.65E-08	2.41E-05	9.088875	PD
cg00982294	0.327593	4.13017	6.44447	1.68E-08	2.44E-05	9.09171	PD
cg04947676	0.387078	3.767509	6.43688	1.73E-08	2.52E-05	9.009871	PD
cg13281547	0.737979	-7.04422	6.435118	1.74E-08	2.53E-05	9.038138	PD

cg18130152	-0.3584	4.221676	-6.43493	1.74E-08	2.53E-05	9.028259	PD
cg06367823	0.625405	3.091239	6.430149	1.78E-08	2.57E-05	8.974427	PD
cg23316161	0.862567	-6.61963	6.430007	1.78E-08	2.57E-05	9.048795	PD
cg27290925	0.277357	-4.60047	6.427205	1.80E-08	2.59E-05	8.988495	PD
cg24200401	-0.33146	3.014243	-6.4266	1.80E-08	2.60E-05	9.044523	PD
cg11138521	-0.33524	4.231933	-6.42152	1.84E-08	2.64E-05	9.021647	PD
cg17813879	1.057825	2.773905	6.420775	1.84E-08	2.65E-05	8.90381	PD
cg01334366	-0.29688	2.674359	-6.41941	1.85E-08	2.66E-05	8.921996	PD
cg08968978	-0.36673	3.306302	-6.41823	1.86E-08	2.67E-05	8.969756	PD
cg08941871	0.239379	1.17075	6.409953	1.93E-08	2.75E-05	8.97918	PD
cg07869723	-0.44758	4.637165	-6.40987	1.93E-08	2.75E-05	8.988455	PD
cg14823900	0.32418	4.20783	6.40835	1.94E-08	2.76E-05	8.988708	PD
cg15397657	0.479648	3.24821	6.401807	1.99E-08	2.83E-05	8.88864	PD
cg18756657	0.47089	3.390923	6.396819	2.03E-08	2.88E-05	8.914894	PD
cg01006898	-0.38045	4.327626	-6.39449	2.05E-08	2.90E-05	8.915859	PD
cg01242677	0.687305	4.017449	6.391035	2.08E-08	2.94E-05	8.890683	PD
cg01861555	0.492518	4.339824	6.387295	2.11E-08	2.98E-05	8.91128	PD
cg03093391	0.367548	3.850564	6.386181	2.12E-08	2.99E-05	8.899075	PD
cg06199438	-0.29404	4.370144	-6.38473	2.13E-08	3.00E-05	8.846735	PD
cg10269948	0.340467	4.641072	6.378878	2.18E-08	3.06E-05	8.86083	PD
cg15295012	-0.83745	4.508231	-6.37302	2.23E-08	3.13E-05	8.776992	PD
cg20281046	-0.40179	5.278416	-6.36902	2.27E-08	3.18E-05	8.799328	PD
cg06500090	-0.42425	3.46658	-6.3686	2.27E-08	3.18E-05	8.803471	PD
cg12855555	0.548147	4.131083	6.367002	2.29E-08	3.19E-05	8.835186	PD
cg27099088	-0.29512	3.857723	-6.36333	2.32E-08	3.23E-05	8.825027	PD
cg02018388	-0.47619	4.32809	-6.36114	2.34E-08	3.26E-05	8.787633	PD
cg24120210	-1.98174	6.636159	-6.36044	2.35E-08	3.26E-05	8.795649	PD
cg03838062	0.492015	4.076965	6.359341	2.36E-08	3.27E-05	8.748259	PD
cg03012785	-0.59275	3.682825	-6.35652	2.38E-08	3.30E-05	8.795797	PD
cg08659016	1.026524	4.553805	6.354874	2.40E-08	3.32E-05	8.719508	PD
cg04860828	-0.29925	4.242651	-6.35458	2.40E-08	3.32E-05	8.710132	PD

cg27433449	0.256425	3.425927	6.354118	2.41E-08	3.32E-05	8.73807	PD
cg05828606	-0.5695	3.24482	-6.35217	2.43E-08	3.34E-05	8.691285	PD
cg02245362	-0.38702	4.352975	-6.34914	2.46E-08	3.37E-05	8.765093	PD
cg16270643	-0.71214	3.944304	-6.34651	2.48E-08	3.40E-05	8.690206	PD
cg21826978	0.40742	3.844325	6.34277	2.52E-08	3.45E-05	8.726475	PD
cg02704963	-0.4358	4.421113	-6.34203	2.53E-08	3.45E-05	8.65428	PD
cg05648672	0.305076	-5.40286	6.340142	2.55E-08	3.47E-05	8.730195	PD
cg02831106	-0.36303	3.736851	-6.33758	2.57E-08	3.50E-05	8.689666	PD
cg09828339	-0.28167	3.449095	-6.33634	2.59E-08	3.52E-05	8.702989	PD
cg23117085	-1.32855	3.736223	-6.33541	2.59E-08	3.52E-05	8.548089	PD
cg15904834	-0.22147	3.944219	-6.32991	2.65E-08	3.60E-05	8.634028	PD
cg20940921	-0.54866	3.104502	-6.32614	2.69E-08	3.64E-05	8.670065	PD
cg02145160	-0.46801	3.405445	-6.32482	2.71E-08	3.65E-05	8.581644	PD
cg16627198	0.376184	4.168763	6.324803	2.71E-08	3.65E-05	8.659613	PD
cg24311695	-0.93115	4.57216	-6.32229	2.73E-08	3.68E-05	8.506942	PD
cg22065513	-0.38501	4.459621	-6.32038	2.76E-08	3.71E-05	8.623676	PD
cg01546248	-1.21342	-6.43558	-6.31883	2.77E-08	3.72E-05	8.605914	PD
cg18391514	-0.37772	2.783918	-6.31558	2.81E-08	3.76E-05	8.593041	PD
cg05848866	-0.40161	4.677874	-6.31555	2.81E-08	3.76E-05	8.643303	PD
cg09255850	0.998942	-3.78612	6.31219	2.85E-08	3.80E-05	8.522356	PD
cg24074149	0.585026	3.034759	6.310843	2.86E-08	3.82E-05	8.555977	PD
cg04072177	-0.51051	3.423519	-6.30863	2.89E-08	3.84E-05	8.501625	PD
cg12086421	-2.13039	4.405915	-6.30844	2.89E-08	3.84E-05	8.557321	PD
cg08942682	-0.25412	2.889151	-6.307	2.91E-08	3.86E-05	8.583746	PD
cg24675983	0.35112	3.396135	6.306897	2.91E-08	3.86E-05	8.532936	PD
cg23622129	-0.84778	3.058894	-6.30334	2.95E-08	3.90E-05	8.480959	PD
cg26153182	0.474477	3.965797	6.303	2.95E-08	3.90E-05	8.524282	PD
cg22179177	0.77613	-5.68621	6.298679	3.01E-08	3.97E-05	8.513652	PD
cg20462978	-1.231	4.634089	-6.29783	3.02E-08	3.97E-05	8.284238	PD
cg15935227	-3.07099	5.456779	-6.29756	3.02E-08	3.97E-05	8.570933	PD
cg16007185	0.896232	-4.65069	6.29648	3.03E-08	3.98E-05	8.49202	PD

cg06241915	-0.39954	3.748037	-6.2963	3.03E-08	3.98E-05	8.558258	PD
cg23962250	-0.30777	-5.35408	-6.28697	3.15E-08	4.11E-05	8.52856	PD
cg23243080	-0.88534	-5.06121	-6.28696	3.15E-08	4.11E-05	8.511442	PD
cg19377944	-0.25668	3.090313	-6.28649	3.15E-08	4.11E-05	8.501556	PD
cg26077897	-0.34493	4.32282	-6.28648	3.16E-08	4.11E-05	8.508999	PD
cg02340312	-0.47459	3.502992	-6.28433	3.18E-08	4.14E-05	8.322252	PD
cg10608717	-0.56127	4.484757	-6.28285	3.20E-08	4.16E-05	8.485332	PD
cg21735491	0.18082	-3.68057	6.280779	3.23E-08	4.19E-05	8.45741	PD
cg04785931	0.736463	3.548124	6.280228	3.23E-08	4.19E-05	8.458018	PD
cg04704414	-0.37182	2.935304	-6.27922	3.25E-08	4.19E-05	8.495646	PD
cg05342816	0.752085	3.266464	6.279185	3.25E-08	4.19E-05	8.461805	PD
cg02955490	0.515293	2.744094	6.278477	3.26E-08	4.20E-05	8.451369	PD
cg17411020	-0.3852	4.15254	-6.27455	3.31E-08	4.26E-05	8.490397	PD
cg11039604	0.595877	4.524634	6.272994	3.33E-08	4.28E-05	8.452173	PD
cg00077566	0.600064	4.313117	6.262428	3.47E-08	4.46E-05	8.3841	PD
cg05779148	-0.47362	-4.5628	-6.26212	3.48E-08	4.46E-05	8.437918	PD
cg23330137	-0.45535	4.457821	-6.2612	3.49E-08	4.47E-05	8.362952	PD
cg23053688	0.761665	-5.33344	6.260022	3.51E-08	4.48E-05	8.413822	PD
cg11344530	-0.29935	3.255114	-6.25815	3.53E-08	4.51E-05	8.390915	PD
cg11504445	0.342171	4.458567	6.255077	3.58E-08	4.55E-05	8.402745	PD
cg25267284	-0.56564	6.025995	-6.25492	3.58E-08	4.55E-05	8.405055	PD
cg00347599	-0.35524	4.157217	-6.25002	3.65E-08	4.63E-05	8.391774	PD
cg25047307	-0.44237	3.728676	-6.24841	3.67E-08	4.66E-05	8.352865	PD
cg01222306	-0.33983	3.063814	-6.24442	3.73E-08	4.72E-05	8.318016	PD
cg24668570	-0.66627	-4.46225	-6.24369	3.74E-08	4.73E-05	8.2776	PD
cg23970422	-0.37969	4.125697	-6.24185	3.77E-08	4.76E-05	8.354441	PD
cg06032500	0.431761	3.639799	6.240883	3.78E-08	4.77E-05	8.356361	PD
cg14577958	-0.42494	4.33851	-6.24076	3.79E-08	4.77E-05	8.304403	PD
cg07959977	-0.29343	3.790584	-6.23053	3.94E-08	4.96E-05	8.339279	PD
cg02938130	0.564616	-5.12177	6.227967	3.98E-08	5.00E-05	8.328992	PD
cg08839808	-0.68505	2.821905	-6.22664	4.01E-08	5.02E-05	8.312643	PD

cg01860297	-0.30828	3.691211	-6.22593	4.02E-08	5.03E-05	8.283567	PD
cg00477015	-0.29863	3.955627	-6.22544	4.02E-08	5.03E-05	8.300141	PD
cg01824498	-0.36322	3.579771	-6.22374	4.05E-08	5.05E-05	8.275324	PD
cg15185795	-0.2987	3.672504	-6.22352	4.06E-08	5.05E-05	8.308835	PD
cg01649456	-0.37502	4.083927	-6.21988	4.12E-08	5.12E-05	8.272388	PD
cg00318166	-0.74034	3.357713	-6.21689	4.16E-08	5.17E-05	8.071463	PD
cg10578072	-0.44603	4.155455	-6.21142	4.26E-08	5.28E-05	8.242359	PD
cg14316237	-0.33236	3.114652	-6.20845	4.31E-08	5.33E-05	8.201261	PD
cg27315579	-0.25942	3.72814	-6.20709	4.33E-08	5.35E-05	8.225694	PD
cg00587941	1.723648	4.311358	6.206148	4.35E-08	5.37E-05	8.185551	PD
cg05386249	-0.30405	3.828438	-6.20352	4.39E-08	5.41E-05	8.214137	PD
cg12554413	-0.3334	3.438901	-6.20349	4.39E-08	5.41E-05	8.202746	PD
cg12320848	-0.31189	4.028755	-6.20317	4.40E-08	5.41E-05	8.16158	PD
cg15384061	-0.25347	3.17469	-6.20143	4.43E-08	5.44E-05	8.226946	PD
cg04464523	1.112249	3.371535	6.201012	4.44E-08	5.44E-05	8.192638	PD
cg06631775	-0.79598	3.862749	-6.19876	4.48E-08	5.47E-05	8.139547	PD
cg17111463	-0.21225	3.756054	-6.19863	4.48E-08	5.47E-05	8.186153	PD
cg07614295	-0.44327	4.387266	-6.1976	4.50E-08	5.49E-05	8.206791	PD
cg02607330	0.350961	4.283734	6.19689	4.51E-08	5.50E-05	8.177421	PD
cg23263057	-0.42303	3.972966	-6.19578	4.53E-08	5.51E-05	8.180575	PD
cg04725144	0.503715	2.462217	6.191988	4.60E-08	5.59E-05	8.1208	PD
cg07918844	-0.364	4.568811	-6.19019	4.63E-08	5.62E-05	8.192827	PD
cg06228648	-0.3605	4.138157	-6.18807	4.67E-08	5.66E-05	8.145193	PD
cg15147841	-0.29645	3.275238	-6.18761	4.68E-08	5.66E-05	8.151718	PD
cg11616262	-0.35236	3.170273	-6.1871	4.69E-08	5.67E-05	8.166887	PD
cg10748874	-0.32477	4.010768	-6.17964	4.83E-08	5.83E-05	8.101539	PD
cg03452630	-0.28905	4.112929	-6.17636	4.89E-08	5.90E-05	8.13338	PD
cg05213073	-0.32846	3.990484	-6.17587	4.90E-08	5.90E-05	8.138286	PD
cg07536762	-0.39418	4.088994	-6.17379	4.94E-08	5.94E-05	8.132733	PD
cg07036796	-0.36821	3.685717	-6.17254	4.97E-08	5.96E-05	8.080277	PD
cg04354128	0.380613	3.563546	6.171355	4.99E-08	5.98E-05	8.121015	PD

cg15160746	-0.2948	4.059821	-6.16858	5.05E-08	6.04E-05	8.0804	PD
cg23769996	-0.28941	4.291439	-6.1675	5.07E-08	6.06E-05	8.103053	PD
cg00525828	-1.85853	6.236196	-6.16582	5.10E-08	6.09E-05	8.081421	PD
cg15894103	-0.25183	1.960066	-6.16541	5.11E-08	6.09E-05	8.060936	PD
cg10902548	-0.44839	3.790565	-6.16252	5.17E-08	6.14E-05	8.087306	PD
cg21517812	-0.58382	3.13769	-6.16244	5.17E-08	6.14E-05	8.070777	PD
cg11478607	-1.30102	-5.84564	-6.15952	5.23E-08	6.21E-05	8.065912	PD
cg17336044	0.806223	4.055883	6.159154	5.24E-08	6.21E-05	7.996232	PD
cg08323123	0.720061	3.4588	6.158804	5.25E-08	6.21E-05	8.007865	PD
cg18313051	-0.43559	4.19122	-6.15636	5.30E-08	6.26E-05	8.002992	PD
cg07423892	-0.23479	3.86636	-6.15518	5.32E-08	6.28E-05	8.035676	PD
cg02730364	-0.46227	3.916089	-6.15011	5.43E-08	6.40E-05	8.022062	PD
cg27253603	-0.32637	3.984312	-6.149	5.46E-08	6.42E-05	8.016906	PD
cg08397999	-0.31864	3.328149	-6.1471	5.50E-08	6.46E-05	8.031707	PD
cg12280692	1.236166	5.961937	6.142499	5.60E-08	6.57E-05	7.900149	PD
cg00779294	0.369462	3.746233	6.140272	5.65E-08	6.62E-05	7.962109	PD
cg09894294	-0.35978	2.323899	-6.13921	5.67E-08	6.64E-05	7.99233	PD
cg11735305	-0.64464	-6.11985	-6.13877	5.68E-08	6.64E-05	7.942613	PD
cg27617640	-0.2569	3.880481	-6.13803	5.70E-08	6.65E-05	7.951413	PD
cg15588177	-0.29709	4.185992	-6.1366	5.73E-08	6.68E-05	7.974397	PD
cg00290994	-0.2134	-0.92557	-6.13459	5.78E-08	6.72E-05	7.908709	PD
cg04501493	-0.39446	3.040002	-6.13379	5.80E-08	6.73E-05	7.907781	PD
cg12820310	-0.34185	4.060431	-6.13081	5.86E-08	6.80E-05	7.976407	PD
cg13614741	-0.67521	6.351522	-6.13054	5.87E-08	6.80E-05	7.969824	PD
cg07894204	0.370216	3.64083	6.129876	5.89E-08	6.81E-05	7.930628	PD
cg23629809	-0.48754	3.262286	-6.12908	5.91E-08	6.82E-05	7.896308	PD
cg27083395	-0.3799	4.208916	-6.12834	5.92E-08	6.83E-05	7.975552	PD
cg14475857	-0.29404	3.882022	-6.1257	5.99E-08	6.90E-05	7.930581	PD
cg16897387	-0.97314	3.1148	-6.12522	6.00E-08	6.90E-05	7.904965	PD
cg18708796	-0.42038	4.22514	-6.11986	6.13E-08	7.04E-05	7.88878	PD
cg01901290	-0.43663	3.608983	-6.11863	6.16E-08	7.06E-05	7.935715	PD

cg14283647	0.365348	4.02147	6.118398	6.16E-08	7.06E-05	7.871985	PD
cg14830033	-0.37514	3.431862	-6.11647	6.21E-08	7.11E-05	7.898778	PD
cg05679229	-0.26877	2.383684	-6.11405	6.27E-08	7.16E-05	7.876964	PD
cg03382278	-0.3377	3.972759	-6.11392	6.27E-08	7.16E-05	7.873375	PD
cg03492634	0.328341	2.604944	6.113331	6.29E-08	7.17E-05	7.75242	PD
cg19245663	-0.27614	3.602797	-6.10979	6.38E-08	7.26E-05	7.857321	PD
cg19743728	0.380927	3.896941	6.106655	6.46E-08	7.34E-05	7.89817	PD
cg04617569	-0.31002	4.120038	-6.10307	6.55E-08	7.43E-05	7.80194	PD
cg11040238	0.444925	3.346619	6.102909	6.55E-08	7.43E-05	7.82464	PD
cg08537289	0.610025	4.143896	6.100984	6.60E-08	7.48E-05	7.866906	PD
cg12420787	-0.27657	4.131275	-6.10054	6.61E-08	7.48E-05	7.857773	PD
cg24722720	-0.45226	4.154141	-6.09977	6.63E-08	7.49E-05	7.848438	PD
cg11879814	0.609955	3.580091	6.098587	6.67E-08	7.52E-05	7.761628	PD
cg21146652	-0.36407	4.345673	-6.09772	6.69E-08	7.53E-05	7.803598	PD
cg02312559	0.388614	3.495896	6.092219	6.84E-08	7.69E-05	7.83517	PD
cg24328002	-0.4146	2.263898	-6.09066	6.88E-08	7.73E-05	7.759066	PD
cg24934858	-0.34266	3.165127	-6.08947	6.91E-08	7.75E-05	7.81238	PD
cg00001593	-0.22273	3.797693	-6.0879	6.95E-08	7.79E-05	7.810234	PD
cg08128889	-0.26405	3.847278	-6.08743	6.97E-08	7.79E-05	7.81295	PD
cg16920828	-0.71695	3.812286	-6.08277	7.10E-08	7.93E-05	7.716648	PD
cg01208126	-0.75319	3.963687	-6.08115	7.14E-08	7.97E-05	7.727864	PD
cg23878202	-0.3781	4.507693	-6.08059	7.16E-08	7.98E-05	7.774483	PD
cg12960305	0.332462	-4.49752	6.078434	7.22E-08	8.03E-05	7.772491	PD
cg22887166	-0.60104	1.921443	-6.07724	7.25E-08	8.06E-05	7.738094	PD
cg23373087	0.28182	2.094523	6.073228	7.37E-08	8.18E-05	7.771249	PD
cg07389958	-0.52379	4.284923	-6.07234	7.40E-08	8.20E-05	7.758633	PD
cg03273069	1.084104	4.085785	6.067731	7.53E-08	8.34E-05	7.669726	PD
cg18055075	-0.23828	3.528746	-6.06414	7.64E-08	8.45E-05	7.723209	PD
cg22865908	-0.32958	3.425437	-6.0638	7.65E-08	8.45E-05	7.711098	PD
cg26483669	0.255539	2.225597	6.061769	7.71E-08	8.50E-05	7.69952	PD
cg23231268	-0.38155	6.16369	-6.05718	7.85E-08	8.64E-05	7.644619	PD

cg09132376	0.759057	3.74531	6.057024	7.86E-08	8.64E-05	7.63326	PD
cg12469277	-0.60982	3.615214	-6.05546	7.91E-08	8.69E-05	7.581955	PD
cg12720148	-0.47949	4.632127	-6.05401	7.95E-08	8.72E-05	7.652739	PD
cg02228118	-0.25665	2.855507	-6.05134	8.04E-08	8.81E-05	7.695193	PD
cg20574502	1.897853	-6.2813	6.050894	8.05E-08	8.81E-05	7.681307	PD
cg02448395	-0.39148	3.46431	-6.04678	8.18E-08	8.94E-05	7.671272	PD
cg20441035	-0.48823	3.895008	-6.04462	8.25E-08	9.01E-05	7.647774	PD
cg08960812	-0.50384	4.127739	-6.03976	8.41E-08	9.17E-05	7.595502	PD
cg15974300	0.708888	3.756001	6.038517	8.46E-08	9.19E-05	7.617885	PD
cg10773082	-0.51124	5.231289	-6.03847	8.46E-08	9.19E-05	7.619876	PD
cg26336213	0.404202	4.579865	6.036347	8.53E-08	9.26E-05	7.628286	PD
cg24305906	-0.27221	1.803393	-6.03384	8.61E-08	9.33E-05	7.568557	PD
cg22350200	0.495677	4.123128	6.033778	8.62E-08	9.33E-05	7.630186	PD
cg07863834	0.389518	3.952946	6.033388	8.63E-08	9.33E-05	7.573754	PD
cg08219786	-0.40223	4.043059	-6.03289	8.65E-08	9.34E-05	7.596342	PD
cg05629169	0.670414	3.933478	6.025935	8.89E-08	9.58E-05	7.572015	PD
cg00302003	0.274673	4.012852	6.025786	8.89E-08	9.58E-05	7.5736	PD
cg24137123	-0.6728	3.434791	-6.02488	8.93E-08	9.60E-05	7.560056	PD
cg03180148	-0.27948	3.309968	-6.02415	8.95E-08	9.62E-05	7.559734	PD
cg24141001	-0.92946	3.009404	-6.02291	9.00E-08	9.65E-05	7.566782	PD
cg00042080	0.355091	4.510055	6.022287	9.02E-08	9.67E-05	7.553317	PD
cg12130840	0.524786	3.250505	6.021658	9.04E-08	9.67E-05	7.495434	PD
cg04881348	0.618061	-5.4447	6.021483	9.05E-08	9.67E-05	7.596129	PD
cg14464551	-0.32225	3.20197	-6.02087	9.07E-08	9.68E-05	7.547814	PD
cg14711243	-0.7192	-4.47829	-6.01953	9.12E-08	9.70E-05	7.500314	PD
cg00377727	1.602952	4.404582	6.019186	9.13E-08	9.70E-05	7.525209	PD
cg14945473	-0.98467	-4.46648	-6.01911	9.13E-08	9.70E-05	7.52857	PD
cg26403285	0.508496	4.002434	6.0185	9.15E-08	9.70E-05	7.541769	PD
cg04858267	-0.33339	2.728641	-6.01848	9.15E-08	9.70E-05	7.54212	PD
cg04486301	-0.2603	2.837922	-6.01831	9.16E-08	9.70E-05	7.525105	PD
cg10991855	-2.41299	4.348422	-6.01811	9.17E-08	9.70E-05	7.530359	PD

cg05025732	-0.38	2.591913	-6.01663	9.22E-08	9.75E-05	7.499171	PD
cg20036207	0.538925	3.78751	6.013672	9.33E-08	9.83E-05	7.551239	PD
cg14506194	0.826081	3.566205	6.013657	9.33E-08	9.83E-05	7.507761	PD
cg27129744	1.186454	-4.11429	6.013579	9.33E-08	9.83E-05	7.500393	PD
cg13302054	0.385553	4.102842	6.012184	9.39E-08	9.87E-05	7.522284	PD
cg19503586	0.25174	4.449036	6.011297	9.42E-08	9.90E-05	7.546408	PD
cg15544633	0.391396	-3.67144	6.009978	9.47E-08	9.93E-05	7.431589	PD
cg10099574	-0.55865	3.755318	-6.00141	9.79E-08	0.000103	7.491217	PD
cg06953717	-0.29118	4.04338	-5.99957	9.86E-08	0.000103	7.471849	PD
cg05224424	-0.35713	4.218313	-5.99913	9.88E-08	0.000103	7.441536	PD
cg04781238	-0.36661	4.405333	-5.99895	9.89E-08	0.000103	7.437834	PD
cg13337135	-0.2297	4.026504	-5.99828	9.92E-08	0.000103	7.483245	PD
cg17831694	-0.53342	4.847281	-5.99617	1.00E-07	0.000104	7.47623	PD
cg16503053	0.433204	3.294788	5.99589	1.00E-07	0.000104	7.388769	PD
cg23129178	-0.85914	4.68943	-5.9953	1.00E-07	0.000104	7.42701	PD
cg26417346	-0.26345	3.711922	-5.99398	1.01E-07	0.000105	7.457775	PD
cg14181874	2.624777	5.374561	5.991906	1.02E-07	0.000105	7.462039	PD
cg24882192	-0.21296	3.848872	-5.98816	1.03E-07	0.000107	7.439863	PD
cg15090119	-0.24025	3.836406	-5.98581	1.04E-07	0.000108	7.435285	PD
cg11180789	-0.19403	3.709484	-5.98435	1.05E-07	0.000108	7.419117	PD
cg13264811	0.463084	3.116309	5.984114	1.05E-07	0.000108	7.344648	PD
cg01418885	0.556243	-4.48165	5.981258	1.06E-07	0.000109	7.375045	PD
cg12324426	-0.41885	4.42463	-5.98034	1.06E-07	0.000109	7.411127	PD
cg18695931	-0.24987	4.005067	-5.98018	1.07E-07	0.000109	7.423801	PD
cg08700141	1.111106	3.32038	5.975414	1.09E-07	0.000111	7.332562	PD
cg26378179	-0.31497	3.520892	-5.97434	1.09E-07	0.000112	7.36746	PD
cg21789615	-0.3824	3.085585	-5.97382	1.09E-07	0.000112	7.404928	PD
cg10262389	-0.39372	4.080794	-5.9719	1.10E-07	0.000113	7.395754	PD
cg09363218	-0.40551	4.018167	-5.97114	1.10E-07	0.000113	7.374614	PD
cg13724780	-0.67289	-4.76216	-5.96591	1.13E-07	0.000115	7.345813	PD
cg06355720	0.293827	2.946499	5.965825	1.13E-07	0.000115	7.375713	PD

cg03079981	-0.83229	2.700017	-5.96267	1.14E-07	0.000116	7.255335	PD
cg09298628	0.33879	3.65664	5.962141	1.14E-07	0.000116	7.366186	PD
cg16579940	-0.54177	3.831131	-5.96149	1.15E-07	0.000116	7.327715	PD
cg12411992	-0.53882	3.281303	-5.96098	1.15E-07	0.000117	7.341364	PD
cg05927229	-0.52442	2.994133	-5.95908	1.16E-07	0.000117	7.308965	PD
cg09567282	-0.27499	2.492153	-5.95792	1.16E-07	0.000118	7.304621	PD
cg14874216	0.9107	3.111946	5.95608	1.17E-07	0.000118	7.276682	PD
cg16333269	-0.23407	3.614499	-5.95569	1.17E-07	0.000118	7.329269	PD
cg21920469	0.492185	4.012454	5.953438	1.18E-07	0.000119	7.225196	PD
cg26284388	-0.67373	3.9169	-5.95289	1.19E-07	0.000119	7.287193	PD
cg20245641	0.560197	-4.39558	5.952054	1.19E-07	0.00012	7.308263	PD
cg16271481	-0.32363	4.189592	-5.9515	1.19E-07	0.00012	7.272383	PD
cg23458898	-0.79546	4.493681	-5.94815	1.21E-07	0.000121	7.285428	PD
cg04819611	-0.61246	3.735438	-5.94558	1.22E-07	0.000122	7.27789	PD
cg19115205	-0.24077	3.787845	-5.94311	1.23E-07	0.000123	7.187423	PD
cg09740308	-0.84056	-5.77502	-5.94199	1.24E-07	0.000124	7.269626	PD
cg23273453	1.091195	-5.72873	5.938568	1.26E-07	0.000125	7.226013	PD
cg27655855	0.498014	3.599826	5.935347	1.27E-07	0.000127	7.249775	PD
cg23828814	-0.40502	6.247014	-5.9335	1.28E-07	0.000128	7.18801	PD
cg21223341	-0.28112	4.133434	-5.92975	1.30E-07	0.000129	7.229043	PD
cg13424603	0.363873	3.842257	5.929083	1.30E-07	0.000129	7.192363	PD
cg06547209	0.532016	3.134163	5.928664	1.31E-07	0.00013	7.188002	PD
cg07720053	0.398345	2.29859	5.927554	1.31E-07	0.00013	7.179193	PD
cg00393487	-0.50502	3.603873	-5.92462	1.33E-07	0.000131	7.206823	PD
cg22939408	-0.22843	3.766794	-5.92034	1.35E-07	0.000133	7.169075	PD
cg05522180	0.862446	4.002025	5.915123	1.38E-07	0.000136	7.133374	PD
cg24725640	0.258275	4.396693	5.915079	1.38E-07	0.000136	7.181796	PD
cg08165083	-0.23583	3.052313	-5.91411	1.38E-07	0.000136	7.17902	PD
cg04585937	-0.29775	3.043126	-5.91396	1.38E-07	0.000136	7.182616	PD
cg00475456	0.315848	4.449501	5.909896	1.41E-07	0.000138	7.140344	PD
cg23886495	0.322256	-5.3365	5.908189	1.41E-07	0.000139	7.168923	PD

cg14036303	-0.20752	4.355088	-5.90714	1.42E-07	0.000139	7.159797	PD
cg24775327	-0.43319	2.986134	-5.90644	1.42E-07	0.00014	7.151007	PD
cg09615585	-0.38338	4.597934	-5.90218	1.45E-07	0.000142	7.151426	PD
cg26446056	-0.44038	3.634485	-5.9008	1.46E-07	0.000142	7.117848	PD
cg00356334	0.233371	4.404777	5.897596	1.47E-07	0.000144	7.13885	PD
cg16785502	-0.5122	4.189968	-5.89677	1.48E-07	0.000144	7.103698	PD
cg03240883	-0.23911	4.120063	-5.89466	1.49E-07	0.000145	7.117423	PD
cg23150798	-0.26266	3.911721	-5.89262	1.50E-07	0.000146	7.098044	PD
cg04567756	-0.44657	4.256693	-5.89248	1.50E-07	0.000146	7.108926	PD
cg16576544	0.703056	3.078495	5.891386	1.51E-07	0.000147	7.101546	PD
cg03654147	-0.22643	4.505588	-5.89134	1.51E-07	0.000147	7.016466	PD
cg22989407	-0.27969	5.4243	-5.88739	1.54E-07	0.000149	7.071818	PD
cg14332813	-0.34388	3.594694	-5.88611	1.54E-07	0.000149	7.091674	PD
cg17898762	-0.25511	3.754783	-5.88545	1.55E-07	0.00015	7.085802	PD
cg25964180	-0.25429	4.055208	-5.88487	1.55E-07	0.00015	7.092673	PD
cg19523505	-0.23838	2.786747	-5.88486	1.55E-07	0.00015	7.044981	PD
cg11751810	-0.44255	3.458686	-5.8836	1.56E-07	0.00015	7.064494	PD
cg07319199	-1.25329	3.506625	-5.88277	1.56E-07	0.00015	6.990737	PD
cg01799902	0.336929	4.576624	5.882399	1.57E-07	0.00015	7.046882	PD
cg13597585	0.356479	4.584785	5.881625	1.57E-07	0.000151	7.064414	PD
cg19806292	-0.34864	4.002108	-5.88059	1.58E-07	0.000151	7.067144	PD
cg12321193	-0.58283	4.314855	-5.88	1.58E-07	0.000151	7.03722	PD
cg03033873	-0.32552	2.843976	-5.87986	1.58E-07	0.000151	7.031564	PD
cg17927096	-0.26957	2.776837	-5.876	1.61E-07	0.000153	7.026487	PD
cg14019393	0.256385	4.133615	5.861892	1.70E-07	0.000162	6.985963	PD
cg17693656	-0.32451	4.250098	-5.85971	1.71E-07	0.000163	6.987809	PD
cg09677856	-1.02282	4.7442	-5.85825	1.72E-07	0.000164	6.941082	PD
cg12256941	-0.26007	3.590396	-5.85485	1.74E-07	0.000166	6.961832	PD
cg27045724	0.67519	-5.57154	5.854655	1.75E-07	0.000166	6.913398	PD
cg00535430	-0.18793	4.680656	-5.85301	1.76E-07	0.000167	6.965577	PD
cg12777474	0.510494	3.040331	5.852149	1.76E-07	0.000167	6.951253	PD

cg07245624	-0.30506	3.269828	-5.85118	1.77E-07	0.000168	6.950251	PD
cg04060288	-0.70897	3.757375	-5.84988	1.78E-07	0.000168	6.954816	PD
cg26212163	-0.51569	-4.32028	-5.84911	1.78E-07	0.000169	6.921941	PD
cg03624707	0.335815	4.143188	5.848364	1.79E-07	0.000169	6.943032	PD
cg06515049	0.493356	2.887099	5.844303	1.82E-07	0.000171	6.92372	PD
cg27273264	-0.28951	2.80275	-5.83969	1.85E-07	0.000174	6.891091	PD
cg00540792	-0.46547	4.25978	-5.83835	1.86E-07	0.000175	6.916045	PD
cg13790719	-0.75623	-4.76733	-5.83798	1.86E-07	0.000175	6.91669	PD
cg10913457	-0.31098	4.193191	-5.8369	1.87E-07	0.000176	6.912337	PD
cg00319741	-0.56374	3.429719	-5.83656	1.87E-07	0.000176	6.886411	PD
cg14328273	0.319051	4.153487	5.835786	1.88E-07	0.000176	6.909366	PD
cg01044608	-1.10887	-4.79244	-5.83558	1.88E-07	0.000176	6.824079	PD
cg01951573	-0.45236	3.182205	-5.83382	1.90E-07	0.000177	6.893634	PD
cg26233408	-0.40472	4.792556	-5.8317	1.91E-07	0.000178	6.868922	PD
cg13748755	-0.24203	4.227709	-5.82771	1.94E-07	0.000181	6.853164	PD
cg02892043	0.36451	4.199357	5.823591	1.97E-07	0.000184	6.874879	PD
cg17433211	-0.21867	3.666323	-5.8229	1.98E-07	0.000184	6.839234	PD
cg16149820	-2.30363	3.892105	-5.82193	1.99E-07	0.000184	6.787273	PD
cg03292225	-2.44925	4.038517	-5.82173	1.99E-07	0.000184	6.818175	PD
cg06779232	-0.47815	4.007383	-5.81668	2.03E-07	0.000188	6.813334	PD
cg06907539	-0.34483	3.164632	-5.81638	2.03E-07	0.000188	6.815404	PD
cg15536089	-0.34845	4.580544	-5.81486	2.04E-07	0.000189	6.812882	PD
cg26301516	-0.32232	2.916643	-5.81348	2.05E-07	0.00019	6.803488	PD
cg00355799	-0.27636	4.310591	-5.81154	2.07E-07	0.000191	6.809306	PD
cg06382562	0.681018	3.182026	5.810477	2.08E-07	0.000192	6.781249	PD
cg07668809	-0.36255	3.402147	-5.80962	2.08E-07	0.000192	6.799461	PD
cg22022041	-0.3865	3.729979	-5.80877	2.09E-07	0.000192	6.685269	PD
cg05747582	0.511852	4.28042	5.807502	2.10E-07	0.000193	6.764106	PD
cg26598859	0.340395	2.985212	5.807359	2.10E-07	0.000193	6.793712	PD
cg21544644	0.288045	2.338604	5.807218	2.10E-07	0.000193	6.797714	PD
cg19245511	0.288042	-4.78752	5.805487	2.12E-07	0.000194	6.757197	PD

cg21729825	-0.30973	4.376317	-5.80543	2.12E-07	0.000194	6.794174	PD
cg19802542	-0.21435	3.898161	-5.8054	2.12E-07	0.000194	6.790149	PD
cg05393343	-0.25903	3.832751	-5.80441	2.13E-07	0.000194	6.7932	PD
cg01691989	-0.17743	6.104298	-5.80034	2.16E-07	0.000197	6.748703	PD
cg20571761	0.667033	4.964504	5.798928	2.17E-07	0.000198	6.716687	PD
cg26987049	-0.40543	4.044171	-5.79424	2.21E-07	0.000202	6.758351	PD
cg00994032	-0.2697	3.401311	-5.79339	2.22E-07	0.000202	6.744736	PD
cg17301160	0.827589	3.468644	5.793357	2.22E-07	0.000202	6.719068	PD
cg26537272	-0.27431	4.056406	-5.79238	2.23E-07	0.000202	6.7608	PD
cg04145937	-0.43172	3.346412	-5.79212	2.23E-07	0.000202	6.708402	PD
cg09564679	-0.31079	4.369053	-5.79142	2.24E-07	0.000203	6.755278	PD
cg18260555	0.220008	3.039323	5.790404	2.25E-07	0.000203	6.702565	PD
cg15877883	0.648005	-5.82143	5.78965	2.25E-07	0.000204	6.756233	PD
cg22386646	-0.2097	4.206299	-5.78724	2.27E-07	0.000205	6.729725	PD
cg21803535	-0.23783	3.984026	-5.78434	2.30E-07	0.000207	6.697187	PD
cg05194552	-0.27068	3.208386	-5.78318	2.31E-07	0.000208	6.724025	PD
cg01960445	0.351952	4.115422	5.782278	2.32E-07	0.000209	6.621757	PD
cg09847728	-0.38117	3.393734	-5.78223	2.32E-07	0.000209	6.729707	PD
cg27000335	-0.91796	2.094235	-5.78077	2.33E-07	0.000209	6.707746	PD
cg07547996	-0.38836	3.430423	-5.77665	2.37E-07	0.000213	6.702001	PD
cg11730435	-0.36944	4.196644	-5.77648	2.37E-07	0.000213	6.678618	PD
cg19114576	-0.2204	2.576268	-5.77358	2.40E-07	0.000215	6.66984	PD
cg14512491	-0.22766	3.36879	-5.77218	2.41E-07	0.000216	6.666451	PD
cg00257817	0.388087	4.355904	5.771278	2.42E-07	0.000216	6.692113	PD
cg23114211	0.442224	3.807979	5.76458	2.49E-07	0.000222	6.664468	PD
cg25203856	0.430759	3.615098	5.76335	2.50E-07	0.000223	6.641406	PD
cg04706801	0.689876	4.082611	5.762509	2.51E-07	0.000223	6.650953	PD
cg25893357	0.189937	-6.28583	5.758485	2.55E-07	0.000226	6.605081	PD
cg19812670	0.46686	3.537538	5.758467	2.55E-07	0.000226	6.638322	PD
cg17020263	-0.39176	3.99068	-5.75706	2.56E-07	0.000227	6.603715	PD
cg15248037	-0.47463	2.98133	-5.75538	2.58E-07	0.000228	6.511215	PD

cg16517367	-0.19181	3.509694	-5.75421	2.59E-07	0.000229	6.578254	PD
cg06566454	-0.3366	2.161621	-5.75226	2.61E-07	0.000231	6.570873	PD
cg02807146	-0.36049	-5.4648	-5.75096	2.62E-07	0.000232	6.601171	PD
cg11699065	0.728519	3.90048	5.750317	2.63E-07	0.000232	6.561719	PD
cg08942192	0.301837	4.472045	5.749722	2.63E-07	0.000232	6.459549	PD
cg08906366	-0.20842	3.509523	-5.7483	2.65E-07	0.000233	6.574517	PD
cg13935765	0.276948	3.870273	5.747225	2.66E-07	0.000234	6.563025	PD
cg05248081	-0.23705	4.123855	-5.74706	2.66E-07	0.000234	6.57555	PD
cg13700458	0.244541	4.093079	5.746849	2.66E-07	0.000234	6.589416	PD
cg22273955	0.321297	4.661589	5.745222	2.68E-07	0.000235	6.545868	PD
cg15069850	-0.30132	3.963806	-5.74376	2.70E-07	0.000236	6.559675	PD
cg23951171	-0.40626	4.080374	-5.74317	2.70E-07	0.000237	6.561422	PD
cg01896130	-0.35586	3.506727	-5.74282	2.71E-07	0.000237	6.583671	PD
cg06552546	-0.2872	2.919148	-5.74251	2.71E-07	0.000237	6.57313	PD
cg17311420	-0.34806	3.468154	-5.74241	2.71E-07	0.000237	6.577368	PD
cg20656154	0.449173	4.071528	5.738809	2.75E-07	0.00024	6.534515	PD
cg10306658	-0.70466	4.198212	-5.73763	2.76E-07	0.000241	6.542803	PD
cg00756172	-0.40087	4.262026	-5.73659	2.77E-07	0.000241	6.463981	PD
cg22622658	-0.28235	3.874817	-5.73636	2.78E-07	0.000241	6.544665	PD
cg24618875	-0.26914	3.637063	-5.73544	2.79E-07	0.000242	6.542115	PD
cg04085039	-0.2394	3.715432	-5.73464	2.79E-07	0.000242	6.55313	PD
cg14921169	0.486846	-4.75881	5.734178	2.80E-07	0.000243	6.548275	PD
cg16786268	-0.50449	4.169435	-5.72956	2.85E-07	0.000247	6.532144	PD
cg19001113	-0.20918	4.238418	-5.72661	2.88E-07	0.000249	6.512606	PD
cg14823083	-0.35348	2.921005	-5.72527	2.90E-07	0.00025	6.497226	PD
cg19709737	0.51906	3.965668	5.725021	2.90E-07	0.00025	6.511963	PD
cg00020438	0.7137	3.722805	5.724742	2.90E-07	0.00025	6.490128	PD
cg02163033	-0.29255	3.753063	-5.72457	2.91E-07	0.00025	6.513531	PD
cg14231326	-0.38476	4.352082	-5.7227	2.93E-07	0.000252	6.492414	PD
cg20009356	-0.44892	5.070019	-5.72076	2.95E-07	0.000254	6.484958	PD
cg00459550	-0.20392	3.590755	-5.71985	2.96E-07	0.000254	6.487194	PD

cg06385407	0.274283	3.588571	5.719682	2.96E-07	0.000254	6.480677	PD
cg04089444	-0.99887	4.676054	-5.71905	2.97E-07	0.000255	6.460185	PD
cg15302407	-0.26561	2.306121	-5.71777	2.98E-07	0.000256	6.466075	PD
cg17628903	-0.34978	4.242736	-5.71627	3.00E-07	0.000257	6.491035	PD
cg04582206	-0.29417	3.673385	-5.71552	3.01E-07	0.000257	6.484201	PD
cg06950937	1.233911	3.401617	5.714206	3.03E-07	0.000258	6.455065	PD
cg27581211	-0.23881	3.523655	-5.71283	3.04E-07	0.000259	6.453786	PD
cg23450922	-0.25666	2.413989	-5.71266	3.05E-07	0.000259	6.455064	PD
cg16395248	-0.30947	4.316388	-5.71166	3.06E-07	0.00026	6.456979	PD
cg05757385	1.029803	-4.23403	5.710292	3.07E-07	0.000261	6.437026	PD
cg17271269	-1.08565	3.463703	-5.70887	3.09E-07	0.000262	6.352169	PD
cg00944794	-0.43076	4.065434	-5.70725	3.11E-07	0.000264	6.405351	PD
cg02390561	-0.26785	3.326327	-5.70649	3.12E-07	0.000264	6.458024	PD
cg18925616	-0.23901	3.079083	-5.70623	3.12E-07	0.000264	6.433165	PD
cg07530005	-0.28784	4.053735	-5.7048	3.14E-07	0.000266	6.413737	PD
cg13269368	-0.5598	3.742094	-5.70444	3.14E-07	0.000266	6.409531	PD
cg13443968	-0.17741	3.56605	-5.70421	3.15E-07	0.000266	6.450282	PD
cg04678404	0.474796	3.653678	5.704002	3.15E-07	0.000266	6.435559	PD
cg22954744	-0.28975	3.638918	-5.70383	3.15E-07	0.000266	6.434362	PD
cg21273370	-0.4211	3.015628	-5.70323	3.16E-07	0.000266	6.400961	PD
cg10449329	-0.22385	3.257318	-5.70306	3.16E-07	0.000266	6.421172	PD
cg06774972	0.361564	2.847606	5.702718	3.17E-07	0.000266	6.376638	PD
cg21758451	-0.24158	4.055974	-5.70174	3.18E-07	0.000267	6.432391	PD
cg15371835	-0.21545	3.826289	-5.70005	3.20E-07	0.000268	6.420895	PD
cg08463839	-0.47895	3.666553	-5.69875	3.21E-07	0.000269	6.389435	PD
cg20889841	0.790129	-6.57136	5.696868	3.24E-07	0.000271	6.424109	PD
cg10318197	-0.57145	5.562413	-5.69486	3.26E-07	0.000273	6.377826	PD
cg06085525	-0.32594	3.25393	-5.69458	3.27E-07	0.000273	6.402039	PD
cg17948614	-0.3261	4.099014	-5.69453	3.27E-07	0.000273	6.392862	PD
cg05172172	-0.45011	3.823867	-5.6932	3.29E-07	0.000274	6.399592	PD
cg03080505	-0.22621	3.056056	-5.6894	3.33E-07	0.000278	6.345161	PD

cg01334818	0.416594	-5.29973	5.689155	3.34E-07	0.000278	6.381673	PD
cg08398691	-0.33964	2.906167	-5.68844	3.35E-07	0.000278	6.358914	PD
cg09318637	-0.26815	3.577542	-5.68839	3.35E-07	0.000278	6.367562	PD
cg01669244	0.35795	3.932028	5.685678	3.38E-07	0.00028	6.365032	PD
cg02546601	-0.57111	4.540453	-5.68563	3.38E-07	0.00028	6.383686	PD
cg07338041	-0.55426	4.123805	-5.68468	3.40E-07	0.000281	6.345698	PD
cg04576491	-0.35112	3.469579	-5.68416	3.40E-07	0.000281	6.33232	PD
cg12062936	-0.2261	3.961551	-5.68409	3.40E-07	0.000281	6.360913	PD
cg01911981	0.902918	5.015288	5.682199	3.43E-07	0.000283	6.34429	PD
cg05526565	-0.44959	3.864233	-5.68193	3.43E-07	0.000283	6.354983	PD
cg16326826	-0.38295	4.430194	-5.68182	3.43E-07	0.000283	6.363918	PD
cg08293614	-1.54511	3.451229	-5.68066	3.45E-07	0.000284	6.320594	PD
cg12338576	0.446527	3.595234	5.67858	3.48E-07	0.000286	6.342355	PD
cg15070960	1.032595	3.95437	5.674219	3.54E-07	0.00029	6.207635	PD
cg17373109	-0.41218	2.235054	-5.67319	3.55E-07	0.000291	6.332899	PD
cg01419361	0.51088	3.940618	5.673043	3.55E-07	0.000291	6.342343	PD
cg04499147	-0.40689	1.756231	-5.67054	3.59E-07	0.000294	6.323024	PD
cg15852440	0.666381	2.741702	5.66885	3.61E-07	0.000295	6.27248	PD
cg17682590	-0.30984	4.145131	-5.66765	3.63E-07	0.000297	6.283501	PD
cg14379850	-0.30065	3.574883	-5.6665	3.65E-07	0.000298	6.296138	PD
cg06013974	0.267326	4.187218	5.665362	3.66E-07	0.000299	6.28387	PD
cg10544093	0.44087	-5.06056	5.663957	3.68E-07	0.0003	6.282823	PD
cg06180061	-0.25434	2.434682	-5.66383	3.68E-07	0.0003	6.287996	PD
cg24796010	-0.29716	4.264531	-5.66318	3.69E-07	0.0003	6.294353	PD
cg18959065	-0.4176	3.261972	-5.65832	3.76E-07	0.000306	6.28169	PD
cg20426275	0.868628	3.49689	5.657762	3.77E-07	0.000306	6.230136	PD
cg02444715	-0.37397	2.645938	-5.65771	3.77E-07	0.000306	6.280792	PD
cg10741696	-0.30551	4.009013	-5.65754	3.77E-07	0.000306	6.29367	PD
cg19185092	-0.70296	-4.81408	-5.65677	3.79E-07	0.000306	6.234852	PD
cg24529615	-0.35779	2.884108	-5.65573	3.80E-07	0.000307	6.227492	PD
cg05871851	-0.45969	2.901985	-5.65424	3.82E-07	0.000309	6.21975	PD

cg25270367	1.017924	3.26721	5.65101	3.87E-07	0.000312	6.229769	PD
cg10406520	0.353258	4.060494	5.650525	3.88E-07	0.000313	6.235486	PD
cg07648641	-0.2592	3.912858	-5.64949	3.90E-07	0.000314	6.243173	PD
cg15846482	-0.65553	-5.13234	-5.64928	3.90E-07	0.000314	6.245972	PD
cg21782309	0.430513	3.623681	5.647857	3.92E-07	0.000315	6.236742	PD
cg12121080	0.367683	3.43264	5.645546	3.96E-07	0.000318	6.238759	PD
cg20482143	-0.24405	-0.96871	-5.64353	3.99E-07	0.00032	6.22443	PD
cg00349608	-0.21916	2.663521	-5.64315	3.99E-07	0.00032	6.194254	PD
cg12071317	-0.32304	3.910651	-5.64025	4.04E-07	0.000323	6.193003	PD
cg18543074	0.369016	4.081946	5.639686	4.05E-07	0.000324	6.193666	PD
cg06885374	0.812401	-5.66169	5.639636	4.05E-07	0.000324	6.16585	PD
cg07158816	-0.26115	3.419513	-5.63859	4.06E-07	0.000325	6.204486	PD
cg05878104	0.582554	-3.53202	5.636753	4.09E-07	0.000327	6.185562	PD
cg26217318	-0.35771	3.842867	-5.63561	4.11E-07	0.000328	6.178182	PD
cg17879774	-0.29599	3.386988	-5.63543	4.11E-07	0.000328	6.178288	PD
cg14332079	-0.60633	-4.6724	-5.63475	4.12E-07	0.000328	6.156578	PD
cg08607266	-0.59438	4.686263	-5.6345	4.13E-07	0.000328	6.167628	PD
cg06386009	-0.38325	2.765269	-5.63123	4.18E-07	0.000332	6.169274	PD
cg00393585	-1.05924	-4.89738	-5.63001	4.20E-07	0.000333	6.128507	PD
cg09931872	0.557139	3.776143	5.629285	4.21E-07	0.000334	6.181335	PD
cg03870432	0.33064	4.122092	5.62769	4.24E-07	0.000336	6.152933	PD
cg14140447	-0.3446	3.095773	-5.6265	4.26E-07	0.000337	6.159082	PD
cg11456854	0.318431	3.59273	5.624559	4.29E-07	0.000339	6.155111	PD
cg18096398	-0.26064	4.327639	-5.62311	4.32E-07	0.000341	6.149506	PD
cg08826854	-0.33651	4.260106	-5.62304	4.32E-07	0.000341	6.142373	PD
cg00447581	0.209717	3.756412	5.621064	4.35E-07	0.000343	6.145742	PD
cg18186496	-0.2794	3.756583	-5.62053	4.36E-07	0.000343	6.121419	PD
cg02459859	-0.37061	4.645399	-5.61815	4.40E-07	0.000346	6.138312	PD
cg02108367	-1.26563	3.426751	-5.61637	4.43E-07	0.000348	6.038836	PD
cg05716270	-0.2954	2.371073	-5.61315	4.49E-07	0.000352	6.102665	PD
cg03545635	-0.32686	4.060911	-5.61233	4.50E-07	0.000353	6.072655	PD

cg16639595	-0.42932	3.616522	-5.61148	4.52E-07	0.000354	6.091311	PD
cg08038637	-0.3215	3.234527	-5.60977	4.55E-07	0.000356	6.058081	PD
cg10487316	0.50252	-4.00069	5.609594	4.55E-07	0.000356	6.101403	PD
cg22401033	0.592655	3.107915	5.608939	4.56E-07	0.000356	6.038895	PD
cg26474732	0.784709	3.159425	5.607992	4.58E-07	0.000357	6.08581	PD
cg06135487	-0.55232	3.583856	-5.6028	4.67E-07	0.000364	6.063339	PD
cg06350542	1.426688	5.184021	5.601501	4.69E-07	0.000366	6.049885	PD
cg08131310	-0.37549	4.31756	-5.60125	4.70E-07	0.000366	6.060848	PD
cg07510270	-0.22828	3.109071	-5.60112	4.70E-07	0.000366	6.052471	PD
cg11525047	-1.0214	4.260256	-5.59981	4.72E-07	0.000367	6.065956	PD
cg01195881	-0.66489	4.49567	-5.59903	4.74E-07	0.000368	6.051666	PD
cg05127995	-0.23505	2.198779	-5.59767	4.76E-07	0.00037	6.032007	PD
cg05831083	-0.39526	3.6223	-5.59561	4.80E-07	0.000372	6.025353	PD
cg07938921	0.410252	4.339247	5.594292	4.83E-07	0.000374	6.029101	PD
cg08458132	0.85515	3.387698	5.594229	4.83E-07	0.000374	6.004208	PD
cg17331757	-0.41984	3.303726	-5.59372	4.84E-07	0.000374	6.043938	PD
cg02674186	0.799466	4.497855	5.592562	4.86E-07	0.000375	6.012111	PD
cg03547368	-0.21145	4.258479	-5.59168	4.88E-07	0.000376	6.025027	PD
cg06034807	0.493719	-4.99441	5.590394	4.90E-07	0.000378	6.023303	PD
cg12897690	1.306366	3.867665	5.588854	4.93E-07	0.00038	5.996256	PD
cg24747107	-0.31285	3.785936	-5.58832	4.94E-07	0.00038	6.014036	PD
cg15584430	-0.20593	3.323567	-5.58663	4.97E-07	0.000382	6.008136	PD
cg21605333	-0.50213	-5.15272	-5.58584	4.99E-07	0.000383	6.003082	PD
cg02822944	-1.24791	3.480972	-5.58553	4.99E-07	0.000383	5.935348	PD
cg05445477	-0.22692	4.052537	-5.58411	5.02E-07	0.000385	6.000824	PD
cg17329110	-0.19439	3.311638	-5.58189	5.06E-07	0.000388	5.992398	PD
cg09461420	-0.26317	3.292495	-5.57915	5.12E-07	0.000392	5.995251	PD
cg19423485	0.426652	3.76305	5.578268	5.14E-07	0.000393	5.988102	PD
cg01728951	-0.23837	4.394032	-5.57805	5.14E-07	0.000393	5.985228	PD
cg05282260	-0.46775	2.900612	-5.57652	5.17E-07	0.000395	5.988175	PD
cg14075211	-0.26664	4.007113	-5.57444	5.21E-07	0.000398	5.996265	PD

cg00046991	-0.4437	4.322078	-5.57232	5.26E-07	0.0004	5.968344	PD
cg14397242	-0.27279	3.966853	-5.57224	5.26E-07	0.0004	5.934219	PD
cg00852518	-0.27226	4.001539	-5.57182	5.27E-07	0.000401	5.965915	PD
cg18168751	-0.21488	3.855685	-5.57125	5.28E-07	0.000401	5.973611	PD
cg11650164	-0.39332	4.46583	-5.5691	5.32E-07	0.000404	5.940871	PD
cg04976254	-0.19929	3.806096	-5.56838	5.34E-07	0.000405	5.969701	PD
cg19982232	-0.43923	4.031954	-5.56693	5.37E-07	0.000407	5.953904	PD
cg25795711	-0.34836	3.895898	-5.56617	5.38E-07	0.000407	5.958209	PD
cg15624719	-0.48122	2.773242	-5.56604	5.39E-07	0.000407	5.904627	PD
cg05824180	0.525014	3.55584	5.563687	5.43E-07	0.000411	5.938553	PD
cg15230952	-0.58263	3.332835	-5.56343	5.44E-07	0.000411	5.918773	PD
cg02896926	-0.83935	3.329728	-5.5632	5.45E-07	0.000411	5.927759	PD
cg21023093	-0.33015	3.98229	-5.56188	5.47E-07	0.000413	5.947842	PD
cg11067736	0.340663	-6.60255	5.559698	5.52E-07	0.000416	5.940386	PD
cg16028310	-0.39577	4.488927	-5.55927	5.53E-07	0.000416	5.898504	PD
cg24976563	-2.84312	4.581181	-5.55924	5.53E-07	0.000416	5.862697	PD
cg20064778	-1.80485	-5.44441	-5.55899	5.53E-07	0.000416	5.907506	PD
cg11223723	-0.25887	3.585539	-5.55853	5.54E-07	0.000416	5.934581	PD
cg11516226	-0.43862	3.118716	-5.55807	5.55E-07	0.000416	5.773963	PD
cg22614372	-0.34132	3.191549	-5.55751	5.57E-07	0.000417	5.902947	PD
cg17561092	-0.45641	3.580181	-5.55612	5.60E-07	0.000419	5.901907	PD
cg07878540	1.316663	-5.68591	5.553861	5.65E-07	0.000422	5.876605	PD
cg09365942	-0.26779	3.479424	-5.55019	5.73E-07	0.000428	5.844622	PD
cg27199099	0.43383	4.223702	5.549712	5.74E-07	0.000428	5.883365	PD
cg03570708	-1.45434	3.366668	-5.54963	5.74E-07	0.000428	5.851311	PD
cg07694806	-0.27536	2.767765	-5.54876	5.76E-07	0.000429	5.859658	PD
cg01099258	-0.61467	3.263331	-5.54657	5.81E-07	0.000432	5.883025	PD
cg06527100	-0.45369	4.77441	-5.54484	5.85E-07	0.000435	5.877636	PD
cg09654261	-0.40986	4.048658	-5.54452	5.85E-07	0.000435	5.873556	PD
cg00807008	-0.3522	3.425128	-5.54348	5.88E-07	0.000436	5.792609	PD
cg20814202	-0.70106	3.755768	-5.54257	5.90E-07	0.000437	5.826296	PD

cg12053296	-0.20736	3.70546	-5.54255	5.90E-07	0.000437	5.883569	PD
cg05868255	-0.49728	3.690776	-5.54174	5.92E-07	0.000438	5.830901	PD
cg24697463	0.251833	3.137117	5.540248	5.95E-07	0.00044	5.85134	PD
cg11143152	-0.30267	4.287096	-5.53851	5.99E-07	0.000443	5.831177	PD
cg16936864	-0.35687	3.743162	-5.53832	6.00E-07	0.000443	5.851618	PD
cg13930739	0.243543	4.211858	5.537766	6.01E-07	0.000443	5.836874	PD
cg08540945	0.534638	-4.58111	5.535608	6.06E-07	0.000447	5.840984	PD
cg21004032	-0.37478	4.090099	-5.53483	6.08E-07	0.000447	5.826973	PD
cg06646587	-0.2337	3.698121	-5.53473	6.08E-07	0.000447	5.825416	PD
cg01160347	-0.40763	3.657796	-5.53459	6.08E-07	0.000447	5.81208	PD
cg04292615	1.169574	-5.64941	5.533739	6.10E-07	0.000448	5.817864	PD
cg07930587	-0.40432	3.621742	-5.53295	6.12E-07	0.000449	5.813413	PD
cg13416895	-0.30549	3.869501	-5.53267	6.13E-07	0.00045	5.828671	PD
cg20914168	-0.19324	4.231085	-5.53242	6.13E-07	0.00045	5.810703	PD
cg25011627	-0.27949	3.479564	-5.53001	6.19E-07	0.000453	5.818404	PD
cg24728257	0.462335	2.939009	5.529235	6.21E-07	0.000454	5.790462	PD
cg13528863	-0.19311	3.28374	-5.52891	6.22E-07	0.000455	5.819618	PD
cg11756968	-0.2399	3.971929	-5.5279	6.24E-07	0.000456	5.792805	PD
cg02197923	-0.29579	3.932653	-5.52692	6.27E-07	0.000457	5.813598	PD
cg23210369	-0.28178	3.414205	-5.52672	6.27E-07	0.000457	5.800154	PD
cg00320696	-0.29398	3.914943	-5.52447	6.33E-07	0.00046	5.798353	PD
cg00583304	-0.22377	3.077304	-5.52429	6.33E-07	0.00046	5.800026	PD
cg12150991	-1.77708	-4.50951	-5.52425	6.33E-07	0.00046	5.78836	PD
cg00019085	1.333741	-6.78339	5.523608	6.35E-07	0.000461	5.789651	PD
cg17640620	-0.31933	4.566624	-5.52344	6.35E-07	0.000461	5.796265	PD
cg18372633	-0.36182	3.977847	-5.52242	6.38E-07	0.000462	5.806127	PD
cg13355253	-0.44291	4.519556	-5.522	6.39E-07	0.000463	5.776571	PD
cg16900705	0.433158	3.410493	5.519774	6.44E-07	0.000466	5.744386	PD
cg16283179	0.190705	2.201881	5.516742	6.52E-07	0.000472	5.773634	PD
cg16696513	0.54604	3.32593	5.514511	6.57E-07	0.000475	5.753979	PD
cg11922616	0.330073	3.495401	5.514215	6.58E-07	0.000475	5.755735	PD

cg03650179	0.33008	4.414529	5.513287	6.60E-07	0.000477	5.749525	PD
cg11633177	-0.26065	2.577483	-5.51268	6.62E-07	0.000477	5.739388	PD
cg15954363	-0.27823	4.455826	-5.51234	6.63E-07	0.000478	5.74498	PD
cg01758046	0.304705	3.923459	5.512076	6.64E-07	0.000478	5.754266	PD
cg13531460	-0.38903	3.372968	-5.50951	6.70E-07	0.000482	5.688152	PD
cg06733558	0.178347	-0.44653	5.508179	6.74E-07	0.000484	5.731173	PD
cg05552814	-0.28196	3.933699	-5.50667	6.78E-07	0.000486	5.74731	PD
cg14182339	-0.42221	2.669423	-5.50585	6.80E-07	0.000488	5.727472	PD
cg15269045	-0.21864	3.763782	-5.50514	6.82E-07	0.000489	5.732166	PD
cg09478051	-0.66476	3.251646	-5.5048	6.82E-07	0.000489	5.642992	PD
cg18438461	-0.31928	2.754876	-5.50458	6.83E-07	0.000489	5.735744	PD
cg19179910	-0.99576	3.707164	-5.50317	6.87E-07	0.000491	5.664544	PD
cg09858237	0.162565	-3.8079	5.501483	6.91E-07	0.000494	5.716842	PD
cg02628513	0.575279	-4.91892	5.500608	6.94E-07	0.000495	5.714546	PD
cg25533397	-0.31925	4.198239	-5.49975	6.96E-07	0.000496	5.684162	PD
cg01717830	-0.60113	-5.12945	-5.49973	6.96E-07	0.000496	5.715508	PD
cg17221143	-0.69962	4.097707	-5.49926	6.97E-07	0.000496	5.708857	PD
cg13122347	-0.82076	3.071397	-5.49925	6.97E-07	0.000496	5.682472	PD
cg23089491	-0.26912	1.822383	-5.49887	6.98E-07	0.000496	5.714637	PD
cg07404352	-0.55844	4.050763	-5.49851	6.99E-07	0.000497	5.66351	PD
cg06655840	-0.24182	4.162529	-5.49715	7.03E-07	0.000499	5.702942	PD
cg07539997	-0.32902	6.030632	-5.49577	7.07E-07	0.000501	5.702343	PD
cg14170201	-0.59834	3.297251	-5.49474	7.10E-07	0.000503	5.690054	PD
cg13007184	-0.261	2.767204	-5.49452	7.10E-07	0.000503	5.689922	PD
cg14204081	0.551237	-3.60482	5.494203	7.11E-07	0.000503	5.656777	PD
cg13146020	0.279937	4.336565	5.493368	7.13E-07	0.000504	5.682824	PD
cg27442805	-0.22261	3.72821	-5.49284	7.15E-07	0.000505	5.704491	PD
cg09222609	0.511418	-4.39129	5.492303	7.16E-07	0.000505	5.684387	PD
cg04965012	0.53307	3.218185	5.492088	7.17E-07	0.000505	5.680261	PD
cg19616844	-0.47753	4.092655	-5.49156	7.18E-07	0.000506	5.666228	PD
cg15268549	0.468166	-5.06876	5.491473	7.18E-07	0.000506	5.660651	PD

cg12141457	-0.25801	2.837389	-5.48931	7.25E-07	0.000509	5.67352	PD
cg15400159	-0.36631	4.347461	-5.4872	7.30E-07	0.000513	5.673255	PD
cg00308503	0.17138	-0.6704	5.485815	7.34E-07	0.000515	5.666499	PD
cg03321222	-0.27594	4.337802	-5.48465	7.38E-07	0.000517	5.662915	PD
cg16496709	0.5082	3.486475	5.484477	7.38E-07	0.000517	5.666509	PD
cg15985875	-0.345	4.023285	-5.48381	7.40E-07	0.000518	5.641236	PD
cg09485007	-0.26016	-5.43277	-5.48259	7.44E-07	0.00052	5.663415	PD
cg01253826	0.307082	-4.72973	5.482381	7.44E-07	0.00052	5.617349	PD
cg06648556	0.511808	3.998341	5.477257	7.59E-07	0.00053	5.649834	PD
cg18650027	-0.27985	4.136091	-5.47662	7.61E-07	0.000531	5.649782	PD
cg09217797	0.478849	-4.16048	5.476305	7.62E-07	0.000531	5.641209	PD
cg03558010	0.320796	3.34544	5.476217	7.62E-07	0.000531	5.621751	PD
cg10922881	-0.24697	2.519345	-5.47529	7.65E-07	0.000532	5.635068	PD
cg19829381	0.305635	2.586694	5.475279	7.65E-07	0.000532	5.601068	PD
cg13274029	0.199914	-3.8992	5.475004	7.66E-07	0.000532	5.611195	PD
cg17035857	-0.31879	4.062589	-5.47468	7.67E-07	0.000532	5.633853	PD
cg10883539	0.298189	3.512983	5.474581	7.67E-07	0.000532	5.620828	PD
cg08093959	-0.2799	4.393991	-5.46735	7.89E-07	0.000547	5.609714	PD
cg18632774	-0.33081	2.51589	-5.46685	7.90E-07	0.000547	5.588996	PD
cg15043847	-0.30042	4.014278	-5.46592	7.93E-07	0.000549	5.600602	PD
cg09142243	-0.3853	4.368137	-5.46401	7.99E-07	0.000552	5.531055	PD
cg06322011	-0.24844	4.02494	-5.46114	8.08E-07	0.000558	5.566019	PD
cg19643314	-0.50533	-5.26562	-5.45846	8.16E-07	0.000563	5.570922	PD
cg06152855	0.400524	4.133485	5.457308	8.20E-07	0.000565	5.562148	PD
cg26089664	-0.25403	3.932299	-5.45623	8.23E-07	0.000567	5.549297	PD
cg14940871	-0.60019	3.7308	-5.45586	8.24E-07	0.000568	5.562232	PD
cg23982595	0.357087	4.417057	5.455486	8.25E-07	0.000568	5.556092	PD
cg16981421	-0.35998	4.519073	-5.45513	8.27E-07	0.000568	5.528666	PD
cg05079405	-0.24627	4.057327	-5.45375	8.31E-07	0.000571	5.561337	PD
cg09503106	-0.20239	3.879864	-5.45116	8.39E-07	0.000576	5.524342	PD
cg14464852	0.452154	4.700894	5.450877	8.40E-07	0.000576	5.526148	PD

cg20558423	-0.9843	4.528339	-5.45077	8.41E-07	0.000576	5.521239	PD
cg11618995	-0.17526	4.341745	-5.44957	8.44E-07	0.000578	5.526134	PD
cg16604299	-0.37008	4.082685	-5.44866	8.47E-07	0.00058	5.474019	PD
cg27452855	-0.17719	4.228647	-5.44842	8.48E-07	0.00058	5.535599	PD
cg02770835	-0.38714	2.608913	-5.44376	8.64E-07	0.00059	5.508612	PD
cg09265417	-0.19698	3.831398	-5.43946	8.78E-07	0.000599	5.50393	PD
cg01569845	-0.2151	3.712206	-5.43913	8.79E-07	0.000599	5.505491	PD
cg13061941	-1.00596	3.585798	-5.43898	8.80E-07	0.000599	5.469243	PD
cg06903658	0.345491	4.610879	5.4383	8.82E-07	0.000601	5.498225	PD
cg08882646	-0.34595	3.173381	-5.43697	8.86E-07	0.000603	5.494963	PD
cg02743284	-0.31002	3.058059	-5.43664	8.87E-07	0.000603	5.501812	PD
cg20800154	-0.26203	3.967818	-5.43548	8.91E-07	0.000606	5.483657	PD
cg18013789	0.249063	4.075293	5.434992	8.93E-07	0.000606	5.49438	PD
cg20312418	0.466538	3.250217	5.432936	9.00E-07	0.000611	5.478446	PD
cg25406865	-0.51474	2.111018	-5.42734	9.20E-07	0.000623	5.426787	PD
cg02662576	-0.32375	3.555735	-5.42633	9.23E-07	0.000625	5.448621	PD
cg15614872	-0.60784	3.845355	-5.42445	9.30E-07	0.000629	5.386174	PD
cg00428927	-0.25847	3.649765	-5.42196	9.39E-07	0.000635	5.444338	PD
cg01226797	0.57195	4.06466	5.420104	9.46E-07	0.000639	5.405147	PD
cg22495298	-0.50182	3.839628	-5.41821	9.53E-07	0.000643	5.405321	PD
cg24428653	-0.24555	3.493343	-5.41821	9.53E-07	0.000643	5.424232	PD
cg21477881	-0.18807	3.634895	-5.41741	9.56E-07	0.000644	5.439806	PD
cg06511138	-0.43614	3.609027	-5.41536	9.63E-07	0.000649	5.328691	PD
cg21353045	0.56013	2.482304	5.41338	9.70E-07	0.000653	5.379366	PD
cg06129092	-0.51932	3.458956	-5.41318	9.71E-07	0.000653	5.389751	PD
cg16144436	1.190417	2.914627	5.41295	9.72E-07	0.000653	5.414593	PD
cg15489593	0.597617	-4.24068	5.412561	9.74E-07	0.000653	5.390145	PD
cg07273313	-0.31009	3.616804	-5.41105	9.79E-07	0.000657	5.405932	PD
cg27533233	-0.36845	4.132189	-5.40879	9.88E-07	0.000662	5.388828	PD
cg07285167	-0.33307	3.089589	-5.40557	1.00E-06	0.00067	5.334745	PD
cg07647179	1.023585	5.094378	5.403543	1.01E-06	0.000674	5.304204	PD

cg18047595	-0.45325	3.869115	-5.4024	1.01E-06	0.000677	5.359001	PD
cg22055730	0.201483	3.362023	5.401596	1.02E-06	0.000678	5.346592	PD
cg25911692	-0.27669	4.005097	-5.40065	1.02E-06	0.00068	5.366048	PD
cg25039336	-0.31059	3.858614	-5.39993	1.02E-06	0.000682	5.328277	PD
cg18772531	-0.49401	3.376625	-5.39858	1.03E-06	0.000685	5.330118	PD
cg20525255	-0.19125	3.130222	-5.39708	1.03E-06	0.000687	5.349445	PD
cg06767126	-0.38557	4.523648	-5.39698	1.03E-06	0.000687	5.337651	PD
cg03714554	-0.17905	4.210494	-5.39693	1.03E-06	0.000687	5.34436	PD
cg03351510	-0.3255	4.120857	-5.39689	1.03E-06	0.000687	5.343911	PD
cg03022891	-1.20797	5.183671	-5.39493	1.04E-06	0.000692	5.331341	PD
cg16890385	-0.43746	2.986894	-5.39361	1.05E-06	0.000695	5.333218	PD
cg09905495	-0.31298	3.973244	-5.3929	1.05E-06	0.000696	5.325508	PD
cg13024511	0.844277	4.254101	5.391119	1.06E-06	0.0007	5.327791	PD
cg16119776	0.664788	3.338924	5.389503	1.06E-06	0.000704	5.331523	PD
cg07653043	0.398534	3.792284	5.387548	1.07E-06	0.000708	5.3111	PD
cg03520265	-0.20568	2.437319	-5.3875	1.07E-06	0.000708	5.312628	PD
cg16692211	-0.56166	4.492295	-5.38529	1.08E-06	0.000714	5.308582	PD
cg14415168	-0.25116	4.033056	-5.38244	1.09E-06	0.000721	5.299766	PD
cg10957001	0.340793	3.170547	5.380842	1.10E-06	0.000725	5.265095	PD
cg09532679	0.513688	-4.39133	5.378696	1.11E-06	0.00073	5.28058	PD
cg02567750	0.526089	2.664328	5.37838	1.11E-06	0.000731	5.279151	PD
cg06342957	-0.19469	3.619508	-5.37539	1.12E-06	0.000738	5.243781	PD
cg24588582	0.654301	4.958703	5.375062	1.12E-06	0.000739	5.290438	PD
cg15171063	-0.24	3.281514	-5.37222	1.14E-06	0.000746	5.271356	PD
cg00986626	0.334391	3.09889	5.370904	1.14E-06	0.000749	5.277589	PD
cg00983705	1.613375	4.075254	5.369527	1.15E-06	0.000753	5.250564	PD
cg05692055	-0.2334	3.728258	-5.36669	1.16E-06	0.00076	5.256041	PD
cg03425785	-0.35715	3.843162	-5.36586	1.16E-06	0.000762	5.231247	PD
cg26883836	-0.20102	4.112892	-5.36579	1.16E-06	0.000762	5.245219	PD
cg08383438	-0.34587	2.516029	-5.36386	1.17E-06	0.000767	5.208462	PD
cg08577424	0.584892	-5.17187	5.363336	1.18E-06	0.000768	5.236243	PD

cg08436396	0.497865	1.991327	5.361697	1.18E-06	0.000772	5.197344	PD
cg03533702	0.23433	-5.72692	5.360234	1.19E-06	0.000776	5.230101	PD
cg21812084	-0.27163	4.443347	-5.35982	1.19E-06	0.000777	5.218509	PD
cg14334652	-0.32963	2.654537	-5.35943	1.19E-06	0.000777	5.231531	PD
cg25780590	-0.26684	3.708443	-5.35941	1.19E-06	0.000777	5.227186	PD
cg18825995	-0.20783	3.925528	-5.35815	1.20E-06	0.00078	5.202045	PD
cg15322185	-0.23218	3.903664	-5.35761	1.20E-06	0.000781	5.228697	PD
cg22402398	0.25326	4.759784	5.357224	1.20E-06	0.000781	5.211356	PD
cg16215362	-0.247	3.1497	-5.35719	1.20E-06	0.000781	5.218043	PD
cg11759860	0.453025	3.055216	5.356914	1.21E-06	0.000781	5.185745	PD
cg17918270	-0.26647	3.430649	-5.3561	1.21E-06	0.000782	5.225363	PD
cg21091189	-0.24972	3.587025	-5.35606	1.21E-06	0.000782	5.184113	PD
cg21189727	-0.43995	-4.62533	-5.35598	1.21E-06	0.000782	5.227993	PD
cg10553102	-0.27064	3.548362	-5.35553	1.21E-06	0.000783	5.203706	PD
cg14583323	-0.23372	3.056871	-5.35318	1.22E-06	0.000789	5.201316	PD
cg21467050	-0.15397	3.322849	-5.35115	1.23E-06	0.000795	5.195919	PD
cg01479031	-0.40499	3.987839	-5.35032	1.24E-06	0.000797	5.199563	PD
cg04196561	-0.34071	3.354325	-5.34829	1.25E-06	0.000802	5.179324	PD
cg22902288	-1.19262	4.792863	-5.34812	1.25E-06	0.000802	5.18157	PD
cg11457367	-0.56267	-4.77667	-5.34741	1.25E-06	0.000804	5.185907	PD
cg08310558	-0.32056	3.873006	-5.34717	1.25E-06	0.000804	5.191873	PD
cg19609351	-0.47155	4.094805	-5.34342	1.27E-06	0.000815	5.1803	PD
cg12050288	-0.22473	3.080119	-5.34144	1.28E-06	0.000821	5.162329	PD
cg14659008	-0.21906	4.434309	-5.33994	1.29E-06	0.000825	5.157913	PD
cg13442839	-0.57895	4.944451	-5.33554	1.31E-06	0.000838	5.124808	PD
cg14698405	-0.22955	2.561621	-5.33299	1.32E-06	0.000846	5.111145	PD
cg20816047	-0.38087	3.990094	-5.33279	1.32E-06	0.000846	5.135044	PD
cg02144242	-0.38749	4.332878	-5.3307	1.33E-06	0.000852	5.118901	PD
cg21110585	0.183788	-4.0512	5.328511	1.34E-06	0.000858	5.101808	PD
cg10067182	-0.23149	3.511571	-5.32704	1.35E-06	0.000862	5.091526	PD
cg18814052	0.266796	3.827604	5.326435	1.35E-06	0.000863	5.098429	PD

cg19471466	-0.51844	-4.12621	-5.32643	1.35E-06	0.000863	5.090409	PD
cg06725997	-0.88062	-7.15408	-5.32499	1.36E-06	0.000867	5.09987	PD
cg02348995	-0.31423	3.591467	-5.32357	1.37E-06	0.000871	5.095408	PD
cg09175792	2.762978	5.263376	5.322887	1.37E-06	0.000873	5.111247	PD
cg04051894	0.643452	3.447211	5.322778	1.37E-06	0.000873	5.098235	PD
cg08884363	-0.22108	1.719751	-5.32248	1.37E-06	0.000873	5.075124	PD
cg03867979	-0.34007	3.857602	-5.32225	1.38E-06	0.000873	5.086535	PD
cg00428468	0.490914	3.325175	5.321134	1.38E-06	0.000876	5.081891	PD
cg18325664	0.475461	4.033824	5.320359	1.39E-06	0.000878	5.085042	PD
cg19764279	-0.35087	3.929102	-5.31567	1.41E-06	0.000893	5.087008	PD
cg13612275	0.280825	3.480377	5.312791	1.43E-06	0.000902	5.034867	PD
cg22511877	-0.34231	3.22602	-5.31276	1.43E-06	0.000902	5.061097	PD
cg07926770	-0.18304	3.909256	-5.3109	1.44E-06	0.000907	5.060919	PD
cg16280215	-0.31457	4.363003	-5.31085	1.44E-06	0.000907	5.054508	PD
cg03485240	-0.46486	4.090863	-5.31009	1.44E-06	0.000909	5.030158	PD
cg14219896	-0.2103	3.239228	-5.30939	1.45E-06	0.00091	5.034391	PD
cg24194674	-0.49776	3.237293	-5.30937	1.45E-06	0.00091	5.038054	PD
cg08096367	-0.36593	3.979508	-5.30887	1.45E-06	0.000911	5.064202	PD
cg00750627	-0.25116	3.882536	-5.30703	1.46E-06	0.000917	5.050054	PD
cg07564598	0.219464	3.97796	5.306535	1.46E-06	0.000918	5.030821	PD
cg08296601	-1.17429	-5.35281	-5.30604	1.46E-06	0.000919	5.007309	PD
cg14747775	0.403133	3.64423	5.304689	1.47E-06	0.000923	5.044424	PD
cg07963816	-0.70807	5.17124	-5.3033	1.48E-06	0.000927	5.042908	PD
cg07795916	-0.34596	2.949402	-5.30032	1.50E-06	0.000937	5.034015	PD
cg13935454	0.351908	3.999662	5.300039	1.50E-06	0.000938	5.014947	PD
cg25962639	-0.49642	5.267329	-5.29911	1.50E-06	0.00094	5.011965	PD
cg14256359	-0.28992	2.379996	-5.29837	1.51E-06	0.000942	5.017677	PD
cg00382572	-0.23619	3.747667	-5.29693	1.52E-06	0.000947	5.003283	PD
cg22557003	0.336432	3.137764	5.296602	1.52E-06	0.000947	4.954954	PD
cg14638554	-0.29598	3.887125	-5.29628	1.52E-06	0.000948	4.986343	PD
cg10435618	-0.44485	3.717316	-5.29447	1.53E-06	0.000953	5.006785	PD

cg08655877	-0.34715	4.207941	-5.29395	1.53E-06	0.000955	5.009307	PD
cg13497401	-0.3862	4.253138	-5.29358	1.54E-06	0.000955	4.994578	PD
cg01401135	-1.06446	4.787921	-5.29232	1.54E-06	0.000959	4.985174	PD
cg08610403	0.287779	4.457283	5.292112	1.54E-06	0.000959	4.986114	PD
cg08646654	-0.24241	3.137376	-5.29135	1.55E-06	0.000961	4.977933	PD
cg08176551	-0.13674	4.23302	-5.29128	1.55E-06	0.000961	4.989631	PD
cg20349024	-0.86121	-4.16361	-5.28829	1.57E-06	0.000971	4.911461	PD
cg17527669	0.235981	3.2408	5.288088	1.57E-06	0.000971	4.973134	PD
cg10776646	0.258134	3.949794	5.28772	1.57E-06	0.000972	4.980363	PD
cg00828126	-0.20435	3.929866	-5.28557	1.58E-06	0.000979	4.967029	PD
cg24777071	-0.26429	2.504182	-5.28343	1.60E-06	0.000986	4.968654	PD
cg08348957	0.610158	3.61861	5.280743	1.61E-06	0.000996	4.94783	PD
cg14157917	-0.26805	4.93981	-5.27752	1.63E-06	0.001007	4.92651	PD
cg17189664	0.494428	3.758172	5.275396	1.65E-06	0.001015	4.913267	PD
cg06228565	-0.66573	3.137599	-5.27487	1.65E-06	0.001016	4.921117	PD
cg09127495	-0.25526	3.97537	-5.27484	1.65E-06	0.001016	4.94407	PD
cg10932815	-0.29541	2.997261	-5.27426	1.65E-06	0.001017	4.933328	PD
cg11022516	0.221106	4.21737	5.273535	1.66E-06	0.001019	4.926148	PD
cg09858583	0.191118	-3.6021	5.271616	1.67E-06	0.001026	4.928812	PD
cg04248502	-0.4055	3.87755	-5.27149	1.67E-06	0.001026	4.904266	PD
cg06660550	-0.13953	4.414883	-5.26916	1.68E-06	0.001034	4.92245	PD
cg23211310	0.305054	4.127493	5.267819	1.69E-06	0.001039	4.898923	PD
cg14345464	-0.43758	4.507651	-5.2669	1.70E-06	0.001041	4.886933	PD
cg23497383	2.255251	-4.39908	5.266821	1.70E-06	0.001041	4.915098	PD
cg10918652	-0.25944	3.75379	-5.26584	1.71E-06	0.001044	4.906341	PD
cg07878625	0.581166	3.68556	5.265689	1.71E-06	0.001044	4.867604	PD
cg10713875	0.888499	3.087076	5.265597	1.71E-06	0.001044	4.870634	PD
cg09500421	-0.28205	4.237702	-5.26333	1.72E-06	0.001052	4.906796	PD
cg18211761	-0.44959	3.376901	-5.26218	1.73E-06	0.001056	4.87371	PD
cg25299580	0.883234	3.680589	5.260542	1.74E-06	0.001061	4.865156	PD
cg18808466	-0.2494	2.621729	-5.26049	1.74E-06	0.001061	4.882883	PD

cg01213447	-0.27144	-2.22544	-5.26005	1.74E-06	0.001062	4.867377	PD
cg26812610	-0.30645	3.950482	-5.25936	1.75E-06	0.001064	4.876563	PD
cg00583096	-0.25254	4.07064	-5.25913	1.75E-06	0.001064	4.805426	PD
cg15998629	-0.3426	2.477114	-5.25793	1.76E-06	0.001068	4.8711	PD
cg18107818	0.542702	-3.72422	5.257678	1.76E-06	0.001069	4.871638	PD
cg22305574	-0.24108	3.151991	-5.25711	1.76E-06	0.00107	4.87318	PD
cg21103668	0.42955	4.143037	5.256786	1.77E-06	0.00107	4.846758	PD
cg24765783	-0.53011	3.817997	-5.25668	1.77E-06	0.00107	4.82744	PD
cg19937331	-0.39885	3.890503	-5.25626	1.77E-06	0.001071	4.869995	PD
cg00561191	-0.41135	4.03002	-5.25609	1.77E-06	0.001071	4.862684	PD
cg26851374	0.233972	4.110316	5.255765	1.77E-06	0.001072	4.843542	PD
cg22398347	-0.26091	3.800042	-5.25461	1.78E-06	0.001075	4.866238	PD
cg26255261	-0.23394	3.38254	-5.25459	1.78E-06	0.001075	4.860788	PD
cg13672736	1.507691	5.465051	5.254421	1.78E-06	0.001075	4.816128	PD
cg20622565	0.386757	2.851788	5.253614	1.79E-06	0.001077	4.864978	PD
cg15007809	0.862665	4.664381	5.25315	1.79E-06	0.001078	4.815009	PD
cg04783450	-0.32949	3.036238	-5.25301	1.79E-06	0.001078	4.819337	PD
cg21639922	0.766427	5.083054	5.252815	1.79E-06	0.001078	4.861217	PD
cg06064861	0.55048	3.335936	5.251937	1.80E-06	0.001081	4.846556	PD
cg10383724	-0.36583	3.676801	-5.25187	1.80E-06	0.001081	4.850522	PD
cg24298514	-0.24269	3.052978	-5.24974	1.81E-06	0.001089	4.82813	PD
cg15515461	-0.5371	3.668868	-5.24902	1.82E-06	0.001091	4.814151	PD
cg00021325	0.739878	-4.89907	5.246946	1.83E-06	0.001099	4.824584	PD
cg08183425	-0.32413	3.192388	-5.24627	1.84E-06	0.001101	4.843018	PD
cg20734984	-0.25393	2.871975	-5.24507	1.85E-06	0.001105	4.833978	PD
cg01360802	0.461683	3.344423	5.243568	1.86E-06	0.001111	4.818203	PD
cg05459970	-0.30438	3.573171	-5.24256	1.86E-06	0.001114	4.824145	PD
cg27018984	0.573417	4.011437	5.240342	1.88E-06	0.001123	4.803947	PD
cg00371891	0.467222	-5.55837	5.240131	1.88E-06	0.001123	4.823265	PD
cg01963035	-0.39045	3.019056	-5.2376	1.90E-06	0.001133	4.776611	PD
cg00656475	0.574471	-5.03709	5.236825	1.91E-06	0.001136	4.754124	PD

cg06680281	-0.21351	4.272552	-5.23591	1.91E-06	0.001139	4.766932	PD
cg02299602	-0.27475	3.823725	-5.23547	1.91E-06	0.00114	4.792824	PD
cg20278291	0.399023	-3.48796	5.234929	1.92E-06	0.001141	4.808547	PD
cg08523887	-0.37287	2.909231	-5.23424	1.92E-06	0.001144	4.778485	PD
cg04970496	-0.31186	3.125639	-5.23269	1.94E-06	0.001149	4.782257	PD
cg25776355	-0.51591	4.558068	-5.23053	1.95E-06	0.001158	4.74709	PD
cg15057747	-0.98329	6.065161	-5.23016	1.95E-06	0.001159	4.757771	PD
cg13429895	-0.2592	3.406522	-5.22807	1.97E-06	0.001167	4.773996	PD
cg07933092	-0.24037	3.856143	-5.22768	1.97E-06	0.001168	4.778917	PD
cg23547048	-0.42356	3.748033	-5.22693	1.98E-06	0.001171	4.758005	PD
cg06838090	-0.28982	3.578594	-5.22599	1.99E-06	0.001174	4.748092	PD
cg23913001	0.240355	3.843083	5.225606	1.99E-06	0.001175	4.760017	PD
cg26477117	-0.87868	3.051183	-5.22504	1.99E-06	0.001177	4.730161	PD
cg03324099	-1.38922	3.65041	-5.22448	2.00E-06	0.001178	4.746817	PD
cg16373426	-0.20456	3.969798	-5.22337	2.00E-06	0.001181	4.749022	PD
cg23336397	-0.38229	4.069245	-5.22334	2.01E-06	0.001181	4.756406	PD
cg16071328	0.310094	3.920908	5.223257	2.01E-06	0.001181	4.744116	PD
cg17853051	-0.27054	3.781575	-5.22204	2.02E-06	0.001186	4.708744	PD
cg10377163	-0.30062	3.697883	-5.22124	2.02E-06	0.001189	4.759158	PD
cg07142068	0.287589	4.228071	5.221061	2.02E-06	0.001189	4.737886	PD
cg18162838	-0.34141	3.359547	-5.21964	2.03E-06	0.001194	4.750747	PD
cg03715204	-0.25602	4.229976	-5.21869	2.04E-06	0.001198	4.734783	PD
cg17324880	0.467036	2.68696	5.218413	2.04E-06	0.001198	4.732785	PD
cg15594302	-0.41048	2.835741	-5.21383	2.08E-06	0.001218	4.720882	PD
cg04337937	-0.38196	3.037009	-5.20922	2.12E-06	0.001239	4.704608	PD
cg09865955	-0.20092	3.771112	-5.20897	2.12E-06	0.001239	4.694548	PD
cg17562787	-0.33667	3.017463	-5.20758	2.13E-06	0.001245	4.666623	PD
cg07462790	0.411014	-4.14087	5.206764	2.14E-06	0.001248	4.706198	PD
cg26531664	-0.44982	3.95902	-5.20658	2.14E-06	0.001248	4.697922	PD
cg16362189	-0.29939	4.261698	-5.20638	2.14E-06	0.001248	4.692125	PD
cg27063720	0.148746	-3.80579	5.205254	2.15E-06	0.001253	4.675292	PD

cg26838315	0.558267	4.180733	5.203918	2.16E-06	0.001258	4.679616	PD
cg20799941	-0.29009	3.773086	-5.20336	2.16E-06	0.00126	4.693491	PD
cg27063932	-0.27677	3.737229	-5.20285	2.17E-06	0.001261	4.68697	PD
cg11475942	1.060618	3.992644	5.202828	2.17E-06	0.001261	4.656368	PD
cg25409708	-0.19529	4.10851	-5.20258	2.17E-06	0.001261	4.683187	PD
cg11140497	-0.22432	3.966006	-5.2011	2.18E-06	0.001267	4.652374	PD
cg24202123	-0.57456	-6.84839	-5.20023	2.19E-06	0.001271	4.668789	PD
cg25307371	-0.69306	-4.48336	-5.19905	2.20E-06	0.001275	4.668705	PD
cg10712974	-0.3841	3.702958	-5.19899	2.20E-06	0.001275	4.664806	PD
cg08750372	-0.37979	3.775798	-5.19638	2.22E-06	0.001287	4.664781	PD
cg12317792	0.32908	2.422731	5.196155	2.22E-06	0.001287	4.652598	PD
cg14458762	-0.32463	2.773709	-5.19134	2.26E-06	0.00131	4.647946	PD
cg09106728	-0.34928	4.246789	-5.19081	2.27E-06	0.001311	4.640841	PD
cg23254196	0.201181	4.022612	5.190127	2.27E-06	0.001314	4.624636	PD
cg08404173	-0.26755	3.042791	-5.18922	2.28E-06	0.001317	4.646163	PD
cg01576720	-0.3363	2.836762	-5.18906	2.28E-06	0.001317	4.626411	PD
cg11318906	0.68091	-5.21315	5.188695	2.29E-06	0.001318	4.598298	PD
cg22754921	-0.22851	4.52109	-5.18846	2.29E-06	0.001319	4.631794	PD
cg16985301	-0.3536	3.955502	-5.18791	2.29E-06	0.00132	4.618212	PD
cg03695081	-0.48263	2.896652	-5.18757	2.30E-06	0.001321	4.629168	PD
cg11396157	0.609295	-6.16686	5.187355	2.30E-06	0.001321	4.641349	PD
cg27550589	0.184301	4.261965	5.185764	2.31E-06	0.001328	4.626197	PD
cg01588652	-0.19604	3.183477	-5.18562	2.31E-06	0.001328	4.622997	PD
cg08360281	-0.54361	-5.46158	-5.18541	2.32E-06	0.001329	4.593118	PD
cg07489732	-0.27763	3.926419	-5.18395	2.33E-06	0.001335	4.629908	PD
cg25193485	-0.5357	4.5812	-5.18261	2.34E-06	0.001341	4.608371	PD
cg03078816	-0.95106	2.809866	-5.18211	2.34E-06	0.001342	4.613712	PD
cg13220290	-0.3501	2.59161	-5.18176	2.35E-06	0.001343	4.584935	PD
cg17584296	0.34605	-6.55995	5.181211	2.35E-06	0.001345	4.62461	PD
cg07962291	-0.32851	3.844946	-5.18012	2.36E-06	0.00135	4.607137	PD
cg27261176	-0.27232	0.762818	-5.17676	2.39E-06	0.001366	4.59493	PD

cg20974139	0.261508	3.632352	5.17259	2.43E-06	0.001387	4.580593	PD
cg05675570	0.34372	3.634911	5.171213	2.44E-06	0.001393	4.485518	PD
cg05598595	-0.29078	2.337027	-5.17118	2.44E-06	0.001393	4.570429	PD
cg03921823	-0.2563	3.643449	-5.17033	2.45E-06	0.001396	4.564088	PD
cg03256578	-0.30043	3.601185	-5.17008	2.45E-06	0.001396	4.557882	PD
cg14389539	-0.39753	3.298185	-5.16974	2.46E-06	0.001397	4.563393	PD
cg25969728	-0.47481	2.201345	-5.16841	2.47E-06	0.001403	4.54939	PD
cg13728529	-1.32275	4.501943	-5.16727	2.48E-06	0.001409	4.548416	PD
cg08477000	-0.28441	3.391139	-5.16511	2.50E-06	0.001419	4.554889	PD
cg16088142	-0.3104	3.676379	-5.16457	2.51E-06	0.001421	4.558457	PD
cg06526693	-0.23456	-6.25256	-5.16383	2.51E-06	0.001424	4.554773	PD
cg07031888	-0.2259	2.66989	-5.16153	2.53E-06	0.001436	4.549843	PD
cg07967630	0.603846	-5.41674	5.160701	2.54E-06	0.001439	4.546629	PD
cg12209881	-0.69679	4.422635	-5.15977	2.55E-06	0.001443	4.513635	PD
cg04951677	-0.30442	3.290516	-5.15939	2.55E-06	0.001444	4.52597	PD
cg01017773	0.388025	-4.56719	5.158804	2.56E-06	0.001447	4.510567	PD
cg22988851	-0.25507	2.668651	-5.15595	2.59E-06	0.001461	4.509101	PD
cg07254667	0.31583	3.485249	5.155341	2.59E-06	0.001464	4.500894	PD
cg15461550	-0.23175	-1.27365	-5.15487	2.60E-06	0.001465	4.510903	PD
cg01571203	-0.2849	4.074846	-5.15481	2.60E-06	0.001465	4.514557	PD
cg09900894	-0.44922	3.888108	-5.15368	2.61E-06	0.00147	4.50697	PD
cg24698780	-0.42848	3.385904	-5.15359	2.61E-06	0.00147	4.516224	PD
cg22051345	-0.17493	4.622654	-5.15324	2.61E-06	0.00147	4.508075	PD
cg03269667	0.381058	4.370616	5.153027	2.62E-06	0.001471	4.51386	PD
cg14920808	-0.71387	2.659936	-5.15236	2.62E-06	0.001473	4.510646	PD
cg25144224	-0.21608	3.025023	-5.15178	2.63E-06	0.001474	4.501857	PD
cg21265295	0.371159	3.644074	5.15175	2.63E-06	0.001474	4.517731	PD
cg26196860	0.340723	3.759535	5.15164	2.63E-06	0.001474	4.507316	PD
cg20550863	0.407088	3.759397	5.150328	2.64E-06	0.001481	4.486983	PD
cg20679096	0.301507	4.463351	5.150088	2.65E-06	0.001481	4.509918	PD
cg19388782	-1.14768	-5.88799	-5.14819	2.67E-06	0.001491	4.477747	PD

cg24140860	-0.18143	3.868208	-5.14491	2.70E-06	0.001508	4.476049	PD
cg02418195	0.521404	3.27525	5.142434	2.72E-06	0.001522	4.478194	PD
cg14100137	-0.62108	3.282837	-5.1368	2.78E-06	0.001553	4.461128	PD
cg18135502	-0.4087	3.190041	-5.13639	2.79E-06	0.001555	4.449957	PD
cg08853575	-0.23335	3.255922	-5.13487	2.80E-06	0.001563	4.446201	PD
cg20416769	-0.38122	2.959225	-5.13456	2.81E-06	0.001563	4.43928	PD
cg16515379	-0.14146	4.197845	-5.13378	2.81E-06	0.001567	4.451308	PD
cg08066306	-0.33836	4.068176	-5.13305	2.82E-06	0.00157	4.455039	PD
cg24416181	0.38552	4.030158	5.132464	2.83E-06	0.001572	4.429615	PD
cg01166145	0.240004	-2.86562	5.132337	2.83E-06	0.001572	4.413517	PD
cg16644803	-0.34095	5.493302	-5.13174	2.84E-06	0.001575	4.438014	PD
cg22790973	-0.25577	-5.05275	-5.12791	2.88E-06	0.001597	4.435361	PD
cg13237094	0.161919	0.442128	5.126724	2.89E-06	0.001603	4.42678	PD
cg19810457	0.269082	4.537039	5.126092	2.90E-06	0.001606	4.435321	PD
cg07476285	-0.28652	3.430755	-5.1242	2.92E-06	0.001616	4.404551	PD
cg26893022	-0.48735	3.34461	-5.12258	2.94E-06	0.001624	4.401718	PD
cg09698441	-0.2938	3.381635	-5.12257	2.94E-06	0.001624	4.386423	PD
cg08161947	0.277559	-2.74133	5.121918	2.94E-06	0.001627	4.407061	PD
cg09945745	0.282066	4.16371	5.121457	2.95E-06	0.001629	4.405324	PD
cg07414422	-0.39935	3.715997	-5.11954	2.97E-06	0.001639	4.362906	PD
cg14373797	-0.41141	3.37255	-5.11854	2.98E-06	0.001644	4.365982	PD
cg02055799	0.33154	-4.53038	5.118473	2.98E-06	0.001644	4.394483	PD
cg22364165	-0.18848	3.92533	-5.11798	2.99E-06	0.001646	4.387886	PD
cg14512563	-0.2553	-4.28726	-5.11607	3.01E-06	0.001657	4.382594	PD
cg16858146	-0.32045	2.991746	-5.11495	3.02E-06	0.001663	4.387679	PD
cg21576187	-0.34347	3.668353	-5.11459	3.03E-06	0.001664	4.385272	PD
cg04868734	-0.26683	3.898628	-5.11365	3.04E-06	0.001669	4.370056	PD
cg06853506	-0.49246	4.989931	-5.11336	3.04E-06	0.001669	4.365781	PD
cg23203918	-0.53403	3.971571	-5.11147	3.06E-06	0.00168	4.385172	PD
cg10633103	-0.50446	3.356918	-5.1102	3.08E-06	0.001687	4.352302	PD
cg11470063	-0.21077	2.614906	-5.10985	3.08E-06	0.001688	4.359297	PD

cg09901532	-0.26686	3.921285	-5.10915	3.09E-06	0.001692	4.374416	PD
cg20922839	-0.23041	3.638445	-5.1086	3.09E-06	0.001694	4.353414	PD
cg26467717	0.272085	3.627354	5.10644	3.12E-06	0.001707	4.346967	PD
cg26883190	-0.22944	2.460119	-5.10623	3.12E-06	0.001707	4.353992	PD
cg14050838	0.310175	-4.19012	5.103664	3.15E-06	0.001722	4.326438	PD
cg18077636	-0.39955	4.084301	-5.1032	3.16E-06	0.001724	4.335021	PD
cg00353675	-0.24898	3.618002	-5.10227	3.17E-06	0.001729	4.345667	PD
cg23980070	-0.19099	3.361828	-5.10074	3.19E-06	0.001738	4.328744	PD
cg09895065	-0.18295	2.796201	-5.1002	3.19E-06	0.00174	4.316538	PD
cg12973586	-1.41206	4.168231	-5.09963	3.20E-06	0.001743	4.318052	PD
cg18902820	-0.20883	3.597635	-5.09893	3.21E-06	0.001747	4.331576	PD
cg11426344	0.38527	3.821885	5.098382	3.22E-06	0.001749	4.324939	PD
cg14433374	-0.30096	3.539387	-5.09536	3.25E-06	0.001768	4.320744	PD
cg26659797	-0.43627	3.493197	-5.09501	3.26E-06	0.001769	4.292617	PD
cg27191554	-0.21697	5.489047	-5.09483	3.26E-06	0.001769	4.316263	PD
cg19419926	-0.30589	3.113173	-5.09344	3.28E-06	0.001777	4.315152	PD
cg02086996	-0.4155	2.968705	-5.09317	3.28E-06	0.001778	4.29486	PD
cg09361368	-0.24131	3.830242	-5.09266	3.29E-06	0.00178	4.308884	PD
cg18246392	-0.22107	3.198045	-5.09213	3.29E-06	0.001783	4.301883	PD
cg04503500	-0.24815	2.7044	-5.09008	3.32E-06	0.001794	4.291442	PD
cg01728692	0.213474	3.984836	5.09006	3.32E-06	0.001794	4.28952	PD
cg14152141	-0.62656	3.415319	-5.08931	3.33E-06	0.001798	4.227977	PD
cg00041829	-0.38278	4.174645	-5.08815	3.34E-06	0.001805	4.28165	PD
cg23012654	-0.41392	3.19475	-5.08787	3.35E-06	0.001806	4.269472	PD
cg02844820	-0.37826	3.535088	-5.08767	3.35E-06	0.001806	4.2401	PD
cg09174817	-0.74001	2.54478	-5.0871	3.36E-06	0.001809	4.229983	PD
cg17976697	-0.55876	3.380095	-5.08497	3.38E-06	0.001822	4.255141	PD
cg13340813	-0.15904	2.842328	-5.08465	3.39E-06	0.001823	4.276374	PD
cg23761504	0.264917	2.454644	5.083658	3.40E-06	0.001828	4.280871	PD
cg25654556	-0.26314	3.328527	-5.08354	3.40E-06	0.001828	4.271932	PD
cg02885046	-0.21414	2.753749	-5.08338	3.40E-06	0.001828	4.264431	PD

cg21996217	-0.2344	2.295249	-5.08318	3.40E-06	0.001828	4.265176	PD
cg27151480	-0.41251	4.08387	-5.08289	3.41E-06	0.001829	4.260084	PD
cg00850470	-0.36995	4.173575	-5.08249	3.41E-06	0.001831	4.271745	PD
cg18765479	-0.20343	3.726978	-5.08104	3.43E-06	0.00184	4.25697	PD
cg25198316	-0.50378	3.450489	-5.0805	3.44E-06	0.001842	4.234416	PD
cg00913191	-0.23658	3.47999	-5.07949	3.45E-06	0.001847	4.255644	PD
cg18971282	-0.34569	3.743861	-5.07942	3.45E-06	0.001847	4.203401	PD
cg08197665	0.572079	3.130239	5.078283	3.47E-06	0.001854	4.247616	PD
cg09576532	-0.48955	-5.14267	-5.07813	3.47E-06	0.001854	4.254289	PD
cg01091261	-0.22079	3.314857	-5.07649	3.49E-06	0.001863	4.253427	PD
cg00961450	0.14704	-0.909	5.076382	3.49E-06	0.001863	4.246289	PD
cg10033663	-0.2836	3.476367	-5.07631	3.49E-06	0.001863	4.242162	PD
cg14582642	0.366385	-3.58067	5.075208	3.51E-06	0.00187	4.239381	PD
cg05567734	-0.43858	2.835715	-5.07169	3.56E-06	0.001894	4.225109	PD
cg16127555	-0.52237	3.629646	-5.0707	3.57E-06	0.001899	4.198402	PD
cg12549493	-0.22089	3.10017	-5.0689	3.59E-06	0.001911	4.227081	PD
cg20310149	-0.41897	4.311797	-5.06876	3.59E-06	0.001911	4.230413	PD
cg11101081	-0.28621	4.604176	-5.06805	3.60E-06	0.001915	4.209033	PD
cg27126508	-0.97577	3.458945	-5.06763	3.61E-06	0.001916	4.183417	PD
cg05238740	-0.27614	4.058852	-5.06753	3.61E-06	0.001916	4.21558	PD
cg01763875	2.068681	3.513661	5.0668	3.62E-06	0.00192	4.20682	PD
cg20877443	0.189333	4.341473	5.063963	3.66E-06	0.001939	4.200518	PD
cg12307698	-1.63306	-5.86855	-5.06369	3.66E-06	0.00194	4.208378	PD
cg06640728	-0.23022	3.347381	-5.06228	3.68E-06	0.001949	4.206088	PD
cg20245361	0.506606	3.566275	5.061858	3.69E-06	0.001951	4.198777	PD
cg25726128	-0.2489	2.762384	-5.0609	3.70E-06	0.001957	4.191785	PD
cg08581878	0.230197	4.253782	5.059307	3.72E-06	0.001968	4.200292	PD
cg23652363	0.424456	4.397412	5.059027	3.73E-06	0.001968	4.204671	PD
cg17516160	1.259853	-6.34839	5.057334	3.75E-06	0.00198	4.190422	PD
cg20237815	-0.23285	3.852186	-5.05682	3.76E-06	0.001981	4.182211	PD
cg11247402	-0.98158	4.415999	-5.05682	3.76E-06	0.001981	4.170228	PD

cg13440864	-0.41493	2.59266	-5.05607	3.77E-06	0.001985	4.180295	PD
cg02811312	0.265439	-7.45808	5.055884	3.77E-06	0.001986	4.18987	PD
cg22460896	-0.2082	-1.49666	-5.05551	3.78E-06	0.001987	4.172502	PD
cg10047572	-0.1435	0.469841	-5.05522	3.78E-06	0.001987	4.181899	PD
cg09050490	0.390399	4.531578	5.055197	3.78E-06	0.001987	4.186147	PD
cg26671605	0.228942	4.124072	5.054148	3.80E-06	0.001992	4.187077	PD
cg10924085	-0.41205	4.277075	-5.05386	3.80E-06	0.001992	4.1784	PD
cg23570146	0.258304	3.878645	5.053832	3.80E-06	0.001992	4.186498	PD
cg26808328	-0.30797	3.917427	-5.05377	3.80E-06	0.001992	4.174351	PD
cg10982913	0.8551	-3.40582	5.053695	3.80E-06	0.001992	4.14789	PD
cg11115976	0.450007	3.862029	5.053371	3.81E-06	0.001993	4.169112	PD
cg22130585	0.494725	-3.3398	5.05313	3.81E-06	0.001994	4.176059	PD
cg23372527	0.476626	3.627202	5.052117	3.83E-06	0.002	4.164175	PD
cg16465598	0.519197	4.077508	5.051944	3.83E-06	0.002	4.146578	PD
cg21464891	-0.39325	4.197834	-5.05181	3.83E-06	0.002	4.162282	PD
cg25518362	0.44637	3.162103	5.050125	3.85E-06	0.002011	4.166029	PD
cg01910953	-0.93431	3.550777	-5.04991	3.86E-06	0.002012	4.153167	PD
cg04604215	0.324369	3.140213	5.048161	3.88E-06	0.002024	4.141934	PD
cg05728588	-0.33683	3.996719	-5.04712	3.90E-06	0.00203	4.153714	PD
cg16957163	-0.25653	3.330548	-5.04631	3.91E-06	0.002035	4.14637	PD
cg13117369	-0.13288	4.954194	-5.04413	3.94E-06	0.00205	4.133054	PD
cg13828899	-0.51868	3.962602	-5.044	3.94E-06	0.00205	4.134781	PD
cg12140527	-0.24299	3.538514	-5.04373	3.95E-06	0.002051	4.143495	PD
cg10731798	-0.34953	3.44808	-5.04346	3.95E-06	0.002052	4.119863	PD
cg10108617	-0.19038	4.615316	-5.04195	3.97E-06	0.002062	4.133514	PD
cg15547657	-0.28734	2.443979	-5.04126	3.99E-06	0.002067	4.139399	PD
cg09777172	0.716917	3.408567	5.038258	4.03E-06	0.002089	4.073128	PD
cg23319854	-0.25709	4.13159	-5.03779	4.04E-06	0.002091	4.126187	PD
cg02562299	-0.46188	4.594745	-5.03727	4.05E-06	0.002094	4.110668	PD
cg18868178	0.521225	-4.04438	5.03571	4.07E-06	0.002105	4.110278	PD
cg19505184	-0.24638	3.581947	-5.03374	4.10E-06	0.002119	4.102758	PD

cg11091004	0.44901	-5.31014	5.032811	4.11E-06	0.002125	4.098427	PD
cg24908179	0.288823	4.529934	5.031328	4.14E-06	0.002136	4.095661	PD
cg27517502	-0.30693	3.720164	-5.03035	4.15E-06	0.002142	4.092856	PD
cg00253313	0.396883	3.338176	5.030232	4.15E-06	0.002142	4.072769	PD
cg00727225	0.491283	2.981551	5.030043	4.16E-06	0.002142	4.101605	PD
cg19976628	0.588681	3.685426	5.029822	4.16E-06	0.002143	3.993271	PD
cg02207084	-0.36479	4.726868	-5.02964	4.16E-06	0.002143	4.085295	PD
cg02293425	-0.2838	3.592859	-5.02894	4.17E-06	0.002147	4.086777	PD
cg23335398	-0.16519	4.401507	-5.02869	4.18E-06	0.002148	4.0842	PD
cg18834544	-0.22959	3.978972	-5.02789	4.19E-06	0.002153	4.071705	PD
cg13499708	-0.25201	3.039017	-5.02738	4.20E-06	0.002156	4.092028	PD
cg08335008	-0.41638	4.197659	-5.02698	4.20E-06	0.002158	4.082701	PD
cg20226743	-0.35779	3.679872	-5.02666	4.21E-06	0.002159	4.080382	PD
cg18757828	-1.49848	3.951059	-5.02615	4.22E-06	0.002162	4.061243	PD
cg10059542	-0.24397	3.12229	-5.02567	4.22E-06	0.002164	4.081426	PD
cg25970452	0.314538	4.250936	5.024804	4.24E-06	0.002169	4.078416	PD
cg04891815	-0.19752	3.150953	-5.0248	4.24E-06	0.002169	4.078903	PD
cg26639401	-0.29857	3.221779	-5.02382	4.25E-06	0.002175	4.073946	PD
cg12050475	-0.46706	4.477529	-5.02231	4.28E-06	0.002186	4.071344	PD
cg16624231	-0.39546	4.102664	-5.02196	4.28E-06	0.002188	4.062229	PD
cg03101580	-0.11723	0.601103	-5.02061	4.31E-06	0.002198	4.04787	PD
cg14623093	0.358764	-4.66941	5.020321	4.31E-06	0.002199	4.0428	PD
cg13289993	-0.23964	2.246731	-5.01863	4.34E-06	0.002211	4.04972	PD
cg04997580	-0.20293	3.933214	-5.01484	4.40E-06	0.002242	4.040125	PD
cg06419846	-0.35879	2.805884	-5.01337	4.42E-06	0.002252	4.037842	PD
cg16237847	-0.4471	3.939484	-5.01326	4.43E-06	0.002252	4.030857	PD
cg14498749	-0.39827	3.818118	-5.01291	4.43E-06	0.002254	4.037853	PD
cg22496001	-0.17831	4.389596	-5.01234	4.44E-06	0.002257	4.040538	PD
cg13548081	-0.28414	3.740429	-5.0108	4.47E-06	0.002269	3.995236	PD
cg26302238	-0.46517	3.477342	-5.00964	4.49E-06	0.002277	4.024257	PD
cg00376941	-0.19192	3.996791	-5.00906	4.50E-06	0.002281	4.018262	PD

cg17031489	-0.21464	3.787259	-5.00856	4.50E-06	0.002283	4.016917	PD
cg01808640	-0.23443	3.866048	-5.00855	4.50E-06	0.002283	4.018685	PD
cg02354225	0.539107	3.345514	5.006591	4.54E-06	0.002298	3.938554	PD
cg11035303	-1.45479	-5.49519	-5.00609	4.55E-06	0.0023	3.992596	PD
cg16912836	-0.22242	4.269504	-5.00595	4.55E-06	0.0023	4.007894	PD
cg17694494	-0.28012	3.446465	-5.00589	4.55E-06	0.0023	4.005967	PD
cg20619667	-0.22299	3.764084	-5.00515	4.56E-06	0.002305	4.006714	PD
cg08551408	0.681857	2.041018	5.004187	4.58E-06	0.002311	3.999998	PD
cg09417503	-0.17979	1.256647	-5.00407	4.58E-06	0.002311	3.993634	PD
cg27599856	-0.26841	3.721868	-5.00097	4.63E-06	0.002337	4.001038	PD
cg15986644	-0.20089	5.978619	-5.00079	4.64E-06	0.002337	3.99473	PD
cg02157482	-0.20794	3.553187	-5.00037	4.64E-06	0.002339	3.993407	PD
cg17290159	0.125834	-4.60315	4.999281	4.66E-06	0.002348	3.975097	PD
cg09205538	-0.13243	4.481751	-4.99908	4.67E-06	0.002348	3.994458	PD
cg01365452	-0.15547	4.387963	-4.9985	4.68E-06	0.002352	3.994176	PD
cg10509179	-0.59977	2.459216	-4.99748	4.69E-06	0.002359	3.970713	PD
cg06128054	0.396179	3.955027	4.997104	4.70E-06	0.002361	3.979677	PD
cg06090322	0.436484	3.243484	4.997001	4.70E-06	0.002361	3.987821	PD
cg00580789	-0.22416	3.264999	-4.99677	4.71E-06	0.002361	3.970039	PD
cg06619301	-0.28552	3.665005	-4.99576	4.72E-06	0.002369	3.979411	PD
cg18375960	-0.30184	4.181964	-4.9948	4.74E-06	0.002376	3.969833	PD
cg10920905	0.336769	3.808365	4.993729	4.76E-06	0.002384	3.963984	PD
cg16387532	0.275149	-3.27231	4.992858	4.78E-06	0.00239	3.971167	PD
cg00060975	-0.6011	-4.52909	-4.99233	4.79E-06	0.002394	3.967422	PD
cg22435982	-0.20361	-4.77167	-4.99214	4.79E-06	0.002394	3.959561	PD
cg04498082	1.051026	-7.03221	4.990186	4.82E-06	0.00241	3.958673	PD
cg13205059	-0.29592	3.674116	-4.9898	4.83E-06	0.002412	3.957593	PD
cg15572325	-0.34415	2.001013	-4.98873	4.85E-06	0.00242	3.930935	PD
cg09223563	0.350494	3.909898	4.988181	4.86E-06	0.002424	3.950546	PD
cg01075305	-0.28893	4.382959	-4.98742	4.87E-06	0.002429	3.953122	PD
cg10104451	0.838628	-3.68986	4.987095	4.88E-06	0.002431	3.948785	PD

cg25301364	-0.16747	4.041294	-4.9868	4.89E-06	0.002432	3.922064	PD
cg00880674	-0.35787	3.417744	-4.98643	4.89E-06	0.002434	3.948784	PD
cg20370507	-0.23933	2.313678	-4.98626	4.90E-06	0.002434	3.951437	PD
cg22610647	0.165044	-3.80884	4.986125	4.90E-06	0.002434	3.937274	PD
cg06713968	0.281056	4.547869	4.985887	4.90E-06	0.002434	3.938001	PD
cg06307212	-0.29501	2.950779	-4.98585	4.90E-06	0.002434	3.943377	PD
cg07742368	0.206085	0.852049	4.985521	4.91E-06	0.002435	3.946132	PD
cg16424195	0.310959	4.476127	4.985082	4.92E-06	0.002438	3.933744	PD
cg09638005	-0.19041	3.020861	-4.98423	4.93E-06	0.002444	3.914842	PD
cg26785303	-0.42772	2.355651	-4.97915	5.03E-06	0.002489	3.911816	PD
cg26615813	-0.38709	-5.9053	-4.97831	5.04E-06	0.002495	3.914357	PD
cg25467233	-0.3049	3.724074	-4.97821	5.04E-06	0.002495	3.901333	PD
cg13415069	-0.32225	4.119108	-4.97707	5.07E-06	0.002504	3.910303	PD
cg08177522	-0.25851	2.833669	-4.97488	5.11E-06	0.002523	3.911635	PD
cg09748403	-0.23348	3.247493	-4.97392	5.13E-06	0.002531	3.885903	PD
cg09222863	0.559211	-3.65761	4.973658	5.13E-06	0.002532	3.908687	PD
cg18521725	-0.20688	4.132624	-4.97273	5.15E-06	0.002539	3.888206	PD
cg04204558	-0.19956	4.322544	-4.97187	5.17E-06	0.002545	3.898496	PD
cg11500779	-0.23981	3.787991	-4.97173	5.17E-06	0.002545	3.897798	PD
cg14076827	-0.21642	3.194471	-4.97163	5.17E-06	0.002545	3.891607	PD
cg14673198	-0.24909	3.929477	-4.97076	5.19E-06	0.002552	3.899432	PD
cg25009451	-0.25463	-4.55145	-4.97037	5.19E-06	0.002554	3.891298	PD
cg07851262	0.353979	3.528979	4.968013	5.24E-06	0.002575	3.88933	PD
cg14970802	-0.22492	3.08374	-4.96618	5.28E-06	0.002591	3.872926	PD
cg06312066	-0.16051	4.633561	-4.96475	5.30E-06	0.002604	3.875659	PD
cg06196926	-0.18576	3.465857	-4.96353	5.33E-06	0.002614	3.861232	PD
cg00714256	0.211914	3.965693	4.962035	5.36E-06	0.002627	3.873062	PD
cg04091505	0.298296	4.444213	4.961255	5.37E-06	0.002633	3.861805	PD
cg01088726	-0.19968	3.403919	-4.9598	5.40E-06	0.002646	3.851734	PD
cg13524748	-0.36515	2.042824	-4.95894	5.42E-06	0.002652	3.848393	PD
cg15133049	0.218111	-5.49893	4.958848	5.42E-06	0.002652	3.860777	PD

cg02275213	-0.28438	2.436327	-4.95869	5.43E-06	0.002652	3.851612	PD
cg03978067	-0.59881	3.648208	-4.95759	5.45E-06	0.002661	3.775684	PD
cg20139683	1.681812	3.459444	4.95753	5.45E-06	0.002661	3.83092	PD
cg24962513	0.287823	4.373263	4.956665	5.47E-06	0.002668	3.84609	PD
cg13422185	0.346704	4.042535	4.956367	5.47E-06	0.002669	3.842437	PD
cg06481168	-0.91816	-4.42015	-4.95579	5.48E-06	0.002673	3.831505	PD
cg27395839	-0.56224	3.334048	-4.95413	5.52E-06	0.002689	3.83829	PD
cg04529860	0.722935	-5.32288	4.953373	5.53E-06	0.002695	3.818158	PD
cg09169014	-0.22094	2.029873	-4.95286	5.54E-06	0.002698	3.834011	PD
cg03017753	-0.14638	4.22318	-4.95134	5.58E-06	0.002712	3.828569	PD
cg13521643	0.740281	3.27385	4.950584	5.59E-06	0.002718	3.81054	PD
cg10193870	-0.25909	3.244472	-4.95031	5.60E-06	0.002719	3.821215	PD
cg06304518	-0.28169	4.28293	-4.9478	5.65E-06	0.002743	3.817628	PD
cg19891517	-0.27587	3.129544	-4.94755	5.66E-06	0.002744	3.819838	PD
cg01802043	-0.26763	4.09807	-4.94725	5.66E-06	0.002746	3.823394	PD
cg24603152	0.467775	-5.49657	4.946145	5.68E-06	0.002755	3.80138	PD
cg11747586	-0.28423	4.090893	-4.94495	5.71E-06	0.002766	3.8122	PD
cg25513610	-0.3541	3.640024	-4.943	5.75E-06	0.002785	3.803195	PD
cg12531919	0.185534	3.912886	4.941707	5.78E-06	0.002794	3.79544	PD
cg12415687	-0.25438	3.608379	-4.94168	5.78E-06	0.002794	3.787991	PD
cg16045677	-0.35594	4.332465	-4.94158	5.78E-06	0.002794	3.799767	PD
cg01957786	-0.30821	4.044486	-4.94091	5.80E-06	0.0028	3.793219	PD
cg15808266	-0.31636	3.873123	-4.93809	5.86E-06	0.002828	3.790526	PD
cg16704061	0.253776	3.959024	4.937711	5.87E-06	0.00283	3.779366	PD
cg00261325	-0.20485	4.434163	-4.93673	5.89E-06	0.002838	3.784498	PD
cg12169771	-0.1927	4.539242	-4.93665	5.89E-06	0.002838	3.781658	PD
cg25718322	-0.23981	3.979101	-4.93433	5.94E-06	0.002861	3.769963	PD
cg22166835	0.286801	4.573973	4.934175	5.94E-06	0.002861	3.75317	PD
cg20484215	-0.34191	2.925553	-4.93387	5.95E-06	0.002863	3.768106	PD
cg03753284	0.727301	-4.22807	4.932776	5.97E-06	0.002873	3.754125	PD
cg06447952	0.195311	3.823997	4.932593	5.98E-06	0.002873	3.770125	PD

cg17357341	-0.31269	3.499588	-4.9322	5.99E-06	0.002875	3.767784	PD
cg26306683	-0.29977	-5.33693	-4.9321	5.99E-06	0.002875	3.771954	PD
cg15209489	-0.18459	3.940792	-4.92968	6.04E-06	0.002899	3.74447	PD
cg09525623	-0.28381	2.077874	-4.92955	6.05E-06	0.002899	3.746676	PD
cg19850149	-0.50801	4.568563	-4.92924	6.05E-06	0.0029	3.731895	PD
cg01120822	-0.26407	3.873042	-4.92918	6.05E-06	0.0029	3.758841	PD
cg04632468	0.267712	3.874297	4.928837	6.06E-06	0.002902	3.7536	PD
cg17358054	0.270879	2.613063	4.92872	6.07E-06	0.002902	3.708687	PD
cg16742775	-0.27433	4.081402	-4.92852	6.07E-06	0.002902	3.74612	PD
cg19146456	0.432545	-4.7443	4.928164	6.08E-06	0.002904	3.74308	PD
cg01991743	-0.37424	3.976679	-4.92737	6.10E-06	0.00291	3.707088	PD
cg13931996	-0.45932	4.97985	-4.92726	6.10E-06	0.00291	3.738246	PD
cg13521769	-0.3425	-4.50577	-4.92716	6.10E-06	0.00291	3.748611	PD
cg17143606	0.338572	4.158293	4.926109	6.12E-06	0.00292	3.740239	PD
cg07889482	-0.28801	4.357013	-4.9258	6.13E-06	0.002922	3.721179	PD
cg18754910	-0.24324	3.056224	-4.92529	6.14E-06	0.002926	3.740158	PD
cg13373361	-0.1867	4.608309	-4.92242	6.21E-06	0.002952	3.73829	PD
cg12848345	0.370171	-3.53122	4.922378	6.21E-06	0.002952	3.726853	PD
cg23168520	-0.47436	4.338985	-4.92236	6.21E-06	0.002952	3.708764	PD
cg13376960	0.631446	3.486476	4.921042	6.24E-06	0.002965	3.708993	PD
cg18814808	-0.2452	3.261837	-4.92095	6.24E-06	0.002965	3.726575	PD
cg15321195	0.332457	-4.40445	4.919756	6.27E-06	0.002975	3.704293	PD
cg07042985	0.329506	4.18448	4.91973	6.27E-06	0.002975	3.709157	PD
cg02124734	0.303152	4.151153	4.919269	6.28E-06	0.002978	3.70995	PD
cg08275953	-0.38091	3.405298	-4.91917	6.28E-06	0.002978	3.723953	PD
cg13945667	-0.29872	3.164954	-4.91674	6.34E-06	0.003003	3.705663	PD
cg12434322	0.306271	4.079298	4.916464	6.35E-06	0.003004	3.719528	PD
cg04833646	-0.59119	-5.19855	-4.91612	6.36E-06	0.003006	3.713038	PD
cg05821000	0.284163	4.34058	4.914505	6.39E-06	0.003023	3.703626	PD
cg22753522	-0.17308	4.099418	-4.91182	6.46E-06	0.003051	3.698527	PD
cg24500294	-0.23275	2.125373	-4.91155	6.46E-06	0.003053	3.695053	PD

cg01494994	-0.26469	2.483031	-4.91099	6.48E-06	0.003057	3.695671	PD
cg24385954	-0.26643	3.579814	-4.91081	6.48E-06	0.003058	3.692113	PD
cg14979300	-0.23665	2.322866	-4.90759	6.56E-06	0.003093	3.673611	PD
cg20849032	-0.18137	3.751723	-4.90709	6.57E-06	0.003097	3.677936	PD
cg21374438	-0.29992	3.585108	-4.90643	6.59E-06	0.003103	3.677194	PD
cg24060660	-0.27554	3.10782	-4.90603	6.60E-06	0.003106	3.668026	PD
cg14976342	-0.31099	2.278152	-4.90523	6.62E-06	0.003113	3.663803	PD
cg11407942	-0.18169	3.492262	-4.90446	6.64E-06	0.00312	3.662663	PD
cg26667508	-0.29619	3.144079	-4.90404	6.65E-06	0.003122	3.666146	PD
cg13011331	-0.40519	3.099466	-4.90403	6.65E-06	0.003122	3.657977	PD
cg05225404	-0.28515	4.02234	-4.90374	6.65E-06	0.003123	3.675816	PD
cg22099896	1.604398	-4.61064	4.903452	6.66E-06	0.003124	3.663401	PD
cg13215579	-0.21641	3.724398	-4.90338	6.66E-06	0.003124	3.674747	PD
cg19883066	-0.25775	2.264955	-4.90299	6.67E-06	0.003127	3.652076	PD
cg18959327	0.342867	4.336117	4.898916	6.77E-06	0.003173	3.652875	PD
cg11715742	-0.29613	3.488117	-4.89704	6.82E-06	0.003193	3.644876	PD
cg10411146	0.331354	6.066484	4.896933	6.82E-06	0.003193	3.639595	PD
cg03020379	-0.74466	3.006949	-4.89655	6.83E-06	0.003195	3.569018	PD
cg10545467	0.411164	3.02896	4.896382	6.84E-06	0.003196	3.640885	PD
cg07356283	-0.19196	3.66553	-4.8961	6.85E-06	0.003197	3.636043	PD
cg01417401	0.57916	3.916421	4.894387	6.89E-06	0.003212	3.622711	PD
cg13433259	0.646282	3.760091	4.894339	6.89E-06	0.003212	3.63545	PD
cg19857758	-0.16528	4.312035	-4.89433	6.89E-06	0.003212	3.637476	PD
cg27244385	-0.42391	3.198267	-4.89423	6.89E-06	0.003212	3.632775	PD
cg02502145	1.370756	-3.74067	4.893007	6.92E-06	0.003225	3.633925	PD
cg23855260	-0.30776	3.937373	-4.89287	6.93E-06	0.003225	3.638768	PD
cg08917821	-0.24705	3.620478	-4.89275	6.93E-06	0.003225	3.616842	PD
cg22191657	-0.40544	-5.79831	-4.8901	7.00E-06	0.003255	3.630202	PD
cg24545937	0.434953	-4.74442	4.888434	7.04E-06	0.003273	3.582243	PD
cg26232282	0.308447	4.440724	4.887995	7.05E-06	0.003277	3.619734	PD
cg23029573	-0.20768	2.064562	-4.8871	7.08E-06	0.003286	3.614928	PD

cg18707575	-0.1938	3.107127	-4.88662	7.09E-06	0.00329	3.617722	PD
cg00090697	-0.40108	3.348274	-4.88576	7.11E-06	0.003297	3.608935	PD
cg11701091	0.603278	3.854584	4.885736	7.11E-06	0.003297	3.605926	PD
cg05851866	0.24394	4.113632	4.885293	7.13E-06	0.0033	3.604584	PD
cg11023174	-0.20187	3.564752	-4.88457	7.14E-06	0.003307	3.599101	PD
cg08084441	0.626374	4.108229	4.884115	7.16E-06	0.003311	3.590876	PD
cg09312814	-0.2033	3.292488	-4.88255	7.20E-06	0.003329	3.600927	PD
cg19167034	-0.16888	3.625782	-4.88149	7.23E-06	0.00334	3.587405	PD
cg22399555	-0.3504	2.156671	-4.88114	7.24E-06	0.003342	3.587968	PD
cg22068589	-1.38514	3.648413	-4.87952	7.28E-06	0.003361	3.580192	PD
cg10643928	-0.29309	5.701141	-4.87929	7.29E-06	0.003362	3.580638	PD
cg15926677	0.210722	-0.58446	4.879133	7.29E-06	0.003362	3.586518	PD
cg19800155	-0.16521	3.257423	-4.87896	7.29E-06	0.003362	3.577892	PD
cg18394648	0.445278	3.453095	4.87845	7.31E-06	0.003367	3.529354	PD
cg05002712	0.342747	3.589471	4.878012	7.32E-06	0.00337	3.576626	PD
cg14984210	0.283251	4.249016	4.876578	7.36E-06	0.003386	3.58584	PD
cg00362657	-0.2072	-0.21913	-4.87645	7.36E-06	0.003386	3.563763	PD
cg03649521	-0.29269	3.929752	-4.87634	7.37E-06	0.003386	3.568062	PD
cg23685586	-0.18231	3.715999	-4.87608	7.37E-06	0.003387	3.57989	PD
cg10493994	-0.23665	-3.64754	-4.87469	7.41E-06	0.003403	3.577371	PD
cg18305853	0.773298	5.356778	4.873622	7.44E-06	0.003414	3.574112	PD
cg20153461	0.18636	3.400963	4.873254	7.45E-06	0.003416	3.569629	PD
cg08271298	0.367813	4.078309	4.873205	7.45E-06	0.003416	3.561994	PD
cg17266357	0.288791	3.568753	4.873017	7.46E-06	0.003416	3.552982	PD
cg11942206	0.309256	3.787809	4.872002	7.48E-06	0.003427	3.554324	PD
cg06163789	-0.29343	3.06307	-4.87185	7.49E-06	0.003427	3.560086	PD
cg09634209	-0.45615	2.927758	-4.87159	7.50E-06	0.003427	3.547255	PD
cg25730401	-0.33203	3.722259	-4.87158	7.50E-06	0.003427	3.562375	PD
cg14787155	0.390477	-2.39029	4.869167	7.56E-06	0.003456	3.550183	PD
cg06157924	-1.92633	5.176864	-4.86646	7.64E-06	0.003489	3.547766	PD
cg16675581	0.715043	2.845596	4.865363	7.67E-06	0.003501	3.508376	PD

cg05721771	-0.24179	4.0437	-4.86498	7.68E-06	0.003504	3.531048	PD
cg10217657	-0.26406	3.489431	-4.86444	7.70E-06	0.003509	3.539083	PD
cg14160787	0.334836	3.676441	4.863943	7.71E-06	0.003514	3.53656	PD
cg03756645	-0.22735	2.49725	-4.86374	7.72E-06	0.003515	3.532771	PD
cg00851761	-0.221	3.612747	-4.86249	7.75E-06	0.003529	3.531081	PD
cg00268989	-0.17762	3.583378	-4.86181	7.77E-06	0.003533	3.533467	PD
cg06599402	0.289705	4.488799	4.861792	7.77E-06	0.003533	3.531591	PD
cg14631519	-0.26621	3.668712	-4.86179	7.77E-06	0.003533	3.531342	PD
cg00548971	-0.24336	3.991743	-4.86106	7.79E-06	0.00354	3.527621	PD
cg04105376	-0.60377	3.565952	-4.85755	7.90E-06	0.003585	3.475547	PD
cg21542003	0.371091	3.622302	4.856909	7.91E-06	0.003591	3.508309	PD
cg13390441	0.210613	-0.91351	4.856782	7.92E-06	0.003591	3.504959	PD
cg15707176	-0.91023	3.354382	-4.85629	7.93E-06	0.003595	3.453941	PD
cg16808927	-0.57985	-4.75939	-4.85561	7.95E-06	0.003601	3.507405	PD
cg04963199	0.509237	2.327102	4.855525	7.95E-06	0.003601	3.501931	PD
cg07287078	0.254715	3.623237	4.855461	7.96E-06	0.003601	3.499556	PD
cg02494958	-0.25342	3.09745	-4.85496	7.97E-06	0.003604	3.507198	PD
cg01714809	0.621396	-4.66557	4.854913	7.97E-06	0.003604	3.505687	PD
cg03054767	-0.43575	3.674626	-4.85409	8.00E-06	0.003613	3.499027	PD
cg07509323	-0.35257	3.249334	-4.85342	8.02E-06	0.00362	3.499775	PD
cg20199549	-0.33963	3.667264	-4.85331	8.02E-06	0.00362	3.499936	PD
cg13411867	0.233876	4.604436	4.8528	8.04E-06	0.003625	3.498038	PD
cg16705446	-0.24327	4.520573	-4.85185	8.06E-06	0.003634	3.490158	PD
cg22359548	-0.27684	3.463951	-4.85181	8.06E-06	0.003634	3.489824	PD
cg27539721	-0.1673	4.443742	-4.85169	8.07E-06	0.003634	3.494836	PD
cg22661773	-0.27372	3.836386	-4.85116	8.08E-06	0.003639	3.490348	PD
cg06393245	0.401132	-6.41313	4.849785	8.13E-06	0.003655	3.487252	PD
cg06604497	-0.23547	4.135918	-4.8495	8.13E-06	0.003657	3.483658	PD
cg01470839	-0.20003	3.685266	-4.84906	8.15E-06	0.00366	3.484086	PD
cg08900396	-1.14958	2.928404	-4.84904	8.15E-06	0.00366	3.457643	PD
cg15172804	-0.23465	4.079317	-4.84844	8.17E-06	0.003666	3.480137	PD

cg16678111	-0.22229	3.486853	-4.84782	8.18E-06	0.003672	3.478442	PD	
cg05285291	0.360707	-3.58465	4.84673	8.22E-06	0.003685	3.462104	PD	
cg22629375	0.395132	3.731346	4.846156	8.23E-06	0.003691	3.469064	PD	
cg24867784	-0.20192	4.378743	-4.84585	8.24E-06	0.003693	3.461626	PD	
cg06720544	-0.25939	2.629553	-4.84456	8.28E-06	0.003709	3.473471	PD	
cg20202792	-0.27347	3.087143	-4.84367	8.31E-06	0.003719	3.458022	PD	
cg09493401	-0.28017	-5.39781	-4.84303	8.33E-06	0.003726	3.469675	PD	
cg24136288	-0.25877	3.771479	-4.8425	8.35E-06	0.003731	3.464302	PD	
cg21913335	0.762484	4.229128	4.841746	8.37E-06	0.003738	3.455577	PD	
cg21158664	-1.87271	5.35216	-4.84171	8.37E-06	0.003738	3.456461	PD	
cg07312178	-0.21144	4.158257	-4.84123	8.39E-06	0.003743	3.450926	PD	
cg12650985	-0.17734	5.256653	-4.84108	8.39E-06	0.003743	3.464842	PD	
cg02853812	-0.22482	2.945934	-4.84052	8.41E-06	0.003749	3.461076	PD	
cg04533116	-0.44304	3.258797	-4.83976	8.43E-06	0.003757	3.45722	PD	
cg00799637	-0.26734	3.665546	-4.83965	8.43E-06	0.003757	3.453329	PD	
cg11118092	-0.17363	3.556931	-4.83947	8.44E-06	0.003757	3.446431	PD	
cg25215885	0.388605	-3.75171	4.839045	8.45E-06	0.003761	3.436234	PD	
cg03435325	-0.27951	4.178914	-4.8387	8.46E-06	0.003764	3.449546	PD	
cg21390684	-0.26161	3.580954	-4.8379	8.49E-06	0.003773	3.456314	PD	
cg18157503	0.299003	3.053872	4.837183	8.51E-06	0.003781	3.43772	PD	
cg06294321	-0.40273	-4.57558	-4.83708	8.52E-06	0.003781	3.446675	PD	
cg00212272	0.157444	-3.36342	4.836632	8.53E-06	0.003785	3.434688	PD	
cg10881071	-0.28854	2.907699	-4.83641	8.54E-06	0.003786	3.437992	PD	
cg13641898	0.335434	-4.87515	4.835798	8.56E-06	0.003793	3.440298	PD	
cg24452347	-0.38955	-4.97763	-4.8343	8.60E-06	0.003812	3.441253	PD	
cg25507885	-0.295	1.811048	-4.8341	8.61E-06	0.003812	3.439523	PD	
cg01854211	-0.21997	3.141496	-4.83312	8.64E-06	0.003824	3.43304	PD	
cg27045832	0.265597	4.295449	4.83287	8.65E-06	0.003826	3.437536	PD	
cg18797504	-0.23378	2.632668	-4.82891	8.78E-06	0.00388	3.405314	PD	
cg04280969	0.367296	-5.36787	4.828193	8.80E-06	0.003888	3.419776	PD	
cg09663081	-0.26089	3.044422	-4.82704	8.84E-06	0.003903	3.404112	PD	

cg27185772	-0.1783	4.416763	-4.82631	8.86E-06	0.003911	3.410457	PD
cg11714377	0.301124	2.987736	4.824478	8.92E-06	0.003936	3.382541	PD
cg06453853	-0.216	2.869142	-4.82402	8.94E-06	0.00394	3.393433	PD
cg00257187	0.322196	3.778099	4.82289	8.97E-06	0.003955	3.395514	PD
cg00475194	-0.15222	3.666229	-4.82254	8.98E-06	0.003958	3.398843	PD
cg04621353	-0.2371	2.929256	-4.82043	9.05E-06	0.003986	3.363651	PD
cg06717750	-0.23936	3.867243	-4.82009	9.07E-06	0.003989	3.392522	PD
cg23349326	-0.26473	3.229028	-4.81803	9.13E-06	0.004018	3.376308	PD
cg23185217	0.462826	3.647927	4.817422	9.16E-06	0.004025	3.385805	PD
cg06574296	-0.30843	3.551527	-4.81712	9.17E-06	0.004027	3.374851	PD
cg07911933	-0.30817	3.532336	-4.81691	9.17E-06	0.004028	3.368509	PD
cg02554272	-0.30155	4.096452	-4.81678	9.18E-06	0.004028	3.381323	PD
cg03879623	-0.32211	3.266444	-4.81591	9.21E-06	0.004037	3.375722	PD
cg17111663	-0.25097	2.240698	-4.81588	9.21E-06	0.004037	3.373397	PD
cg09711801	0.266358	3.944354	4.815412	9.22E-06	0.004041	3.365786	PD
cg24412659	-0.20821	3.585607	-4.81532	9.23E-06	0.004041	3.372121	PD
cg15788649	-0.29327	2.95661	-4.81476	9.25E-06	0.004047	3.369815	PD
cg09392046	-0.15189	4.23971	-4.81453	9.25E-06	0.004048	3.369971	PD
cg26478331	-0.25967	3.127357	-4.81441	9.26E-06	0.004048	3.35353	PD
cg27247252	-0.35908	3.753416	-4.81365	9.28E-06	0.004058	3.370513	PD
cg22850234	0.609543	-5.03682	4.81247	9.32E-06	0.00407	3.343595	PD
cg09015824	0.337836	4.111687	4.812433	9.32E-06	0.00407	3.352972	PD
cg11468315	0.345561	-4.75425	4.812382	9.33E-06	0.00407	3.355751	PD
cg03972040	-0.22955	3.798462	-4.81026	9.40E-06	0.004096	3.356272	PD
cg25343135	-0.22047	3.516618	-4.81023	9.40E-06	0.004096	3.358782	PD
cg22379708	-0.20528	3.44154	-4.81014	9.40E-06	0.004096	3.354249	PD
cg05630994	-0.22851	2.885628	-4.81009	9.41E-06	0.004096	3.360836	PD
cg01963607	-0.23128	4.32463	-4.80997	9.41E-06	0.004096	3.357141	PD
cg15901999	-0.31421	3.916277	-4.80912	9.44E-06	0.004107	3.353074	PD
cg03400808	-0.26757	3.300158	-4.8082	9.47E-06	0.004119	3.341414	PD
cg23442796	0.183959	-5.56546	4.807765	9.49E-06	0.004121	3.335333	PD

cg04397457	-0.15368	3.695542	-4.80773	9.49E-06	0.004121	3.340844	PD
cg03580247	0.269579	4.221237	4.807095	9.51E-06	0.004129	3.336709	PD
cg00557293	0.253292	-6.17347	4.806597	9.53E-06	0.004132	3.33872	PD
cg12655768	0.331422	3.313	4.806597	9.53E-06	0.004132	3.3395	PD
cg22672496	-0.17422	3.412428	-4.80543	9.57E-06	0.004148	3.339584	PD
cg21268770	-0.2516	2.284453	-4.8041	9.62E-06	0.004166	3.336019	PD
cg12046362	-0.35024	4.448537	-4.8038	9.63E-06	0.004169	3.339622	PD
cg15532407	0.347828	4.057809	4.80353	9.64E-06	0.004171	3.334326	PD
cg11889058	-0.31309	4.461212	-4.80288	9.66E-06	0.004179	3.324974	PD
cg01655658	-2.68757	-4.14488	-4.8022	9.68E-06	0.004187	3.330783	PD
cg14429846	-0.27423	3.392349	-4.8018	9.70E-06	0.004191	3.33157	PD
cg22585117	0.187851	0.924151	4.801268	9.72E-06	0.004197	3.328758	PD
cg11210670	-0.29608	3.248976	-4.80053	9.74E-06	0.004206	3.315807	PD
cg15273811	-1.1855	3.070439	-4.79965	9.77E-06	0.004218	3.29375	PD
cg23604090	-0.2975	2.502096	-4.799	9.80E-06	0.004225	3.320443	PD
cg09375013	0.264052	-5.04275	4.798888	9.80E-06	0.004225	3.310566	PD
cg16275825	1.635397	2.905844	4.798784	9.81E-06	0.004225	3.308985	PD
cg23574719	-0.54795	2.531806	-4.79801	9.83E-06	0.004235	3.261188	PD
cg14696662	-0.14982	5.973656	-4.79757	9.85E-06	0.004239	3.298216	PD
cg11630554	-1.12264	-4.57244	-4.79711	9.87E-06	0.004244	3.302647	PD
cg21426686	-0.22622	3.86516	-4.79668	9.88E-06	0.004247	3.310959	PD
cg17274673	-0.22527	3.01731	-4.79663	9.88E-06	0.004247	3.30601	PD
cg00330746	-0.30471	3.307377	-4.79516	9.94E-06	0.004268	3.308254	PD
cg18464244	-0.30483	3.780704	-4.79487	9.95E-06	0.004271	3.294077	PD
cg23090400	-0.2445	3.180741	-4.79432	9.97E-06	0.004277	3.299589	PD
cg22820238	-0.33163	3.37017	-4.79366	9.99E-06	0.004283	3.300685	PD
cg16562217	-0.28824	4.046385	-4.79364	9.99E-06	0.004283	3.29153	PD
cg00939556	-0.231	3.952416	-4.7932	1.00E-05	0.004288	3.300206	PD
cg19447984	-0.21092	3.596176	-4.79285	1.00E-05	0.004292	3.295287	PD
cg19124596	-0.21554	2.725004	-4.79232	1.00E-05	0.004298	3.299487	PD
cg10130694	0.159489	-3.54971	4.792127	1.00E-05	0.004299	3.294765	PD

cg06099512	0.359609	3.230688	4.791355	1.01E-05	0.004309	3.299099	PD
cg12445586	-0.25554	3.215461	-4.79061	1.01E-05	0.004316	3.277633	PD
cg10220320	-0.15423	3.7766	-4.7906	1.01E-05	0.004316	3.254231	PD
cg12251126	0.657251	3.621235	4.790396	1.01E-05	0.004317	3.29414	PD
cg25776930	-0.37949	3.746576	-4.79022	1.01E-05	0.004318	3.292016	PD
cg21766141	-0.25501	1.898251	-4.78998	1.01E-05	0.00432	3.289613	PD
cg08331427	-0.2842	3.629541	-4.78945	1.01E-05	0.004325	3.286814	PD
cg24226687	1.072575	-6.12654	4.789362	1.02E-05	0.004325	3.281253	PD
cg14933676	-0.44445	4.095109	-4.78875	1.02E-05	0.004333	3.235519	PD
cg23760945	-0.22788	3.198186	-4.78779	1.02E-05	0.004346	3.285196	PD
cg27398051	-0.19506	3.856195	-4.78655	1.03E-05	0.004363	3.276132	PD
cg13395017	-0.21908	3.670411	-4.78609	1.03E-05	0.004368	3.27666	PD
cg16901161	0.204852	3.611899	4.785282	1.03E-05	0.004379	3.262465	PD
cg15639842	0.278614	3.297906	4.784932	1.03E-05	0.004383	3.263501	PD
cg11426844	-0.31166	3.985849	-4.78388	1.04E-05	0.004397	3.262944	PD
cg13745593	-0.23544	3.594733	-4.78337	1.04E-05	0.004403	3.266093	PD
cg18118147	-0.13829	4.496685	-4.78193	1.04E-05	0.004425	3.258805	PD
cg02121736	-0.66693	-4.31989	-4.78089	1.05E-05	0.004439	3.231293	PD
cg11108115	-0.20626	4.019093	-4.77942	1.05E-05	0.004461	3.257446	PD
cg00837434	0.252705	-4.25999	4.778073	1.06E-05	0.004481	3.246493	PD
cg22387113	-0.31176	3.100725	-4.77762	1.06E-05	0.004486	3.247006	PD
cg17276106	0.329631	2.39378	4.777098	1.06E-05	0.004493	3.232967	PD
cg20252997	0.340217	-1.7095	4.776137	1.07E-05	0.004506	3.225789	PD
cg03307425	-0.16044	4.738404	-4.77539	1.07E-05	0.004516	3.240484	PD
cg23348610	0.172772	3.845738	4.774725	1.07E-05	0.004525	3.236736	PD
cg04041880	-0.15927	-0.41578	-4.77388	1.07E-05	0.004537	3.239632	PD
cg03938711	0.294689	3.730506	4.77297	1.08E-05	0.00455	3.233079	PD
cg25399148	-0.25173	2.701232	-4.77268	1.08E-05	0.004552	3.235646	PD
cg03721887	0.690851	3.00226	4.772475	1.08E-05	0.004553	3.223211	PD
cg02912041	0.327281	4.081898	4.772045	1.08E-05	0.004558	3.226492	PD
cg23274883	-0.43912	3.457175	-4.7695	1.09E-05	0.004598	3.211481	PD

cg06210526	-0.30607	2.902712	-4.76942	1.09E-05	0.004598	3.205768	PD
cg13627446	-0.21573	2.757271	-4.76785	1.10E-05	0.004622	3.21333	PD
cg10099679	-0.17681	4.040217	-4.76646	1.10E-05	0.004642	3.211592	PD
cg08291385	0.482014	3.548559	4.766398	1.10E-05	0.004642	3.206559	PD
cg10610477	0.529672	2.698329	4.765808	1.11E-05	0.00465	3.204343	PD
cg10185767	-0.49049	2.654456	-4.76564	1.11E-05	0.00465	3.210221	PD
cg09939441	-0.22215	2.60775	-4.76532	1.11E-05	0.004654	3.207651	PD
cg09966920	-0.51355	3.817442	-4.76504	1.11E-05	0.004655	3.211096	PD
cg12562232	0.383798	4.196991	4.76488	1.11E-05	0.004655	3.165559	PD
cg26159128	-0.45178	3.769791	-4.76484	1.11E-05	0.004655	3.208243	PD
cg01451880	-0.27372	4.451241	-4.76462	1.11E-05	0.004656	3.193257	PD
cg11917413	-0.30723	2.770658	-4.76401	1.11E-05	0.004664	3.202934	PD
cg27162392	-0.21744	3.421842	-4.76379	1.11E-05	0.004665	3.203865	PD
cg07817170	-0.26408	1.866749	-4.76374	1.12E-05	0.004665	3.190963	PD
cg15011376	0.462611	3.346182	4.763325	1.12E-05	0.004669	3.194559	PD
cg23796743	-0.2008	3.442651	-4.76312	1.12E-05	0.00467	3.201209	PD
cg03775632	-0.30734	2.86587	-4.76289	1.12E-05	0.004672	3.202278	PD
cg06304986	-0.29111	3.693967	-4.76222	1.12E-05	0.004681	3.195571	PD
cg14749262	-0.32692	3.18527	-4.76115	1.13E-05	0.004697	3.194143	PD
cg10735834	-0.75027	3.711109	-4.75923	1.13E-05	0.004728	3.170802	PD
cg21888978	-0.2489	3.093452	-4.75846	1.14E-05	0.004737	3.185351	PD
cg09460477	0.214375	-3.38765	4.758444	1.14E-05	0.004737	3.182624	PD
cg07865573	-0.21662	4.386693	-4.75827	1.14E-05	0.004738	3.182422	PD
cg00145707	-0.36818	4.268637	-4.75748	1.14E-05	0.004746	3.181407	PD
cg04671971	-0.54699	3.701246	-4.75741	1.14E-05	0.004746	3.182217	PD
cg17226232	-0.16362	4.007359	-4.75739	1.14E-05	0.004746	3.177519	PD
cg04954056	-0.22823	4.235344	-4.75699	1.14E-05	0.004751	3.176394	PD
cg06760904	-0.30421	2.63067	-4.75672	1.14E-05	0.004753	3.179715	PD
cg05287321	0.302756	4.584449	4.756257	1.15E-05	0.004759	3.172656	PD
cg25101337	-0.35665	4.047826	-4.75465	1.15E-05	0.004785	3.170038	PD
cg15849465	-0.34513	3.690174	-4.75404	1.16E-05	0.004793	3.171217	PD

cg02403349	-0.73917	-4.06035	-4.75037	1.17E-05	0.004855	3.114805	PD
cg16440217	-0.25635	3.932099	-4.7497	1.17E-05	0.004865	3.157652	PD
cg07908574	-0.17077	3.639952	-4.7483	1.18E-05	0.004888	3.15084	PD
cg00982736	-0.20792	3.797339	-4.747	1.19E-05	0.004909	3.145898	PD
cg14389535	0.38968	3.663876	4.746605	1.19E-05	0.004913	3.137	PD
cg00341488	-0.25745	3.604023	-4.74294	1.20E-05	0.004977	3.130562	PD
cg08547352	-0.19993	4.188019	-4.74209	1.21E-05	0.00499	3.134535	PD
cg20494878	0.408295	2.642056	4.741534	1.21E-05	0.004998	3.131799	PD
cg19240752	-0.14868	4.313636	-4.74092	1.21E-05	0.005007	3.121032	PD
cg24590621	-0.21782	3.471347	-4.73803	1.23E-05	0.005055	3.101563	PD
cg16584557	-0.56363	3.064162	-4.73801	1.23E-05	0.005055	3.105478	PD
cg17094363	-0.16228	3.89349	-4.73747	1.23E-05	0.005063	3.1182	PD
cg09088153	-0.2628	-1.90185	-4.73718	1.23E-05	0.005066	3.111732	PD
cg01177800	-0.23422	2.771974	-4.73657	1.23E-05	0.005075	3.108033	PD
cg07096313	-0.35589	4.16798	-4.73558	1.24E-05	0.005091	3.100855	PD
cg06426818	-0.23357	3.687481	-4.73543	1.24E-05	0.005091	3.088702	PD
cg05942843	-0.24053	2.886196	-4.7346	1.24E-05	0.005104	3.107472	PD
cg07960381	0.665778	3.51522	4.734141	1.24E-05	0.00511	3.086448	PD
cg12949530	0.286098	4.4804	4.734028	1.24E-05	0.00511	3.104542	PD
cg22478135	-0.1973	4.076085	-4.73364	1.25E-05	0.005114	3.100395	PD
cg04330213	-0.42258	-5.1978	-4.73334	1.25E-05	0.005117	3.10341	PD
cg16088380	-0.18862	3.541052	-4.73243	1.25E-05	0.005132	3.093326	PD
cg12582896	0.91509	5.471034	4.73099	1.26E-05	0.005157	3.075874	PD
cg24140362	-0.23597	3.542668	-4.73001	1.26E-05	0.005173	3.094555	PD
cg26054842	-0.2625	3.241415	-4.72845	1.27E-05	0.0052	3.074074	PD
cg25242498	-0.21957	3.444834	-4.7278	1.27E-05	0.00521	3.081503	PD
cg25808454	1.088223	2.642448	4.727016	1.28E-05	0.005222	3.068211	PD
cg07123579	-0.20458	3.183308	-4.72666	1.28E-05	0.005226	3.072124	PD
cg10252897	-0.20331	2.796716	-4.72486	1.29E-05	0.005258	3.056923	PD
cg04533266	-0.28662	3.367474	-4.72471	1.29E-05	0.005259	3.071109	PD
cg19153300	0.626064	3.186401	4.724254	1.29E-05	0.005265	3.061911	PD

cg03373785	-0.186	3.665106	-4.72413	1.29E-05	0.005265	3.071035	PD
cg15015119	-0.23354	4.391862	-4.72353	1.29E-05	0.005274	3.067395	PD
cg02426324	-0.16434	3.501872	-4.71789	1.32E-05	0.005381	3.049623	PD
cg18715193	-0.52805	2.594408	-4.7171	1.32E-05	0.005394	3.03113	PD
cg20647939	-0.36814	4.687758	-4.71504	1.33E-05	0.005432	3.040933	PD
cg13676665	-0.16115	2.822988	-4.71335	1.34E-05	0.005463	3.035897	PD
cg02444928	-0.35234	3.9014	-4.71113	1.35E-05	0.005505	3.024373	PD
cg12529895	0.31526	3.621225	4.709204	1.36E-05	0.005538	3.022298	PD
cg14382976	-0.24735	3.278123	-4.70908	1.36E-05	0.005538	3.021476	PD
cg25359495	-0.32404	4.088668	-4.70898	1.36E-05	0.005538	3.015814	PD
cg07699237	-0.17348	5.352131	-4.70892	1.36E-05	0.005538	3.020589	PD
cg13276615	0.649782	3.300191	4.707704	1.37E-05	0.00556	3.006035	PD
cg06972043	-0.22162	3.176517	-4.70728	1.37E-05	0.005566	3.013176	PD
cg02747644	-0.25276	3.323483	-4.70684	1.37E-05	0.005573	3.005484	PD
cg01881574	-0.76505	4.766312	-4.70522	1.38E-05	0.005603	3.011176	PD
cg10578777	-0.53645	2.986478	-4.70505	1.38E-05	0.005604	2.993997	PD
cg07892964	-0.5414	3.616418	-4.70478	1.38E-05	0.005606	2.984612	PD
cg08879111	-0.24859	2.243864	-4.70466	1.38E-05	0.005606	3.005356	PD
cg17700087	-0.37328	3.868668	-4.70452	1.38E-05	0.005606	3.002324	PD
cg22095965	0.473233	3.998085	4.704116	1.39E-05	0.005612	3.007096	PD
cg02544829	-0.24603	3.350042	-4.70393	1.39E-05	0.005613	3.004161	PD
cg05920119	0.356924	3.726818	4.703605	1.39E-05	0.005617	2.999505	PD
cg10399865	-0.22126	4.199228	-4.70328	1.39E-05	0.00562	2.999126	PD
cg07664909	-0.26078	2.587836	-4.70322	1.39E-05	0.00562	2.999926	PD
cg11258982	-0.26337	2.66535	-4.70178	1.40E-05	0.005647	2.986295	PD
cg03317682	-0.49402	3.53381	-4.70157	1.40E-05	0.005648	2.982464	PD
cg25113595	0.270965	4.471182	4.699514	1.41E-05	0.005688	2.985983	PD
cg17589883	-0.19489	4.192416	-4.69861	1.41E-05	0.005704	2.986427	PD
cg17173881	-0.24447	2.7293	-4.69837	1.42E-05	0.005706	2.98748	PD
cg24536474	-0.25407	2.917739	-4.69708	1.42E-05	0.00573	2.982995	PD
cg10859636	-0.30461	3.071338	-4.69668	1.42E-05	0.005736	2.980604	PD

cg03777414	-0.22308	2.442371	-4.69655	1.43E-05	0.005736	2.975866	PD
cg08826095	-0.27068	3.139798	-4.69621	1.43E-05	0.005741	2.978703	PD
cg05679504	-0.2384	3.004999	-4.6956	1.43E-05	0.005751	2.955965	PD
cg04103706	-1.43239	3.354702	-4.69521	1.43E-05	0.005756	2.967567	PD
cg09674368	-0.26782	3.113347	-4.69499	1.43E-05	0.005758	2.973426	PD
cg11858698	-0.42872	3.52278	-4.69461	1.44E-05	0.005763	2.975742	PD
cg10699846	0.467751	3.743821	4.694358	1.44E-05	0.005766	2.959379	PD
cg24479590	0.27565	-4.25915	4.693515	1.44E-05	0.005781	2.968801	PD
cg07987135	-0.42252	4.203731	-4.69336	1.44E-05	0.005781	2.97116	PD
cg12777296	-0.27497	3.592987	-4.69218	1.45E-05	0.005803	2.96347	PD
cg27535148	-0.18752	2.050654	-4.69121	1.45E-05	0.005821	2.959508	PD
cg01663649	-0.21417	2.77607	-4.69022	1.46E-05	0.005839	2.952228	PD
cg01195053	-0.3937	-5.86271	-4.6901	1.46E-05	0.005839	2.954972	PD
cg00506629	0.192523	-4.83677	4.689118	1.46E-05	0.005857	2.942706	PD
cg09933401	-0.1895	4.089533	-4.68888	1.47E-05	0.00586	2.954571	PD
cg03821718	0.175538	3.866918	4.687613	1.47E-05	0.005884	2.949979	PD
cg01190419	-0.32995	4.06611	-4.68742	1.47E-05	0.005885	2.918714	PD
cg06060803	0.29891	4.144268	4.687068	1.48E-05	0.00589	2.946393	PD
cg10758676	-0.35839	3.860098	-4.68669	1.48E-05	0.005895	2.936482	PD
cg01094351	-0.38709	4.770492	-4.6849	1.49E-05	0.005931	2.94311	PD
cg18687588	-0.32332	4.083846	-4.6844	1.49E-05	0.005939	2.940607	PD
cg01600968	-0.31699	4.087778	-4.68418	1.49E-05	0.005941	2.940904	PD
cg04949238	-0.36041	3.155656	-4.68369	1.49E-05	0.005948	2.936082	PD
cg12902803	-0.24783	2.359917	-4.68358	1.49E-05	0.005948	2.939059	PD
cg03735043	0.767939	4.266313	4.68296	1.50E-05	0.005957	2.929967	PD
cg15123087	-0.61064	3.675169	-4.68283	1.50E-05	0.005957	2.934399	PD
cg09981013	-0.39538	5.847611	-4.68278	1.50E-05	0.005957	2.936861	PD
cg24075745	-0.11038	4.830167	-4.68198	1.50E-05	0.005972	2.916486	PD
cg08455905	1.138171	3.051619	4.680548	1.51E-05	0.006	2.922523	PD
cg22173380	-0.17634	3.225769	-4.68029	1.51E-05	0.006003	2.918334	PD
cg12312765	-0.19377	3.358378	-4.68008	1.51E-05	0.006005	2.923795	PD

cg02154924	0.706921	3.716106	4.679763	1.52E-05	0.006009	2.877592	PD
cg05280944	-0.32779	3.901147	-4.67961	1.52E-05	0.00601	2.922647	PD
cg01157447	-0.22419	2.905328	-4.67906	1.52E-05	0.006019	2.912474	PD
cg23340313	-0.40533	-6.16079	-4.6786	1.52E-05	0.006026	2.917666	PD
cg05231837	-0.34277	3.637141	-4.67812	1.52E-05	0.006034	2.919761	PD
cg06754542	-0.1808	4.223397	-4.67612	1.54E-05	0.006075	2.907691	PD
cg05515244	0.914483	1.763578	4.675977	1.54E-05	0.006075	2.902608	PD
cg25340432	-0.20793	4.299236	-4.67568	1.54E-05	0.006077	2.914753	PD
cg07812047	-0.32564	2.976009	-4.67564	1.54E-05	0.006077	2.91176	PD
cg01538218	0.182619	-1.51891	4.674493	1.54E-05	0.0061	2.908363	PD
cg03559186	-0.25802	2.023397	-4.6732	1.55E-05	0.006126	2.904294	PD
cg18635968	0.231947	-5.16218	4.67277	1.55E-05	0.006132	2.901188	PD
cg15207993	-0.2111	3.345566	-4.67188	1.56E-05	0.006149	2.898132	PD
cg22226928	-0.18507	3.035905	-4.67178	1.56E-05	0.006149	2.884668	PD
cg03728327	0.268746	3.907306	4.671456	1.56E-05	0.006153	2.901632	PD
cg12536112	-0.30005	3.396664	-4.67133	1.56E-05	0.006153	2.891019	PD
cg02417576	-0.23392	3.572861	-4.67094	1.56E-05	0.006159	2.89761	PD
cg12952273	0.351581	-5.09738	4.667014	1.59E-05	0.006245	2.861352	PD
cg26944725	-0.58555	4.274723	-4.66577	1.59E-05	0.00627	2.881617	PD
cg06641959	-0.31953	-5.869	-4.66358	1.61E-05	0.006317	2.873788	PD
cg02051562	-0.21949	2.935976	-4.66306	1.61E-05	0.006326	2.871514	PD
cg08775122	-0.16504	3.477927	-4.66141	1.62E-05	0.006361	2.859743	PD
cg01075095	-0.25591	3.318294	-4.66055	1.63E-05	0.006377	2.861733	PD
cg25359981	0.258258	3.559491	4.660465	1.63E-05	0.006377	2.861374	PD
cg17693878	-0.17532	3.55706	-4.66022	1.63E-05	0.00638	2.85988	PD
cg12130225	-0.25008	3.036789	-4.65954	1.63E-05	0.006393	2.855203	PD
cg01026064	-0.28856	3.597428	-4.65848	1.64E-05	0.006414	2.846672	PD
cg23431098	0.23076	3.767735	4.657692	1.64E-05	0.00643	2.85223	PD
cg08017245	-0.17354	4.277707	-4.65625	1.65E-05	0.006461	2.840101	PD
cg04499104	-0.16774	3.60823	-4.65612	1.65E-05	0.006461	2.84679	PD
cg13823477	-0.20678	3.537302	-4.65572	1.65E-05	0.006467	2.842515	PD

cg05778559	0.404887	4.216437	4.653895	1.66E-05	0.006507	2.83709	PD
cg27487288	-0.34296	3.297916	-4.65308	1.67E-05	0.006523	2.835478	PD
cg06572748	0.446302	2.52734	4.652922	1.67E-05	0.006524	2.835886	PD
cg24992936	0.305842	-4.96394	4.652042	1.68E-05	0.006541	2.81586	PD
cg25050541	-0.21638	3.64163	-4.65195	1.68E-05	0.006541	2.834498	PD
cg24204477	-0.18484	3.395241	-4.65128	1.68E-05	0.006554	2.829973	PD
cg10437627	0.173153	-3.65766	4.651065	1.68E-05	0.006556	2.82959	PD
cg19444390	-0.15171	4.544765	-4.65047	1.69E-05	0.006567	2.830821	PD
cg02017860	0.27615	4.305864	4.648432	1.70E-05	0.006613	2.823315	PD
cg04411166	-0.18596	4.613384	-4.64724	1.71E-05	0.006638	2.820497	PD
cg21008046	-0.20153	3.657881	-4.64696	1.71E-05	0.006642	2.804697	PD
cg04048705	0.230963	4.037436	4.646389	1.71E-05	0.006649	2.812508	PD
cg17392909	0.194657	-3.18606	4.646334	1.71E-05	0.006649	2.812646	PD
cg11684734	-0.24335	3.871491	-4.64628	1.71E-05	0.006649	2.817768	PD
cg20830627	-0.29421	3.497283	-4.64605	1.71E-05	0.006652	2.814556	PD
cg03681640	-0.319	3.830925	-4.64545	1.72E-05	0.006663	2.80919	PD
cg05428777	-0.30881	3.885008	-4.64487	1.72E-05	0.006674	2.810572	PD
cg06929471	-0.23677	3.537891	-4.64423	1.72E-05	0.006687	2.803958	PD
cg16371865	-0.2537	3.408301	-4.64361	1.73E-05	0.006699	2.806658	PD
cg06390585	-0.19032	3.951432	-4.6429	1.73E-05	0.006713	2.799905	PD
cg12213910	-0.45668	4.282464	-4.64225	1.74E-05	0.006726	2.804789	PD
cg04710757	-0.25854	3.6199	-4.64203	1.74E-05	0.006728	2.799312	PD
cg26679027	-0.1881	3.749186	-4.64157	1.74E-05	0.006736	2.800492	PD
cg07570618	-0.27468	3.440032	-4.64143	1.74E-05	0.006737	2.787728	PD
cg02719051	-0.24434	2.094858	-4.64066	1.75E-05	0.006747	2.797251	PD
cg13168317	-0.455	5.533432	-4.64048	1.75E-05	0.006747	2.79241	PD
cg14522097	-0.36883	2.711549	-4.64044	1.75E-05	0.006747	2.796878	PD
cg21325760	-0.15705	4.290458	-4.64017	1.75E-05	0.006747	2.794681	PD
cg08542066	-0.71369	3.588607	-4.64013	1.75E-05	0.006747	2.772322	PD
cg06239923	-0.44348	4.20955	-4.64003	1.75E-05	0.006747	2.793859	PD
cg00768873	-0.33244	2.436098	-4.63996	1.75E-05	0.006747	2.792655	PD

cg06879681	-0.3521	2.882658	-4.63991	1.75E-05	0.006747	2.793454	PD
cg22167283	-0.34374	4.209104	-4.63985	1.75E-05	0.006747	2.794528	PD
cg22268467	-0.19393	4.256527	-4.63873	1.76E-05	0.006772	2.790334	PD
cg12461086	-0.30953	3.548519	-4.63817	1.76E-05	0.006783	2.785812	PD
cg23738564	0.63212	3.335938	4.637401	1.77E-05	0.006798	2.785356	PD
cg00657529	-0.27459	2.730016	-4.63678	1.77E-05	0.00681	2.783763	PD
cg15479572	-0.43813	3.993913	-4.63662	1.77E-05	0.006811	2.780389	PD
cg01613643	-0.20811	2.483786	-4.63629	1.77E-05	0.006816	2.770586	PD
cg05651393	-0.13256	3.306218	-4.63601	1.78E-05	0.00682	2.773063	PD
cg27631766	-0.22273	3.851931	-4.63588	1.78E-05	0.00682	2.777494	PD
cg23800304	-0.27965	4.29239	-4.63473	1.78E-05	0.006846	2.777944	PD
cg24300714	0.312703	-5.14021	4.633879	1.79E-05	0.006864	2.770331	PD
cg16768379	-0.26272	2.914862	-4.63349	1.79E-05	0.00687	2.767107	PD
cg09337174	-0.23349	2.764789	-4.6328	1.80E-05	0.006884	2.77063	PD
cg14759378	0.24875	4.200714	4.632186	1.80E-05	0.006896	2.771614	PD
cg27000503	-0.2607	3.175893	-4.63207	1.80E-05	0.006896	2.768989	PD
cg12315994	-0.28839	3.633843	-4.63114	1.81E-05	0.006917	2.764469	PD
cg05640992	-0.21058	3.47905	-4.63072	1.81E-05	0.006924	2.763869	PD
cg09765807	0.390882	4.063527	4.629794	1.82E-05	0.006944	2.75699	PD
cg09385032	-0.30213	3.474562	-4.62844	1.83E-05	0.006975	2.755077	PD
cg13527508	0.35375	-5.28629	4.628136	1.83E-05	0.00698	2.753671	PD
cg15819225	-0.22625	2.509916	-4.62743	1.83E-05	0.006993	2.753529	PD
cg02388319	-0.17099	4.011583	-4.62735	1.83E-05	0.006993	2.75463	PD
cg06882198	0.283491	3.50107	4.627142	1.83E-05	0.006993	2.745599	PD
cg08245053	-0.19814	4.0313	-4.62703	1.84E-05	0.006993	2.743223	PD
cg09927311	-0.33826	3.760153	-4.62677	1.84E-05	0.006993	2.752038	PD
cg11885489	-0.3131	4.086164	-4.62676	1.84E-05	0.006993	2.748505	PD
cg04830695	-0.28136	3.565304	-4.62672	1.84E-05	0.006993	2.742814	PD
cg02348989	0.320204	3.842226	4.625931	1.84E-05	0.00701	2.741403	PD
cg14483806	0.77459	3.653401	4.625445	1.85E-05	0.007019	2.742186	PD
cg14501138	-0.32792	3.517473	-4.62526	1.85E-05	0.007021	2.745293	PD

cg16657886	-0.16025	2.901902	-4.62467	1.85E-05	0.007033	2.74123	PD
cg03343363	-0.26962	2.55161	-4.62397	1.86E-05	0.007047	2.741851	PD
cg06834916	0.429592	4.188238	4.623217	1.86E-05	0.007064	2.738658	PD
cg22673332	-0.28375	3.065256	-4.62248	1.87E-05	0.007079	2.723268	PD
cg06203744	-0.28257	2.391355	-4.62235	1.87E-05	0.007079	2.731921	PD
cg18647048	-0.26408	2.052493	-4.62101	1.88E-05	0.007111	2.730635	PD
cg08785933	-0.34978	3.687741	-4.61923	1.89E-05	0.007154	2.714905	PD
cg19952704	-0.98821	-4.94726	-4.61908	1.89E-05	0.007154	2.703658	PD
cg06893172	-0.16569	3.680287	-4.61891	1.89E-05	0.007155	2.726839	PD
cg26405198	-0.62266	4.159108	-4.6188	1.89E-05	0.007155	2.719526	PD
cg21261798	-0.2294	3.833436	-4.61859	1.89E-05	0.007157	2.722808	PD
cg13683667	-0.3647	3.523187	-4.61828	1.89E-05	0.007162	2.724348	PD
cg10258241	-0.17089	4.476203	-4.61817	1.90E-05	0.007162	2.72557	PD
cg09858892	-0.29021	1.490486	-4.61727	1.90E-05	0.007182	2.715386	PD
cg17951978	-0.46698	3.486227	-4.61696	1.90E-05	0.007187	2.720908	PD
cg03378637	-0.19866	3.685392	-4.61666	1.91E-05	0.007191	2.718065	PD
cg10449466	-0.20305	2.976202	-4.61584	1.91E-05	0.007209	2.715185	PD
cg09041747	0.394948	3.598436	4.615452	1.91E-05	0.007216	2.712734	PD
cg09907509	-0.28244	-5.90912	-4.61472	1.92E-05	0.007229	2.707883	PD
cg06869039	-0.21558	2.493733	-4.6147	1.92E-05	0.007229	2.711313	PD
cg16876255	0.212866	-5.90715	4.614155	1.92E-05	0.007241	2.704734	PD
cg14333548	-0.16527	3.550793	-4.61346	1.93E-05	0.007256	2.698685	PD
cg21056275	-0.18559	3.27645	-4.61191	1.94E-05	0.007293	2.703006	PD
cg11855895	-0.17503	3.572199	-4.61075	1.95E-05	0.00732	2.691129	PD
cg19166759	-0.29055	3.471746	-4.60993	1.95E-05	0.007339	2.690005	PD
cg19588538	-0.42205	4.447527	-4.60918	1.96E-05	0.007356	2.686064	PD
cg01869224	-0.45563	3.142316	-4.60903	1.96E-05	0.007356	2.689458	PD
cg25552548	-0.44836	3.659858	-4.60878	1.96E-05	0.00736	2.685812	PD
cg23344964	-0.22501	3.020844	-4.60836	1.96E-05	0.007368	2.690565	PD
cg10317162	-0.33034	4.150814	-4.60652	1.98E-05	0.007414	2.683155	PD
cg11654794	-0.30422	4.128154	-4.60578	1.98E-05	0.00743	2.682764	PD

cg21697769	-0.35575	3.030562	-4.60466	1.99E-05	0.007457	2.675858	PD
cg08266576	-0.21241	2.590902	-4.60404	1.99E-05	0.00747	2.671572	PD
cg09819346	0.127657	-0.60373	4.60356	2.00E-05	0.00748	2.673084	PD
cg10951117	-0.26951	1.634638	-4.60304	2.00E-05	0.007491	2.673185	PD
cg25509106	-0.26664	3.645946	-4.60278	2.00E-05	0.007494	2.670961	PD
cg02555585	-0.27496	3.017143	-4.60229	2.01E-05	0.007502	2.669111	PD
cg06291698	-0.43145	-4.43066	-4.60226	2.01E-05	0.007502	2.661098	PD
cg06188476	0.285493	4.023965	4.60201	2.01E-05	0.007505	2.672213	PD
cg09183742	-0.33146	-4.08438	-4.60105	2.02E-05	0.007526	2.665951	PD
cg23479557	-0.21568	2.625872	-4.60098	2.02E-05	0.007526	2.664013	PD
cg11153505	-0.17167	3.877992	-4.60089	2.02E-05	0.007526	2.666973	PD
cg01073521	-0.23944	3.669012	-4.60067	2.02E-05	0.007529	2.668575	PD
cg24430322	-0.22429	2.649562	-4.6005	2.02E-05	0.00753	2.66348	PD
cg10845608	-0.18195	3.796685	-4.6003	2.02E-05	0.00753	2.665941	PD
cg13089154	-0.27225	-5.27719	-4.60019	2.02E-05	0.00753	2.663085	PD
cg09533458	0.254436	4.454868	4.600118	2.02E-05	0.00753	2.659144	PD
cg20294640	-0.17552	3.567344	-4.59991	2.02E-05	0.007533	2.663156	PD
cg07189898	-0.3868	3.801413	-4.59974	2.03E-05	0.007534	2.658568	PD
cg13745933	-0.21411	4.653002	-4.59818	2.04E-05	0.007573	2.656906	PD
cg11807238	-0.3372	3.789462	-4.59619	2.05E-05	0.007624	2.65105	PD
cg05664331	-0.23844	3.304227	-4.59534	2.06E-05	0.007644	2.649566	PD
cg08559711	-0.19621	3.064009	-4.59524	2.06E-05	0.007644	2.647141	PD
cg04619437	-0.17157	3.810551	-4.59485	2.06E-05	0.007651	2.646734	PD
cg09484009	0.27516	-4.71321	4.594473	2.06E-05	0.007658	2.63944	PD
cg08955358	-0.21901	3.015559	-4.59428	2.07E-05	0.00766	2.642198	PD
cg18762849	0.19309	-5.5806	4.593869	2.07E-05	0.007668	2.637847	PD
cg04560163	-0.18969	3.541561	-4.59361	2.07E-05	0.007669	2.633114	PD
cg12034488	-0.14388	3.803033	-4.5936	2.07E-05	0.007669	2.636655	PD
cg02338146	-0.26694	3.601545	-4.5924	2.08E-05	0.007699	2.631073	PD
cg27233174	-0.35014	3.769706	-4.59229	2.08E-05	0.007699	2.634449	PD
cg17834606	-0.42164	3.393852	-4.59212	2.08E-05	0.0077	2.633533	PD

cg20496693	0.853469	-6.23843	4.59194	2.08E-05	0.007702	2.635929	PD
cg25980682	-0.19287	3.546454	-4.59161	2.09E-05	0.007708	2.633231	PD
cg00027420	-0.24907	2.746119	-4.59072	2.09E-05	0.007727	2.624178	PD
cg26875874	0.507632	-4.72786	4.590682	2.09E-05	0.007727	2.630452	PD
cg10286056	-0.2567	2.824783	-4.59045	2.09E-05	0.007729	2.631498	PD
cg21078196	-0.17927	3.702623	-4.59037	2.10E-05	0.007729	2.627213	PD
cg10365337	-0.2061	3.401694	-4.58987	2.10E-05	0.007739	2.626047	PD
cg07380506	-0.69801	-4.41135	-4.58929	2.10E-05	0.007752	2.620399	PD
cg05622550	-0.16484	4.284946	-4.58894	2.11E-05	0.007758	2.628443	PD
cg12837896	-0.26015	-5.81035	-4.58861	2.11E-05	0.007763	2.627002	PD
cg00258199	0.247688	2.646917	4.588551	2.11E-05	0.007763	2.586918	PD
cg08781069	-0.37739	3.852172	-4.58842	2.11E-05	0.007763	2.622593	PD
cg27555130	-0.24901	2.875916	-4.58766	2.12E-05	0.007778	2.616421	PD
cg16389924	-0.18503	3.67096	-4.58763	2.12E-05	0.007778	2.613464	PD
cg00792976	-0.26277	3.580142	-4.58624	2.13E-05	0.007814	2.62112	PD
cg24486518	-0.23322	2.881695	-4.585	2.14E-05	0.007846	2.612469	PD
cg19040148	-0.28122	4.11977	-4.58451	2.14E-05	0.007856	2.611416	PD
cg02633953	-0.19528	3.961682	-4.58361	2.15E-05	0.007876	2.608033	PD
cg19263847	-0.29127	4.233326	-4.58347	2.15E-05	0.007876	2.610003	PD
cg23421288	0.344658	3.51783	4.583462	2.15E-05	0.007876	2.611747	PD
cg11610140	-0.21899	2.952452	-4.58236	2.16E-05	0.007904	2.605532	PD
cg21646366	0.271135	1.479916	4.582197	2.16E-05	0.007905	2.596087	PD
cg14228300	-1.17756	-4.20706	-4.58197	2.16E-05	0.007907	2.579362	PD
cg15457689	-0.23644	2.833918	-4.58189	2.16E-05	0.007907	2.593793	PD
cg03257308	0.386893	3.182599	4.581531	2.16E-05	0.007911	2.602281	PD
cg21625445	-0.15527	1.095706	-4.58151	2.16E-05	0.007911	2.604613	PD
cg21623395	-0.20679	3.067425	-4.58098	2.17E-05	0.007922	2.603685	PD
cg21922656	-0.23189	4.148741	-4.58025	2.17E-05	0.00794	2.599513	PD
cg20832020	0.205028	4.218121	4.580056	2.17E-05	0.007941	2.599335	PD
cg04607412	-0.25692	1.860398	-4.57996	2.18E-05	0.007941	2.598166	PD
cg15788231	-0.25822	3.525563	-4.57976	2.18E-05	0.007944	2.598898	PD

cg11829680	-0.27362	3.269385	-4.57964	2.18E-05	0.007944	2.582135	PD
cg02188665	0.210519	1.172656	4.579452	2.18E-05	0.007946	2.592782	PD
cg04918770	-0.21325	2.986	-4.57913	2.18E-05	0.007951	2.58566	PD
cg08220377	-0.24521	2.386213	-4.57857	2.19E-05	0.007964	2.592216	PD
cg12861945	-0.84441	-4.54991	-4.57819	2.19E-05	0.007971	2.585619	PD
cg14654833	0.476126	2.648865	4.577982	2.19E-05	0.007974	2.58533	PD
cg04124112	0.251371	-5.40447	4.57698	2.20E-05	0.007999	2.582828	PD
cg07577837	-0.2482	2.880954	-4.57673	2.20E-05	0.008003	2.584505	PD
cg07012231	-0.13334	4.579321	-4.57662	2.20E-05	0.008003	2.586643	PD
cg05886537	0.18001	4.196283	4.57638	2.20E-05	0.008006	2.57507	PD
cg04363000	-0.23865	2.803137	-4.57415	2.22E-05	0.008065	2.5777	PD
cg12332172	-0.30307	2.339274	-4.57413	2.22E-05	0.008065	2.575057	PD
cg04260575	-0.18086	3.518491	-4.57386	2.22E-05	0.008069	2.573018	PD
cg16832567	-0.38141	4.173032	-4.57374	2.22E-05	0.008069	2.578843	PD
cg09006514	-0.23276	2.936259	-4.57346	2.23E-05	0.008074	2.57607	PD
cg08990543	-0.36089	3.387646	-4.57334	2.23E-05	0.008074	2.576399	PD
cg11991449	0.290601	4.061598	4.572846	2.23E-05	0.008085	2.573101	PD
cg23174475	-0.24042	3.110592	-4.5719	2.24E-05	0.008109	2.572636	PD
cg01124843	-0.21118	-5.02284	-4.57118	2.25E-05	0.008124	2.569133	PD
cg10506607	0.418496	3.137716	4.571142	2.25E-05	0.008124	2.563161	PD
cg25061445	-0.18313	3.5508	-4.57056	2.25E-05	0.008138	2.566558	PD
cg12527909	-0.26992	3.149322	-4.57044	2.25E-05	0.008138	2.567637	PD
cg01733217	-0.48209	-5.72755	-4.56802	2.27E-05	0.008205	2.558528	PD
cg14344539	-0.27659	4.071416	-4.56723	2.28E-05	0.008225	2.555867	PD
cg02938066	1.952817	4.186325	4.566464	2.28E-05	0.008243	2.547353	PD
cg00013255	-0.24933	3.326531	-4.5664	2.28E-05	0.008243	2.547269	PD
cg00038857	-0.16931	3.395063	-4.56586	2.29E-05	0.008255	2.55201	PD
cg10439651	-0.31443	-4.06517	-4.56556	2.29E-05	0.008261	2.542939	PD
cg22100563	-0.27591	3.103547	-4.56508	2.30E-05	0.008271	2.548896	PD
cg19767249	-0.47074	-4.25734	-4.56421	2.30E-05	0.008294	2.542927	PD
cg12403142	-0.24702	5.029807	-4.56319	2.31E-05	0.008321	2.538547	PD

cg21025681	-0.25966	2.975769	-4.56264	2.32E-05	0.008333	2.537545	PD
cg19786799	0.34805	4.556673	4.562511	2.32E-05	0.008333	2.540717	PD
cg11705432	-0.24384	4.1681	-4.56236	2.32E-05	0.008333	2.541313	PD
cg27594583	0.204271	-4.03188	4.562325	2.32E-05	0.008333	2.538649	PD
cg18455772	-0.32387	3.869364	-4.56211	2.32E-05	0.008336	2.539586	PD
cg27207358	0.395135	4.47237	4.561931	2.32E-05	0.008337	2.542074	PD
cg23257697	-0.29021	3.428309	-4.56165	2.32E-05	0.008342	2.537389	PD
cg10317450	0.498438	2.741856	4.560863	2.33E-05	0.008363	2.529722	PD
cg17876831	-0.27617	3.108725	-4.56034	2.33E-05	0.008375	2.532533	PD
cg17026391	-0.16242	3.15691	-4.56018	2.34E-05	0.008376	2.532814	PD
cg22304239	-0.34508	3.06605	-4.55634	2.37E-05	0.008489	2.521648	PD
cg08711521	0.200805	-1.09713	4.555981	2.37E-05	0.008496	2.517259	PD
cg14220830	-0.24209	4.189513	-4.55237	2.40E-05	0.008604	2.502219	PD
cg04864648	-0.14177	4.516267	-4.55153	2.41E-05	0.008625	2.499815	PD
cg05799317	-0.19029	3.987686	-4.55136	2.41E-05	0.008625	2.503444	PD
cg03929761	-0.19597	3.927464	-4.55134	2.41E-05	0.008625	2.505788	PD
cg03678860	-0.25493	3.572237	-4.55076	2.42E-05	0.008639	2.502195	PD
cg02950621	-0.79112	6.305241	-4.54914	2.43E-05	0.008686	2.49409	PD
cg21173402	-0.38279	3.682433	-4.54859	2.44E-05	0.008699	2.496762	PD
cg06644483	0.258993	2.632304	4.548184	2.44E-05	0.008708	2.489492	PD
cg10679182	-0.27948	3.546428	-4.54701	2.45E-05	0.008742	2.486964	PD
cg07288310	0.475049	-3.39122	4.545203	2.47E-05	0.008795	2.48657	PD
cg03575974	-0.1617	4.148015	-4.54501	2.47E-05	0.008797	2.480694	PD
cg19404209	-0.21943	2.636542	-4.5447	2.47E-05	0.008803	2.483383	PD
cg24828864	-0.30493	3.115219	-4.54305	2.48E-05	0.008852	2.477038	PD
cg10267796	-0.19136	2.978058	-4.54274	2.49E-05	0.008858	2.471446	PD
cg03075631	-0.29118	3.635027	-4.54224	2.49E-05	0.00887	2.451839	PD
cg04999756	-1.18949	3.513051	-4.54213	2.49E-05	0.00887	2.469413	PD
cg13728299	0.314438	3.429137	4.541949	2.49E-05	0.008871	2.468141	PD
cg18226052	-0.29771	4.107854	-4.54187	2.50E-05	0.008871	2.471955	PD
cg17550721	-0.2824	2.978162	-4.54163	2.50E-05	0.008875	2.470449	PD

cg18955850	0.278503	3.427317	4.541216	2.50E-05	0.008883	2.470504	PD
cg12049093	0.345272	4.545518	4.541145	2.50E-05	0.008883	2.471042	PD
cg12175311	0.516935	4.016056	4.540543	2.51E-05	0.008898	2.462442	PD
cg00040322	-0.28141	4.16118	-4.54043	2.51E-05	0.008898	2.467345	PD
cg08062613	-0.19658	2.589368	-4.53994	2.51E-05	0.00891	2.467169	PD
cg08549011	0.252405	4.154115	4.53956	2.52E-05	0.008917	2.464862	PD
cg13114315	-0.27012	3.321194	-4.5394	2.52E-05	0.008917	2.466373	PD
cg10122103	-0.18681	4.212197	-4.53938	2.52E-05	0.008917	2.46725	PD
cg02599615	-0.26717	2.67685	-4.53893	2.52E-05	0.008927	2.455428	PD
cg15337538	-0.24551	3.794936	-4.53869	2.52E-05	0.008928	2.454393	PD
cg02631684	-0.23206	3.613857	-4.53867	2.52E-05	0.008928	2.465553	PD
cg07217846	-0.43821	3.234073	-4.53846	2.53E-05	0.008931	2.462202	PD
cg08686426	0.327245	1.684774	4.537463	2.53E-05	0.008957	2.445329	PD
cg17385247	-0.19656	1.911363	-4.53741	2.54E-05	0.008957	2.45527	PD
cg17682357	0.369665	3.63236	4.537261	2.54E-05	0.008957	2.4587	PD
cg01682370	-0.72255	3.243291	-4.53709	2.54E-05	0.008957	2.431097	PD
cg01066290	-0.56175	2.946998	-4.53708	2.54E-05	0.008957	2.440646	PD
cg11678027	-0.50721	-5.44433	-4.53537	2.55E-05	0.009008	2.438179	PD
cg02606218	0.841599	-4.68604	4.534815	2.56E-05	0.009023	2.449163	PD
cg14037346	-0.27802	3.604075	-4.53456	2.56E-05	0.009025	2.448849	PD
cg20133092	-0.20218	3.189463	-4.5345	2.56E-05	0.009025	2.449592	PD
cg08460590	-0.31448	3.506556	-4.53406	2.57E-05	0.009036	2.444152	PD
cg14883135	-1.47419	3.236131	-4.53179	2.59E-05	0.009106	2.409217	PD
cg16508168	0.170617	-3.18907	4.530702	2.60E-05	0.009138	2.426086	PD
cg15913742	-0.32588	2.569728	-4.52909	2.61E-05	0.009187	2.432642	PD
cg09604238	-0.23525	3.214749	-4.52854	2.62E-05	0.009201	2.430715	PD
cg14159526	0.487443	4.313575	4.528442	2.62E-05	0.009201	2.426587	PD
cg05906530	-0.23882	4.050281	-4.5281	2.62E-05	0.009208	2.425956	PD
cg27237477	-0.17894	3.338864	-4.52544	2.65E-05	0.009292	2.420461	PD
cg00642042	-0.19333	3.017303	-4.52522	2.65E-05	0.009296	2.421973	PD
cg20098758	-0.22924	3.508096	-4.52455	2.66E-05	0.009315	2.419456	PD

cg23653667	-0.17121	4.355089	-4.52324	2.67E-05	0.009354	2.408659	PD
cg18229531	-0.30972	3.608374	-4.52233	2.68E-05	0.009381	2.405542	PD
cg27478030	-0.20248	3.23069	-4.52217	2.68E-05	0.009383	2.408718	PD
cg14450695	-0.48696	3.195405	-4.5211	2.69E-05	0.009415	2.398194	PD
cg26719170	1.854396	-6.54551	4.520946	2.69E-05	0.009416	2.408257	PD
cg07860222	-0.25831	3.764394	-4.5208	2.69E-05	0.009417	2.401386	PD
cg15512458	0.219531	2.835945	4.520586	2.69E-05	0.009421	2.404211	PD
cg03219514	0.3532	4.062957	4.518838	2.71E-05	0.009476	2.401054	PD
cg17029655	0.475455	2.424058	4.518729	2.71E-05	0.009476	2.39577	PD
cg12329407	-0.16504	3.69513	-4.5184	2.71E-05	0.009483	2.399635	PD
cg15579522	-0.20769	3.397389	-4.51816	2.72E-05	0.009487	2.393333	PD
cg02587004	-0.50063	3.897276	-4.51806	2.72E-05	0.009487	2.398938	PD
cg21218567	-0.14391	3.214733	-4.51795	2.72E-05	0.009487	2.391527	PD
cg07238429	-0.16945	3.975577	-4.51776	2.72E-05	0.009489	2.393357	PD
cg03004986	-0.26126	3.35425	-4.51703	2.73E-05	0.00951	2.393093	PD
cg03902462	-0.21284	2.849682	-4.51653	2.73E-05	0.00952	2.391364	PD
cg11501236	-0.30609	-3.42464	-4.51652	2.73E-05	0.00952	2.392705	PD
cg17259298	0.679523	-4.64408	4.516054	2.74E-05	0.009531	2.364588	PD
cg21665557	-0.39375	6.049653	-4.51537	2.74E-05	0.00955	2.390106	PD
cg15331717	-0.23149	4.104753	-4.51528	2.74E-05	0.00955	2.384118	PD
cg14419720	-0.2159	3.279119	-4.51511	2.75E-05	0.009552	2.385601	PD
cg04181991	1.037415	3.52165	4.514458	2.75E-05	0.00957	2.37372	PD
cg08323260	0.204209	3.093855	4.513354	2.76E-05	0.009604	2.378439	PD
cg05011276	0.295506	-5.02006	4.512141	2.78E-05	0.009642	2.377369	PD
cg23528206	-0.24264	3.098125	-4.51201	2.78E-05	0.009643	2.37492	PD
cg10636442	0.188787	2.951577	4.509988	2.80E-05	0.009709	2.363618	PD
cg24813562	0.348581	-4.58533	4.509738	2.80E-05	0.009714	2.369632	PD
cg03517614	0.178919	4.114898	4.509448	2.80E-05	0.009719	2.369984	PD
cg11247887	-0.28146	3.969947	-4.50936	2.80E-05	0.009719	2.365545	PD
cg07409153	-0.24334	3.931249	-4.5091	2.81E-05	0.009724	2.367872	PD
cg08651205	-0.28542	2.991235	-4.50815	2.82E-05	0.009753	2.362931	PD

cg15241587	-0.21579	3.340992	-4.50781	2.82E-05	0.009761	2.362908	PD
cg25362585	-0.16192	3.932629	-4.50714	2.83E-05	0.00978	2.345556	PD
cg27136847	-0.37172	3.554067	-4.50638	2.83E-05	0.009803	2.353696	PD
cg04237250	0.150656	-0.98298	4.50576	2.84E-05	0.009821	2.353546	PD
cg01195135	-0.22666	3.267157	-4.50453	2.85E-05	0.00986	2.351952	PD
cg11813940	-0.15064	3.984725	-4.50411	2.86E-05	0.009871	2.351477	PD
cg16869625	-0.35166	2.686449	-4.50354	2.86E-05	0.009887	2.349595	PD
cg04245870	-0.92679	3.191255	-4.50289	2.87E-05	0.009906	2.340282	PD
cg23660064	-0.43386	5.7486	-4.5023	2.88E-05	0.009923	2.346392	PD
cg13491343	-0.30389	3.882163	-4.50164	2.88E-05	0.009942	2.344779	PD
cg24771017	-0.11531	5.051154	-4.50103	2.89E-05	0.00996	2.339422	PD
cg04578890	0.504815	3.089413	4.500265	2.90E-05	0.009983	2.309983	PD
cg15519913	0.254304	2.187725	4.499424	2.91E-05	0.010009	2.334859	PD
cg23418075	0.597162	-3.78292	4.498266	2.92E-05	0.010047	2.320052	PD
cg07771150	-0.32492	3.248204	-4.49787	2.92E-05	0.010057	2.332208	PD
cg12617546	-0.41582	3.062662	-4.49726	2.93E-05	0.010074	2.33082	PD
cg26557162	-0.24713	3.400897	-4.49657	2.94E-05	0.01009	2.323242	PD
cg23127183	0.787771	-4.51174	4.49657	2.94E-05	0.01009	2.326268	PD
cg18512205	-0.23746	3.452312	-4.49651	2.94E-05	0.01009	2.327126	PD
cg22523619	-0.30727	3.137892	-4.49613	2.94E-05	0.010099	2.324509	PD
cg07981590	-0.17395	3.851516	-4.49562	2.95E-05	0.010113	2.322094	PD
cg15551687	-0.1734	4.45753	-4.4954	2.95E-05	0.010117	2.322555	PD
cg11417667	0.261401	4.217997	4.495047	2.95E-05	0.010126	2.318588	PD
cg17830280	-0.40062	2.316118	-4.4948	2.95E-05	0.010131	2.316384	PD
cg13533335	-0.22821	3.15589	-4.49434	2.96E-05	0.010143	2.31972	PD
cg16813187	0.468019	3.464025	4.493455	2.97E-05	0.010169	2.314846	PD
cg17736982	-0.31616	2.741258	-4.49341	2.97E-05	0.010169	2.316482	PD
cg03096957	-0.25619	4.025201	-4.49323	2.97E-05	0.010171	2.315421	PD
cg00604009	-0.18332	3.264969	-4.49275	2.98E-05	0.010185	2.306074	PD
cg25493764	-0.34474	4.962245	-4.49244	2.98E-05	0.010192	2.313003	PD
cg08190109	-0.22347	3.554453	-4.49181	2.99E-05	0.010211	2.311623	PD

cg14774511	-0.4387	6.126228	-4.49164	2.99E-05	0.010211	2.312367	PD
cg24836822	-0.12762	4.577415	-4.49157	2.99E-05	0.010211	2.30953	PD
cg02850401	-0.24123	3.3758	-4.4907	3.00E-05	0.010235	2.308647	PD
cg26576978	0.576389	3.707358	4.490681	3.00E-05	0.010235	2.285842	PD
cg10130155	-0.1996	3.626871	-4.49043	3.00E-05	0.01024	2.308276	PD
cg06059755	0.20637	-4.5089	4.489213	3.01E-05	0.010281	2.302222	PD
cg27548450	-0.22976	4.512625	-4.48845	3.02E-05	0.010305	2.299672	PD
cg05288324	-0.20461	2.702476	-4.48747	3.03E-05	0.010333	2.296334	PD
cg17047033	-0.17968	3.540245	-4.48747	3.03E-05	0.010333	2.295638	PD
cg11344572	0.435597	4.63763	4.487358	3.03E-05	0.010333	2.299367	PD
cg17760120	0.291348	4.167459	4.48712	3.04E-05	0.010333	2.297246	PD
cg01006048	0.497686	3.186664	4.487091	3.04E-05	0.010333	2.285707	PD
cg11410473	0.552214	4.747362	4.486998	3.04E-05	0.010333	2.295421	PD
cg20442816	-0.28673	2.84197	-4.48659	3.04E-05	0.010344	2.293782	PD
cg26075639	0.580632	4.020778	4.486459	3.04E-05	0.010345	2.288921	PD
cg23062771	-0.23212	3.88621	-4.48621	3.05E-05	0.010349	2.287751	PD
cg10296410	-0.36931	-3.78489	-4.48614	3.05E-05	0.010349	2.291437	PD
cg01335210	-0.27427	2.768292	-4.48524	3.06E-05	0.010375	2.289913	PD
cg08305552	-0.15397	3.3579	-4.48519	3.06E-05	0.010375	2.288438	PD
cg09267498	-0.25373	4.313336	-4.48488	3.06E-05	0.010383	2.284828	PD
cg03760658	-0.22928	3.319944	-4.48354	3.08E-05	0.010428	2.286613	PD
cg07334487	0.250082	3.481059	4.482715	3.08E-05	0.010455	2.283188	PD
cg11372447	-0.18588	2.841994	-4.48129	3.10E-05	0.010504	2.277984	PD
cg08100000	-0.25101	0.787734	-4.4805	3.11E-05	0.01053	2.270244	PD
cg03760138	0.348879	3.444186	4.479951	3.11E-05	0.010546	2.272125	PD
cg01647917	1.004182	4.936194	4.479822	3.12E-05	0.010547	2.270239	PD
cg05624234	-0.23662	4.122008	-4.4797	3.12E-05	0.010547	2.269044	PD
cg19010949	0.2677	3.322799	4.47842	3.13E-05	0.010591	2.26911	PD
cg02250535	-0.30177	3.467043	-4.47828	3.13E-05	0.010592	2.26857	PD
cg14567877	-0.41019	2.78507	-4.47691	3.15E-05	0.01064	2.263282	PD
cg17823561	-0.29198	2.699382	-4.47659	3.15E-05	0.010648	2.261931	PD

cg16013223	-0.24727	4.198104	-4.47637	3.15E-05	0.010652	2.26287	PD
cg10647191	0.28011	4.179782	4.476262	3.16E-05	0.010652	2.25785	PD
cg24785250	-0.24263	3.23357	-4.47592	3.16E-05	0.01066	2.260119	PD
cg20575936	-0.3908	2.525995	-4.47517	3.17E-05	0.010685	2.258229	PD
cg13202845	0.422346	-5.93095	4.474612	3.17E-05	0.010702	2.251392	PD
cg09229106	-0.19284	2.836247	-4.47305	3.19E-05	0.010757	2.249137	PD
cg12806067	-0.15058	4.943247	-4.47253	3.20E-05	0.010773	2.250015	PD
cg16865475	-0.16619	4.624417	-4.4723	3.20E-05	0.010775	2.243625	PD
cg14827527	0.201047	-4.06407	4.472248	3.20E-05	0.010775	2.235525	PD
cg08572407	-0.19102	-4.92607	-4.47175	3.21E-05	0.01079	2.246458	PD
cg02063485	-0.20733	4.377394	-4.47071	3.22E-05	0.01082	2.24349	PD
cg15921757	0.226003	-2.28504	4.470614	3.22E-05	0.01082	2.241775	PD
cg00364778	-0.25385	-5.90111	-4.47055	3.22E-05	0.01082	2.244165	PD
cg25707945	-0.20794	3.494561	-4.47054	3.22E-05	0.01082	2.239554	PD
cg03818992	0.259841	-4.86601	4.470118	3.23E-05	0.010829	2.241564	PD
cg06655030	-0.13116	4.76197	-4.47005	3.23E-05	0.010829	2.241288	PD
cg25217842	-0.23236	3.079979	-4.46995	3.23E-05	0.010829	2.240992	PD
cg23811942	-0.27534	0.783536	-4.46939	3.23E-05	0.010844	2.236708	PD
cg27009285	-0.41115	3.238447	-4.46932	3.24E-05	0.010844	2.2365	PD
cg26930498	-0.3987	3.415434	-4.46925	3.24E-05	0.010844	2.236355	PD
cg11705053	-0.37149	2.93436	-4.46907	3.24E-05	0.010846	2.226952	PD
cg24904436	2.576126	4.053248	4.468395	3.25E-05	0.010868	2.234016	PD
cg22106375	0.328002	4.107729	4.468236	3.25E-05	0.01087	2.234157	PD
cg21404476	-0.17841	3.407554	-4.46794	3.25E-05	0.010877	2.228982	PD
cg17727125	-0.32701	3.333844	-4.46743	3.26E-05	0.01089	2.233194	PD
cg10287611	-0.21756	3.201217	-4.46738	3.26E-05	0.01089	2.229399	PD
cg23205818	-0.18164	4.49069	-4.46711	3.26E-05	0.010896	2.233407	PD
cg20654471	0.256793	3.631792	4.466619	3.27E-05	0.010911	2.229882	PD
cg09822356	0.375216	3.284197	4.465655	3.28E-05	0.010945	2.22223	PD
cg06869965	-1.57082	-4.47667	-4.46487	3.29E-05	0.010971	2.2248	PD
cg17719126	-0.24401	3.862703	-4.46451	3.29E-05	0.010981	2.224433	PD

cg10708271	-0.20443	2.782117	-4.46425	3.29E-05	0.010986	2.218156	PD
cg12664780	-0.4769	3.891675	-4.4639	3.30E-05	0.010996	2.219242	PD
cg08791293	-0.30994	4.296103	-4.46366	3.30E-05	0.011001	2.219455	PD
cg03191565	0.177027	-6.09832	4.463428	3.30E-05	0.011002	2.218381	PD
cg03434853	-0.25983	4.123915	-4.46341	3.30E-05	0.011002	2.215675	PD
cg25897896	-0.16198	3.420163	-4.46328	3.31E-05	0.011003	2.21763	PD
cg08119506	-0.24399	2.153127	-4.46315	3.31E-05	0.011003	2.219295	PD
cg03314158	-0.2287	-5.18847	-4.46304	3.31E-05	0.011003	2.218762	PD
cg14225483	0.253785	4.385957	4.461447	3.33E-05	0.011062	2.213817	PD
cg14635269	0.348164	-1.40779	4.46132	3.33E-05	0.011063	2.206033	PD
cg19411922	-0.2524	3.207403	-4.46096	3.33E-05	0.011073	2.21255	PD
cg08797772	-0.25322	3.484742	-4.46057	3.34E-05	0.011083	2.211047	PD
cg04528961	0.284628	-2.87661	4.459715	3.35E-05	0.011113	2.206281	PD
cg20219943	-0.20324	3.046902	-4.45929	3.35E-05	0.011123	2.200736	PD
cg04992974	-0.18286	5.001428	-4.45923	3.35E-05	0.011123	2.20614	PD
cg02269574	-0.25621	1.875789	-4.45913	3.35E-05	0.011123	2.205214	PD
cg16165759	-0.37292	4.049977	-4.45843	3.36E-05	0.011146	2.205064	PD
cg00507855	0.44525	2.894943	4.457205	3.38E-05	0.011191	2.194183	PD
cg16821977	-0.30454	4.002056	-4.45694	3.38E-05	0.011197	2.200239	PD
cg24617647	-0.16996	3.211634	-4.45679	3.38E-05	0.011198	2.195769	PD
cg01947277	-0.30911	4.015474	-4.45626	3.39E-05	0.011215	2.196991	PD
cg04404956	-0.24984	3.480066	-4.45572	3.40E-05	0.011232	2.194464	PD
cg09973371	-0.39713	4.508952	-4.45442	3.41E-05	0.01128	2.190726	PD
cg02494004	0.292883	3.871744	4.454103	3.42E-05	0.011289	2.188209	PD
cg04385200	0.240032	3.195063	4.4533	3.43E-05	0.011317	2.178236	PD
cg19081470	-0.25935	2.512288	-4.4526	3.43E-05	0.01134	2.185079	PD
cg11200248	0.22916	1.879291	4.452235	3.44E-05	0.011351	2.182355	PD
cg23079911	0.611784	3.600421	4.451131	3.45E-05	0.011391	2.156018	PD
cg07897132	-0.21711	3.512655	-4.45072	3.46E-05	0.011403	2.176546	PD
cg09871679	-0.25876	3.354622	-4.45015	3.46E-05	0.011422	2.177417	PD
cg05443751	-0.22326	4.045898	-4.44836	3.49E-05	0.011489	2.172461	PD

cg09519972	-0.16165	4.051944	-4.44828	3.49E-05	0.011489	2.169928	PD
cg11599469	-0.175	3.84232	-4.44753	3.50E-05	0.011515	2.16965	PD
cg07349805	-0.4177	4.667833	-4.44742	3.50E-05	0.011515	2.168275	PD
cg20214135	-0.28533	4.081585	-4.44724	3.50E-05	0.011518	2.1686	PD
cg15274171	-0.1619	2.076815	-4.44646	3.51E-05	0.011546	2.162159	PD
cg00935234	-0.2557	3.736893	-4.44614	3.51E-05	0.011554	2.16284	PD
cg08429705	-2.52675	3.153538	-4.44535	3.52E-05	0.011582	2.162652	PD
cg26385993	-0.19608	4.133269	-4.44458	3.53E-05	0.01161	2.158839	PD
cg00684101	-0.1453	2.941648	-4.44396	3.54E-05	0.011631	2.157929	PD
cg11354576	0.13294	-4.43062	4.442954	3.55E-05	0.011668	2.15386	PD
cg00292073	-0.16626	3.975987	-4.44262	3.56E-05	0.011678	2.153819	PD
cg09929430	-0.24474	4.126022	-4.44245	3.56E-05	0.01168	2.150874	PD
cg14632926	-0.17975	3.10506	-4.44228	3.56E-05	0.011683	2.149771	PD
cg11702172	-0.35878	4.50659	-4.44158	3.57E-05	0.011707	2.149052	PD
cg23973576	-0.16209	3.657274	-4.44068	3.58E-05	0.01174	2.147274	PD
cg16018729	-0.35962	2.68847	-4.43927	3.60E-05	0.011795	2.138947	PD
cg10045206	-0.275	3.840424	-4.439	3.60E-05	0.011801	2.14263	PD
cg26292575	-0.30575	4.154372	-4.43879	3.61E-05	0.011805	2.140094	PD
cg00581720	-0.27134	3.871374	-4.43813	3.62E-05	0.011829	2.136975	PD
cg04671526	-0.15855	3.987618	-4.43793	3.62E-05	0.011832	2.137985	PD
cg14586570	0.260596	4.090933	4.437837	3.62E-05	0.011832	2.118938	PD
cg11863674	0.199427	0.374967	4.437154	3.63E-05	0.011856	2.136092	PD
cg18099070	-0.19078	3.918847	-4.43683	3.63E-05	0.011865	2.134428	PD
cg05681957	-0.15203	3.033431	-4.4366	3.63E-05	0.011867	2.134662	PD
cg23662947	-0.17701	4.328513	-4.43657	3.64E-05	0.011867	2.133863	PD
cg17912835	0.345874	-3.49244	4.436111	3.64E-05	0.011879	2.131247	PD
cg18397726	-0.38442	3.27007	-4.43608	3.64E-05	0.011879	2.130044	PD
cg13109115	-0.25548	3.484564	-4.43588	3.64E-05	0.011882	2.129077	PD
cg10970751	-0.19092	2.22699	-4.43532	3.65E-05	0.011901	2.129022	PD
cg09083178	-0.26073	3.380152	-4.43523	3.65E-05	0.011901	2.127884	PD
cg19708055	-0.18746	4.560602	-4.43396	3.67E-05	0.011947	2.121139	PD

cg23264682	-0.28624	4.691374	-4.43387	3.67E-05	0.011947	2.12183	PD
cg04069099	0.147433	-4.08902	4.433823	3.67E-05	0.011947	2.124687	PD
cg05016408	-0.44016	-4.61276	-4.43361	3.67E-05	0.011951	2.124095	PD
cg16639417	-0.15868	3.767999	-4.43344	3.68E-05	0.011954	2.124254	PD
cg27073218	-0.25881	3.331841	-4.43315	3.68E-05	0.011961	2.121063	PD
cg03902989	-0.23335	2.547767	-4.43304	3.68E-05	0.011961	2.122679	PD
cg00802613	-0.3026	3.669124	-4.43267	3.69E-05	0.011972	2.118504	PD
cg23697575	-0.22012	3.718612	-4.43255	3.69E-05	0.011973	2.116763	PD
cg00359846	-0.20754	2.450079	-4.43216	3.69E-05	0.011985	2.117976	PD
cg14467771	0.445857	4.629706	4.431817	3.70E-05	0.011993	2.11796	PD
cg05774868	-0.25502	3.694113	-4.43177	3.70E-05	0.011993	2.119257	PD
cg02717656	-0.24958	4.048143	-4.4312	3.71E-05	0.012012	2.11562	PD
cg12485320	-0.22655	3.40015	-4.43088	3.71E-05	0.012019	2.11366	PD
cg00523683	-0.36352	2.617851	-4.43083	3.71E-05	0.012019	2.114836	PD
cg16870958	-0.31164	4.005343	-4.43061	3.71E-05	0.012024	2.114606	PD
cg24057218	-0.21224	3.157083	-4.42769	3.75E-05	0.012144	2.104752	PD
cg26235434	-0.111	4.722211	-4.42624	3.77E-05	0.012202	2.100478	PD
cg04165024	-0.38272	4.577642	-4.426	3.77E-05	0.012208	2.094897	PD
cg10693587	-0.30272	0.017552	-4.42531	3.78E-05	0.012234	2.097105	PD
cg05171452	-0.27295	4.136868	-4.42476	3.79E-05	0.012253	2.090958	PD
cg06158184	-0.28484	3.656349	-4.4243	3.80E-05	0.012265	2.095187	PD
cg13445664	-0.25543	3.441798	-4.42427	3.80E-05	0.012265	2.094251	PD
cg19622269	-0.24238	3.400053	-4.42337	3.81E-05	0.012299	2.08966	PD
cg03484139	0.335844	4.163124	4.423004	3.81E-05	0.012306	2.089682	PD
cg19379406	0.22679	3.294763	4.422993	3.82E-05	0.012306	2.091166	PD
cg09045429	-0.43419	3.03247	-4.42287	3.82E-05	0.012307	2.062065	PD
cg10608783	-0.3574	3.933167	-4.42261	3.82E-05	0.012314	2.086983	PD
cg16717988	-0.24738	2.220868	-4.42228	3.82E-05	0.012324	2.086709	PD
cg08793658	-0.18179	3.79701	-4.4216	3.83E-05	0.012348	2.083794	PD
cg25032030	0.510164	-6.19693	4.421422	3.84E-05	0.012351	2.086004	PD
cg14080798	0.340735	3.402612	4.420881	3.84E-05	0.012366	2.072481	PD

cg18633154	0.197659	-1.7469	4.42087	3.84E-05	0.012366	2.083286	PD
cg05839347	-0.21391	3.561667	-4.42063	3.85E-05	0.012372	2.082639	PD
cg02207286	-0.24042	2.84497	-4.42041	3.85E-05	0.012377	2.080575	PD
cg09341027	-0.25364	4.109338	-4.42025	3.85E-05	0.012379	2.08213	PD
cg10011113	-0.29339	2.829448	-4.41967	3.86E-05	0.0124	2.080298	PD
cg10832800	0.385126	4.058863	4.419469	3.86E-05	0.012404	2.079106	PD
cg22727670	0.26564	3.541292	4.419292	3.87E-05	0.012407	2.079221	PD
cg10057252	-0.23624	-3.13851	-4.41873	3.87E-05	0.012428	2.077536	PD
cg19937577	0.349689	3.737779	4.41814	3.88E-05	0.012449	2.074597	PD
cg01514624	0.244674	4.084758	4.417992	3.88E-05	0.012451	2.075312	PD
cg03819562	-0.18477	3.184958	-4.41786	3.89E-05	0.012452	2.07191	PD
cg22038372	1.038398	3.774412	4.416792	3.90E-05	0.012492	2.0685	PD
cg18640509	-0.4046	-5.07495	-4.41669	3.90E-05	0.012492	2.069953	PD
cg18622977	-0.26426	2.420188	-4.41664	3.90E-05	0.012492	2.067933	PD
cg03023189	0.492628	4.365177	4.416462	3.90E-05	0.012495	2.068198	PD
cg14368529	-0.30412	3.353363	-4.41584	3.91E-05	0.012518	2.06716	PD
cg19179869	0.259697	3.538516	4.41554	3.92E-05	0.012526	2.067113	PD
cg10167888	-0.1717	3.716611	-4.41458	3.93E-05	0.012564	2.062372	PD
cg13972337	-0.27166	3.551751	-4.4144	3.93E-05	0.012568	2.062056	PD
cg09100767	0.280881	2.650591	4.413559	3.94E-05	0.012599	2.059542	PD
cg11605617	0.26246	-6.79056	4.413474	3.95E-05	0.012599	2.057511	PD
cg12037916	-0.2236	2.725002	-4.41338	3.95E-05	0.012599	2.057288	PD
cg27209154	-0.2397	4.218699	-4.41326	3.95E-05	0.012599	2.05912	PD
cg23799292	0.209761	3.232269	4.413066	3.95E-05	0.012603	2.0577	PD
cg06962326	-0.15304	6.071589	-4.41286	3.95E-05	0.012608	2.058024	PD
cg04688238	0.299697	3.891379	4.412581	3.96E-05	0.012615	2.057582	PD
cg04867345	-0.32553	2.82372	-4.4123	3.96E-05	0.012623	2.051479	PD
cg12033075	-0.17671	1.723224	-4.41209	3.97E-05	0.012624	2.055743	PD
cg16109819	0.328077	-5.38963	4.412062	3.97E-05	0.012624	2.054561	PD
cg17453460	0.279712	-2.83595	4.409071	4.01E-05	0.012754	2.045565	PD
cg24460235	-0.1788	2.858479	-4.40839	4.02E-05	0.01278	2.041461	PD

cg13954965	-0.14657	5.026298	-4.40794	4.02E-05	0.012796	2.04071	PD
cg15838760	-0.28955	3.646529	-4.40759	4.03E-05	0.012806	2.041554	PD
cg24614245	0.147149	4.215794	4.407496	4.03E-05	0.012806	2.039621	PD
cg03460027	-0.31621	3.604147	-4.40714	4.04E-05	0.012818	2.039986	PD
cg09207246	-0.1625	3.31038	-4.40619	4.05E-05	0.012856	2.037209	PD
cg02269984	-0.29408	4.22235	-4.40415	4.08E-05	0.012945	2.027347	PD
cg04904300	-0.26768	3.288164	-4.40391	4.08E-05	0.012951	2.02941	PD
cg03492092	0.153275	-1.10523	4.403714	4.09E-05	0.012955	2.029327	PD
cg03764027	0.180977	3.369148	4.403285	4.09E-05	0.01297	2.020603	PD
cg05302426	-0.55495	3.077237	-4.40299	4.10E-05	0.012979	2.024886	PD
cg22413215	0.210034	4.48908	4.402331	4.11E-05	0.013004	2.024511	PD
cg01791669	-0.22523	3.955838	-4.40194	4.11E-05	0.013017	2.020255	PD
cg09167779	-0.22733	3.85978	-4.40088	4.13E-05	0.013061	2.019885	PD
cg24711482	0.326761	2.445295	4.400586	4.13E-05	0.01307	2.016596	PD
cg13797019	-0.16316	1.819412	-4.40014	4.14E-05	0.013086	2.017069	PD
cg07974506	-0.31789	4.155877	-4.39994	4.14E-05	0.01309	2.016549	PD
cg03212272	0.214265	-5.37482	4.39948	4.15E-05	0.013106	2.014655	PD
cg06851844	-0.25769	4.655661	-4.39913	4.15E-05	0.013115	2.01337	PD
cg03644703	-0.18434	3.737536	-4.39909	4.15E-05	0.013115	2.013655	PD
cg19899806	-0.23067	3.442857	-4.3984	4.16E-05	0.013142	2.011996	PD
cg12936921	0.581782	3.294002	4.398213	4.17E-05	0.013146	1.989029	PD
cg07207136	-0.30132	3.219732	-4.3973	4.18E-05	0.013183	2.007884	PD
cg14191113	-0.36263	3.89534	-4.39686	4.19E-05	0.013199	2.00728	PD
cg02321536	0.241579	4.061843	4.396435	4.19E-05	0.013214	2.004015	PD
cg08316009	-0.18868	2.379622	-4.39613	4.20E-05	0.013223	2.003634	PD
cg00895174	-0.25904	3.971244	-4.39527	4.21E-05	0.013259	2.001846	PD
cg16017429	-0.15351	4.965513	-4.39469	4.22E-05	0.013281	2.000212	PD
cg07091781	-0.19947	3.609333	-4.39453	4.22E-05	0.013284	1.997095	PD
cg22438763	0.195647	-5.62268	4.394324	4.22E-05	0.013285	1.995762	PD
cg24358762	-0.47345	1.056875	-4.3943	4.22E-05	0.013285	1.98409	PD
cg16478154	-0.21823	3.244454	-4.39415	4.23E-05	0.013286	1.994303	PD

cg20239584	-0.32419	-5.36984	-4.39284	4.25E-05	0.013343	1.986797	PD
cg17509959	0.246445	4.129412	4.392364	4.25E-05	0.013361	1.992114	PD
cg04824469	-0.13252	2.923923	-4.39145	4.27E-05	0.013399	1.985141	PD
cg14285365	-0.1656	3.753315	-4.39075	4.28E-05	0.013425	1.987462	PD
cg01517188	-0.20196	4.325994	-4.3907	4.28E-05	0.013425	1.987514	PD
cg27338738	-0.18854	3.809073	-4.39038	4.28E-05	0.013435	1.984832	PD
cg02401524	-0.29541	3.727561	-4.39025	4.28E-05	0.013436	1.982884	PD
cg03707117	-0.194	3.259935	-4.38998	4.29E-05	0.013444	1.981806	PD
cg06197811	-0.31503	2.92686	-4.3896	4.29E-05	0.013457	1.983922	PD
cg12654250	-0.18155	2.861162	-4.38942	4.30E-05	0.013461	1.982622	PD
cg00685969	-0.15464	3.48406	-4.38919	4.30E-05	0.013467	1.981703	PD
cg15840110	-0.27895	3.274551	-4.38905	4.30E-05	0.013468	1.982363	PD
cg01074797	-0.29717	2.102229	-4.38895	4.30E-05	0.013468	1.974125	PD
cg02421175	-0.35144	2.702311	-4.38832	4.31E-05	0.013493	1.979384	PD
cg24536624	-0.25607	3.56748	-4.38758	4.33E-05	0.013524	1.977119	PD
cg04574991	-0.32501	-6.35458	-4.38746	4.33E-05	0.013525	1.973383	PD
cg20906082	-0.25233	2.542448	-4.38716	4.33E-05	0.013534	1.973101	PD
cg14134417	-0.37975	4.00254	-4.38633	4.34E-05	0.013565	1.970271	PD
cg07294132	-0.17814	4.565572	-4.38623	4.35E-05	0.013565	1.97159	PD
cg03388769	-0.20732	2.585252	-4.3861	4.35E-05	0.013565	1.968096	PD
cg03479065	0.275841	-2.14126	4.386097	4.35E-05	0.013565	1.972475	PD
cg21403580	-0.20426	3.535311	-4.3858	4.35E-05	0.013574	1.971815	PD
cg20545941	-0.12686	1.434545	-4.38535	4.36E-05	0.013591	1.969843	PD
cg23845077	0.287954	4.376117	4.384618	4.37E-05	0.013621	1.965275	PD
cg21787755	0.214125	4.586688	4.384068	4.38E-05	0.013638	1.962577	PD
cg15551993	-0.18868	3.639385	-4.38399	4.38E-05	0.013638	1.965749	PD
cg16991905	0.374797	4.039055	4.383941	4.38E-05	0.013638	1.965889	PD
cg10030633	0.668237	3.002633	4.382636	4.40E-05	0.013696	1.951194	PD
cg10534302	-0.17386	3.119356	-4.38144	4.42E-05	0.01374	1.95722	PD
cg21076170	-0.20615	3.208461	-4.38144	4.42E-05	0.01374	1.957301	PD
cg25009553	0.66998	2.503307	4.381327	4.42E-05	0.01374	1.956065	PD

cg04850714	0.513846	3.942695	4.381326	4.42E-05	0.01374	1.949016	PD
cg25227542	-0.38646	3.906642	-4.38047	4.44E-05	0.013776	1.952773	PD
cg13146913	0.207598	3.587315	4.380078	4.44E-05	0.01379	1.95254	PD
cg03624594	0.18061	-4.2433	4.379809	4.45E-05	0.013798	1.948408	PD
cg22809408	-0.38709	-4.86553	-4.37952	4.45E-05	0.013807	1.951875	PD
cg20367421	0.710826	-5.02865	4.378892	4.46E-05	0.013833	1.94381	PD
cg09123484	0.102193	-4.80081	4.378608	4.46E-05	0.013837	1.945963	PD
cg17023254	-0.22911	3.260987	-4.37861	4.46E-05	0.013837	1.947778	PD
cg17179678	-0.38816	3.199809	-4.37804	4.47E-05	0.01386	1.94605	PD
cg22747746	0.212965	-5.41829	4.377512	4.48E-05	0.013877	1.941864	PD
cg01744944	-0.21695	3.608587	-4.37747	4.48E-05	0.013877	1.944555	PD
cg14361148	-0.23227	3.483282	-4.37673	4.49E-05	0.013908	1.942816	PD
cg17552759	-0.2457	3.042768	-4.37612	4.50E-05	0.013932	1.939177	PD
cg06414921	0.759017	-4.78346	4.376037	4.51E-05	0.013932	1.940098	PD
cg21490597	-0.16561	4.188792	-4.37595	4.51E-05	0.013932	1.940103	PD
cg26379705	-0.27778	3.037547	-4.37564	4.51E-05	0.013942	1.939136	PD
cg05045161	0.466152	-4.80758	4.374192	4.53E-05	0.014008	1.93358	PD
cg04856138	-0.17854	2.561097	-4.37313	4.55E-05	0.014056	1.930122	PD
cg27044023	-0.14456	4.373022	-4.37289	4.56E-05	0.014063	1.929686	PD
cg09001028	-0.25168	2.597605	-4.37215	4.57E-05	0.014094	1.926592	PD
cg11796919	-0.13993	3.788508	-4.37155	4.58E-05	0.014119	1.926029	PD
cg06879777	-0.20531	1.380388	-4.37058	4.59E-05	0.014158	1.921782	PD
cg13361585	-0.23403	2.867211	-4.37046	4.59E-05	0.014158	1.922249	PD
cg22041682	-0.18204	4.264156	-4.37045	4.60E-05	0.014158	1.922726	PD
cg09731745	-0.17633	4.688493	-4.37031	4.60E-05	0.01416	1.922302	PD
cg24890697	-0.30029	3.920202	-4.36901	4.62E-05	0.01422	1.91814	PD
cg24584890	0.107395	-4.54053	4.368608	4.63E-05	0.014235	1.912857	PD
cg19862839	-1.40638	3.81635	-4.36811	4.63E-05	0.014255	1.90773	PD
cg02097890	-0.37523	3.348448	-4.36796	4.64E-05	0.014257	1.897959	PD
cg15216858	-0.17207	4.917779	-4.36768	4.64E-05	0.014266	1.913862	PD
cg16577588	-0.16224	3.570275	-4.36757	4.64E-05	0.014267	1.910457	PD

cg11508013	-0.35643	4.436854	-4.36694	4.65E-05	0.014293	1.90819	PD
cg23612222	-0.19456	3.506285	-4.36607	4.67E-05	0.014332	1.906973	PD
cg16529007	0.240374	3.117888	4.365734	4.67E-05	0.014344	1.90731	PD
cg13153698	-0.21362	3.864178	-4.36563	4.67E-05	0.014344	1.906282	PD
cg27330502	-0.22565	1.944021	-4.36541	4.68E-05	0.01435	1.905943	PD
cg08577693	-0.23853	2.103679	-4.36517	4.68E-05	0.014352	1.90555	PD
cg03018942	-0.42353	3.354159	-4.36516	4.68E-05	0.014352	1.905506	PD
cg17733241	-0.1904	3.707408	-4.36444	4.69E-05	0.014383	1.902927	PD
cg01027820	-0.21713	3.193358	-4.36423	4.70E-05	0.014389	1.902188	PD
cg23853156	-0.16381	3.897	-4.3638	4.70E-05	0.014405	1.898916	PD
cg19418629	-0.18452	3.124415	-4.36328	4.71E-05	0.014426	1.894299	PD
cg17951166	-0.43422	4.275347	-4.36233	4.73E-05	0.01447	1.895734	PD
cg23450399	-0.15708	6.117032	-4.36188	4.74E-05	0.014483	1.894937	PD
cg03673191	-0.17389	3.166531	-4.36186	4.74E-05	0.014483	1.893676	PD
cg26366735	-0.14693	3.439572	-4.36147	4.74E-05	0.014498	1.893317	PD
cg08357257	-0.21029	3.90287	-4.36132	4.75E-05	0.014501	1.892787	PD
cg12011406	-0.13411	3.792926	-4.361	4.75E-05	0.014512	1.891552	PD
cg10206882	0.205266	0.701822	4.360868	4.75E-05	0.014513	1.882951	PD
cg21966754	-0.22856	1.48408	-4.36066	4.76E-05	0.014518	1.889774	PD
cg25935164	-0.2241	2.786356	-4.3602	4.76E-05	0.014537	1.888831	PD
cg06961290	-0.15751	3.46976	-4.35936	4.78E-05	0.01457	1.886235	PD
cg10052667	-0.20528	2.386787	-4.35936	4.78E-05	0.01457	1.885862	PD
cg14822216	0.41723	3.01874	4.359209	4.78E-05	0.014572	1.886866	PD
cg02627335	-0.22049	2.372895	-4.35857	4.79E-05	0.0146	1.884794	PD
cg05985448	-0.19329	4.048128	-4.35809	4.80E-05	0.014619	1.879746	PD
cg02782205	-0.17326	3.891589	-4.35734	4.81E-05	0.014652	1.878223	PD
cg10981717	-0.31841	3.977628	-4.35711	4.82E-05	0.014657	1.879988	PD
cg22807110	0.372892	3.457803	4.357013	4.82E-05	0.014657	1.879773	PD
cg03870162	0.273039	-3.43308	4.356885	4.82E-05	0.014657	1.87889	PD
cg24598637	-0.23379	2.792833	-4.35684	4.82E-05	0.014657	1.878834	PD
cg05350036	-0.21487	2.522418	-4.35661	4.82E-05	0.014664	1.878303	PD

cg07122404	0.181508	2.044628	4.356188	4.83E-05	0.01468	1.877274	PD
cg15244290	0.549922	-3.05455	4.355756	4.84E-05	0.014695	1.873872	PD
cg26423951	-0.33728	3.33977	-4.3557	4.84E-05	0.014695	1.875342	PD
cg16305846	-0.63284	3.235822	-4.355	4.85E-05	0.014726	1.866114	PD
cg24354632	-0.25316	2.795967	-4.35415	4.87E-05	0.014765	1.870382	PD
cg16309518	-0.22351	3.142084	-4.35326	4.88E-05	0.014806	1.866641	PD
cg07591442	-0.24672	3.197816	-4.35283	4.89E-05	0.014823	1.866587	PD
cg25052312	-0.17149	-2.17673	-4.35247	4.90E-05	0.014837	1.86382	PD
cg03698948	0.160823	-5.97362	4.3511	4.92E-05	0.014903	1.861068	PD
cg26912541	-0.17292	4.249988	-4.35094	4.92E-05	0.014906	1.859376	PD
cg03480698	-0.14236	4.103958	-4.35026	4.93E-05	0.014934	1.858393	PD
cg12545044	0.254141	-0.17008	4.350215	4.94E-05	0.014934	1.85793	PD
cg09809224	-0.18673	4.042792	-4.34937	4.95E-05	0.014969	1.854816	PD
cg17049213	-0.29217	2.981964	-4.34934	4.95E-05	0.014969	1.854456	PD
cg05530630	-0.22937	3.514372	-4.34895	4.96E-05	0.014984	1.853979	PD
cg10900049	-0.19556	4.974193	-4.34884	4.96E-05	0.014985	1.85317	PD
cg13184225	0.261094	4.183607	4.348527	4.96E-05	0.014986	1.852104	PD
cg12358759	-0.15767	3.758273	-4.34852	4.96E-05	0.014986	1.852781	PD
cg05299108	-0.26838	2.873124	-4.34852	4.96E-05	0.014986	1.84948	PD
cg20139145	-0.22247	2.79232	-4.348	4.97E-05	0.015002	1.851177	PD
cg13828131	0.304737	2.314508	4.347947	4.97E-05	0.015002	1.850604	PD
cg22951556	-0.19184	3.502319	-4.34791	4.98E-05	0.015002	1.850552	PD
cg14464893	-0.16273	4.1705	-4.34654	5.00E-05	0.015069	1.84577	PD
cg06085456	0.295265	3.759608	4.346389	5.00E-05	0.015072	1.84597	PD
cg04507446	-0.42458	3.965577	-4.34628	5.00E-05	0.015072	1.833106	PD
cg03259038	-0.24624	5.275061	-4.34557	5.02E-05	0.015105	1.840302	PD
cg05324843	-0.22221	2.549299	-4.34424	5.04E-05	0.015159	1.838668	PD
cg16199944	-0.18817	2.941177	-4.34416	5.04E-05	0.015159	1.838162	PD
cg12438155	-0.2576	2.293411	-4.34413	5.04E-05	0.015159	1.837539	PD
cg15170234	0.198735	4.13179	4.344033	5.04E-05	0.015159	1.836445	PD
cg10303967	-0.19356	4.109875	-4.34401	5.04E-05	0.015159	1.834141	PD

cg01124477	-0.30057	2.519604	-4.34394	5.05E-05	0.015159	1.838122	PD
cg14283756	0.57251	-3.37241	4.343732	5.05E-05	0.015165	1.836331	PD
cg01741056	0.348391	1.276858	4.343244	5.06E-05	0.015185	1.818553	PD
cg22859054	-0.13258	-0.51173	-4.34226	5.08E-05	0.015233	1.83211	PD
cg12021706	-0.27259	2.913682	-4.34177	5.08E-05	0.015249	1.831222	PD
cg01713272	0.273481	-2.85313	4.341701	5.09E-05	0.015249	1.831141	PD
cg19961312	-0.16398	3.608219	-4.34166	5.09E-05	0.015249	1.831013	PD
cg23730622	-0.25899	4.436566	-4.34148	5.09E-05	0.015253	1.829555	PD
cg08471835	0.324379	-5.54998	4.341326	5.09E-05	0.015256	1.829238	PD
cg09782621	0.419914	-3.8822	4.340461	5.11E-05	0.015297	1.827067	PD
cg05287104	-0.2206	2.914854	-4.33923	5.13E-05	0.015358	1.821668	PD
cg10946576	-0.24792	2.210667	-4.33903	5.13E-05	0.015364	1.822416	PD
cg06335220	-0.16516	3.480979	-4.33891	5.14E-05	0.015364	1.82209	PD
cg08484668	-0.18028	5.45635	-4.33878	5.14E-05	0.015366	1.819367	PD
cg12849065	0.368199	4.122014	4.338522	5.14E-05	0.015374	1.819597	PD
cg25497742	-0.22838	4.019928	-4.33817	5.15E-05	0.015388	1.819884	PD
cg14112041	0.318287	4.215141	4.337555	5.16E-05	0.015411	1.817875	PD
cg01191058	-0.15763	4.271018	-4.33754	5.16E-05	0.015411	1.817784	PD
cg22686854	0.245508	-4.21789	4.336979	5.17E-05	0.015436	1.815635	PD
cg26664107	-0.17811	3.173255	-4.3365	5.18E-05	0.015457	1.813918	PD
cg25367431	-0.34678	2.708288	-4.3356	5.20E-05	0.0155	1.811726	PD
cg13599021	0.419534	3.025999	4.335283	5.20E-05	0.015512	1.800086	PD
cg02061224	-0.33805	3.66971	-4.33376	5.23E-05	0.01559	1.805855	PD
cg03291732	0.36175	3.362405	4.331268	5.28E-05	0.015722	1.796879	PD
cg23043764	-0.27231	3.443317	-4.33052	5.29E-05	0.015758	1.79163	PD
cg15029699	0.133926	1.273257	4.329759	5.30E-05	0.015794	1.791684	PD
cg17195394	-0.24811	3.576371	-4.32937	5.31E-05	0.015806	1.791504	PD
cg17432898	0.344246	-5.27776	4.329353	5.31E-05	0.015806	1.791213	PD
cg02827731	0.515783	5.07397	4.329064	5.32E-05	0.015816	1.790954	PD
cg12074474	-0.33014	4.054922	-4.32864	5.32E-05	0.015834	1.78876	PD
cg13453244	-0.26919	3.447966	-4.32848	5.33E-05	0.015837	1.787016	PD

cg02326806	-0.36992	-2.5603	-4.32809	5.33E-05	0.015851	1.785336	PD
cg07588442	-0.49221	-5.62015	-4.32793	5.34E-05	0.015851	1.785623	PD
cg13530938	1.832792	-4.3221	4.327932	5.34E-05	0.015851	1.78736	PD
cg08195247	0.365487	-3.88065	4.327672	5.34E-05	0.01586	1.786534	PD
cg05828893	0.463912	-4.38861	4.327291	5.35E-05	0.015876	1.783021	PD
cg04728932	-0.2457	3.440519	-4.32586	5.38E-05	0.01595	1.780059	PD
cg08192683	-0.1357	4.631626	-4.32445	5.40E-05	0.016022	1.771785	PD
cg02653030	0.180726	4.089958	4.324393	5.40E-05	0.016022	1.776058	PD
cg05926478	0.272541	4.008036	4.324016	5.41E-05	0.016035	1.774712	PD
cg02077610	-0.2646	3.624441	-4.32396	5.41E-05	0.016035	1.774736	PD
cg07695058	-0.22239	3.04538	-4.32385	5.41E-05	0.016035	1.755792	PD
cg09243591	-0.1571	3.773857	-4.32354	5.42E-05	0.016047	1.773191	PD
cg11604128	-0.18115	3.224542	-4.3234	5.42E-05	0.01605	1.771032	PD
cg14415312	-0.21602	3.3589	-4.32319	5.43E-05	0.016056	1.772237	PD
cg03809898	-0.35528	2.813332	-4.32288	5.43E-05	0.016062	1.771165	PD
cg15748304	0.162386	4.017701	4.322877	5.43E-05	0.016062	1.771004	PD
cg07505395	-0.17954	3.262436	-4.32254	5.44E-05	0.016076	1.769789	PD
cg12170019	-0.27767	3.342481	-4.32194	5.45E-05	0.016104	1.768332	PD
cg09817814	-0.17516	3.280629	-4.32104	5.47E-05	0.016149	1.764861	PD
cg05921581	-0.24604	4.122586	-4.32046	5.48E-05	0.016176	1.759244	PD
cg14615768	0.093751	-0.75141	4.320095	5.49E-05	0.016192	1.761834	PD
cg07824800	-0.26243	4.353771	-4.31993	5.49E-05	0.016192	1.758292	PD
cg08941853	-0.24281	2.046099	-4.31989	5.49E-05	0.016192	1.761799	PD
cg07455339	-0.2139	3.542871	-4.31871	5.51E-05	0.016254	1.75542	PD
cg05235610	-0.14851	3.96276	-4.31801	5.53E-05	0.016288	1.753222	PD
cg01297265	0.229613	3.135215	4.31729	5.54E-05	0.016323	1.753557	PD
cg14901926	-0.24769	2.469228	-4.31699	5.55E-05	0.016335	1.752621	PD
cg27289137	0.200196	-0.85572	4.316836	5.55E-05	0.016338	1.752147	PD
cg27089000	0.26439	3.235664	4.316257	5.56E-05	0.016366	1.748807	PD
cg12540946	-0.28216	4.116878	-4.31567	5.57E-05	0.016393	1.74768	PD
cg08688086	-0.22286	2.900052	-4.31549	5.58E-05	0.016398	1.747362	PD

cg14498227	-0.23403	3.281078	-4.31501	5.59E-05	0.01642	1.745627	PD
cg03602420	-0.28748	2.922233	-4.31392	5.61E-05	0.016477	1.742884	PD
cg10747758	-0.24379	3.447279	-4.31383	5.61E-05	0.016477	1.741407	PD
cg22282174	0.291065	3.03616	4.3135	5.62E-05	0.01649	1.741395	PD
cg22525129	-0.16952	3.659219	-4.31335	5.62E-05	0.016493	1.739276	PD
cg12234059	-0.21492	2.137236	-4.31309	5.62E-05	0.016503	1.737755	PD
cg21464983	-0.197	3.661345	-4.3123	5.64E-05	0.016543	1.737608	PD
cg20084672	-0.16505	3.833784	-4.31207	5.64E-05	0.01655	1.736903	PD
cg02101279	-0.23491	1.562035	-4.3108	5.67E-05	0.016619	1.727458	PD
cg27159443	-0.26763	2.574127	-4.3101	5.68E-05	0.016652	1.73078	PD
cg05126581	-0.24713	3.576639	-4.31002	5.68E-05	0.016652	1.730497	PD
cg23338468	-0.3755	-5.58612	-4.30984	5.69E-05	0.016657	1.729893	PD
cg21940923	0.511575	-4.12872	4.309538	5.69E-05	0.016669	1.728645	PD
cg13270966	-0.2123	2.66795	-4.30838	5.72E-05	0.016731	1.723132	PD
cg24942990	-0.24973	2.878155	-4.30765	5.73E-05	0.016768	1.721683	PD
cg05364412	-0.19916	5.240647	-4.30735	5.74E-05	0.01678	1.721964	PD
cg22424536	-0.14053	-4.01407	-4.30717	5.74E-05	0.016785	1.721433	PD
cg15866116	-0.74005	3.866866	-4.30673	5.75E-05	0.016805	1.719679	PD
cg10898776	0.406213	2.780036	4.306617	5.75E-05	0.016806	1.717109	PD
cg06777844	-0.46547	2.578252	-4.30648	5.76E-05	0.016808	1.718438	PD
cg16950711	-0.27672	3.764258	-4.30591	5.77E-05	0.016836	1.714762	PD
cg16087684	0.181056	1.609685	4.305744	5.77E-05	0.01684	1.716252	PD
cg06262288	-0.2064	2.919114	-4.30509	5.78E-05	0.016873	1.714775	PD
cg06486935	-0.31121	4.134683	-4.30453	5.79E-05	0.0169	1.713095	PD
cg15671097	-0.18642	3.74345	-4.30431	5.80E-05	0.016906	1.71073	PD
cg10773799	-0.28041	4.029133	-4.30424	5.80E-05	0.016906	1.712239	PD
cg24145369	-0.19672	3.692685	-4.30386	5.81E-05	0.016922	1.710925	PD
cg21585068	-0.21397	1.995912	-4.30277	5.83E-05	0.016982	1.707547	PD
cg27617638	0.167378	-3.95671	4.3023	5.84E-05	0.017001	1.705994	PD
cg01288377	-0.17879	3.090672	-4.30218	5.84E-05	0.017001	1.705558	PD
cg26424468	-0.31615	3.208888	-4.30215	5.84E-05	0.017001	1.705476	PD

cg14890220	-0.14939	2.782228	-4.30171	5.85E-05	0.017021	1.703979	PD
cg22385827	0.183617	-2.66172	4.30107	5.87E-05	0.017053	1.701865	PD
cg10088875	0.352817	-4.39796	4.299916	5.89E-05	0.017117	1.698254	PD
cg07526731	-0.20343	4.256184	-4.29958	5.90E-05	0.017127	1.697277	PD
cg03119748	-0.26616	2.830294	-4.29954	5.90E-05	0.017127	1.69679	PD
cg27326062	-0.30496	2.268709	-4.29905	5.91E-05	0.017151	1.695327	PD
cg17754943	-0.18658	3.684375	-4.29866	5.92E-05	0.017166	1.693431	PD
cg03369465	-0.1975	-4.78143	-4.2986	5.92E-05	0.017166	1.694371	PD
cg01520578	-0.2459	3.08328	-4.29815	5.93E-05	0.017187	1.692995	PD
cg10401356	0.689177	5.586829	4.297798	5.93E-05	0.017203	1.689195	PD
cg12172753	-0.20047	4.288352	-4.29711	5.95E-05	0.017231	1.689673	PD
cg17030173	0.337858	-3.02841	4.297032	5.95E-05	0.017231	1.688332	PD
cg07807575	0.406567	-4.19102	4.29703	5.95E-05	0.017231	1.684673	PD
cg07727134	-0.19563	3.888849	-4.29508	5.99E-05	0.017344	1.683098	PD
cg04795040	-0.15494	3.463863	-4.29439	6.00E-05	0.01738	1.680837	PD
cg09049063	-0.26505	3.61092	-4.2918	6.06E-05	0.017532	1.672012	PD
cg03188793	-0.51058	2.723343	-4.29121	6.07E-05	0.017562	1.670837	PD
cg25970454	-0.20474	3.246691	-4.28978	6.10E-05	0.017642	1.66615	PD
cg00060475	-0.29529	4.023011	-4.28972	6.10E-05	0.017642	1.666361	PD
cg11360931	-0.13306	4.795271	-4.28956	6.11E-05	0.017646	1.66562	PD
cg09177094	-0.19528	2.977529	-4.28934	6.11E-05	0.017649	1.665156	PD
cg01583646	-0.20954	2.052885	-4.28932	6.11E-05	0.017649	1.664915	PD
cg21363515	-0.19795	2.945569	-4.28905	6.12E-05	0.017659	1.664228	PD
cg16535068	-0.23395	2.390423	-4.28836	6.13E-05	0.017696	1.65729	PD
cg03560850	-0.16794	4.318892	-4.28815	6.14E-05	0.017703	1.66133	PD
cg01470419	-0.18189	4.73589	-4.28761	6.15E-05	0.017731	1.658995	PD
cg01176782	-0.23061	4.802136	-4.28629	6.18E-05	0.017807	1.652563	PD
cg22924504	-0.21616	4.061789	-4.28605	6.18E-05	0.017815	1.654511	PD
cg24538512	-0.22539	4.573992	-4.28578	6.19E-05	0.017826	1.653901	PD
cg16744911	0.112421	-0.5495	4.284583	6.21E-05	0.017895	1.649523	PD
cg07191791	-0.28579	3.21451	-4.28402	6.23E-05	0.017921	1.642869	PD

cg16416584	0.663024	3.179683	4.283965	6.23E-05	0.017921	1.641573	PD
cg14020740	-0.20819	3.351093	-4.28355	6.24E-05	0.017941	1.646822	PD
cg04482075	0.672275	6.166475	4.283348	6.24E-05	0.017948	1.64613	PD
cg26882487	-0.23626	2.966608	-4.28299	6.25E-05	0.017954	1.644984	PD
cg25797507	-0.23356	3.575824	-4.28297	6.25E-05	0.017954	1.644977	PD
cg27179498	0.352862	2.588334	4.282898	6.25E-05	0.017954	1.641715	PD
cg04372436	-0.49259	3.177477	-4.28286	6.25E-05	0.017954	1.642183	PD
cg07999547	0.669323	-2.03393	4.282716	6.25E-05	0.017957	1.640033	PD
cg08774513	-0.2414	3.941002	-4.2826	6.26E-05	0.017957	1.642471	PD
cg17619311	0.269851	-5.17249	4.28252	6.26E-05	0.017957	1.64344	PD
cg23392273	-0.23838	3.332992	-4.28189	6.27E-05	0.01799	1.641636	PD
cg02714192	-0.18575	4.293724	-4.28176	6.28E-05	0.01799	1.640539	PD
cg24462263	-0.18506	2.870778	-4.28171	6.28E-05	0.01799	1.64088	PD
cg13811315	-0.26384	3.386945	-4.28157	6.28E-05	0.017992	1.639995	PD
cg05801627	-0.18837	3.614902	-4.28142	6.28E-05	0.017996	1.640057	PD
cg27144719	-0.25677	3.544567	-4.28107	6.29E-05	0.018012	1.638896	PD
cg02429813	0.155142	-5.21012	4.280749	6.30E-05	0.018018	1.634682	PD
cg27116232	-0.17362	3.261135	-4.28069	6.30E-05	0.018018	1.637036	PD
cg26052116	-0.18268	4.110283	-4.28066	6.30E-05	0.018018	1.637728	PD
cg07222745	-0.18425	2.745454	-4.28058	6.30E-05	0.018018	1.637491	PD
cg12174329	0.26218	3.858538	4.279599	6.32E-05	0.018074	1.634085	PD
cg23691406	0.380588	3.441173	4.278125	6.36E-05	0.018161	1.625772	PD
cg19958956	-0.26825	2.193863	-4.27747	6.37E-05	0.018194	1.627482	PD
cg07620571	-0.15206	3.21816	-4.27741	6.37E-05	0.018194	1.624059	PD
cg03054255	0.174894	2.542705	4.277254	6.38E-05	0.018198	1.625753	PD
cg20326580	-0.16853	3.1292	-4.27692	6.38E-05	0.018212	1.625905	PD
cg21467365	-0.2675	3.855935	-4.27685	6.38E-05	0.018212	1.619069	PD
cg04851257	0.238887	3.985914	4.276554	6.39E-05	0.018224	1.621436	PD
cg08765764	-0.15934	3.879366	-4.2762	6.40E-05	0.018241	1.623364	PD
cg26690525	-0.39988	4.349642	-4.2761	6.40E-05	0.018241	1.622072	PD
cg23041109	-0.16436	0.006491	-4.27542	6.42E-05	0.018278	1.621117	PD

cg13682158	-0.21279	2.52451	-4.27497	6.43E-05	0.018296	1.61942	PD
cg26360004	-0.27642	4.525973	-4.27495	6.43E-05	0.018296	1.619487	PD
cg08710088	-0.20517	2.962181	-4.27451	6.44E-05	0.018318	1.617873	PD
cg20271234	0.199914	0.188113	4.274357	6.44E-05	0.018322	1.617825	PD
cg02589878	0.303772	4.230452	4.273638	6.46E-05	0.018362	1.615455	PD
cg24892473	-0.24997	3.813607	-4.27313	6.47E-05	0.018388	1.613645	PD
cg01782410	-0.52718	4.099306	-4.27299	6.47E-05	0.018391	1.602827	PD
cg17080234	-0.33297	4.317383	-4.27285	6.47E-05	0.018394	1.613043	PD
cg08992305	-1.01356	-4.42288	-4.27243	6.48E-05	0.018414	1.61154	PD
cg25453957	-0.24479	4.296476	-4.27219	6.49E-05	0.018418	1.611042	PD
cg15557309	-0.40057	2.620498	-4.27219	6.49E-05	0.018418	1.610997	PD
cg21851666	0.241128	4.54534	4.271574	6.50E-05	0.018451	1.608317	PD
cg21171299	-0.27253	3.511048	-4.27119	6.51E-05	0.018469	1.607824	PD
cg07977152	-0.15178	2.572554	-4.27105	6.52E-05	0.01847	1.607282	PD
cg27147718	-0.13823	4.093991	-4.27099	6.52E-05	0.01847	1.607251	PD
cg18156903	0.272979	4.137378	4.270169	6.54E-05	0.018512	1.604013	PD
cg08216491	-0.27829	2.526911	-4.27015	6.54E-05	0.018512	1.604612	PD
cg20049727	-0.30738	3.610127	-4.26924	6.56E-05	0.018559	1.601555	PD
cg23482839	0.508173	3.488262	4.269229	6.56E-05	0.018559	1.601708	PD
cg13757934	-0.19098	2.210258	-4.2688	6.57E-05	0.018581	1.599869	PD
cg01471923	0.963921	3.303012	4.268085	6.58E-05	0.018621	1.592287	PD
cg07135942	-0.1382	-1.18433	-4.26795	6.59E-05	0.018621	1.597168	PD
cg27085261	-0.29405	3.782338	-4.26787	6.59E-05	0.018621	1.596906	PD
cg19848924	-0.41976	3.587653	-4.26779	6.59E-05	0.018621	1.586322	PD
cg18225364	0.366914	3.337932	4.26765	6.59E-05	0.018624	1.596775	PD
cg04665311	0.480299	3.624743	4.267554	6.60E-05	0.018624	1.593964	PD
cg01382567	-0.57424	4.030499	-4.26721	6.60E-05	0.018641	1.595294	PD
cg05869732	-0.29753	3.574253	-4.26702	6.61E-05	0.018647	1.594581	PD
cg22738440	-0.24297	2.879124	-4.26614	6.63E-05	0.018698	1.591897	PD
cg05307871	-0.2471	4.872581	-4.26603	6.63E-05	0.018699	1.591444	PD
cg18059941	-0.30143	1.397264	-4.26587	6.63E-05	0.018699	1.590811	PD

cg17546605	-0.19766	2.39157	-4.26583	6.63E-05	0.018699	1.589692	PD
cg05164253	0.214444	3.986939	4.265589	6.64E-05	0.018709	1.590248	PD
cg20293433	-0.14494	4.918954	-4.26517	6.65E-05	0.01873	1.588964	PD
cg01305436	-0.30591	3.494553	-4.26475	6.66E-05	0.018752	1.586946	PD
cg06074873	-0.20699	2.236934	-4.2645	6.67E-05	0.018755	1.586878	PD
cg24690092	-0.14293	3.959826	-4.26448	6.67E-05	0.018755	1.586552	PD
cg25252977	0.283557	3.659201	4.264409	6.67E-05	0.018755	1.583696	PD
cg22193691	0.37592	2.526448	4.2643	6.67E-05	0.018756	1.585456	PD
cg24921031	-0.18928	3.680194	-4.26384	6.68E-05	0.01878	1.584766	PD
cg09327046	0.227828	4.227082	4.263267	6.69E-05	0.018802	1.582795	PD
cg11307417	-0.24013	3.048678	-4.26325	6.69E-05	0.018802	1.582677	PD
cg04309025	0.151837	-5.26483	4.263216	6.70E-05	0.018802	1.582501	PD
cg03385530	-0.42668	4.39074	-4.26288	6.70E-05	0.018818	1.581374	PD
cg25983677	-0.63359	-5.30119	-4.26256	6.71E-05	0.018831	1.580579	PD
cg22840232	-0.16859	3.868819	-4.26238	6.72E-05	0.018831	1.580207	PD
cg18368637	-0.18202	3.033753	-4.26237	6.72E-05	0.018831	1.5801	PD
cg11236641	-0.28588	3.995518	-4.2623	6.72E-05	0.018831	1.579963	PD
cg08303483	-0.28202	3.529791	-4.26182	6.73E-05	0.018856	1.578437	PD
cg14680792	-0.20875	2.602657	-4.2615	6.74E-05	0.018871	1.577415	PD
cg17537073	-2.22214	-4.31975	-4.26076	6.75E-05	0.018913	1.574983	PD
cg10982547	-0.37308	3.737388	-4.25993	6.77E-05	0.018962	1.572478	PD
cg21600784	-0.11956	3.596129	-4.25978	6.78E-05	0.018966	1.569379	PD
cg15336645	-0.1695	3.510058	-4.25968	6.78E-05	0.018966	1.571172	PD
cg07657472	0.280632	3.483447	4.259486	6.78E-05	0.018973	1.570474	PD
cg12683085	-0.15272	4.729579	-4.25922	6.79E-05	0.018984	1.569363	PD
cg12046168	0.174169	-6.0862	4.258275	6.81E-05	0.01904	1.567291	PD
cg11829626	-0.17228	4.126336	-4.25809	6.82E-05	0.019046	1.566286	PD
cg24646529	-0.1941	-5.22663	-4.25786	6.82E-05	0.019055	1.565905	PD
cg17678545	0.193029	-4.89715	4.256835	6.85E-05	0.019117	1.558561	PD
cg03294907	-0.29858	4.20816	-4.25668	6.85E-05	0.019121	1.562276	PD
cg15707487	-0.21324	3.612036	-4.25657	6.85E-05	0.019123	1.561885	PD

cg09007239	-0.30544	3.859656	-4.25626	6.86E-05	0.019136	1.560905	PD
cg04741861	-0.38655	5.573814	-4.25568	6.87E-05	0.019169	1.559126	PD
cg11508674	0.155407	-1.59562	4.255489	6.88E-05	0.019176	1.557164	PD
cg25246259	-0.32363	3.075033	-4.25519	6.89E-05	0.019189	1.557561	PD
cg16519574	-0.20772	2.316182	-4.25505	6.89E-05	0.019192	1.554841	PD
cg24216506	-0.17789	3.287794	-4.25484	6.89E-05	0.0192	1.556519	PD
cg03023653	-0.21208	3.166362	-4.25405	6.91E-05	0.019247	1.553914	PD
cg13167860	-0.2249	2.484753	-4.25357	6.92E-05	0.019272	1.552522	PD
cg25382740	-0.26521	4.398135	-4.25307	6.94E-05	0.0193	1.550972	PD
cg03432749	-0.4435	3.211486	-4.25241	6.95E-05	0.019332	1.547638	PD
cg11207705	-0.20476	2.64793	-4.2524	6.95E-05	0.019332	1.548286	PD
cg02162720	-0.24381	2.829358	-4.25039	7.00E-05	0.019456	1.54185	PD
cg07039172	-0.20375	3.015991	-4.25038	7.00E-05	0.019456	1.542319	PD
cg20392585	0.168051	-3.817	4.249699	7.02E-05	0.019496	1.540345	PD
cg02094813	-0.17504	2.683409	-4.24901	7.04E-05	0.019536	1.537717	PD
cg00867594	-0.32105	4.199204	-4.24887	7.04E-05	0.019537	1.537331	PD
cg14150973	0.785599	-4.93213	4.24881	7.04E-05	0.019537	1.537526	PD
cg04315121	-0.31415	2.525275	-4.24802	7.06E-05	0.019585	1.531488	PD
cg18077068	0.298889	3.533163	4.246879	7.09E-05	0.019656	1.530149	PD
cg03119442	-0.15402	4.542667	-4.24654	7.10E-05	0.019673	1.530473	PD
cg02432907	0.37001	2.360583	4.245779	7.12E-05	0.019719	1.527404	PD
cg19751877	0.214957	1.400172	4.245568	7.12E-05	0.019727	1.527348	PD
cg25474235	-0.2824	2.766918	-4.24505	7.13E-05	0.019751	1.52577	PD
cg08533083	-0.19377	2.349435	-4.24499	7.14E-05	0.019751	1.525486	PD
cg23480666	-0.20316	3.793644	-4.24486	7.14E-05	0.019751	1.524698	PD
cg08193925	-0.52788	2.983143	-4.24484	7.14E-05	0.019751	1.524458	PD
cg10696876	-1.67378	-6.0863	-4.24455	7.15E-05	0.01976	1.522824	PD
cg23157239	-0.2096	3.564938	-4.24453	7.15E-05	0.01976	1.524152	PD
cg25474648	-0.2505	3.0444	-4.24355	7.17E-05	0.01982	1.521093	PD
cg00000155	-0.14291	4.237278	-4.24319	7.18E-05	0.019838	1.519677	PD
cg08940546	0.315117	3.859581	4.243106	7.18E-05	0.019838	1.518805	PD

cg01954567	-0.27559	3.802509	-4.24263	7.19E-05	0.019865	1.518195	PD
cg01950479	-0.18323	2.797091	-4.2424	7.20E-05	0.01987	1.517439	PD
cg07477310	-0.23612	3.817653	-4.24237	7.20E-05	0.01987	1.516424	PD
cg27457427	-0.12415	4.002387	-4.24168	7.22E-05	0.019911	1.515198	PD
cg00520305	-0.34586	2.561502	-4.24096	7.24E-05	0.019955	1.512921	PD
cg16145211	-0.12575	3.924429	-4.24083	7.24E-05	0.019957	1.512162	PD
cg15138339	-0.18887	4.106457	-4.24011	7.26E-05	0.02	1.510281	PD
cg12306343	-0.16397	3.880971	-4.23977	7.27E-05	0.020018	1.509121	PD
cg19800069	-0.31414	2.496181	-4.23937	7.28E-05	0.020036	1.508001	PD
cg13476800	-0.21521	3.425388	-4.23927	7.28E-05	0.020036	1.507655	PD
cg22507615	-0.29185	1.545818	-4.23918	7.28E-05	0.020036	1.507295	PD
cg15122343	-0.1658	2.838462	-4.23913	7.28E-05	0.020036	1.50712	PD
cg00445202	-0.36762	3.657489	-4.23898	7.29E-05	0.02004	1.506633	PD
cg12163646	-0.151	3.245666	-4.2383	7.30E-05	0.020081	1.504511	PD
cg14189696	-0.20261	3.095226	-4.23804	7.31E-05	0.020088	1.50355	PD
cg02836767	-0.34315	3.468268	-4.23801	7.31E-05	0.020088	1.503597	PD
cg16987403	-0.21763	2.250781	-4.23787	7.31E-05	0.020091	1.503075	PD
cg26040583	-0.1276	3.697098	-4.23737	7.33E-05	0.020118	1.500575	PD
cg26265657	0.207494	-5.67588	4.23731	7.33E-05	0.020118	1.500106	PD
cg23799720	0.240264	-6.86077	4.237143	7.33E-05	0.020123	1.500705	PD
cg19539732	-0.23903	4.410102	-4.23569	7.37E-05	0.020218	1.496284	PD
cg23674900	-0.21332	1.451321	-4.2353	7.38E-05	0.020239	1.495237	PD
cg15687646	0.26187	4.080991	4.235186	7.38E-05	0.020241	1.494895	PD
cg19818294	-0.21243	3.833554	-4.23508	7.39E-05	0.020241	1.494571	PD
cg09741074	0.104395	-4.32296	4.234914	7.39E-05	0.020247	1.493944	PD
cg08503300	0.411865	3.325651	4.234783	7.39E-05	0.020249	1.493249	PD
cg08487469	-0.18316	3.425798	-4.23439	7.40E-05	0.02027	1.492392	PD
cg15617032	0.173804	-1.01977	4.234267	7.41E-05	0.020273	1.492018	PD
cg04126584	0.291142	3.795333	4.234155	7.41E-05	0.020274	1.490986	PD
cg13844463	0.293977	-2.60211	4.233635	7.42E-05	0.020304	1.489995	PD
cg08212657	0.245592	2.624786	4.232632	7.45E-05	0.020368	1.486814	PD

cg18329052	-0.39894	2.258565	-4.23243	7.45E-05	0.020376	1.486123	PD
cg23335490	0.264375	4.214517	4.231969	7.47E-05	0.020402	1.484379	PD
cg00682427	0.233147	3.572558	4.231655	7.47E-05	0.020418	1.48348	PD
cg25423520	-0.15229	-0.1439	-4.22967	7.53E-05	0.020542	1.477648	PD
cg02224147	-0.22755	3.426572	-4.22967	7.53E-05	0.020542	1.477231	PD
cg01454815	-0.43847	-4.7816	-4.22964	7.53E-05	0.020542	1.477435	PD
cg13971866	-0.30342	3.539466	-4.22783	7.57E-05	0.020665	1.471773	PD
cg26665480	0.733229	-6.05718	4.227577	7.58E-05	0.020676	1.471027	PD
cg07534705	-0.33598	3.027293	-4.22683	7.60E-05	0.020723	1.468711	PD
cg26851650	-0.16621	6.139865	-4.2265	7.61E-05	0.02074	1.467701	PD
cg11115211	-0.2688	-5.44258	-4.22633	7.61E-05	0.020746	1.466334	PD
cg05673830	-0.13852	4.391025	-4.22562	7.63E-05	0.02079	1.464851	PD
cg16860882	-0.26093	2.486256	-4.22542	7.64E-05	0.020799	1.464321	PD
cg04117502	-0.367	3.615033	-4.22502	7.65E-05	0.020821	1.462785	PD
cg09741588	0.277264	-3.83601	4.224575	7.66E-05	0.020846	1.4605	PD
cg08825582	-0.26736	4.717792	-4.22365	7.68E-05	0.020903	1.457906	PD
cg21124175	-0.25758	3.228431	-4.2236	7.69E-05	0.020903	1.458556	PD
cg11664379	-0.19884	0.533317	-4.22275	7.71E-05	0.020958	1.456003	PD
cg05377733	-0.22208	2.855076	-4.22251	7.72E-05	0.020966	1.455144	PD
cg27665181	0.159173	-5.26271	4.222454	7.72E-05	0.020966	1.455084	PD
cg24968869	-0.22458	4.02915	-4.22218	7.72E-05	0.02098	1.454214	PD
cg10051493	-1.10562	3.01077	-4.22185	7.73E-05	0.020995	1.448988	PD
cg14690181	-0.18825	0.009221	-4.22179	7.73E-05	0.020995	1.452308	PD
cg15746449	0.199039	-3.19489	4.221506	7.74E-05	0.021009	1.452102	PD
cg15763823	-0.16124	2.889516	-4.22133	7.75E-05	0.021015	1.449278	PD
cg16328494	-0.20857	2.463536	-4.22004	7.78E-05	0.021102	1.447221	PD
cg07194694	-0.27051	3.894625	-4.21959	7.79E-05	0.021129	1.445828	PD
cg00910107	-0.57935	5.109066	-4.21925	7.80E-05	0.021147	1.443825	PD
cg11534108	-0.24103	2.516361	-4.21907	7.81E-05	0.021153	1.444419	PD
cg18036068	-0.17947	3.210686	-4.21869	7.82E-05	0.021174	1.443003	PD
cg24478926	-0.17654	6.503237	-4.21832	7.83E-05	0.021195	1.441227	PD

cg06244627	0.265938	-4.29325	4.218085	7.83E-05	0.021205	1.441219	PD
cg19610658	-0.17853	4.471131	-4.21774	7.84E-05	0.021224	1.440171	PD
cg18641876	-0.26807	0.427357	-4.21708	7.86E-05	0.021266	1.438307	PD
cg20123108	0.282893	4.034969	4.215814	7.90E-05	0.021353	1.434325	PD
cg00732602	-0.22489	2.518288	-4.21506	7.92E-05	0.021393	1.431987	PD
cg17860861	-0.23632	3.656052	-4.21501	7.92E-05	0.021393	1.426259	PD
cg15448680	0.369454	3.759923	4.214994	7.92E-05	0.021393	1.431436	PD
cg00653519	-0.1575	3.896737	-4.21475	7.93E-05	0.021404	1.431006	PD
cg06751596	0.256097	-2.83043	4.214466	7.93E-05	0.021419	1.43006	PD
cg14194576	-0.20718	2.987932	-4.21435	7.94E-05	0.02142	1.429429	PD
cg17312492	0.295148	-3.86717	4.214042	7.95E-05	0.021436	1.428819	PD
cg23787031	-0.18419	2.275019	-4.21328	7.97E-05	0.021486	1.426405	PD
cg10017978	-0.22401	2.67347	-4.21318	7.97E-05	0.021487	1.425479	PD
cg03786424	0.291339	3.606214	4.212546	7.99E-05	0.021527	1.424108	PD
cg08072145	0.194529	-0.11282	4.212382	7.99E-05	0.021527	1.422937	PD
cg20239174	-0.30158	3.893482	-4.21236	7.99E-05	0.021527	1.42305	PD
cg14109325	-0.18594	4.126052	-4.21108	8.03E-05	0.021616	1.419505	PD
cg13800833	-0.20197	3.381003	-4.21087	8.03E-05	0.021626	1.418182	PD
cg21590782	0.231656	0.515224	4.210731	8.04E-05	0.021626	1.418202	PD
cg05304616	-0.27755	3.782695	-4.21068	8.04E-05	0.021626	1.418269	PD
cg02322562	-0.12297	4.283678	-4.21037	8.05E-05	0.021642	1.417214	PD
cg22581809	-0.28869	4.230166	-4.21015	8.05E-05	0.021651	1.41656	PD
cg16292912	-0.27037	2.571984	-4.21008	8.06E-05	0.021651	1.415974	PD
cg07110142	-0.31256	1.861491	-4.20991	8.06E-05	0.021654	1.41589	PD
cg18059383	-0.15413	2.683364	-4.20985	8.06E-05	0.021654	1.415618	PD
cg09777060	0.220113	3.014749	4.209691	8.07E-05	0.021659	1.415183	PD
cg09522310	-0.22656	3.092643	-4.20948	8.07E-05	0.021666	1.414598	PD
cg06209298	-0.14197	5.263579	-4.20941	8.07E-05	0.021666	1.41433	PD
cg13558516	-0.24495	3.765581	-4.20874	8.09E-05	0.021709	1.412126	PD
cg19198054	-0.27389	2.452922	-4.20832	8.10E-05	0.021735	1.41024	PD
cg05080615	-0.20243	3.818459	-4.20807	8.11E-05	0.021746	1.410204	PD

cg01841306	0.197165	-2.63821	4.207679	8.12E-05	0.021769	1.408968	PD
cg04020319	-0.52239	4.290391	-4.20737	8.13E-05	0.021786	1.407619	PD
cg06222774	-0.2433	2.816964	-4.20699	8.14E-05	0.021798	1.406149	PD
cg22840802	0.130096	-4.82951	4.206972	8.14E-05	0.021798	1.406571	PD
cg14229934	-0.30783	4.202648	-4.20693	8.14E-05	0.021798	1.405992	PD
cg13150689	0.256811	0.5802	4.205829	8.18E-05	0.021875	1.403144	PD
cg02275878	0.366855	-5.15262	4.205366	8.19E-05	0.021903	1.401769	PD
cg22222799	-0.75214	3.167177	-4.20516	8.19E-05	0.021912	1.395955	PD
cg25343576	-0.25543	3.772234	-4.20483	8.20E-05	0.02193	1.399899	PD
cg07308232	-0.2608	4.037574	-4.20448	8.21E-05	0.02195	1.399004	PD
cg21459921	0.166291	-5.74214	4.204147	8.22E-05	0.021968	1.397781	PD
cg00308434	-0.21985	3.835451	-4.20262	8.27E-05	0.022077	1.393218	PD
cg06340704	0.261217	-2.70604	4.202396	8.27E-05	0.022088	1.39216	PD
cg13828839	0.129012	-3.71351	4.202071	8.28E-05	0.022105	1.390969	PD
cg19174044	0.312254	4.203999	4.201468	8.30E-05	0.022145	1.389522	PD
cg04794128	-0.37167	3.82106	-4.20089	8.32E-05	0.022182	1.387845	PD
cg17799868	-0.3489	3.625699	-4.20055	8.33E-05	0.022197	1.386103	PD
cg17713488	-0.50069	1.605531	-4.20051	8.33E-05	0.022197	1.386604	PD
cg06148426	-0.15912	1.13098	-4.20032	8.33E-05	0.022205	1.385634	PD
cg24152718	-0.18134	2.909205	-4.20009	8.34E-05	0.022216	1.385316	PD
cg06364873	0.294465	3.54118	4.199809	8.35E-05	0.02223	1.383103	PD
cg04820411	-0.19952	3.131872	-4.19871	8.38E-05	0.022308	1.38106	PD
cg07393854	-0.14801	3.320196	-4.19848	8.39E-05	0.022319	1.380069	PD
cg03844404	-0.29951	3.280874	-4.19741	8.42E-05	0.022395	1.376985	PD
cg11519751	-0.16906	3.086124	-4.1973	8.42E-05	0.022396	1.376663	PD
cg04605607	0.360993	-4.15786	4.197186	8.42E-05	0.022398	1.376179	PD
cg21103074	-0.25719	1.572954	-4.19663	8.44E-05	0.022434	1.373022	PD
cg20431848	-0.15915	5.796442	-4.19617	8.45E-05	0.022463	1.37295	PD
cg14815051	-0.24983	4.089513	-4.19572	8.47E-05	0.022485	1.370703	PD
cg21034324	-0.16838	1.893637	-4.19568	8.47E-05	0.022485	1.37162	PD
cg01868838	0.145659	3.531063	4.195556	8.47E-05	0.022485	1.371041	PD

cg14137935	-0.14156	4.083089	-4.19552	8.47E-05	0.022485	1.37107	PD
cg01714160	-0.17787	6.280444	-4.19469	8.50E-05	0.022543	1.368418	PD
cg17813475	-0.12252	4.281975	-4.19406	8.52E-05	0.022583	1.365923	PD
cg15103100	-0.17436	3.786604	-4.19399	8.52E-05	0.022583	1.366342	PD
cg18751588	-0.42347	-4.83208	-4.19359	8.53E-05	0.022608	1.365114	PD
cg04483596	-0.34854	3.437187	-4.19326	8.54E-05	0.022626	1.363504	PD
cg00456685	-0.86596	3.170186	-4.19289	8.55E-05	0.022649	1.351587	PD
cg07795623	-0.22267	3.254778	-4.1926	8.56E-05	0.022664	1.360818	PD
cg24986344	-0.38305	3.850411	-4.19184	8.58E-05	0.022717	1.35963	PD
cg06523784	0.687253	3.509206	4.19155	8.59E-05	0.022726	1.356973	PD
cg12982547	-0.20543	3.96977	-4.1915	8.59E-05	0.022726	1.358525	PD
cg22453634	-0.17203	3.643924	-4.19144	8.59E-05	0.022726	1.357951	PD
cg11750619	-0.31459	3.13852	-4.19049	8.62E-05	0.022795	1.355477	PD
cg13535015	0.584782	-4.8798	4.190343	8.63E-05	0.022799	1.354126	PD
cg16575075	-0.49464	3.05963	-4.18946	8.65E-05	0.022862	1.352288	PD
cg23017826	-0.23541	3.705809	-4.18934	8.66E-05	0.022864	1.351921	PD
cg23946663	-0.29712	3.690193	-4.18909	8.66E-05	0.022875	1.350811	PD
cg23931658	-0.21469	3.836838	-4.18902	8.67E-05	0.022875	1.350917	PD
cg00004700	-0.25687	2.665866	-4.18891	8.67E-05	0.022877	1.350281	PD
cg20887711	0.207025	-6.61135	4.188452	8.68E-05	0.022906	1.348917	PD
cg03865485	-0.26755	3.373124	-4.18822	8.69E-05	0.022917	1.348114	PD
cg18383734	-0.19445	2.674594	-4.18808	8.69E-05	0.022921	1.347965	PD
cg18154328	0.229326	-4.93737	4.187864	8.70E-05	0.022931	1.346738	PD
cg11427177	-0.17175	3.882456	-4.18755	8.71E-05	0.022949	1.346278	PD
cg04886391	0.14263	-5.00971	4.187264	8.72E-05	0.022964	1.34542	PD
cg23333911	-0.22317	3.494973	-4.18674	8.73E-05	0.022999	1.342922	PD
cg17714010	-0.40327	3.588192	-4.18622	8.75E-05	0.023033	1.34219	PD
cg05133814	0.183545	2.998723	4.185513	8.77E-05	0.023082	1.339425	PD
cg15508470	-0.2227	3.759405	-4.18533	8.78E-05	0.02309	1.338761	PD
cg19930569	-0.17371	3.273456	-4.18452	8.80E-05	0.023147	1.336939	PD
cg09498014	-0.14756	4.544462	-4.18362	8.83E-05	0.023209	1.333602	PD

cg06324129	-0.19598	3.588882	-4.18357	8.83E-05	0.023209	1.333162	PD
cg00196166	-0.21659	2.827932	-4.18278	8.85E-05	0.023265	1.33092	PD
cg14350662	-0.31542	3.375895	-4.18234	8.87E-05	0.023294	1.330118	PD
cg16549043	-0.32603	2.107288	-4.18148	8.89E-05	0.023355	1.326885	PD
cg20858433	-0.14866	4.027167	-4.18136	8.90E-05	0.023358	1.32713	PD
cg22995255	-0.22062	3.237933	-4.18009	8.94E-05	0.023453	1.322677	PD
cg03841081	-0.21205	3.548098	-4.17986	8.94E-05	0.023463	1.322249	PD
cg04495735	-0.28098	4.078846	-4.17979	8.95E-05	0.023463	1.322272	PD
cg26315272	-0.25261	3.705559	-4.17954	8.95E-05	0.023476	1.320646	PD
cg20986904	-0.21447	3.859107	-4.17918	8.97E-05	0.023498	1.31909	PD
cg02627405	0.153153	-0.22812	4.178997	8.97E-05	0.023506	1.31981	PD
cg06172624	-0.41439	4.013029	-4.17864	8.98E-05	0.023528	1.317999	PD
cg12940965	-0.28798	-5.39582	-4.17836	8.99E-05	0.023543	1.317818	PD
cg07706880	-0.18472	3.763332	-4.17768	9.01E-05	0.023592	1.315709	PD
cg22040867	-0.24913	3.291578	-4.17721	9.03E-05	0.023622	1.313379	PD
cg14522718	-0.47838	4.277723	-4.17712	9.03E-05	0.023622	1.313666	PD
cg04854162	0.241247	3.890069	4.176834	9.04E-05	0.023637	1.312818	PD
cg14894961	-0.11217	-0.34099	-4.17676	9.04E-05	0.023637	1.312682	PD
cg01037262	-0.25015	3.714999	-4.17646	9.05E-05	0.023655	1.311775	PD
cg16217885	-0.31386	3.506848	-4.17567	9.07E-05	0.023711	1.308707	PD
cg24717401	0.332265	-6.88328	4.174763	9.10E-05	0.023779	1.306515	PD
cg01535003	0.131646	4.063984	4.174511	9.11E-05	0.023792	1.3059	PD
cg23944405	-0.51024	3.129309	-4.17398	9.13E-05	0.023828	1.303385	PD
cg25712005	-0.30878	2.065431	-4.17322	9.15E-05	0.023884	1.3019	PD
cg24398822	0.168463	-5.27302	4.172907	9.16E-05	0.023902	1.299729	PD
cg00826304	-0.22341	3.145855	-4.17265	9.17E-05	0.023912	1.29978	PD
cg26548410	-0.36162	4.238167	-4.17261	9.17E-05	0.023912	1.300015	PD
cg00462170	-0.14914	3.305498	-4.17213	9.19E-05	0.023945	1.298499	PD
cg11306587	0.114038	-3.64533	4.171743	9.20E-05	0.023965	1.297126	PD
cg00986133	-0.18108	3.47387	-4.17168	9.20E-05	0.023965	1.297014	PD
cg14677909	-0.36628	3.549341	-4.17159	9.20E-05	0.023965	1.296284	PD

cg13480447	-0.17541	3.455248	-4.17153	9.21E-05	0.023965	1.295952	PD
cg13320898	-0.24534	5.456685	-4.1709	9.23E-05	0.024009	1.294704	PD
cg13294602	-0.14169	4.898846	-4.17057	9.24E-05	0.024029	1.292643	PD
cg05533242	-0.18212	3.797159	-4.17018	9.25E-05	0.024054	1.292438	PD
cg10976213	-0.1568	3.428036	-4.16991	9.26E-05	0.024064	1.291037	PD
cg22352371	-0.19391	2.271299	-4.16986	9.26E-05	0.024064	1.29099	PD
cg10183345	0.215509	4.490011	4.169795	9.26E-05	0.024064	1.290541	PD
cg11528086	0.625792	2.95315	4.169125	9.28E-05	0.024112	1.284118	PD
cg22355889	-0.61288	-3.73356	-4.16892	9.29E-05	0.02412	1.288589	PD
cg12028751	-0.29263	3.083817	-4.16885	9.29E-05	0.02412	1.288086	PD
cg26119382	-0.22407	2.466956	-4.16873	9.30E-05	0.024123	1.287784	PD
cg02359773	-0.30509	6.133655	-4.16805	9.32E-05	0.024172	1.284975	PD
cg04249612	-0.21756	3.563187	-4.166	9.38E-05	0.024337	1.279494	PD
cg01662591	-0.35159	3.467356	-4.16571	9.39E-05	0.024353	1.27825	PD
cg14172981	0.78138	3.792559	4.16451	9.43E-05	0.024447	1.274334	PD
cg27419119	-0.20315	6.64848	-4.16413	9.44E-05	0.024471	1.273309	PD
cg17362351	-0.21399	2.51091	-4.16387	9.45E-05	0.024486	1.272744	PD
cg23553085	0.206621	4.404153	4.163063	9.48E-05	0.024547	1.270162	PD
cg14887482	-0.20467	3.739692	-4.16291	9.48E-05	0.024552	1.269652	PD
cg05601623	2.369786	-3.94805	4.162282	9.50E-05	0.024598	1.266303	PD
cg20950152	0.287494	3.109621	4.161854	9.52E-05	0.024627	1.265907	PD
cg14132364	-0.17993	4.127594	-4.16115	9.54E-05	0.024679	1.262987	PD
cg17258727	-0.2217	3.519409	-4.16087	9.55E-05	0.024695	1.262659	PD
cg08129093	-0.16738	1.583492	-4.16026	9.57E-05	0.024739	1.261467	PD
cg13295812	-0.23224	1.676587	-4.16001	9.58E-05	0.024747	1.260079	PD
cg10558206	-0.34589	3.093986	-4.16	9.58E-05	0.024747	1.259989	PD
cg22898362	-0.31679	3.033689	-4.15969	9.59E-05	0.024763	1.258876	PD
cg01916477	-0.21473	3.802637	-4.15963	9.59E-05	0.024763	1.258844	PD
cg20064577	-0.1804	3.644712	-4.15862	9.63E-05	0.024842	1.256461	PD
cg12762715	0.142278	-4.28701	4.158409	9.63E-05	0.024853	1.255064	PD
cg23714722	0.239964	2.706022	4.158009	9.65E-05	0.024879	1.254795	PD

cg23721529	-0.59283	-6.68612	-4.15758	9.66E-05	0.024909	1.252062	PD
cg09776041	0.356942	2.631112	4.157386	9.67E-05	0.024914	1.252714	PD
cg10277378	-0.24647	3.000625	-4.15728	9.67E-05	0.024914	1.25143	PD
cg05932172	-0.20496	2.684813	-4.15725	9.67E-05	0.024914	1.251244	PD
cg12648931	-1.40644	-4.58427	-4.15706	9.68E-05	0.024923	1.25156	PD
cg14009547	-0.17082	4.478923	-4.15677	9.69E-05	0.024932	1.249757	PD
cg13521254	0.59131	-4.98897	4.156699	9.69E-05	0.024932	1.250757	PD
cg02429595	-0.17078	3.636212	-4.15669	9.69E-05	0.024932	1.250025	PD
cg21715802	-0.20995	3.25047	-4.15641	9.70E-05	0.024948	1.249824	PD
cg19037467	-0.24458	2.686673	-4.15624	9.70E-05	0.024956	1.249217	PD
cg06205922	-0.25587	3.818786	-4.15593	9.71E-05	0.024975	1.24832	PD
cg21521866	0.248422	3.796665	4.1557	9.72E-05	0.024987	1.246875	PD
cg12436713	0.10791	-4.158	4.154706	9.76E-05	0.025065	1.243432	PD
cg14196840	0.281992	-5.11927	4.154583	9.76E-05	0.025068	1.243405	PD
cg08258650	0.330878	-4.74371	4.154197	9.77E-05	0.025094	1.242588	PD
cg17810431	0.180651	4.085234	4.15398	9.78E-05	0.025105	1.242315	PD
cg15726857	0.465368	2.816792	4.153575	9.79E-05	0.025123	1.240817	PD
cg16996965	-0.23464	2.859578	-4.15353	9.80E-05	0.025123	1.239488	PD
cg16561957	-0.30915	5.102634	-4.15351	9.80E-05	0.025123	1.240919	PD
cg23048001	-0.19081	3.965409	-4.15185	9.85E-05	0.02526	1.235114	PD
cg15391105	0.172793	-5.41338	4.150792	9.89E-05	0.025344	1.232534	PD
cg01919011	-0.19978	2.515487	-4.15016	9.91E-05	0.025389	1.230491	PD
cg09576866	-1.00375	3.041576	-4.15009	9.91E-05	0.025389	1.219805	PD
cg12762733	-0.27458	-3.08032	-4.14998	9.92E-05	0.025392	1.22997	PD
cg16935517	0.405783	3.826615	4.149501	9.93E-05	0.025426	1.219279	PD
cg04670673	-0.23481	3.230921	-4.14925	9.94E-05	0.025441	1.226457	PD
cg15933028	0.134551	-3.63095	4.149068	9.95E-05	0.025449	1.22128	PD
cg07064689	-0.21109	2.785813	-4.14865	9.96E-05	0.025478	1.22562	PD
cg13246505	-0.15996	4.362764	-4.14853	9.97E-05	0.025481	1.223124	PD
cg05193110	-0.23809	2.994205	-4.14833	9.97E-05	0.025485	1.224315	PD
cg11973903	-0.30484	3.873786	-4.1483	9.97E-05	0.025485	1.224855	PD

cg06887137	-0.23887	0.91279	-4.14744	0.0001	0.025553	1.220762	PD
cg19717768	-0.13762	4.179455	-4.14692	0.0001	0.025591	1.22042	PD
cg00630399	-0.27456	4.449081	-4.14632	0.0001	0.025636	1.217723	PD
cg15697201	0.953214	3.082017	4.145571	0.000101	0.025695	1.216071	PD
cg04340430	-0.15113	4.270954	-4.14514	0.000101	0.025725	1.214962	PD
cg20722353	-0.31411	3.168884	-4.14452	0.000101	0.025772	1.21187	PD
cg07130601	-0.17076	3.850803	-4.14426	0.000101	0.025787	1.211411	PD
cg00416934	0.155475	-2.47715	4.143717	0.000101	0.025828	1.210484	PD
cg06457099	-0.16884	5.075294	-4.14341	0.000101	0.025848	1.209751	PD
cg18349159	0.227684	2.141145	4.142213	0.000102	0.025947	1.205673	PD
cg19589283	-0.16789	3.382808	-4.14199	0.000102	0.025954	1.205094	PD
cg24433586	-0.24449	3.529492	-4.14193	0.000102	0.025954	1.205008	PD
cg04807512	-0.22083	3.63685	-4.14186	0.000102	0.025954	1.204881	PD
cg12963319	0.388147	1.417552	4.141396	0.000102	0.025988	1.203252	PD
cg11865578	-0.14514	5.548752	-4.14094	0.000102	0.026021	1.202013	PD
cg26356249	-0.26088	2.356851	-4.14064	0.000102	0.02604	1.200386	PD
cg04813403	-0.20044	2.202412	-4.14008	0.000103	0.026082	1.19933	PD
cg22948672	0.13574	-0.50564	4.139931	0.000103	0.026088	1.198525	PD
cg20818222	-0.20812	2.133352	-4.13972	0.000103	0.0261	1.198375	PD
cg16832975	-0.17029	4.053856	-4.13941	0.000103	0.026119	1.194686	PD
cg16967461	-0.19597	3.869853	-4.13893	0.000103	0.026155	1.195254	PD
cg13150279	-0.28593	3.166223	-4.1382	0.000103	0.026212	1.192846	PD
cg08405087	-0.2693	3.47925	-4.13784	0.000103	0.026237	1.191844	PD
cg19008100	-0.4333	3.057773	-4.13747	0.000104	0.026263	1.191337	PD
cg19622415	-0.1438	4.959143	-4.13676	0.000104	0.026319	1.188039	PD
cg25365518	-0.17942	0.352138	-4.13641	0.000104	0.026343	1.187856	PD
cg12134559	0.16818	0.109605	4.136168	0.000104	0.026357	1.186576	PD
cg08355109	-0.17996	4.996152	-4.1358	0.000104	0.026382	1.185199	PD
cg10575758	-0.1876	2.203284	-4.13463	0.000105	0.026481	1.182663	PD
cg01596995	-0.22941	2.318005	-4.13451	0.000105	0.026484	1.182216	PD
cg23324580	-0.20827	3.252673	-4.13422	0.000105	0.026502	1.179812	PD

cg21473728	-0.23714	3.768734	-4.1341	0.000105	0.026503	1.18045	PD
cg00301576	-0.24733	2.460573	-4.13398	0.000105	0.026503	1.17995	PD
cg18522578	-0.28743	3.498583	-4.13395	0.000105	0.026503	1.179079	PD
cg26052200	-0.18422	3.273297	-4.13314	0.000105	0.026569	1.176902	PD
cg18836583	-0.24543	1.724838	-4.13239	0.000105	0.026629	1.175403	PD
cg19451915	-0.13709	3.295763	-4.13231	0.000105	0.026629	1.175347	PD
cg09683682	-0.26647	3.511098	-4.13221	0.000105	0.026631	1.174677	PD
cg00009085	0.158179	-0.62401	4.131664	0.000106	0.026669	1.173443	PD
cg13886354	0.313912	-5.65894	4.131618	0.000106	0.026669	1.172541	PD
cg22039839	-0.15354	4.479665	-4.13135	0.000106	0.026683	1.170909	PD
cg23321477	-0.35162	3.346736	-4.13121	0.000106	0.026683	1.172053	PD
cg07358846	0.258378	3.286295	4.131199	0.000106	0.026683	1.172033	PD
cg03398844	-0.32875	2.85488	-4.13102	0.000106	0.026683	1.170283	PD
cg23452174	-0.28269	3.042088	-4.13089	0.000106	0.026683	1.170928	PD
cg14043652	-0.19497	3.739227	-4.13087	0.000106	0.026683	1.17007	PD
cg24577116	-0.19597	3.355217	-4.13085	0.000106	0.026683	1.170709	PD
cg10739948	0.616033	2.478027	4.13011	0.000106	0.026739	1.168754	PD
cg04615725	-0.32637	2.850562	-4.13007	0.000106	0.026739	1.168398	PD
cg20564330	-0.22046	3.192792	-4.12961	0.000106	0.026768	1.164433	PD
cg25735490	-0.11057	5.920132	-4.12958	0.000106	0.026768	1.165719	PD
cg25577101	-0.19705	3.56615	-4.12895	0.000107	0.026811	1.164627	PD
cg11596893	0.23161	3.96374	4.128938	0.000107	0.026811	1.162435	PD
cg26508465	-0.14195	3.991722	-4.12886	0.000107	0.026811	1.163563	PD
cg08676614	-0.22854	3.023735	-4.12848	0.000107	0.026838	1.161658	PD
cg26083859	-0.2443	4.166109	-4.12777	0.000107	0.026895	1.160938	PD
cg03536879	-0.42674	2.216203	-4.12737	0.000107	0.026919	1.159739	PD
cg03340228	-0.21554	1.502623	-4.12734	0.000107	0.026919	1.159948	PD
cg18077587	-0.26403	2.724373	-4.12692	0.000107	0.02695	1.158047	PD
cg10986104	-0.20606	4.142145	-4.12639	0.000108	0.026991	1.155051	PD
cg03351431	0.194774	3.84318	4.126272	0.000108	0.026994	1.155096	PD
cg01820376	-0.15804	3.033783	-4.12602	0.000108	0.027009	1.156246	PD

cg18174569	-0.14816	3.683676	-4.12585	0.000108	0.027017	1.154495	PD
cg25628542	0.385865	3.33813	4.12541	0.000108	0.02705	1.152688	PD
cg08206848	-0.14495	3.666908	-4.12464	0.000108	0.027113	1.151991	PD
cg06026592	-0.21127	2.384522	-4.12416	0.000108	0.02715	1.147779	PD
cg13363072	0.279541	-3.07633	4.123827	0.000108	0.027173	1.148135	PD
cg27410828	-0.48234	-4.46468	-4.12369	0.000109	0.027178	1.147441	PD
cg18790187	-0.17327	3.94375	-4.12338	0.000109	0.027199	1.147139	PD
cg26882909	-0.20564	3.23682	-4.12238	0.000109	0.027284	1.144877	PD
cg16577546	-0.71186	3.22544	-4.12202	0.000109	0.02731	1.143951	PD
cg16678169	0.425187	-4.97324	4.121753	0.000109	0.027327	1.143111	PD
cg04246437	-0.27863	2.904774	-4.1215	0.000109	0.027343	1.141103	PD
cg11529236	-0.19286	3.560583	-4.12107	0.00011	0.027369	1.140355	PD
cg27024704	-0.14509	3.212262	-4.12101	0.00011	0.027369	1.140717	PD
cg04901499	-0.19517	3.881171	-4.12097	0.00011	0.027369	1.139398	PD
cg14735227	0.104407	-5.26672	4.120589	0.00011	0.027396	1.139428	PD
cg15699386	-0.15888	5.616727	-4.12035	0.00011	0.027405	1.13839	PD
cg11386709	0.174319	4.361585	4.120317	0.00011	0.027405	1.138429	PD
cg24511167	-0.34814	2.38037	-4.11996	0.00011	0.027431	1.136108	PD
cg13858223	-0.34075	3.967617	-4.11984	0.00011	0.027434	1.134263	PD
cg21229317	-0.22573	4.188235	-4.11961	0.00011	0.027448	1.13606	PD
cg17039224	-0.21648	3.316082	-4.11924	0.00011	0.027474	1.134177	PD
cg26462931	0.246299	-6.3149	4.119048	0.00011	0.027484	1.133974	PD
cg00355784	-0.37041	3.882844	-4.11887	0.00011	0.027493	1.134168	PD
cg05955275	-0.14264	3.27868	-4.11871	0.00011	0.0275	1.133719	PD
cg10246450	-0.17332	3.019113	-4.11862	0.00011	0.027501	1.130891	PD
cg26211800	-0.16956	3.618345	-4.11806	0.000111	0.027533	1.130809	PD
cg01806702	-0.13598	4.21065	-4.11804	0.000111	0.027533	1.131393	PD
cg26632446	-0.21418	3.131676	-4.11802	0.000111	0.027533	1.131421	PD
cg14887116	-0.20808	3.594279	-4.11779	0.000111	0.027546	1.128138	PD
cg24723129	0.160596	0.317343	4.117223	0.000111	0.027588	1.128615	PD
cg25034968	-0.23646	3.548943	-4.11715	0.000111	0.027588	1.128522	PD

cg17174493	0.471116	-5.34403	4.117018	0.000111	0.027588	1.126174	PD
cg21239901	0.122654	-4.41381	4.11701	0.000111	0.027588	1.128311	PD
cg18856501	-0.13872	3.64342	-4.11674	0.000111	0.027606	1.127705	PD
cg09473676	-0.14819	3.412785	-4.11664	0.000111	0.027607	1.124789	PD
cg24858658	0.235514	-4.296	4.116359	0.000111	0.027618	1.125822	PD
cg02595823	0.289839	-2.75533	4.116346	0.000111	0.027618	1.125954	PD
cg21296578	-0.18579	3.17026	-4.11602	0.000111	0.02762	1.124064	PD
cg01275669	-0.78477	-4.99817	-4.11591	0.000111	0.02762	1.123911	PD
cg14148441	0.272589	4.221947	4.115728	0.000112	0.02762	1.122093	PD
cg07906050	-0.19107	3.146059	-4.11572	0.000112	0.02762	1.122522	PD
cg11880010	-0.23109	3.053599	-4.11571	0.000112	0.02762	1.122109	PD
cg00102685	-0.32035	3.795732	-4.1157	0.000112	0.02762	1.122967	PD
cg06421658	-0.19586	2.886285	-4.1157	0.000112	0.02762	1.124518	PD
cg06324554	-0.34439	2.369105	-4.11564	0.000112	0.02762	1.124357	PD
cg23501962	0.171716	-3.13957	4.115479	0.000112	0.027628	1.121293	PD
cg07018106	-0.12012	4.828437	-4.1138	0.000112	0.027779	1.118007	PD
cg23989409	-0.22141	3.97846	-4.11353	0.000112	0.027797	1.117588	PD
cg11089813	0.232387	4.120106	4.113425	0.000112	0.027799	1.116538	PD
cg06644655	-0.23522	3.468137	-4.11292	0.000113	0.027833	1.114097	PD
cg03916908	0.193462	0.437188	4.112892	0.000113	0.027833	1.114648	PD
cg13882393	-0.35511	3.538464	-4.11257	0.000113	0.027856	1.114688	PD
cg19800640	0.288567	3.155915	4.112235	0.000113	0.02788	1.111919	PD
cg19137662	0.396065	-5.92905	4.111391	0.000113	0.027934	1.111319	PD
cg11736524	-0.17643	3.500332	-4.11137	0.000113	0.027934	1.109418	PD
cg26886334	-0.16583	3.671258	-4.11131	0.000113	0.027934	1.109911	PD
cg13488570	0.233018	-5.66786	4.111248	0.000113	0.027934	1.110449	PD
cg19556217	-0.31434	3.603315	-4.11125	0.000113	0.027934	1.110418	PD
cg22633988	0.141679	-4.34229	4.109043	0.000114	0.028137	1.104115	PD
cg24562927	-0.13309	4.317666	-4.10884	0.000114	0.028149	1.101157	PD
cg01590359	-0.1428	3.831266	-4.10849	0.000114	0.028175	1.101428	PD
cg26935330	0.378991	3.622712	4.108146	0.000114	0.028199	1.101071	PD

cg20058667	0.13674	-4.26221	4.107914	0.000115	0.028214	1.099645	PD
cg17699910	-0.17099	3.507727	-4.10753	0.000115	0.028243	1.098016	PD
cg13057898	-0.19615	3.703383	-4.1073	0.000115	0.028257	1.096393	PD
cg16737267	-0.11466	4.252643	-4.10689	0.000115	0.028288	1.096	PD
cg12699836	-0.16546	3.759743	-4.1066	0.000115	0.028308	1.095262	PD
cg21876181	-0.14498	3.908682	-4.10632	0.000115	0.028327	1.093413	PD
cg16592781	0.456939	3.167895	4.105271	0.000116	0.028421	1.091391	PD
cg03490776	-0.21258	2.940814	-4.10509	0.000116	0.028431	1.090319	PD
cg18630142	-0.18485	2.496115	-4.10407	0.000116	0.028521	1.087879	PD
cg21855135	-0.24721	-0.9486	-4.10384	0.000116	0.028536	1.086911	PD
cg13561817	0.28341	4.161529	4.102999	0.000117	0.02861	1.084066	PD
cg07460883	0.162155	-1.10143	4.102182	0.000117	0.028682	1.081235	PD
cg17454263	-0.49146	-2.98795	-4.10199	0.000117	0.028684	1.081425	PD
cg08678925	-0.25961	3.265367	-4.10199	0.000117	0.028684	1.080438	PD
cg26536285	-0.34114	3.710541	-4.1018	0.000117	0.028694	1.081408	PD
cg24846332	-0.19216	3.651181	-4.10163	0.000117	0.028702	1.078684	PD
cg00465198	-0.12695	3.769714	-4.10016	0.000118	0.028839	1.07637	PD
cg13689213	-0.22142	3.22851	-4.09952	0.000118	0.028894	1.072591	PD
cg20609368	0.203009	4.223625	4.099283	0.000118	0.028904	1.073605	PD
cg21636610	-0.16008	3.239336	-4.09917	0.000118	0.028904	1.072984	PD
cg14215363	0.199778	3.469159	4.09916	0.000118	0.028904	1.073607	PD
cg13728797	-0.24127	3.93159	-4.09861	0.000118	0.028951	1.070548	PD
cg18755082	-0.24062	3.25248	-4.09797	0.000119	0.029001	1.068704	PD
cg08123100	-0.20532	3.385643	-4.09793	0.000119	0.029001	1.069797	PD
cg23579436	-0.12721	3.248548	-4.09739	0.000119	0.029046	1.066796	PD
cg27087192	-0.23071	3.779913	-4.09723	0.000119	0.029054	1.065329	PD
cg07122365	-0.24935	3.490813	-4.09706	0.000119	0.029063	1.066449	PD
cg24827600	0.465126	3.575866	4.096966	0.000119	0.029063	1.064424	PD
cg19604896	-0.17559	2.564783	-4.09662	0.000119	0.029086	1.063894	PD
cg00074313	0.215893	2.868792	4.096576	0.000119	0.029086	1.06535	PD
cg18348059	0.387561	4.067241	4.096155	0.000119	0.029105	1.063678	PD

cg25612297	-0.12724	3.746117	-4.09615	0.000119	0.029105	1.064533	PD
cg23235069	-0.15342	3.70785	-4.09613	0.000119	0.029105	1.061886	PD
cg21242061	-0.16907	2.922883	-4.09604	0.000119	0.029106	1.064047	PD
cg04994795	-0.32301	2.846496	-4.09589	0.000119	0.029112	1.061424	PD
cg19700150	0.287302	4.369065	4.09576	0.000119	0.029116	1.062818	PD
cg25450196	-0.19259	4.11346	-4.09531	0.00012	0.029153	1.060332	PD
cg18873878	-0.17438	1.442945	-4.09517	0.00012	0.029159	1.060794	PD
cg09606766	0.13756	-3.80166	4.094245	0.00012	0.029235	1.057761	PD
cg11238064	-0.19199	3.366195	-4.09423	0.00012	0.029235	1.057188	PD
cg15099347	0.283798	3.551223	4.093988	0.00012	0.029251	1.053469	PD
cg13738384	0.235151	1.760875	4.093773	0.00012	0.029265	1.056813	PD
cg00082729	0.311369	2.106632	4.093402	0.00012	0.029293	1.055676	PD
cg00507259	0.13119	-3.97148	4.092919	0.000121	0.029332	1.05285	PD
cg25425801	0.308687	-3.84788	4.092849	0.000121	0.029332	1.052789	PD
cg02133140	-0.22954	4.339984	-4.0924	0.000121	0.029369	1.052882	PD
cg16943151	-0.24508	2.788751	-4.09162	0.000121	0.029438	1.050087	PD
cg24456002	0.191729	-2.00025	4.090957	0.000121	0.029497	1.047248	PD
cg27620176	-0.12991	6.170145	-4.09055	0.000122	0.02953	1.045133	PD
cg16439198	0.229927	-4.58481	4.089558	0.000122	0.029621	1.043116	PD
cg21035907	0.388478	3.293957	4.089313	0.000122	0.02963	1.041409	PD
cg14067716	-0.25748	3.880952	-4.0893	0.000122	0.02963	1.041783	PD
cg09990232	-0.15398	2.894812	-4.08892	0.000122	0.029661	1.042181	PD
cg12875901	-0.15099	2.69091	-4.08883	0.000122	0.029661	1.041636	PD
cg06197356	0.237619	3.628269	4.08832	0.000123	0.029704	1.040619	PD
cg23676042	-0.15711	2.360313	-4.08799	0.000123	0.02973	1.039621	PD
cg08396995	-0.20472	2.409103	-4.08738	0.000123	0.029778	1.036758	PD
cg14231959	-0.21789	2.525706	-4.08734	0.000123	0.029778	1.035288	PD
cg11755803	0.254391	3.692958	4.08649	0.000123	0.029852	1.031508	PD
cg20821566	0.551533	4.512067	4.086448	0.000123	0.029852	1.034478	PD
cg00905249	-0.1679	3.303045	-4.08597	0.000124	0.029892	1.03355	PD
cg23003926	-0.14043	2.974214	-4.08589	0.000124	0.029892	1.032845	PD

cg25999148	0.325721	3.646849	4.085547	0.000124	0.029919	1.030833	PD
cg02737782	0.268303	-5.83614	4.084423	0.000124	0.030023	1.027036	PD
cg07901206	-0.17801	3.561152	-4.08436	0.000124	0.030023	1.027938	PD
cg16006184	0.264547	2.918957	4.084106	0.000124	0.030037	1.027161	PD
cg11841722	0.199584	-5.2275	4.084027	0.000124	0.030037	1.027533	PD
cg12087941	-0.13845	5.077455	-4.08395	0.000124	0.030037	1.026439	PD
cg27069372	0.142605	-3.74393	4.083895	0.000124	0.030037	1.026472	PD
cg02193187	-0.20607	1.219474	-4.08379	0.000124	0.030039	1.0244	PD
cg03956737	-0.14504	3.111223	-4.08344	0.000125	0.030065	1.024507	PD
cg21502751	-0.23717	3.129841	-4.08337	0.000125	0.030065	1.024328	PD
cg24192058	0.195661	-0.16968	4.083149	0.000125	0.030079	1.023416	PD
cg16904092	0.25952	1.083957	4.082743	0.000125	0.030112	1.021251	PD
cg16027677	-0.25256	2.569956	-4.08206	0.000125	0.030174	1.021497	PD
cg20490088	0.170635	-1.03816	4.081609	0.000125	0.030212	1.018044	PD
cg13393580	-0.21301	2.325065	-4.08132	0.000126	0.030233	1.018823	PD
cg23537353	-0.27677	3.988171	-4.08121	0.000126	0.030233	1.017501	PD
cg21792018	-0.21855	3.579659	-4.08116	0.000126	0.030233	1.01633	PD
cg24947637	1.150554	4.007545	4.08091	0.000126	0.03025	1.017617	PD
cg15413909	-0.21514	3.656568	-4.08042	0.000126	0.030292	1.015817	PD
cg03365111	-0.26342	3.860997	-4.07837	0.000127	0.030496	1.009304	PD
cg08340572	0.382386	-3.31582	4.078141	0.000127	0.030511	1.008804	PD
cg03172657	-0.2198	3.480595	-4.07798	0.000127	0.030519	1.006237	PD
cg15903974	-0.18557	2.403542	-4.07713	0.000127	0.030599	1.004942	PD
cg21300908	-0.19251	2.340745	-4.07631	0.000128	0.030676	1.002445	PD
cg17699276	3.760594	-4.18309	4.076023	0.000128	0.030693	0.999196	PD
cg00602371	-0.13244	4.759775	-4.07598	0.000128	0.030693	1.002369	PD
cg26994484	0.133323	-2.76938	4.07581	0.000128	0.030703	0.998679	PD
cg15247500	-0.16808	3.656166	-4.07552	0.000128	0.030725	0.999863	PD
cg00883671	-0.26281	2.886363	-4.07526	0.000128	0.030736	1.000808	PD
cg02560739	-0.3201	3.412374	-4.07513	0.000128	0.030736	0.99957	PD
cg02634641	-0.16188	5.596432	-4.0749	0.000128	0.030736	0.998628	PD

cg24452588	-0.35457	2.826785	-4.07489	0.000128	0.030736	0.997234	PD
cg02729641	-0.45107	3.18099	-4.07488	0.000128	0.030736	0.999317	PD
cg05086993	0.190313	-4.87954	4.074872	0.000128	0.030736	0.998076	PD
cg12371336	-0.21936	2.548511	-4.07474	0.000128	0.030736	0.996365	PD
cg21544075	-0.29077	2.459848	-4.07469	0.000128	0.030736	0.997572	PD
cg14696444	-0.18861	1.988407	-4.07467	0.000128	0.030736	0.995299	PD
cg11756300	-0.21398	2.869839	-4.07457	0.000128	0.030736	0.996387	PD
cg10139365	-0.26925	3.960842	-4.07442	0.000128	0.030744	0.9969	PD
cg17132517	-0.20652	3.456799	-4.07364	0.000129	0.030799	0.994403	PD
cg17436460	0.146991	-0.84211	4.073611	0.000129	0.030799	0.994816	PD
cg14590027	0.26001	4.115668	4.0736	0.000129	0.030799	0.994134	PD
cg17381040	0.281984	3.675088	4.073568	0.000129	0.030799	0.994602	PD
cg07270470	0.264478	3.145792	4.073115	0.000129	0.030835	0.992537	PD
cg09442489	-0.19861	3.439549	-4.07305	0.000129	0.030835	0.993109	PD
cg02467435	0.145381	-4.23352	4.072754	0.000129	0.030858	0.993196	PD
cg15172601	0.135214	-4.26578	4.071773	0.00013	0.030953	0.988986	PD
cg13324220	0.776833	3.559959	4.071671	0.00013	0.030955	0.989257	PD
cg03780545	0.117325	-4.56398	4.071449	0.00013	0.03097	0.988407	PD
cg14665227	0.267322	4.042835	4.070609	0.00013	0.031042	0.9845	PD
cg23691040	-0.22554	2.564201	-4.0706	0.00013	0.031042	0.98379	PD
cg02440512	0.225956	3.549934	4.070349	0.00013	0.031059	0.98564	PD
cg09708901	-0.22681	1.900541	-4.07027	0.00013	0.031059	0.985295	PD
cg20909615	-0.25319	2.5877	-4.0698	0.000131	0.031101	0.981154	PD
cg14383264	-0.25478	4.255787	-4.06943	0.000131	0.031129	0.9813	PD
cg21245975	-0.36724	-3.81313	-4.06937	0.000131	0.031129	0.979178	PD
cg01550161	-0.25471	3.92111	-4.0692	0.000131	0.031138	0.981513	PD
cg27641266	0.240134	4.81349	4.068562	0.000131	0.031197	0.976919	PD
cg22017581	0.135586	-4.49073	4.06755	0.000132	0.031296	0.976001	PD
cg23390411	-0.29129	3.441594	-4.06718	0.000132	0.031327	0.97293	PD
cg22402121	1.85503	5.009266	4.066465	0.000132	0.031395	0.971615	PD
cg22702056	-0.12404	4.989666	-4.06633	0.000132	0.0314	0.971298	PD

cg09753772	-0.19494	1.198349	-4.06591	0.000132	0.031436	0.972215	PD
cg13455069	-0.24108	3.566816	-4.06541	0.000133	0.031481	0.966704	PD
cg25336926	-2.22726	-4.88988	-4.06521	0.000133	0.031492	0.966091	PD
cg06530217	-0.2589	3.146299	-4.06514	0.000133	0.031492	0.968525	PD
cg00588689	-0.15241	4.372412	-4.06496	0.000133	0.031503	0.967299	PD
cg11732753	1.153733	3.763948	4.064879	0.000133	0.031503	0.967639	PD
cg07259162	-0.1931	5.272389	-4.06427	0.000133	0.031559	0.966428	PD
cg04241123	0.294056	3.698044	4.063684	0.000133	0.031614	0.962576	PD
cg16505623	0.217985	2.75854	4.063196	0.000134	0.031658	0.96301	PD
cg19746753	-0.20193	3.003005	-4.06272	0.000134	0.0317	0.962127	PD
cg21127601	0.360982	4.243652	4.062599	0.000134	0.031704	0.960439	PD
cg24118713	0.30969	4.238674	4.062311	0.000134	0.031727	0.96135	PD
cg03312426	0.179747	-4.6424	4.061821	0.000134	0.031771	0.959016	PD
cg13084560	-0.17462	4.030743	-4.06148	0.000134	0.031799	0.955349	PD
cg23126094	-0.23983	3.590056	-4.06132	0.000134	0.031807	0.955141	PD
cg20503294	-0.22895	4.05431	-4.06118	0.000134	0.031814	0.956307	PD
cg19011911	0.235947	3.537903	4.06096	0.000135	0.031828	0.956134	PD
cg20072437	-0.31899	3.629195	-4.05984	0.000135	0.031941	0.953557	PD
cg15131854	-0.16212	3.846559	-4.05973	0.000135	0.031944	0.953317	PD
cg00926942	-0.17987	3.756709	-4.05916	0.000135	0.031998	0.949241	PD
cg10371998	-0.21058	2.64259	-4.05906	0.000135	0.032	0.948682	PD
cg14254422	-0.13419	3.956215	-4.05862	0.000136	0.032033	0.947191	PD
cg14999031	0.234171	3.712013	4.058584	0.000136	0.032033	0.94845	PD
cg16469117	-0.32007	2.981108	-4.05835	0.000136	0.032037	0.947916	PD
cg03673016	-0.27591	5.045943	-4.05831	0.000136	0.032037	0.949054	PD
cg24062598	-0.25726	4.049668	-4.05831	0.000136	0.032037	0.946002	PD
cg24203562	-0.30056	1.966224	-4.058	0.000136	0.032061	0.943036	PD
cg04559159	-0.27042	3.802804	-4.05789	0.000136	0.032065	0.94509	PD
cg16650201	-0.29739	3.189803	-4.05776	0.000136	0.03207	0.945405	PD
cg24835883	-0.25626	3.704507	-4.05721	0.000136	0.032121	0.945952	PD
cg23618477	-0.33221	1.754456	-4.05701	0.000136	0.032133	0.940581	PD

cg24083627	-0.16376	3.216255	-4.05667	0.000137	0.032153	0.942781	PD
cg14032070	-0.21086	2.407858	-4.05667	0.000137	0.032153	0.943939	PD
cg10413136	-0.15754	4.12469	-4.05643	0.000137	0.032157	0.940745	PD
cg07809301	-0.27075	3.376725	-4.0564	0.000137	0.032157	0.941664	PD
cg20455874	-0.19495	3.443411	-4.05639	0.000137	0.032157	0.94179	PD
cg24443494	-0.30147	2.472399	-4.05581	0.000137	0.032212	0.9416	PD
cg08225020	-0.09944	5.843645	-4.05527	0.000137	0.032262	0.937582	PD
cg10895682	0.246098	3.367047	4.054986	0.000137	0.032284	0.936043	PD
cg22255808	0.190595	4.016058	4.054804	0.000137	0.032295	0.938157	PD
cg09206169	-0.3235	4.028585	-4.05378	0.000138	0.032399	0.935381	PD
cg10272369	-0.22014	3.106629	-4.05345	0.000138	0.032427	0.932935	PD
cg27047044	-0.17076	2.818277	-4.05336	0.000138	0.032428	0.930931	PD
cg14391923	-0.21316	2.66564	-4.05316	0.000138	0.032441	0.932333	PD
cg20343161	-0.20506	3.640218	-4.053	0.000138	0.032446	0.930001	PD
cg07433706	-0.69316	2.851037	-4.05295	0.000138	0.032446	0.932724	PD
cg01060282	0.253917	3.657204	4.052555	0.000138	0.03248	0.929876	PD
cg21277192	-0.14053	4.186102	-4.05144	0.000139	0.032595	0.924669	PD
cg07389305	-0.18539	3.285417	-4.05129	0.000139	0.032603	0.924937	PD
cg05151461	-0.14107	4.78756	-4.05097	0.000139	0.032629	0.923293	PD
cg07383370	-0.28734	2.415678	-4.05069	0.000139	0.032651	0.924337	PD
cg02612276	-0.13752	3.164428	-4.05036	0.000139	0.032671	0.924934	PD
cg22290206	-0.19348	3.112752	-4.05035	0.000139	0.032671	0.923096	PD
cg19236980	-0.24113	3.705329	-4.0501	0.00014	0.032689	0.923836	PD
cg15941886	0.14038	-1.01143	4.04994	0.00014	0.032698	0.923034	PD
cg06299791	-0.20317	3.98644	-4.04932	0.00014	0.032759	0.920037	PD
cg24356530	-0.23823	3.467799	-4.04913	0.00014	0.032764	0.921029	PD
cg03031009	-0.20924	1.822851	-4.04911	0.00014	0.032764	0.920137	PD
cg07459121	-0.2334	1.74087	-4.04896	0.00014	0.032771	0.92026	PD
cg21533994	0.225476	-2.55909	4.048656	0.00014	0.032792	0.920002	PD
cg08298675	-0.4629	3.375232	-4.04861	0.00014	0.032792	0.917948	PD
cg20887444	0.206796	3.581503	4.048439	0.00014	0.032802	0.914831	PD

cg06548827	-0.35331	2.808231	-4.04818	0.000141	0.032822	0.918549	PD
cg13293988	-0.22504	2.723222	-4.04733	0.000141	0.032908	0.911044	PD
cg05759309	-0.12308	4.245465	-4.0472	0.000141	0.032913	0.915062	PD
cg27102691	-0.16453	3.103454	-4.04707	0.000141	0.032919	0.914015	PD
cg13783387	-0.21468	3.828909	-4.04615	0.000141	0.033013	0.911667	PD
cg18480137	-0.19718	3.860637	-4.04583	0.000142	0.033036	0.908946	PD
cg07204165	-0.17899	3.649873	-4.04578	0.000142	0.033036	0.908359	PD
cg05199282	0.152741	-0.6282	4.045609	0.000142	0.033047	0.906797	PD
cg04032292	0.188834	1.855699	4.045172	0.000142	0.033087	0.908352	PD
cg22852353	0.264405	3.556249	4.045063	0.000142	0.03309	0.906567	PD
cg01738411	-0.30934	3.819002	-4.0449	0.000142	0.033099	0.905755	PD
cg17635903	0.258585	-2.53613	4.044747	0.000142	0.0331	0.905371	PD
cg00716368	-0.2202	3.236826	-4.04473	0.000142	0.0331	0.907001	PD
cg15715690	0.307658	3.867033	4.04417	0.000142	0.033154	0.900983	PD
cg18376999	0.210222	3.308903	4.043968	0.000143	0.033168	0.904833	PD
cg07877725	-0.18492	3.1117	-4.04338	0.000143	0.033225	0.901613	PD
cg17937061	-0.49636	3.291348	-4.04315	0.000143	0.033242	0.902803	PD
cg08409553	-0.53221	4.944057	-4.04284	0.000143	0.033267	0.897478	PD
cg23578720	0.250164	4.327221	4.04269	0.000143	0.033276	0.89783	PD
cg13074273	-0.13548	3.03852	-4.04237	0.000143	0.033302	0.896709	PD
cg03024135	-0.22567	3.438802	-4.04203	0.000143	0.033332	0.896444	PD
cg03958956	-0.21551	6.38166	-4.0419	0.000144	0.033337	0.894193	PD
cg18430990	0.248652	-4.21466	4.041768	0.000144	0.033343	0.895101	PD
cg20685792	-0.18735	2.85251	-4.04052	0.000144	0.033475	0.894134	PD
cg04009254	0.118085	-4.44325	4.040351	0.000144	0.033484	0.893411	PD
cg01271034	-0.31719	1.339281	-4.04029	0.000144	0.033484	0.890351	PD
cg01640711	0.142234	2.986867	4.039158	0.000145	0.033604	0.888331	PD
cg03721362	0.258148	-5.80063	4.038747	0.000145	0.033641	0.886526	PD
cg12528981	-0.22382	6.545877	-4.03805	0.000145	0.033712	0.884965	PD
cg00048764	-0.18854	3.819654	-4.03788	0.000146	0.033723	0.883114	PD
cg02110903	-0.17823	4.409686	-4.03724	0.000146	0.033786	0.88095	PD

cg11384510	0.46751	3.10307	4.036594	0.000146	0.033851	0.881334	PD
cg23248324	0.234907	-4.97538	4.035993	0.000146	0.033911	0.879993	PD
cg27271738	-0.25579	2.974492	-4.03575	0.000147	0.03393	0.876478	PD
cg09326084	-0.1536	4.126567	-4.03519	0.000147	0.033976	0.875361	PD
cg03637815	0.123402	-4.92726	4.035126	0.000147	0.033976	0.878787	PD
cg10862960	-0.47763	3.48116	-4.0351	0.000147	0.033976	0.878923	PD
cg10351869	-0.23289	3.059797	-4.03503	0.000147	0.033976	0.87787	PD
cg09384035	-0.23584	3.007402	-4.03494	0.000147	0.033977	0.876136	PD
cg09224753	0.234832	-3.85881	4.034839	0.000147	0.033978	0.877376	PD
cg08159493	-0.407	2.890634	-4.03473	0.000147	0.033978	0.876333	PD
cg08814170	-0.23624	2.802938	-4.03469	0.000147	0.033978	0.875652	PD
cg21771200	-0.33367	-5.2577	-4.0345	0.000147	0.033991	0.873633	PD
cg20322554	-0.16762	3.962525	-4.03423	0.000147	0.034013	0.87563	PD
cg03688963	0.128458	-5.67002	4.03393	0.000147	0.034038	0.87326	PD
cg01881590	-0.21226	3.730339	-4.033	0.000148	0.034137	0.872237	PD
cg08441455	0.296631	3.26411	4.032682	0.000148	0.034164	0.869779	PD
cg22512847	-0.14306	3.771725	-4.03255	0.000148	0.034167	0.86649	PD
cg12138608	-0.3978	3.909024	-4.03249	0.000148	0.034167	0.866772	PD
cg12798564	-0.20014	2.748217	-4.03223	0.000148	0.034182	0.868147	PD
cg12011688	-0.18477	3.223636	-4.03219	0.000148	0.034182	0.865014	PD
cg01049933	0.346819	2.617788	4.032123	0.000148	0.034182	0.865439	PD
cg10615591	-0.1708	1.427666	-4.03172	0.000149	0.034211	0.867052	PD
cg21128963	-0.20493	2.477862	-4.03165	0.000149	0.034211	0.867895	PD
cg16078269	-0.25248	2.856167	-4.03161	0.000149	0.034211	0.864996	PD
cg01175605	-0.17601	4.11733	-4.03156	0.000149	0.034211	0.863594	PD
cg09349613	-0.40669	2.479946	-4.0311	0.000149	0.034255	0.866833	PD
cg07264401	-0.88195	4.867128	-4.03088	0.000149	0.034271	0.862484	PD
cg02262199	-0.20053	2.932179	-4.03058	0.000149	0.034296	0.861942	PD
cg20536289	-0.23936	3.63582	-4.03023	0.000149	0.034317	0.861852	PD
cg15864349	-0.19747	3.771911	-4.03019	0.000149	0.034317	0.861726	PD
cg17345620	-0.25115	2.830245	-4.03016	0.000149	0.034317	0.861258	PD

cg04626996	0.107261	-5.2168	4.029581	0.00015	0.034376	0.862139	PD
cg01738200	-0.17033	3.246555	-4.02918	0.00015	0.034414	0.858503	PD
cg17640486	-0.18886	3.742912	-4.02896	0.00015	0.03443	0.855143	PD
cg24980040	-0.1838	5.047085	-4.02825	0.00015	0.034503	0.853928	PD
cg14995148	0.175033	-0.52156	4.027988	0.000151	0.034525	0.855492	PD
cg09626833	-0.27989	3.884471	-4.02758	0.000151	0.034563	0.854881	PD
cg02867448	-0.17293	2.923317	-4.02678	0.000151	0.034648	0.852274	PD
cg02252870	-0.21807	3.263363	-4.02609	0.000151	0.034719	0.849517	PD
cg19183248	-0.2072	3.339972	-4.02576	0.000152	0.034749	0.846981	PD
cg01636248	-0.19734	2.230315	-4.02566	0.000152	0.034752	0.848753	PD
cg04199720	-0.24578	3.666941	-4.02519	0.000152	0.034798	0.847423	PD
cg13740936	-0.24376	2.587556	-4.02479	0.000152	0.03483	0.844302	PD
cg08438920	-0.21378	3.33155	-4.02475	0.000152	0.03483	0.845857	PD
cg05435349	-0.18073	4.030912	-4.02445	0.000152	0.034856	0.842157	PD
cg14411137	0.24595	3.890634	4.024201	0.000152	0.034877	0.843797	PD
cg01546873	-0.13479	4.115268	-4.02397	0.000153	0.034893	0.841415	PD
cg06739590	-0.25357	3.565283	-4.0239	0.000153	0.034893	0.841425	PD
cg08826925	-0.24747	3.686796	-4.02361	0.000153	0.034919	0.842068	PD
cg09577851	-0.13739	3.610969	-4.02301	0.000153	0.034969	0.841667	PD
cg26467673	-0.16741	4.694467	-4.02296	0.000153	0.034969	0.839511	PD
cg01512719	-0.12469	4.669574	-4.02294	0.000153	0.034969	0.840301	PD
cg00256226	-0.17449	4.982796	-4.02251	0.000153	0.035011	0.839518	PD
cg14725880	0.199235	-0.09187	4.022424	0.000153	0.035011	0.837228	PD
cg16423843	-0.21564	3.163498	-4.02233	0.000153	0.035011	0.838246	PD
cg02387491	-0.26749	4.148128	-4.02227	0.000153	0.035011	0.836083	PD
cg10846328	-0.2035	2.835597	-4.02211	0.000154	0.035021	0.835011	PD
cg08433912	-0.17618	3.021037	-4.02124	0.000154	0.035105	0.835509	PD
cg09559324	-0.15898	5.418372	-4.02123	0.000154	0.035105	0.835574	PD
cg14549869	-0.32605	4.51818	-4.02116	0.000154	0.035105	0.834392	PD
cg17819066	-0.1543	2.450626	-4.02096	0.000154	0.035113	0.832285	PD
cg16533314	-0.31061	3.80902	-4.02093	0.000154	0.035113	0.83488	PD

cg10794086	0.17196	-0.25333	4.020632	0.000154	0.03514	0.833175	PD
cg24791025	0.116661	-4.39776	4.019326	0.000155	0.035286	0.831253	PD
cg23939166	-0.24809	-4.42352	-4.01882	0.000155	0.035338	0.8262	PD
cg20134916	0.240114	-4.79591	4.018314	0.000156	0.035388	0.825667	PD
cg06903465	-0.1128	3.99672	-4.01759	0.000156	0.035466	0.824284	PD
cg22649546	0.111829	-1.14994	4.017189	0.000156	0.035505	0.821174	PD
cg21860360	0.619101	3.119298	4.017072	0.000156	0.035509	0.823815	PD
cg25873514	-1.15371	3.235098	-4.01692	0.000156	0.035519	0.824012	PD
cg14536764	-0.28207	3.761692	-4.0164	0.000157	0.035562	0.819614	PD
cg25957114	0.185002	4.103282	4.016263	0.000157	0.035562	0.81839	PD
cg09492858	0.198723	4.387485	4.016252	0.000157	0.035562	0.816245	PD
cg08774625	-0.16407	3.584946	-4.01623	0.000157	0.035562	0.818005	PD
cg00738947	-0.2441	2.630228	-4.01616	0.000157	0.035562	0.820996	PD
cg02488258	-0.19939	2.875581	-4.01554	0.000157	0.035627	0.816962	PD
cg04972968	0.389865	-3.79721	4.015334	0.000157	0.035642	0.815915	PD
cg00451227	-0.18779	3.921205	-4.01505	0.000157	0.035666	0.81479	PD
cg04196512	-0.21979	3.212422	-4.01498	0.000157	0.035666	0.815011	PD
cg11476241	-0.18084	1.926632	-4.01473	0.000157	0.035687	0.815502	PD
cg14099160	-0.24946	2.884862	-4.01459	0.000157	0.035688	0.813624	PD
cg03887265	0.322571	2.89166	4.014564	0.000158	0.035688	0.813773	PD
cg03987842	-0.24789	4.401733	-4.0144	0.000158	0.03569	0.816148	PD
cg23304464	0.656904	3.430235	4.014354	0.000158	0.03569	0.814883	PD
cg14551114	-0.14903	2.043739	-4.01425	0.000158	0.03569	0.815834	PD
cg11513352	-0.41833	-6.62922	-4.01423	0.000158	0.03569	0.811152	PD
cg13831540	0.261774	-1.59871	4.013697	0.000158	0.035745	0.812078	PD
cg22887609	-0.25556	3.188244	-4.01334	0.000158	0.035778	0.811817	PD
cg04045436	-0.18319	3.075292	-4.01325	0.000158	0.03578	0.811133	PD
cg13367148	-0.17439	4.081196	-4.01305	0.000158	0.035795	0.807107	PD
cg24974756	-0.15548	5.136921	-4.01202	0.000159	0.035909	0.803895	PD
cg10790181	-0.13344	4.036747	-4.01157	0.000159	0.035956	0.804667	PD
cg05807413	-0.133	3.840689	-4.01121	0.000159	0.035976	0.806548	PD

cg01780928	-0.26076	4.011093	-4.01121	0.000159	0.035976	0.801246	PD
cg18857892	-0.19697	3.782778	-4.01116	0.000159	0.035976	0.802634	PD
cg00269725	-0.24899	2.307086	-4.0104	0.00016	0.036059	0.801199	PD
cg09834061	-0.1383	3.237707	-4.01007	0.00016	0.03609	0.803415	PD
cg26426147	0.27712	3.769885	4.009871	0.00016	0.036105	0.801427	PD
cg16078412	-0.23063	3.638027	-4.00965	0.00016	0.036116	0.79732	PD
cg02140072	-0.21821	3.228837	-4.00962	0.00016	0.036116	0.799816	PD
cg25865108	-0.20901	3.340897	-4.00912	0.00016	0.036167	0.794236	PD
cg04482597	-0.49641	-5.21732	-4.00844	0.000161	0.036241	0.797923	PD
cg18810937	-0.23825	3.002047	-4.00792	0.000161	0.036295	0.793113	PD
cg08651346	0.707162	-4.77881	4.007397	0.000161	0.03635	0.795038	PD
cg02631791	-0.21215	2.664232	-4.007	0.000162	0.036388	0.793708	PD
cg02251859	-0.34876	-4.77977	-4.00693	0.000162	0.036388	0.790438	PD
cg04830773	0.214409	2.499399	4.006838	0.000162	0.03639	0.789983	PD
cg23665668	-0.19361	3.15545	-4.00662	0.000162	0.036407	0.788754	PD
cg09283558	-0.15995	3.970546	-4.00649	0.000162	0.036414	0.788357	PD
cg13462275	-0.29078	3.982455	-4.00571	0.000162	0.0365	0.788622	PD
cg21887544	-0.45339	4.932618	-4.00555	0.000162	0.03651	0.78602	PD
cg21743826	0.258793	2.219421	4.005339	0.000163	0.036527	0.788768	PD
cg16672904	-0.24163	3.712917	-4.00475	0.000163	0.036586	0.782989	PD
cg24345856	0.743307	2.641456	4.004705	0.000163	0.036586	0.785779	PD
cg11439822	-0.21687	3.938108	-4.00443	0.000163	0.03661	0.783648	PD
cg03867746	-0.16653	2.771537	-4.00416	0.000163	0.036634	0.785516	PD
cg22504267	-0.20218	3.87086	-4.00394	0.000163	0.036648	0.783216	PD
cg12734151	0.23647	4.369083	4.003887	0.000163	0.036648	0.779325	PD
cg11294620	-0.4944	3.648035	-4.00355	0.000163	0.036681	0.783107	PD
cg02151754	0.187065	-6.54841	4.003227	0.000164	0.036711	0.781845	PD
cg00659811	-0.19442	2.928291	-4.00288	0.000164	0.036744	0.781701	PD
cg18197973	-0.26343	3.536845	-4.00274	0.000164	0.036752	0.78007	PD
cg11076275	-0.16467	2.487044	-4.00255	0.000164	0.03676	0.779442	PD
cg26281303	-0.27497	3.67929	-4.00252	0.000164	0.03676	0.780621	PD

cg05342655	-0.25362	3.646383	-4.0024	0.000164	0.036764	0.776825	PD
cg13863099	-0.3696	3.093756	-4.00216	0.000164	0.036781	0.775401	PD
cg06178754	-0.16408	4.764774	-4.00205	0.000164	0.036781	0.773737	PD
cg20492230	-0.4752	4.377009	-4.00203	0.000164	0.036781	0.778566	PD
cg27335233	-0.16102	3.663323	-4.0013	0.000165	0.036863	0.775118	PD
cg13924207	0.281803	-2.40884	4.001089	0.000165	0.036879	0.773857	PD
cg24040751	0.207575	0.270333	4.000885	0.000165	0.036885	0.769172	PD
cg05289920	-0.42681	-2.83761	-4.00081	0.000165	0.036885	0.773126	PD
cg21848624	-0.54498	2.717899	-4.00081	0.000165	0.036885	0.775161	PD
cg06295987	-0.26577	4.715113	-4.00035	0.000165	0.036933	0.768413	PD
cg25624115	-0.21005	2.217862	-4.00002	0.000165	0.036965	0.770705	PD
cg14934543	-0.36773	3.992184	-3.99985	0.000166	0.036976	0.76897	PD
cg05691098	-0.1634	4.268318	-3.99942	0.000166	0.037019	0.766925	PD
cg18822951	-0.30363	2.102994	-3.99929	0.000166	0.037026	0.770209	PD
cg04392370	-0.22243	3.196358	-3.99876	0.000166	0.037083	0.767872	PD
cg08802984	-0.30984	3.820146	-3.99864	0.000166	0.037088	0.768661	PD
cg17535559	-0.197	1.861042	-3.99851	0.000166	0.037094	0.76666	PD
cg18277467	-0.29166	4.029482	-3.99844	0.000166	0.037094	0.765128	PD
cg23148094	0.201731	3.905264	3.997925	0.000167	0.037147	0.762593	PD
cg13915353	0.284782	3.547175	3.997858	0.000167	0.037147	0.762208	PD
cg22332045	0.181727	4.041384	3.997774	0.000167	0.037148	0.763013	PD
cg02758410	-0.26877	2.31278	-3.99756	0.000167	0.037165	0.761561	PD
cg02847730	-0.36114	4.104076	-3.99742	0.000167	0.037173	0.758472	PD
cg08326417	-0.29074	3.449186	-3.99689	0.000167	0.037221	0.758876	PD
cg23553867	-0.18191	3.456191	-3.99688	0.000167	0.037221	0.759315	PD
cg14526718	0.163797	-3.84556	3.996743	0.000167	0.037229	0.757405	PD
cg13566468	-0.3687	3.573324	-3.99649	0.000167	0.037251	0.760563	PD
cg10530680	0.221662	4.082041	3.996409	0.000167	0.037251	0.757121	PD
cg06920022	-0.17851	3.496565	-3.99624	0.000168	0.037253	0.759272	PD
cg18908238	-0.28077	3.645017	-3.99624	0.000168	0.037253	0.757114	PD
cg11267055	-0.23099	2.931572	-3.99516	0.000168	0.037374	0.755429	PD

cg00363486	0.268438	-3.5916	3.995127	0.000168	0.037374	0.754615	PD
cg26455973	0.204319	2.796973	3.995015	0.000168	0.037378	0.755842	PD
cg06611375	0.119155	-4.32188	3.994868	0.000168	0.037386	0.752919	PD
cg20231299	-0.41931	2.091323	-3.99479	0.000168	0.037386	0.750337	PD
cg06235847	-0.16096	-3.55916	-3.99453	0.000169	0.037402	0.752824	PD
cg05400405	-0.12878	3.997178	-3.99451	0.000169	0.037402	0.752071	PD
cg03483709	-0.13293	0.080901	-3.99424	0.000169	0.037427	0.752206	PD
cg16245035	-0.12218	6.418043	-3.99405	0.000169	0.037434	0.75039	PD
cg05585321	-0.27741	2.305893	-3.99403	0.000169	0.037434	0.754543	PD
cg09312895	-0.15487	2.968208	-3.99381	0.000169	0.037434	0.748015	PD
cg02571595	-0.17531	4.087121	-3.99375	0.000169	0.037434	0.749766	PD
cg05443459	0.178718	0.801262	3.993744	0.000169	0.037434	0.753601	PD
cg16445596	-0.16849	2.651239	-3.99372	0.000169	0.037434	0.748735	PD
cg22186557	-0.19803	2.63899	-3.99326	0.000169	0.037482	0.749158	PD
cg12050434	-0.1427	0.763418	-3.99289	0.000169	0.037519	0.74949	PD
cg17386710	-0.21957	2.661295	-3.99278	0.00017	0.037523	0.747105	PD
cg18358952	-0.125	1.406676	-3.99255	0.00017	0.037543	0.750537	PD
cg17518290	-0.21375	3.188951	-3.9922	0.00017	0.037577	0.744837	PD
cg16814646	0.134154	-4.62013	3.991432	0.00017	0.037657	0.740421	PD
cg22228134	-0.29443	2.600763	-3.99142	0.00017	0.037657	0.743493	PD
cg23566952	-0.26105	3.488722	-3.99117	0.00017	0.037679	0.741331	PD
cg05102425	0.124819	-0.84032	3.990288	0.000171	0.037781	0.743121	PD
cg12687426	-0.33307	4.059314	-3.99006	0.000171	0.0378	0.743241	PD
cg13822566	-0.15412	3.365892	-3.98959	0.000171	0.037844	0.735546	PD
cg07588018	0.232036	2.330592	3.989568	0.000171	0.037844	0.738605	PD
cg27301180	-0.15557	3.916176	-3.98919	0.000172	0.037882	0.735247	PD
cg20679581	-0.16562	2.194431	-3.98832	0.000172	0.037984	0.735079	PD
cg02832162	-0.2244	3.650747	-3.98798	0.000172	0.038018	0.733422	PD
cg09157668	0.161819	-5.4472	3.987833	0.000172	0.038026	0.732763	PD
cg04379124	-0.23603	3.87698	-3.98746	0.000173	0.038065	0.732258	PD
cg15250295	-0.15531	4.096315	-3.98712	0.000173	0.038098	0.729838	PD

cg03579474	-0.17432	3.84072	-3.9863	0.000173	0.038194	0.729805	PD
cg26716823	-0.21679	3.782189	-3.98616	0.000173	0.038201	0.728798	PD
cg16405582	0.288308	-4.18028	3.986021	0.000173	0.038201	0.724898	PD
cg16364344	0.181965	0.462073	3.986014	0.000173	0.038201	0.727655	PD
cg04141520	-0.20709	3.822856	-3.98536	0.000174	0.038275	0.723707	PD
cg10313337	-0.18939	2.496078	-3.98504	0.000174	0.038307	0.726095	PD
cg12094029	-0.16954	2.758094	-3.98481	0.000174	0.038327	0.724924	PD
cg17723279	0.270386	3.483369	3.984644	0.000174	0.038338	0.724252	PD
cg09362543	-0.13118	4.927394	-3.98423	0.000175	0.038381	0.724283	PD
cg04609640	-0.17983	2.729553	-3.98378	0.000175	0.038427	0.719916	PD
cg02452491	-0.1532	1.681968	-3.98373	0.000175	0.038427	0.71879	PD
cg11829191	-0.14701	3.883494	-3.98358	0.000175	0.038436	0.723833	PD
cg20829834	-0.23072	2.352931	-3.98288	0.000175	0.038516	0.716153	PD
cg13229487	-0.39585	3.332394	-3.98194	0.000176	0.038616	0.718677	PD
cg09592133	-0.22501	3.527508	-3.98192	0.000176	0.038616	0.71279	PD
cg17722719	0.226332	-5.9962	3.98189	0.000176	0.038616	0.716318	PD
cg11481460	0.147026	2.424149	3.981796	0.000176	0.038618	0.711807	PD
cg01427750	-0.20463	3.491081	-3.98132	0.000176	0.03867	0.712049	PD
cg24548341	0.1183	3.508273	3.981178	0.000176	0.038672	0.710263	PD
cg25021893	-0.24978	2.726001	-3.98115	0.000176	0.038672	0.71127	PD
cg11213278	0.188131	0.180672	3.980971	0.000176	0.038679	0.712639	PD
cg20327845	-0.16658	1.029792	-3.98095	0.000176	0.038679	0.712354	PD
cg17481012	0.454019	3.695554	3.980582	0.000177	0.038716	0.714587	PD
cg08353090	-0.23637	3.168318	-3.98043	0.000177	0.038726	0.709269	PD
cg24694947	-0.19304	4.099737	-3.98016	0.000177	0.038751	0.7127	PD
cg08495005	0.113004	-0.76691	3.979841	0.000177	0.038781	0.709664	PD
cg25871427	0.222778	3.576241	3.979778	0.000177	0.038781	0.70566	PD
cg15881372	0.174288	1.294008	3.979501	0.000177	0.038808	0.71007	PD
cg20331137	-0.28468	4.130682	-3.97939	0.000177	0.038812	0.703482	PD
cg22526139	-0.0975	0.664346	-3.97903	0.000178	0.038849	0.706727	PD
cg20216802	0.117445	-4.52227	3.978275	0.000178	0.038938	0.704679	PD

cg14266361	0.251755	3.268391	3.977279	0.000179	0.039059	0.697493	PD
cg11900657	0.123307	-4.15321	3.976766	0.000179	0.039117	0.701717	PD
cg18776945	-0.22947	2.00676	-3.97665	0.000179	0.039121	0.699685	PD
cg21641817	0.258483	-3.15623	3.976579	0.000179	0.039121	0.700765	PD
cg02166236	-0.25718	3.23076	-3.97649	0.000179	0.039123	0.69661	PD
cg07118245	0.287995	2.201838	3.976122	0.000179	0.039161	0.697844	PD
cg12522833	0.253098	3.260147	3.975461	0.00018	0.039238	0.699336	PD
cg20770175	-0.21066	3.370654	-3.97504	0.00018	0.039285	0.694237	PD
cg23742233	-0.27373	-1.56271	-3.97467	0.00018	0.039322	0.694219	PD
cg22812133	-0.27146	2.393621	-3.97455	0.00018	0.039322	0.691461	PD
cg01532713	-0.15743	3.774311	-3.97452	0.00018	0.039322	0.696656	PD
cg23716636	-0.15471	3.082527	-3.9743	0.00018	0.039337	0.690499	PD
cg13139542	0.29666	4.292607	3.974255	0.000181	0.039337	0.691656	PD
cg21655931	-0.21629	3.812258	-3.97401	0.000181	0.039344	0.694184	PD
cg26803596	-0.26576	0.149384	-3.97395	0.000181	0.039344	0.688037	PD
cg21265210	-0.21575	3.982812	-3.97393	0.000181	0.039344	0.687958	PD
cg09622653	-0.14214	3.751237	-3.9739	0.000181	0.039344	0.692952	PD
cg17341113	-0.16228	6.987	-3.9737	0.000181	0.039361	0.689984	PD
cg13873920	-0.31735	-5.94362	-3.97332	0.000181	0.03939	0.684058	PD
cg26108524	-0.16078	4.081667	-3.97326	0.000181	0.03939	0.692485	PD
cg03072082	0.535282	-4.08546	3.973203	0.000181	0.03939	0.684745	PD
cg10229937	-0.1837	4.330849	-3.97317	0.000181	0.03939	0.692321	PD
cg03266059	0.686006	-4.67314	3.972701	0.000181	0.039442	0.688773	PD
cg04498014	0.236029	-2.67508	3.972534	0.000182	0.039454	0.689281	PD
cg02536825	-0.20824	2.761468	-3.97236	0.000182	0.039467	0.687155	PD
cg15682239	0.687957	-4.90812	3.972216	0.000182	0.039476	0.686135	PD
cg01703858	-0.22509	1.454968	-3.97201	0.000182	0.03948	0.681751	PD
cg12271800	-0.20393	3.065742	-3.97198	0.000182	0.03948	0.680742	PD
cg24569116	-0.27709	2.922992	-3.97194	0.000182	0.03948	0.685846	PD
cg22726155	0.161793	0.506696	3.971887	0.000182	0.03948	0.687086	PD
cg15837563	0.143771	-0.20125	3.971193	0.000182	0.039562	0.679052	PD

cg13880275	-0.35171	-4.47064	-3.97044	0.000183	0.039652	0.684019	PD
cg23322933	0.243086	-1.46374	3.970198	0.000183	0.039675	0.677555	PD
cg13427473	-0.28493	3.286486	-3.96815	0.000184	0.039938	0.671096	PD
cg11525297	-0.17211	2.753488	-3.96801	0.000184	0.039947	0.670794	PD
cg06974755	0.139424	-4.728	3.967847	0.000184	0.039959	0.675045	PD
cg07346751	-0.17166	2.766839	-3.96774	0.000185	0.039963	0.671281	PD
cg13955836	-0.17832	2.517008	-3.9674	0.000185	0.039999	0.668779	PD
cg02339015	-0.33865	4.851436	-3.9671	0.000185	0.040029	0.671134	PD
cg19705352	0.29189	4.605178	3.966902	0.000185	0.040046	0.671108	PD
cg12302182	0.23387	3.524152	3.966673	0.000185	0.040061	0.668656	PD
cg27647628	-0.1617	4.220954	-3.96662	0.000185	0.040061	0.666576	PD
cg00454768	-0.75846	3.241561	-3.96656	0.000185	0.040061	0.671329	PD
cg11504176	-0.12828	1.241239	-3.96633	0.000185	0.040082	0.670261	PD
cg17590179	-0.18414	3.222059	-3.96613	0.000186	0.040088	0.665126	PD
cg14388237	-0.24097	-4.00354	-3.96604	0.000186	0.040088	0.67121	PD
cg06999323	0.23077	-4.98373	3.966026	0.000186	0.040088	0.664344	PD
cg11610460	-0.17696	2.143595	-3.96598	0.000186	0.040088	0.668868	PD
cg08362206	-0.26649	3.436697	-3.96582	0.000186	0.040094	0.665311	PD
cg12000509	-0.20651	3.107194	-3.96573	0.000186	0.040094	0.66785	PD
cg19716218	-0.19659	2.796572	-3.96564	0.000186	0.040094	0.662873	PD
cg05899869	-0.19849	3.108074	-3.96563	0.000186	0.040094	0.665648	PD
cg13132307	-0.1079	6.046147	-3.96554	0.000186	0.040096	0.663216	PD
cg05944635	-0.18735	2.2667	-3.96531	0.000186	0.040117	0.665882	PD
cg08703465	-0.22794	2.170989	-3.96482	0.000186	0.040165	0.661608	PD
cg15778745	-0.20998	-6.12369	-3.96481	0.000186	0.040165	0.659908	PD
cg02934718	-0.35424	4.822922	-3.96428	0.000187	0.040226	0.660866	PD
cg05879324	-0.21458	3.776836	-3.96356	0.000187	0.040313	0.660427	PD
cg01767118	0.169334	-5.2287	3.962996	0.000187	0.040369	0.659599	PD
cg12786023	-0.42319	3.143857	-3.96295	0.000188	0.040369	0.657477	PD
cg12063639	-0.42687	4.131177	-3.96287	0.000188	0.040369	0.655974	PD
cg18739031	-0.1685	3.682455	-3.96285	0.000188	0.040369	0.656505	PD

cg25900441	-0.23414	4.428656	-3.96261	0.000188	0.040391	0.65812	PD
cg12601456	0.249202	4.017241	3.96234	0.000188	0.040417	0.656728	PD
cg05700528	0.138637	1.145774	3.962048	0.000188	0.040447	0.656257	PD
cg03297418	-0.40124	-4.50382	-3.96187	0.000188	0.040453	0.656934	PD
cg14210287	0.358119	-4.48733	3.961856	0.000188	0.040453	0.651876	PD
cg00764771	0.193045	-1.59889	3.96095	0.000189	0.040566	0.6521	PD
cg16535080	-0.19254	2.735143	-3.96059	0.000189	0.040605	0.65496	PD
cg13602967	-0.17705	2.908572	-3.96032	0.000189	0.040624	0.649479	PD
cg08897368	-0.13156	4.200127	-3.9603	0.000189	0.040624	0.648344	PD
cg19413958	-0.13827	3.847619	-3.96015	0.000189	0.040627	0.652136	PD
cg13636895	-0.23248	3.010917	-3.96012	0.000189	0.040627	0.649384	PD
cg27577424	-0.30567	3.774918	-3.95946	0.00019	0.040708	0.646768	PD
cg21109279	0.121397	-0.80315	3.959069	0.00019	0.040751	0.646612	PD
cg20013563	-0.32657	4.283365	-3.95884	0.00019	0.040763	0.647694	PD
cg21978924	0.143869	-4.60706	3.958833	0.00019	0.040763	0.64621	PD
cg14161107	-0.20548	2.431179	-3.95851	0.00019	0.040797	0.643819	PD
cg26759925	-0.17854	3.958254	-3.95839	0.00019	0.040799	0.645264	PD
cg21226754	-0.28456	2.717866	-3.95834	0.00019	0.040799	0.647184	PD
cg02129337	2.633088	4.197926	3.958036	0.000191	0.040827	0.639099	PD
cg20237294	-0.32231	3.456683	-3.95799	0.000191	0.040827	0.641813	PD
cg25730351	-0.47561	-3.93909	-3.95755	0.000191	0.040877	0.638008	PD
cg03585096	-0.20579	3.121242	-3.95729	0.000191	0.040902	0.640552	PD
cg00890331	-0.20747	2.905051	-3.95721	0.000191	0.040903	0.640856	PD
cg19777991	-0.15576	3.890695	-3.95695	0.000191	0.040929	0.641334	PD
cg00597091	-0.29401	2.954201	-3.95665	0.000192	0.040953	0.636105	PD
cg10955208	-0.15233	4.499844	-3.95662	0.000192	0.040953	0.635968	PD
cg18954658	-0.64303	2.61353	-3.9564	0.000192	0.040963	0.63755	PD
cg05146499	-0.27704	3.278412	-3.9564	0.000192	0.040963	0.639218	PD
cg11912668	-0.25169	2.436919	-3.95632	0.000192	0.040963	0.638265	PD
cg06031301	0.288405	3.173202	3.956133	0.000192	0.040979	0.636352	PD
cg27384338	-0.14434	4.007236	-3.95581	0.000192	0.041012	0.632624	PD

cg17874034	-0.15978	4.176374	-3.9555	0.000192	0.041046	0.637991	PD
cg04453437	-0.13995	3.879373	-3.95473	0.000193	0.041141	0.635183	PD
cg23767435	-0.17756	3.47046	-3.95412	0.000193	0.041189	0.631938	PD
cg06972389	-0.21874	4.618131	-3.95412	0.000193	0.041189	0.634181	PD
cg19237421	0.195069	-2.6919	3.954112	0.000193	0.041189	0.632611	PD
cg07641839	-0.12314	5.159566	-3.95396	0.000193	0.041189	0.629018	PD
cg02078199	-0.79647	2.360915	-3.95395	0.000193	0.041189	0.634178	PD
cg25814612	-0.13967	3.938603	-3.95388	0.000193	0.041189	0.630499	PD
cg01119680	0.171583	-2.62465	3.953859	0.000193	0.041189	0.63085	PD
cg04901779	0.159614	-0.86136	3.953731	0.000193	0.041192	0.629926	PD
cg16896687	-0.16264	3.641834	-3.95369	0.000193	0.041192	0.62808	PD
cg19160264	0.270184	3.905627	3.953601	0.000194	0.041194	0.631138	PD
cg04130851	0.135404	-1.16842	3.953179	0.000194	0.041242	0.624868	PD
cg19014904	-0.17014	3.320516	-3.95294	0.000194	0.041258	0.630603	PD
cg18776876	-0.20362	2.530017	-3.95288	0.000194	0.041258	0.62902	PD
cg20054739	0.22425	-2.09991	3.952809	0.000194	0.041258	0.627417	PD
cg24145109	-0.42446	2.552421	-3.95276	0.000194	0.041258	0.631745	PD
cg06077426	-0.14625	3.552775	-3.95251	0.000194	0.041281	0.623508	PD
cg14488913	-0.5787	3.085571	-3.95245	0.000194	0.041281	0.630194	PD
cg01001759	0.120338	-1.08463	3.952004	0.000195	0.041333	0.625857	PD
cg08026124	0.180296	-0.22414	3.951588	0.000195	0.04138	0.624266	PD
cg07130392	-0.23071	3.11705	-3.95133	0.000195	0.041406	0.623266	PD
cg11194597	-0.26241	2.298803	-3.95117	0.000195	0.041417	0.621517	PD
cg10726559	-0.13315	4.937142	-3.95089	0.000195	0.041446	0.620741	PD
cg06167221	-0.28019	2.792139	-3.9507	0.000195	0.041462	0.622017	PD
cg14829953	-0.25886	4.234182	-3.95046	0.000196	0.041485	0.618106	PD
cg08603382	-0.23471	4.720775	-3.9489	0.000197	0.041693	0.613774	PD
cg11600511	-0.18403	3.020554	-3.94756	0.000197	0.041857	0.611609	PD
cg02405213	-0.36829	2.505843	-3.94756	0.000197	0.041857	0.615029	PD
cg07046896	-0.17047	2.882191	-3.94747	0.000198	0.041857	0.613832	PD
cg07199524	0.131541	-4.01521	3.947369	0.000198	0.041857	0.607786	PD

cg06329316	-0.21177	2.814253	-3.94736	0.000198	0.041857	0.611748	PD
cg23884076	-0.28732	2.457998	-3.94705	0.000198	0.04187	0.608186	PD
cg26401732	-0.22089	4.80849	-3.94705	0.000198	0.04187	0.608351	PD
cg25137365	-0.26217	5.896598	-3.94704	0.000198	0.04187	0.611163	PD
cg21918736	-0.19854	3.590338	-3.94677	0.000198	0.041895	0.613566	PD
cg27080206	-0.26608	3.765715	-3.94671	0.000198	0.041895	0.606749	PD
cg19981301	-0.20661	2.733972	-3.94645	0.000198	0.041918	0.606656	PD
cg03306486	-0.21743	1.710998	-3.9464	0.000198	0.041918	0.607419	PD
cg07584230	0.136607	-0.42497	3.946055	0.000198	0.041956	0.60857	PD
cg09449123	-0.18162	2.47985	-3.94564	0.000199	0.042002	0.60488	PD
cg15964593	0.313675	3.493906	3.945585	0.000199	0.042002	0.60458	PD
cg20006652	-0.34893	2.577196	-3.94476	0.000199	0.042108	0.601796	PD
cg20032056	-0.21583	3.079542	-3.94467	0.000199	0.04211	0.604696	PD
cg14522790	0.267332	3.76919	3.944465	0.0002	0.042128	0.603417	PD
cg16443633	-0.15055	3.361948	-3.94425	0.0002	0.042149	0.602444	PD
cg15681853	-0.22118	1.834023	-3.94395	0.0002	0.04218	0.603572	PD
cg10625367	-0.1833	5.249531	-3.94378	0.0002	0.042194	0.601225	PD
cg27269947	-0.21376	2.724985	-3.94314	0.0002	0.042274	0.598627	PD
cg07219494	-0.25843	2.206965	-3.94224	0.000201	0.042392	0.594251	PD
cg16546758	-0.44463	2.569322	-3.94166	0.000201	0.042463	0.595169	PD
cg19137581	-0.23074	3.703481	-3.94098	0.000202	0.04255	0.589551	PD
cg18837576	-0.26692	4.083873	-3.94079	0.000202	0.042566	0.588099	PD
cg04845171	-0.22528	3.919431	-3.94065	0.000202	0.042576	0.591094	PD
cg09792707	-0.14525	3.294842	-3.94032	0.000202	0.042608	0.590937	PD
cg04636811	-0.20632	3.297591	-3.94028	0.000202	0.042608	0.589111	PD
cg02297484	0.205713	4.355132	3.940129	0.000202	0.042619	0.584531	PD
cg15009895	-0.12082	2.393593	-3.93997	0.000203	0.042627	0.591059	PD
cg15633369	-0.20805	2.479468	-3.93992	0.000203	0.042627	0.585047	PD
cg23501836	-0.35233	3.39942	-3.93954	0.000203	0.042664	0.588208	PD
cg21580394	-0.30971	3.275091	-3.93951	0.000203	0.042664	0.586465	PD
cg11499854	-0.16144	2.720398	-3.93921	0.000203	0.042679	0.585826	PD

cg03998598	-0.23729	3.809987	-3.93913	0.000203	0.042679	0.584998	PD
cg09383910	-0.2707	2.894388	-3.93906	0.000203	0.042679	0.58752	PD
cg15281710	-0.1878	-1.43439	-3.93905	0.000203	0.042679	0.5861	PD
cg16894214	0.201335	-5.69107	3.939041	0.000203	0.042679	0.583523	PD
cg22320764	0.213133	3.28279	3.938	0.000204	0.042816	0.5796	PD
cg12380237	-0.1964	3.012481	-3.93794	0.000204	0.042816	0.585316	PD
cg14313932	-0.20326	2.925245	-3.93766	0.000204	0.042845	0.579527	PD
cg04799859	-0.33132	3.176355	-3.93759	0.000204	0.042845	0.584077	PD
cg22092126	-0.24579	3.888696	-3.93702	0.000205	0.042915	0.579008	PD
cg19523937	-0.17169	3.537297	-3.93653	0.000205	0.04297	0.577123	PD
cg16683508	-0.16727	2.354323	-3.93643	0.000205	0.04297	0.579441	PD
cg11703222	-0.15413	4.285445	-3.93643	0.000205	0.04297	0.578042	PD
cg13663091	0.181974	3.635091	3.936303	0.000205	0.042977	0.576726	PD
cg06448284	-0.26583	3.611355	-3.93616	0.000205	0.042987	0.57421	PD
cg22549547	-0.20403	3.666305	-3.93604	0.000205	0.042993	0.574795	PD
cg09533868	-0.34195	1.80891	-3.93577	0.000205	0.043022	0.575748	PD
cg09910601	-0.13776	3.287236	-3.93569	0.000206	0.043023	0.578027	PD
cg09052854	-0.1877	3.213775	-3.93559	0.000206	0.043027	0.577542	PD
cg06377353	-2.46024	-4.5987	-3.93536	0.000206	0.043039	0.571425	PD
cg26553477	-0.18914	2.980948	-3.93535	0.000206	0.043039	0.576837	PD
cg01469719	-0.14504	2.458291	-3.9351	0.000206	0.043064	0.572413	PD
cg07420041	-0.26529	1.8709	-3.93496	0.000206	0.043075	0.573735	PD
cg13449535	-0.19308	3.854659	-3.93464	0.000206	0.043102	0.573268	PD
cg06532877	0.282762	3.842616	3.93454	0.000206	0.043102	0.571194	PD
cg27310827	-0.09738	5.096628	-3.9345	0.000206	0.043102	0.573896	PD
cg00256675	-0.15499	1.424648	-3.93448	0.000206	0.043102	0.571482	PD
cg21925299	0.192697	0.878341	3.934008	0.000207	0.043159	0.575777	PD
cg07012062	-0.23368	4.000168	-3.93383	0.000207	0.043174	0.568561	PD
cg22838568	-0.30569	2.212044	-3.93371	0.000207	0.043181	0.570404	PD
cg21078868	0.127209	0.37113	3.933326	0.000207	0.043217	0.570825	PD
cg04311686	-0.99667	4.703644	-3.93331	0.000207	0.043217	0.572829	PD

cg27038439	-0.2138	1.504166	-3.93292	0.000207	0.043263	0.569413	PD
cg06287951	0.150514	-1.75951	3.93272	0.000208	0.043273	0.565371	PD
cg11472030	-0.55549	2.496979	-3.93269	0.000208	0.043273	0.568355	PD
cg15539239	-0.29951	3.019342	-3.93263	0.000208	0.043273	0.564465	PD
cg02509204	-0.18574	2.896176	-3.93233	0.000208	0.043307	0.566639	PD
cg17325792	-0.17404	2.9128	-3.93217	0.000208	0.043319	0.566324	PD
cg17103171	0.111562	-4.95937	3.931734	0.000208	0.043372	0.566252	PD
cg14120642	-0.18719	3.238911	-3.93158	0.000208	0.043383	0.562897	PD
cg20770459	-0.21781	2.928632	-3.93128	0.000209	0.043417	0.561019	PD
cg13273076	-0.16792	2.694731	-3.93105	0.000209	0.043439	0.56291	PD
cg14506152	-0.15338	3.394494	-3.93092	0.000209	0.043448	0.562423	PD
cg01077039	-0.1404	4.089759	-3.93059	0.000209	0.043483	0.562564	PD
cg10003974	-0.20097	1.522729	-3.9305	0.000209	0.043483	0.561948	PD
cg17146731	0.239849	-1.66303	3.930444	0.000209	0.043483	0.557465	PD
cg25713736	-0.19872	1.046282	-3.93038	0.000209	0.043483	0.563792	PD
cg24588096	0.11033	-4.77578	3.929893	0.00021	0.043531	0.560336	PD
cg16364629	0.603802	2.9737	3.929852	0.00021	0.043531	0.561914	PD
cg11438555	-0.16366	3.769202	-3.92984	0.00021	0.043531	0.558047	PD
cg03436208	0.202016	0.038032	3.929658	0.00021	0.043546	0.55564	PD
cg21149248	-0.30502	1.651213	-3.92902	0.00021	0.043628	0.554133	PD
cg25274598	-0.19083	2.812548	-3.92887	0.00021	0.04364	0.552593	PD
cg18101783	-0.1889	2.754509	-3.92877	0.00021	0.043643	0.555592	PD
cg17904870	-0.18581	3.470829	-3.9287	0.00021	0.043643	0.554519	PD
cg04828133	0.176069	-5.85443	3.92854	0.00021	0.043656	0.558992	PD
cg26927998	0.324543	-5.41693	3.928445	0.000211	0.043659	0.553864	PD
cg16582726	0.204109	4.127344	3.928107	0.000211	0.043698	0.550413	PD
cg02296242	-0.24897	2.523673	-3.92794	0.000211	0.043711	0.555234	PD
cg20690458	-0.20925	2.494347	-3.92781	0.000211	0.043721	0.551998	PD
cg14375928	-0.33864	3.392095	-3.92757	0.000211	0.043739	0.54813	PD
cg20368182	-0.24678	2.800503	-3.92754	0.000211	0.043739	0.550016	PD
cg15704909	-0.16575	3.121003	-3.92718	0.000211	0.04378	0.546959	PD

cg16011604	-0.13243	4.489998	-3.92696	0.000212	0.043794	0.548949	PD
cg13347354	-0.27049	2.135983	-3.92694	0.000212	0.043794	0.545623	PD
cg15342214	-0.1873	2.2305	-3.92675	0.000212	0.043811	0.548427	PD
cg05991223	-0.16579	3.416884	-3.92651	0.000212	0.043836	0.548142	PD
cg10251456	-0.44076	3.340219	-3.92622	0.000212	0.043868	0.548525	PD
cg12216730	-0.21656	2.445731	-3.92586	0.000212	0.043901	0.543953	PD
cg16291167	-0.16416	4.073473	-3.92585	0.000212	0.043901	0.546607	PD
cg17010160	-0.27721	2.310958	-3.92534	0.000213	0.043956	0.544965	PD
cg20353750	0.157597	-1.08213	3.925327	0.000213	0.043956	0.542317	PD
cg03467235	0.356844	2.973665	3.92517	0.000213	0.043964	0.548864	PD
cg10544367	-0.17073	3.318986	-3.92511	0.000213	0.043964	0.542131	PD
cg02338405	0.177047	2.926841	3.925053	0.000213	0.043964	0.546969	PD
cg03826759	-0.11164	5.08612	-3.9248	0.000213	0.043991	0.54166	PD
cg16912134	-0.12969	3.116481	-3.92448	0.000213	0.044027	0.547617	PD
cg09925075	-0.22471	2.489595	-3.92431	0.000213	0.044034	0.540451	PD
cg08382124	-0.12126	2.996227	-3.92429	0.000214	0.044034	0.543957	PD
cg09540629	-0.21637	5.075491	-3.92415	0.000214	0.044038	0.537232	PD
cg09135866	0.220065	-5.18051	3.924113	0.000214	0.044038	0.541458	PD
cg24365306	-0.1504	-1.44648	-3.92378	0.000214	0.044076	0.539108	PD
cg24079981	-0.35532	4.00016	-3.92365	0.000214	0.044076	0.540287	PD
cg27574929	0.555961	3.479905	3.923641	0.000214	0.044076	0.54122	PD
cg18910548	-0.1132	4.116045	-3.92342	0.000214	0.044098	0.538861	PD
cg27275200	-0.17652	2.635834	-3.92302	0.000214	0.044146	0.542481	PD
cg17910071	0.272707	3.548042	3.922696	0.000215	0.044155	0.535106	PD
cg25720128	-0.13732	4.022905	-3.92269	0.000215	0.044155	0.534083	PD
cg01963897	-0.21547	3.008909	-3.92268	0.000215	0.044155	0.538249	PD
cg25408960	0.209694	3.739758	3.922671	0.000215	0.044155	0.533293	PD
cg16218074	-0.21692	3.249193	-3.92232	0.000215	0.044196	0.53539	PD
cg25701617	-0.12737	4.333113	-3.92163	0.000215	0.044287	0.537648	PD
cg11714554	-0.21915	3.387515	-3.92152	0.000215	0.044289	0.531272	PD
cg18586515	-0.15252	3.292932	-3.92147	0.000216	0.044289	0.530654	PD

cg06965169	-0.68662	5.459552	-3.92106	0.000216	0.04434	0.531559	PD
cg27309253	0.356139	-5.17569	3.920484	0.000216	0.044414	0.526323	PD
cg07834510	-0.24069	3.044273	-3.92035	0.000216	0.044424	0.529316	PD
cg24481303	0.304327	2.322176	3.920166	0.000216	0.04444	0.529371	PD
cg05125206	0.22835	4.012962	3.919983	0.000217	0.044457	0.527294	PD
cg02507246	-0.12803	3.221711	-3.91928	0.000217	0.044551	0.525834	PD
cg25082212	-0.26039	3.218993	-3.91901	0.000217	0.044581	0.528528	PD
cg04349509	0.164604	2.105288	3.918857	0.000217	0.044592	0.519504	PD
cg10124255	-0.18612	3.237793	-3.91864	0.000218	0.044614	0.524743	PD
cg24511397	0.173514	4.19242	3.918449	0.000218	0.044617	0.522547	PD
cg10371280	-0.32661	3.616821	-3.91839	0.000218	0.044617	0.520722	PD
cg03836087	-0.26612	3.052145	-3.91836	0.000218	0.044617	0.525548	PD
cg25714865	-0.21016	2.82278	-3.91833	0.000218	0.044617	0.526895	PD
cg09999194	0.205166	-5.22058	3.917914	0.000218	0.044668	0.523076	PD
cg24501676	-0.19291	2.424206	-3.91737	0.000219	0.044734	0.51948	PD
cg04481715	-0.15287	3.498957	-3.91733	0.000219	0.044734	0.517887	PD
cg04605872	2.798942	3.232946	3.917086	0.000219	0.044759	0.518128	PD
cg07124183	-0.19033	3.523932	-3.91701	0.000219	0.044759	0.524276	PD
cg10603824	0.340125	-3.81603	3.916872	0.000219	0.04477	0.520655	PD
cg05338724	-0.12	5.197785	-3.91605	0.000219	0.044882	0.513297	PD
cg27567206	-0.16485	5.219866	-3.91584	0.00022	0.044902	0.514196	PD
cg12846691	-0.24602	3.806878	-3.91564	0.00022	0.044912	0.515942	PD
cg20039512	0.62957	4.456168	3.915524	0.00022	0.044912	0.517395	PD
cg13058512	-0.18283	2.95889	-3.91542	0.00022	0.044912	0.512956	PD
cg09637963	-0.18422	2.95832	-3.91541	0.00022	0.044912	0.516594	PD
cg07409629	-0.16926	2.684043	-3.91541	0.00022	0.044912	0.513145	PD
cg11402875	0.359482	3.868978	3.915349	0.00022	0.044912	0.517231	PD
cg07724670	0.215989	-4.0195	3.91525	0.00022	0.044916	0.516106	PD
cg22568451	-0.18864	4.150409	-3.91501	0.00022	0.044927	0.509191	PD
cg18995844	-0.17847	2.953592	-3.91497	0.00022	0.044927	0.511001	PD
cg19722347	0.222355	3.348106	3.914962	0.00022	0.044927	0.510186	PD

cg10354771	-0.20002	4.573895	-3.91432	0.000221	0.045004	0.510157	PD
cg03338584	0.548296	-4.6757	3.914302	0.000221	0.045004	0.513259	PD
cg14288249	0.53979	3.024957	3.914068	0.000221	0.045028	0.516683	PD
cg04854505	0.154936	3.400032	3.913789	0.000221	0.04506	0.515628	PD
cg06706443	-0.34993	3.932702	-3.91357	0.000221	0.045082	0.515403	PD
cg03026689	0.174298	-4.54883	3.913175	0.000222	0.045131	0.508757	PD
cg14519323	0.117372	-1.39452	3.9131	0.000222	0.045131	0.511843	PD
cg17471294	-0.16708	2.905355	-3.91251	0.000222	0.045202	0.506523	PD
cg01103513	0.232446	4.266194	3.912487	0.000222	0.045202	0.505493	PD
cg21451841	-0.26538	1.903476	-3.91194	0.000223	0.045274	0.506094	PD
cg25815083	-0.16659	4.142958	-3.91164	0.000223	0.045308	0.503869	PD
cg14536812	-0.1346	4.620699	-3.91153	0.000223	0.045315	0.498986	PD
cg16245706	-0.39537	4.315892	-3.91121	0.000223	0.04535	0.500169	PD
cg10966440	-0.26766	3.833445	-3.91115	0.000223	0.04535	0.499413	PD
cg24517467	-0.24504	1.464443	-3.91025	0.000224	0.045476	0.498576	PD
cg01818596	-0.17556	3.535944	-3.90962	0.000224	0.04555	0.497119	PD
cg06445916	-0.17016	4.544285	-3.90962	0.000224	0.04555	0.499244	PD
cg21066028	-0.14835	3.938693	-3.9093	0.000224	0.045587	0.492429	PD
cg18625147	0.303986	-3.63363	3.908581	0.000225	0.045686	0.494171	PD
cg03340052	-0.22381	3.939872	-3.90756	0.000226	0.045831	0.49339	PD
cg17343385	-0.71149	3.493041	-3.90709	0.000226	0.045892	0.495111	PD
cg10769722	-0.19267	2.939783	-3.90679	0.000226	0.04592	0.486871	PD
cg01387720	-0.2477	2.951994	-3.90677	0.000226	0.04592	0.489178	PD
cg00727334	-0.26158	3.622009	-3.9066	0.000227	0.045934	0.493889	PD
cg05148373	0.165557	-3.76278	3.906152	0.000227	0.045992	0.485836	PD
cg13106758	-0.23944	2.136657	-3.90596	0.000227	0.046011	0.490013	PD
cg01101936	0.159711	4.346371	3.905586	0.000227	0.046057	0.48385	PD
cg03226599	-0.2522	3.12056	-3.9054	0.000227	0.046074	0.488081	PD
cg18004311	0.245225	1.590976	3.905312	0.000228	0.046077	0.483192	PD
cg01518970	-0.16139	3.379357	-3.90505	0.000228	0.046107	0.485571	PD
cg23023673	-0.16528	4.053954	-3.90411	0.000228	0.046224	0.479856	PD

cg16084969	-0.30379	3.415708	-3.90406	0.000228	0.046224	0.482482	PD
cg24073022	-0.14108	3.313827	-3.90405	0.000228	0.046224	0.480562	PD
cg09351263	2.586561	-4.02744	3.904003	0.000229	0.046224	0.485324	PD
cg21577110	-0.29574	2.846289	-3.90345	0.000229	0.046295	0.480342	PD
cg13722821	-0.26469	-7.03147	-3.9034	0.000229	0.046295	0.475449	PD
cg07310677	0.243075	-0.87555	3.903086	0.000229	0.046333	0.47346	PD
cg09572125	-0.15046	1.141674	-3.90296	0.000229	0.046336	0.473272	PD
cg05502732	-0.1937	2.812643	-3.90292	0.000229	0.046336	0.479811	PD
cg17025149	-0.28534	1.399882	-3.90247	0.00023	0.046394	0.473026	PD
cg07340660	-0.15235	2.982519	-3.90219	0.00023	0.046426	0.474996	PD
cg09139874	-0.23501	3.774626	-3.90206	0.00023	0.046436	0.476077	PD
cg03367273	-0.30157	3.246309	-3.90192	0.00023	0.046446	0.472833	PD
cg09420665	0.136988	-5.50926	3.901199	0.000231	0.046548	0.468841	PD
cg00138804	-0.15948	1.050334	-3.90038	0.000231	0.046664	0.465347	PD
cg22224989	-0.23529	-5.29108	-3.89939	0.000232	0.046797	0.465771	PD
cg06271067	-0.23734	2.564892	-3.89938	0.000232	0.046797	0.46874	PD
cg00306390	0.135697	-3.72064	3.899092	0.000232	0.046832	0.467559	PD
cg17122758	-0.18109	1.477727	-3.8986	0.000233	0.046897	0.463018	PD
cg14878128	-0.40093	2.267578	-3.89812	0.000233	0.046962	0.464325	PD
cg25407044	0.246359	3.223363	3.898026	0.000233	0.046965	0.460344	PD
cg15388832	-0.33379	3.913176	-3.89788	0.000233	0.046976	0.461498	PD
cg23755398	-0.32192	2.037272	-3.89769	0.000233	0.046996	0.464056	PD
cg26533193	0.152399	1.365671	3.897609	0.000233	0.046997	0.465079	PD
cg19895882	0.166012	-6.37872	3.897445	0.000234	0.047011	0.460978	PD
cg21264521	-0.24153	2.229611	-3.89733	0.000234	0.047019	0.461132	PD
cg07346595	-0.27388	3.847338	-3.89712	0.000234	0.04704	0.461022	PD
cg23514537	-0.28814	3.390726	-3.89661	0.000234	0.047109	0.460067	PD
cg24258125	0.380219	-2.82638	3.89654	0.000234	0.047109	0.461762	PD
cg13444005	-0.09887	6.061751	-3.89643	0.000234	0.047115	0.452933	PD
cg02232067	-0.21066	4.085944	-3.896	0.000235	0.047171	0.452948	PD
cg23741612	-0.16716	3.280225	-3.89582	0.000235	0.047179	0.451064	PD

cg15934372	-0.20701	2.206107	-3.89581	0.000235	0.047179	0.454186	PD
cg04910505	-0.61442	3.712579	-3.89568	0.000235	0.04718	0.458629	PD
cg13430687	-0.21923	3.795303	-3.89566	0.000235	0.04718	0.455824	PD
cg01073602	-0.18356	2.816703	-3.89539	0.000235	0.047211	0.454326	PD
cg27339395	-0.16596	4.734349	-3.89486	0.000236	0.047284	0.450019	PD
cg01317037	-0.22114	1.92553	-3.89402	0.000236	0.047404	0.4502	PD
cg07626409	-0.15925	3.227178	-3.89343	0.000237	0.047479	0.452296	PD
cg12158933	-0.16405	3.709793	-3.89341	0.000237	0.047479	0.451004	PD
cg19333201	-0.15057	3.820488	-3.89332	0.000237	0.047482	0.452071	PD
cg18456782	-0.15935	3.932605	-3.89319	0.000237	0.047492	0.448072	PD
cg11028188	-0.19785	3.86984	-3.89308	0.000237	0.047497	0.448836	PD
cg01388803	0.168178	-6.6185	3.89289	0.000237	0.047516	0.451735	PD
cg15438506	-0.16181	3.867185	-3.89222	0.000238	0.047611	0.446653	PD
cg19234412	-0.14609	3.039348	-3.89188	0.000238	0.047654	0.446248	PD
cg22482014	-0.14502	4.328543	-3.89168	0.000238	0.047669	0.439916	PD
cg07134292	0.195707	-6.08975	3.891643	0.000238	0.047669	0.450301	PD
cg11303839	-0.15842	2.634687	-3.89136	0.000238	0.0477	0.441106	PD
cg14286546	-0.17177	4.220369	-3.89131	0.000238	0.0477	0.441601	PD
cg27507412	-0.12348	4.004259	-3.89098	0.000239	0.047741	0.446448	PD
cg27089789	-0.20809	3.052016	-3.89079	0.000239	0.047759	0.441064	PD
cg26270263	-0.20635	2.727454	-3.89052	0.000239	0.047791	0.438242	PD
cg03506768	-0.20077	3.97315	-3.89041	0.000239	0.047798	0.440107	PD
cg02785332	0.195109	0.247896	3.890324	0.000239	0.0478	0.435895	PD
cg01155411	-0.36258	3.28493	-3.8902	0.000239	0.047807	0.436139	PD
cg18032575	-0.20061	3.783852	-3.89014	0.000239	0.047807	0.441696	PD
cg20284323	0.281957	4.262241	3.890036	0.000239	0.047811	0.439871	PD
cg20189474	-0.23831	3.507608	-3.88997	0.000239	0.047811	0.439875	PD
cg14030282	0.453096	-6.5935	3.889419	0.00024	0.047888	0.438935	PD
cg03498228	-0.30626	5.373096	-3.88925	0.00024	0.047903	0.438868	PD
cg21612334	0.325257	-3.66925	3.88895	0.00024	0.047932	0.435366	PD
cg07855471	-0.16042	3.258478	-3.88886	0.00024	0.047932	0.431695	PD

cg23634560	-0.15114	2.579546	-3.88886	0.00024	0.047932	0.434334	PD
cg10387537	-0.30415	2.915874	-3.88849	0.000241	0.047981	0.429348	PD
cg09917907	-0.17812	3.597866	-3.8883	0.000241	0.047999	0.431779	PD
cg21767391	-0.15609	1.224389	-3.88804	0.000241	0.048029	0.434227	PD
cg16543923	-0.24123	-5.37653	-3.88771	0.000241	0.048071	0.429159	PD
cg23644680	-0.2212	2.513224	-3.88732	0.000242	0.048122	0.425852	PD
cg02244204	-0.1548	4.930132	-3.88717	0.000242	0.048135	0.433973	PD
cg08732245	-0.3595	2.383256	-3.88702	0.000242	0.048147	0.429424	PD
cg19677978	-0.26009	3.319139	-3.8869	0.000242	0.048155	0.431214	PD
cg09014674	-0.21783	2.146758	-3.88632	0.000242	0.048238	0.425794	PD
cg03931865	-0.27463	2.067277	-3.8855	0.000243	0.048358	0.427156	PD
cg13791713	-0.11453	-0.72422	-3.88461	0.000244	0.048485	0.421535	PD
cg04114767	-0.1989	3.084625	-3.88457	0.000244	0.048485	0.423253	PD
cg04403473	-0.1268	3.320412	-3.88418	0.000244	0.048538	0.419449	PD
cg21127079	-0.28231	3.852477	-3.88394	0.000244	0.048564	0.421088	PD
cg20901971	0.125622	-0.67562	3.883648	0.000245	0.0486	0.415697	PD
cg01200344	0.192557	-0.1785	3.883313	0.000245	0.048643	0.417945	PD
ch.5.1837083R	0.255932	-4.71351	3.883205	0.000245	0.048649	0.417882	PD
cg04297660	0.404146	3.323387	3.883061	0.000245	0.048661	0.424677	PD
cg03733026	0.448031	4.265387	3.882818	0.000245	0.048689	0.419716	PD
cg11089117	0.210765	4.043294	3.882453	0.000246	0.048735	0.417834	PD
cg02472077	-0.21898	3.275854	-3.88236	0.000246	0.048735	0.419083	PD
cg10190679	-0.31473	3.495582	-3.88232	0.000246	0.048735	0.418342	PD
cg23626862	-0.25696	2.331946	-3.88224	0.000246	0.048736	0.417843	PD
cg19908211	-0.24602	3.153561	-3.88201	0.000246	0.048762	0.411781	PD
cg26850224	-0.29789	3.152728	-3.88175	0.000246	0.048794	0.414119	PD
cg08698639	-0.26693	3.909141	-3.8812	0.000247	0.048872	0.410641	PD
cg24330514	-0.27502	2.301064	-3.88056	0.000247	0.048965	0.415347	PD
cg01026744	-0.12096	0.539923	-3.88032	0.000247	0.048989	0.412447	PD
cg18698096	0.207784	-1.99627	3.880273	0.000247	0.048989	0.406881	PD
cg18761422	-0.36724	-3.11815	-3.88006	0.000248	0.049013	0.410876	PD

cg09882535	-0.27132	1.408993	-3.87998	0.000248	0.049013	0.409764	PD
cg14926485	0.305289	3.374681	3.879823	0.000248	0.049028	0.409117	PD
cg13588517	-0.34693	2.622389	-3.87967	0.000248	0.049041	0.407284	PD
cg02448481	-0.26308	5.213038	-3.87945	0.000248	0.049065	0.403925	PD
cg11927004	0.680305	5.647063	3.879303	0.000248	0.049075	0.408849	PD
cg21420099	-0.22261	3.348208	-3.8792	0.000248	0.049075	0.409482	PD
cg18400151	-0.1761	3.236766	-3.87918	0.000248	0.049075	0.403111	PD
cg18840931	-0.26596	1.117141	-3.8789	0.000248	0.049109	0.400446	PD
cg12738627	0.213565	2.941156	3.878542	0.000249	0.049148	0.40277	PD
cg14298807	-1.90507	-5.02081	-3.87853	0.000249	0.049148	0.403537	PD
cg06439124	0.241631	-4.27916	3.878208	0.000249	0.049186	0.404892	PD
cg16511076	-0.15196	3.308159	-3.87815	0.000249	0.049186	0.404467	PD
cg14290506	-0.23467	2.841995	-3.87803	0.000249	0.049194	0.40419	PD
cg25280936	-0.20812	3.371843	-3.87795	0.000249	0.049197	0.399955	PD
cg17484771	-0.305	5.31565	-3.87776	0.000249	0.049215	0.402243	PD
cg05324501	-0.22759	3.515253	-3.87648	0.00025	0.049411	0.393856	PD
cg09168797	-0.28263	3.631292	-3.87642	0.000251	0.049411	0.396108	PD
cg03178232	-0.14879	2.791679	-3.87636	0.000251	0.049411	0.39394	PD
cg26017679	-0.12889	4.714662	-3.87623	0.000251	0.049421	0.395447	PD
cg26143874	-0.307	3.679692	-3.87608	0.000251	0.049422	0.394797	PD
cg01749295	-0.23366	2.726562	-3.87605	0.000251	0.049422	0.398908	PD
cg13948402	-0.23804	2.946462	-3.87601	0.000251	0.049422	0.396319	PD
cg05396121	-0.1809	2.908824	-3.87569	0.000251	0.049461	0.395771	PD
cg26145268	0.411957	2.708562	3.875634	0.000251	0.049461	0.398781	PD
cg00062367	0.254045	4.194474	3.87546	0.000251	0.049479	0.393076	PD
cg10338348	-0.27549	4.081769	-3.87522	0.000252	0.0495	0.398295	PD
cg11732681	-0.21425	3.335447	-3.87519	0.000252	0.0495	0.402314	PD
cg01132515	-0.3163	3.22715	-3.87508	0.000252	0.049507	0.392918	PD
cg08834518	0.1921	4.025926	3.874676	0.000252	0.04956	0.391594	PD
cg26141856	0.142119	0.729535	3.874619	0.000252	0.04956	0.395008	PD
cg01792612	-0.21675	3.528561	-3.87447	0.000252	0.049572	0.391938	PD

cg03026011	-0.19916	2.886462	-3.87439	0.000252	0.049574	0.396342	PD
cg15271066	-0.25324	3.61318	-3.87353	0.000253	0.049704	0.387916	PD
cg22991191	0.160864	4.002108	3.87287	0.000253	0.049782	0.382243	PD
cg06566772	0.234517	-4.2067	3.872821	0.000254	0.049782	0.388527	PD
cg14286187	0.307136	-3.60384	3.872798	0.000254	0.049782	0.382728	PD
cg23476830	0.517289	-4.47209	3.872786	0.000254	0.049782	0.390636	PD
cg00605127	-0.19925	3.060108	-3.87267	0.000254	0.049789	0.383567	PD
cg00650614	-0.31206	2.258485	-3.87244	0.000254	0.049816	0.385545	PD
cg23766756	0.312838	-5.40975	3.872157	0.000254	0.049851	0.384718	PD
cg27362525	-0.50543	-4.5122	-3.87201	0.000254	0.049864	0.386245	PD
cg16590643	0.341312	2.263467	3.871379	0.000255	0.049957	0.390545	PD

Supplementary Table ST4: DNA methylation changes in various genomic regions in Parkinson's disease brain.

**Contrast**	**Category-hg19**	**Type**	**OR**	**Lo**	**Hi**	**P**	**Q**
PD	genes_promoters	All	0.62	0.57	0.67	5.21E-33	5.73E-32
PD	genes_promoters	Hyper-	1.05	0.92	1.20	0.43	0.47
PD	genes_promoters	Hypo-	0.46	0.41	0.52	1.29E-51	4.28E-50
PD	genes_1to5kb	All	0.80	0.73	0.87	1.99E-07	4.70E-07
PD	genes_1to5kb	Hyper-	0.90	0.77	1.05	0.2	0.26
PD	genes_1to5kb	Hypo-	0.76	0.68	0.84	9.29E-08	2.35E-07
PD	cpg_shores	All	0.65	0.59	0.71	2.75E-23	1.30E-22
PD	cpg_shores	Hyper-	0.83	0.70	0.96	0.014638352	0.02
PD	cpg_shores	Hypo-	0.58	0.51	0.64	1.01E-24	6.71E-24
PD	genes_intergenic	All	1.26	1.18	1.35	2.93E-11	8.80E-11
PD	genes_intergenic	Hyper-	1.07	0.93	1.22	0.33	0.38
PD	genes_intergenic	Hypo-	1.34	1.24	1.45	4.98E-13	1.82E-12
PD	cpg_inter	All	1.42	1.33	1.51	6.47E-29	5.34E-28
PD	cpg_inter	Hyper-	0.99	0.88	1.11	0.86	0.86

PD	cpg_inter	Hypo-	1.66	1.54	1.80	3.92E-41	6.47E-40
PD	genes_introns	All	1.06	1.00	1.12	0.06	0.1
PD	genes_introns	Hyper-	1.05	0.94	1.18	0.35	0.398
PD	genes_introns	Hypo-	1.06	0.98	1.14	0.12	0.163
PD	genes_exons	All	0.84	0.77	0.91	2.49E-05	0.000054
PD	genes_exons	Hyper-	0.88	0.75	1.03	0.12	0.16
PD	genes_exons	Hypo-	0.82	0.74	0.90	6.73E-05	0.00013
PD	genes_3UTRs	All	0.97	0.81	1.16	0.79	0.81
PD	genes_3UTRs	Hyper-	0.62	0.40	0.93	0.015	0.02
PD	genes_3UTRs	Hypo-	1.12	0.91	1.36	0.255	0.31
PD	cpg_islands	All	0.76	0.69	0.82	2.30E-11	7.60E-11
PD	cpg_islands	Hyper-	1.23	1.07	1.40	0.0036	0.006
PD	cpg_islands	Hypo-	0.59	0.52	0.65	3.33E-24	1.83E-23
PD	genes_5UTRs	All	0.63	0.54	0.74	1.45E-09	4.00E-09
PD	genes_5UTRs	Hyper-	1.21	0.96	1.51	0.090	0.12
PD	genes_5UTRs	Hypo-	0.41	0.32	0.52	1.29E-18	5.35E-18
PD	cpg_shelves	All	1.16	1.04	1.29	0.009	0.015
PD	cpg_shelves	Hyper-	0.94	0.74	1.17	0.621	0.66
PD	cpg_shelves	Hypo-	1.25	1.10	1.42	0.001	0.0012

Supplementary Table ST5: Enrichment of regulatory regions on genomic regions and genes.

Category	Type	OR	Lo	Hi	P	P < 0.05	Q
25_Quies	All	1.08	1.02	1.14	0.006095505	**	0.024450979
25_Quies	Hyper-	0.89	0.82	0.96	0.00205755	**	0.010119098
25_Quies	Hypo-	1.32	1.22	1.43	1.39768174499133e-12	***	4.19304523497399e-11
21_Het	All	0.92	0.62	1.32	0.724321148		0.798883619
21_Het	Hyper-	1.07	0.63	1.69	0.713728431		0.792361261
21_Het	Hypo-	0.77	0.40	1.34	0.444208043		0.576503365
19_DNase	All	0.85	0.71	1.00	0.057471771	.	0.13576009

19_DNase	Hyper-	0.78	0.60	1.00	0.050087144	.	0.125217859
19_DNase	Hypo-	0.92	0.71	1.16	0.521859659		0.644271184
17_EnhW2	All	1.00	0.82	1.20	1		1
17_EnhW2	Hyper-	0.86	0.63	1.14	0.314513218		0.437186232
17_EnhW2	Hypo-	1.14	0.87	1.48	0.295274391		0.415879424
4_PromD2	All	0.74	0.45	1.14	0.207000625		0.330320146
4_PromD2	Hyper-	0.57	0.25	1.12	0.137202706		0.24754622
4_PromD2	Hypo-	0.93	0.48	1.62	1		1
1_TssA	All	1.16	1.04	1.28	0.005858953	**	0.024077889
1_TssA	Hyper-	1.84	1.63	2.06	2.88428812683197e-21	***	8.6528643804959e-19
1_TssA	Hypo-	0.48	0.38	0.60	1.62282218600138e-13	***	6.08558319750516e-12
22_PromP	All	1.08	0.87	1.31	0.462483384		0.587902607
22_PromP	Hyper-	1.27	0.97	1.64	0.06913023	.	0.148136207
22_PromP	Hypo-	0.87	0.62	1.19	0.449384292		0.576503365
2_PromU	All	0.92	0.83	1.01	0.097854565	.	0.186983246
2_PromU	Hyper-	1.14	1.00	1.29	0.042654176	*	0.113450096
2_PromU	Hypo-	0.70	0.59	0.82	2.52126E-06	***	2.8014E-05
13_EnhA1	All	0.67	0.53	0.84	0.000182252	***	0.001298594
13_EnhA1	Hyper-	0.76	0.56	1.01	0.063485781	.	0.145297556
13_EnhA1	Hypo-	0.58	0.40	0.81	0.000854484	***	0.004747131
14_EnhA2	All	0.74	0.58	0.94	0.013730958	*	0.047348132
14_EnhA2	Hyper-	0.55	0.36	0.80	0.001050181	**	0.005625969
14_EnhA2	Hypo-	0.96	0.69	1.29	0.879969329		0.926283504
15_EnhAF	All	1.02	0.80	1.28	0.860615885		0.915548814
15_EnhAF	Hyper-	0.67	0.43	0.98	0.042158806	*	0.113450096
15_EnhAF	Hypo-	1.40	1.04	1.86	0.022446005	*	0.070882121
8_TxWk	All	1.03	0.92	1.15	0.565761696		0.676327732
8_TxWk	Hyper-	0.82	0.68	0.97	0.018906367	*	0.061651197
8_TxWk	Hypo-	1.27	1.10	1.47	0.001649391	**	0.008386732
12_TxEnhW	All	0.85	0.65	1.09	0.210806947		0.334614202
12_TxEnhW	Hyper-	0.80	0.54	1.15	0.248078381		0.372117572
12_TxEnhW	Hypo-	0.90	0.61	1.28	0.606843393		0.711420927
5_Tx5'	All	1.07	0.90	1.27	0.405419248		0.535796363
5_Tx5'	Hyper-	0.94	0.73	1.20	0.715766339		0.792361261
5_Tx5'	Hypo-	1.21	0.96	1.52	0.10062319		0.19105669
3_PromD1	All	0.90	0.81	0.99	0.0305254	*	0.091576201
3_PromD1	Hyper-	1.17	1.03	1.32	0.012330056	*	0.043517844
3_PromD1	Hypo-	0.62	0.52	0.73	1.22477730407236e-9	***	3.06194326018091e-8
7_Tx3'	All	1.06	0.94	1.19	0.316152881		0.437186232
7_Tx3'	Hyper-	0.71	0.58	0.86	0.000341495	***	0.00222714
7_Tx3'	Hypo-	1.45	1.25	1.68	1.72789E-06	***	2.02344E-05

16_EnhW1	All	1.05	0.86	1.26	0.630394307		0.730186457
16_EnhW1	Hyper-	1.04	0.79	1.34	0.738921065		0.81200117
16_EnhW1	Hypo-	1.05	0.79	1.37	0.677735424		0.765505415
24_ReprPC	All	1.15	0.99	1.33	0.065265644	.	0.146117114
24_ReprPC	Hyper-	1.55	1.29	1.85	4.92537E-06	***	5.09521E-05
24_ReprPC	Hypo-	0.73	0.55	0.95	0.015333738	*	0.052274105
23_PromBiv	All	1.05	0.89	1.24	0.525153746		0.645680835
23_PromBiv	Hyper-	1.55	1.27	1.87	1.68212E-05	***	0.000140947
23_PromBiv	Hypo-	0.53	0.37	0.73	3.04582E-05	***	0.000240459
11_TxEnh3'	All	0.86	0.63	1.13	0.316231374		0.437186232
11_TxEnh3'	Hyper-	0.37	0.18	0.66	0.000135505	***	0.000991501
11_TxEnh3'	Hypo-	1.39	0.98	1.92	0.053347714	.	0.131182903
6_Tx	All	1.22	1.00	1.47	0.04326947	*	0.113552908
6_Tx	Hyper-	0.83	0.59	1.13	0.268925215		0.391638663
6_Tx	Hypo-	1.65	1.29	2.09	0.00011662	***	0.000874649
9_TxReg	All	0.77	0.63	0.93	0.005201927	**	0.021979975
9_TxReg	Hyper-	0.78	0.59	1.01	0.064827528	.	0.146117114
9_TxReg	Hypo-	0.75	0.56	0.99	0.047361848	*	0.120411477
10_TxEnh5'	All	0.84	0.68	1.04	0.115711985		0.214281454
10_TxEnh5'	Hyper-	0.68	0.48	0.94	0.01704086	*	0.056802865
10_TxEnh5'	Hypo-	1.02	0.76	1.34	0.887301335		0.926647951
20_ZNF/Rpts	All	1.02	0.51	1.82	0.878473464		0.926283504
20_ZNF/Rpts	Hyper-	1.25	0.50	2.58	0.520888649		0.644271184
20_ZNF/Rpts	Hypo-	0.77	0.21	1.97	0.824809098		0.886891504
18_EnhAc	All	0.77	0.44	1.25	0.322407512		0.443680063
18_EnhAc	Hyper-	0.64	0.26	1.33	0.286668625		0.405663149
18_EnhAc	Hypo-	0.90	0.41	1.71	0.874460654		0.926283504
25_Quies	All	1.08	1.02	1.14	0.005117879	**	0.021979975
25_Quies	Hyper-	0.89	0.82	0.96	0.00248945	**	0.011854524
25_Quies	Hypo-	1.32	1.22	1.43	1.19050597868269e-12	***	3.96835326227565e-11
19_DNase	All	0.84	0.69	1.01	0.06737072	.	0.147328927
19_DNase	Hyper-	0.86	0.66	1.11	0.27702266		0.397640182
19_DNase	Hypo-	0.82	0.62	1.08	0.167352796		0.282055273
17_EnhW2	All	1.06	0.91	1.22	0.440654502		0.574766742
17_EnhW2	Hyper-	0.76	0.60	0.95	0.016709246	*	0.056323301
17_EnhW2	Hypo-	1.39	1.15	1.66	0.000573167	***	0.003439004
4_PromD2	All	0.86	0.58	1.24	0.486928217		0.613775064
4_PromD2	Hyper-	0.51	0.23	0.98	0.039999642	*	0.112148529
4_PromD2	Hypo-	1.24	0.76	1.92	0.315876998		0.437186232
1_TssA	All	1.12	1.01	1.23	0.027040353	*	0.082776589
1_TssA	Hyper-	1.77	1.57	1.98	4.29840869550998e-20	***	6.44761304326496e-18

1_TssA	Hypo-	0.48	0.38	0.59	4.19819028071243e-15	***	2.51891416842746e-13
22_PromP	All	0.91	0.71	1.15	0.457071998		0.583496167
22_PromP	Hyper-	1.26	0.94	1.67	0.096112134	.	0.186023484
22_PromP	Hypo-	0.53	0.32	0.82	0.002854501	**	0.013380474
21_Het	All	0.90	0.59	1.30	0.651949458		0.743668583
21_Het	Hyper-	1.11	0.66	1.76	0.616629754		0.719801269
21_Het	Hypo-	0.67	0.32	1.23	0.241093394		0.365293021
2_PromU	All	0.89	0.80	0.99	0.024295155	*	0.075139656
2_PromU	Hyper-	1.05	0.92	1.20	0.435460376		0.570472108
2_PromU	Hypo-	0.72	0.61	0.85	2.66585E-05	***	0.00021615
18_EnhAc	All	0.92	0.47	1.61	0.889579896		0.926647951
18_EnhAc	Hyper-	1.03	0.41	2.13	0.846443644		0.903676488
18_EnhAc	Hypo-	0.80	0.26	1.86	0.839923627		0.899918172
13_EnhA1	All	0.80	0.63	1.00	0.050807643	.	0.125969364
13_EnhA1	Hyper-	0.93	0.68	1.24	0.669841035		0.761182995
13_EnhA1	Hypo-	0.66	0.45	0.94	0.017785924	*	0.058634914
15_EnhAF	All	0.82	0.64	1.03	0.093912314	.	0.184141792
15_EnhAF	Hyper-	0.52	0.33	0.78	0.000709718	***	0.00401727
15_EnhAF	Hypo-	1.14	0.84	1.52	0.349593206		0.478894803
8_TxWk	All	1.03	0.92	1.15	0.58748053		0.696617229
8_TxWk	Hyper-	0.88	0.74	1.04	0.132812127		0.241476595
8_TxWk	Hypo-	1.20	1.02	1.39	0.021195855	*	0.067646344
12_TxEnhW	All	0.78	0.61	0.98	0.032724815	*	0.095314996
12_TxEnhW	Hyper-	0.58	0.39	0.84	0.002403013	**	0.011627484
12_TxEnhW	Hypo-	0.99	0.72	1.33	1		1
5_Tx5'	All	1.17	0.98	1.39	0.072431365	.	0.15302401
5_Tx5'	Hyper-	1.16	0.90	1.47	0.237817784		0.364006813
5_Tx5'	Hypo-	1.18	0.91	1.51	0.194962466		0.31445559
3_PromD1	All	0.91	0.82	1.02	0.106025791		0.198798358
3_PromD1	Hyper-	1.16	1.01	1.33	0.036346978	*	0.103848508
3_PromD1	Hypo-	0.66	0.55	0.79	1.75365E-06	***	2.02344E-05
7_Tx3'	All	1.05	0.93	1.17	0.395087303		0.52678307
7_Tx3'	Hyper-	0.72	0.59	0.86	0.00025061	***	0.001708703
7_Tx3'	Hypo-	1.43	1.23	1.64	3.17781E-06	***	3.4048E-05
16_EnhW1	All	0.85	0.68	1.04	0.111912533		0.208532671
16_EnhW1	Hyper-	0.69	0.49	0.94	0.019261727	*	0.062134603
16_EnhW1	Hypo-	1.02	0.76	1.33	0.889582033		0.926647951
24_ReprPC	All	1.13	0.99	1.28	0.075082709	.	0.155137812
24_ReprPC	Hyper-	1.55	1.32	1.81	2.1200384477777e-7	***	3.34743E-06
24_ReprPC	Hypo-	0.69	0.54	0.87	0.001270417	**	0.006686407
23_PromBiv	All	1.09	0.93	1.26	0.261958895		0.386234222

23_PromBiv	Hyper-	1.59	1.34	1.89	4.19147025442047e-7	***	6.28721E-06
23_PromBiv	Hypo-	0.56	0.41	0.74	1.65482E-05	***	0.000140947
6_Tx	All	1.19	0.97	1.45	0.080505897	.	0.16209241
6_Tx	Hyper-	0.85	0.60	1.17	0.363813074		0.49163929
6_Tx	Hypo-	1.57	1.20	2.01	0.000896812	***	0.004891704
14_EnhA2	All	0.83	0.64	1.06	0.149857214		0.261378861
14_EnhA2	Hyper-	0.75	0.50	1.07	0.12903571		0.236040934
14_EnhA2	Hypo-	0.92	0.63	1.28	0.678748135		0.765505415
10_TxEnh5'	All	0.80	0.62	1.02	0.077730997	.	0.157562832
10_TxEnh5'	Hyper-	0.68	0.46	0.97	0.032585508	*	0.095314996
10_TxEnh5'	Hypo-	0.94	0.66	1.29	0.751323086		0.819625185
9_TxReg	All	0.75	0.59	0.93	0.008629129	**	0.032273232
9_TxReg	Hyper-	0.77	0.56	1.04	0.092681082	.	0.183102836
9_TxReg	Hypo-	0.72	0.51	0.99	0.049629688	*	0.125116861
20_ZNF/Rpts	All	1.15	0.60	2.02	0.534514492		0.648866411
20_ZNF/Rpts	Hyper-	1.48	0.64	2.93	0.272315481		0.394660117
20_ZNF/Rpts	Hypo-	0.80	0.22	2.05	0.824088286		0.886891504
11_TxEnh3'	All	0.83	0.64	1.06	0.149523628		0.261378861
11_TxEnh3'	Hyper-	0.42	0.24	0.68	6.00091E-05	***	0.000461609
11_TxEnh3'	Hypo-	1.28	0.94	1.70	0.09693885	.	0.186420866
25_Quies	All	1.05	1.00	1.11	0.060140964	.	0.13878684
25_Quies	Hyper-	0.88	0.81	0.95	0.001621279	**	0.008385923
25_Quies	Hypo-	1.27	1.17	1.37	3.42693005020945e-9	***	7.90830011586796e-8
19_DNase	All	0.95	0.80	1.13	0.640369239		0.738887583
19_DNase	Hyper-	0.92	0.72	1.17	0.556723294		0.670750956
19_DNase	Hypo-	0.99	0.76	1.26	1		1
8_TxWk	All	1.03	0.92	1.15	0.565860869		0.676327732
8_TxWk	Hyper-	0.90	0.76	1.06	0.232952775		0.358388885
8_TxWk	Hypo-	1.17	1.00	1.37	0.038647282	*	0.109379101
7_Tx3'	All	1.09	0.97	1.22	0.142453899		0.251389234
7_Tx3'	Hyper-	0.77	0.64	0.92	0.0038984	**	0.017719999
7_Tx3'	Hypo-	1.44	1.24	1.66	1.37532E-06	***	1.71915E-05
22_PromP	All	1.01	0.80	1.26	0.910707814		0.942111532
22_PromP	Hyper-	1.29	0.96	1.68	0.073560698	.	0.153251453
22_PromP	Hypo-	0.71	0.47	1.03	0.075500402	.	0.155137812
1_TssA	All	1.10	0.99	1.22	0.067919178	.	0.147328927
1_TssA	Hyper-	1.74	1.54	1.96	8.81579465260012e-18	***	8.81579465260012e-16
1_TssA	Hypo-	0.47	0.37	0.58	1.00499260510313e-14	***	5.02496302551565e-13
21_Het	All	0.89	0.58	1.30	0.64713049		0.74098911
21_Het	Hyper-	1.01	0.58	1.65	0.899076185		0.933297078

21_Het	Hypo-	0.75	0.38	1.35	0.428783687		0.564189062
16_EnhW1	All	1.13	0.94	1.35	0.181052367		0.298437967
16_EnhW1	Hyper-	0.90	0.67	1.18	0.5084003		0.632863444
16_EnhW1	Hypo-	1.38	1.08	1.74	0.00882135	**	0.032273232
2_PromU	All	0.90	0.81	0.99	0.035627668	*	0.102772118
2_PromU	Hyper-	1.08	0.94	1.22	0.264176815		0.386600216
2_PromU	Hypo-	0.71	0.60	0.84	1.46386E-05	***	0.000133079
13_EnhA1	All	0.72	0.55	0.94	0.011452421	*	0.040901505
13_EnhA1	Hyper-	0.69	0.46	0.99	0.042644093	*	0.113450096
13_EnhA1	Hypo-	0.77	0.52	1.10	0.168464553		0.282342827
18_EnhAc	All	0.80	0.43	1.38	0.530929017		0.647474412
18_EnhAc	Hyper-	0.71	0.26	1.55	0.600141612		0.708828676
18_EnhAc	Hypo-	0.90	0.36	1.86	1		1
15_EnhAF	All	0.94	0.76	1.15	0.585077961		0.696521383
15_EnhAF	Hyper-	0.73	0.52	1.00	0.05391493	.	0.131499829
15_EnhAF	Hypo-	1.16	0.88	1.51	0.252337111		0.376622554
10_TxEnh5'	All	0.73	0.58	0.92	0.006275751	**	0.024450979
10_TxEnh5'	Hyper-	0.64	0.45	0.89	0.007192693	**	0.027314026
10_TxEnh5'	Hypo-	0.84	0.60	1.13	0.283255699		0.402733222
12_TxEnhW	All	0.90	0.72	1.11	0.382721199		0.512573034
12_TxEnhW	Hyper-	0.73	0.51	1.01	0.054687259	.	0.132307884
12_TxEnhW	Hypo-	1.09	0.81	1.44	0.505806944		0.632863444
5_Tx5'	All	1.14	0.96	1.35	0.126012831		0.231925456
5_Tx5'	Hyper-	1.06	0.82	1.36	0.607079191		0.711420927
5_Tx5'	Hypo-	1.23	0.95	1.55	0.094833028	.	0.184739666
3_PromD1	All	0.90	0.81	1.00	0.0552577	.	0.132555529
3_PromD1	Hyper-	1.16	1.01	1.33	0.028564306	*	0.086558503
3_PromD1	Hypo-	0.63	0.52	0.76	8.86087342680021e-8	***	1.56368E-06
24_ReprPC	All	1.13	0.99	1.29	0.058359322	.	0.136779662
24_ReprPC	Hyper-	1.51	1.29	1.77	8.35206989317551e-7	***	1.0894E-05
24_ReprPC	Hypo-	0.73	0.58	0.92	0.005490034	**	0.022875141
23_PromBiv	All	1.06	0.91	1.22	0.445931061		0.576503365
23_PromBiv	Hyper-	1.63	1.37	1.92	8.33490605612666e-8	***	1.56279E-06
23_PromBiv	Hypo-	0.47	0.33	0.64	6.4235367844686e-8	***	1.28471E-06
11_TxEnh3'	All	0.78	0.61	0.99	0.045366563	*	0.116324521
11_TxEnh3'	Hyper-	0.41	0.25	0.65	1.25143E-05	***	0.000117321
11_TxEnh3'	Hypo-	1.19	0.88	1.58	0.212731289		0.335891508
6_Tx	All	1.20	0.99	1.45	0.055673322	.	0.132555529
6_Tx	Hyper-	0.84	0.60	1.14	0.283249329		0.402733222
6_Tx	Hypo-	1.60	1.25	2.02	0.000186132	***	0.001298594
4_PromD2	All	0.75	0.49	1.10	0.15220011		0.263930827

4_PromD2	Hyper-	0.27	0.09	0.62	0.00040226	***	0.002567618
4_PromD2	Hypo-	1.28	0.80	1.95	0.225500294		0.348711794
17_EnhW2	All	0.94	0.80	1.09	0.397993736		0.528310269
17_EnhW2	Hyper-	0.73	0.57	0.92	0.006871892	**	0.026430353
17_EnhW2	Hypo-	1.16	0.95	1.41	0.137800729		0.24754622
9_TxReg	All	0.82	0.66	1.01	0.063930925	.	0.145297556
9_TxReg	Hyper-	0.78	0.57	1.05	0.105100077		0.198302031
9_TxReg	Hypo-	0.86	0.63	1.16	0.361650756		0.49163929
20_ZNF/Rpts	All	1.14	0.57	2.05	0.625840543		0.727721562
20_ZNF/Rpts	Hyper-	1.40	0.56	2.89	0.362938078		0.49163929
20_ZNF/Rpts	Hypo-	0.86	0.23	2.21	1		1
14_EnhA2	All	0.85	0.67	1.06	0.16654374		0.282055273
14_EnhA2	Hyper-	0.78	0.55	1.08	0.16102031		0.274466438
14_EnhA2	Hypo-	0.92	0.65	1.25	0.646200335		0.74098911
25_Quies	All	1.08	1.02	1.14	0.004891544	**	0.021580343
25_Quies	Hyper-	0.89	0.82	0.97	0.004168821	**	0.018666361
25_Quies	Hypo-	1.32	1.22	1.42	3.38098056029921e-12	***	9.22085607354331e-11
19_DNase	All	0.83	0.69	0.99	0.041501632	*	0.113450096
19_DNase	Hyper-	0.77	0.58	0.99	0.043528615	*	0.113552908
19_DNase	Hypo-	0.90	0.69	1.15	0.47195032		0.597405469
8_TxWk	All	1.06	0.95	1.19	0.262639271		0.386234222
8_TxWk	Hyper-	0.85	0.72	1.01	0.067152479	.	0.147328927
8_TxWk	Hypo-	1.30	1.12	1.50	0.000681035	***	0.00392905
22_PromP	All	1.12	0.92	1.36	0.220391411		0.343858247
22_PromP	Hyper-	1.44	1.13	1.82	0.002917549	**	0.013465609
22_PromP	Hypo-	0.78	0.55	1.08	0.141253682		0.250746181
1_TssA	All	1.10	0.99	1.22	0.068262403	.	0.147328927
1_TssA	Hyper-	1.72	1.53	1.94	8.78384532402764e-17	***	6.58788399302073e-15
1_TssA	Hypo-	0.48	0.38	0.60	1.37613116807788e-13	***	5.89770500604805e-12
21_Het	All	0.87	0.57	1.26	0.527348267		0.645732572
21_Het	Hyper-	1.05	0.61	1.69	0.801934714		0.86852135
21_Het	Hypo-	0.67	0.32	1.23	0.240860165		0.365293021
14_EnhA2	All	0.87	0.68	1.09	0.246471704		0.371565382
14_EnhA2	Hyper-	0.94	0.67	1.28	0.759821828		0.825893291
14_EnhA2	Hypo-	0.79	0.54	1.12	0.203422519		0.326346287
2_PromU	All	0.90	0.82	1.00	0.042732869	*	0.113450096
2_PromU	Hyper-	1.13	0.99	1.28	0.066038302	.	0.146751782
2_PromU	Hypo-	0.67	0.57	0.79	4.45198417507836e-7	***	6.35998E-06
17_EnhW2	All	0.91	0.77	1.07	0.259789869		0.385826538
17_EnhW2	Hyper-	0.64	0.49	0.83	0.000453459	***	0.002776277

17_EnhW2	Hypo-	1.21	0.98	1.48	0.073038043	.	0.153226664
15_EnhAF	All	0.91	0.72	1.13	0.449672625		0.576503365
15_EnhAF	Hyper-	0.63	0.42	0.91	0.010844078	*	0.039195464
15_EnhAF	Hypo-	1.21	0.90	1.60	0.185470867		0.304050602
12_TxEnhW	All	0.85	0.67	1.06	0.158416923		0.271571868
12_TxEnhW	Hyper-	0.73	0.51	1.02	0.070068441	.	0.149081788
12_TxEnhW	Hypo-	0.97	0.71	1.31	0.940058255		0.969132222
5_Tx5'	All	1.03	0.86	1.23	0.71252219		0.792361261
5_Tx5'	Hyper-	0.98	0.75	1.27	0.949169658		0.975174307
5_Tx5'	Hypo-	1.08	0.83	1.39	0.507032119		0.632863444
3_PromD1	All	0.91	0.81	1.02	0.092772103	.	0.183102836
3_PromD1	Hyper-	1.14	0.98	1.31	0.07743193	.	0.157562832
3_PromD1	Hypo-	0.67	0.56	0.81	8.14707E-06	***	8.14707E-05
7_Tx3'	All	1.08	0.97	1.21	0.140594335		0.250746181
7_Tx3'	Hyper-	0.76	0.64	0.91	0.001677934	**	0.008389668
7_Tx3'	Hypo-	1.45	1.25	1.66	4.79606949231599e-7	***	6.54009E-06
16_EnhW1	All	0.87	0.71	1.06	0.17906582		0.296794177
16_EnhW1	Hyper-	0.81	0.61	1.07	0.157959413		0.271571868
16_EnhW1	Hypo-	0.94	0.70	1.23	0.688944347		0.774094772
24_ReprPC	All	1.12	0.98	1.27	0.090377653	.	0.180755306
24_ReprPC	Hyper-	1.54	1.32	1.79	1.33354182815246e-7	***	2.22257E-06
24_ReprPC	Hypo-	0.67	0.53	0.85	0.000432855	***	0.002705341
23_PromBiv	All	1.10	0.95	1.27	0.177659078		0.296098463
23_PromBiv	Hyper-	1.62	1.37	1.91	5.94390209749138e-8	***	1.27369E-06
23_PromBiv	Hypo-	0.56	0.42	0.74	1.1692E-05	***	0.000113149
6_Tx	All	1.25	1.03	1.52	0.022869546	*	0.071467331
6_Tx	Hyper-	0.92	0.66	1.25	0.707462606		0.791935753
6_Tx	Hypo-	1.61	1.25	2.06	0.000308909	***	0.002059392
4_PromD2	All	0.79	0.53	1.13	0.219423082		0.343858247
4_PromD2	Hyper-	0.30	0.11	0.66	0.000595954	***	0.003505612
4_PromD2	Hypo-	1.32	0.85	1.98	0.192195552		0.311668463
10_TxEnh5'	All	0.78	0.61	0.99	0.041538048	*	0.113450096
10_TxEnh5'	Hyper-	0.69	0.47	0.97	0.031245758	*	0.092809182
10_TxEnh5'	Hypo-	0.89	0.62	1.22	0.536396233		0.648866411
9_TxReg	All	0.73	0.58	0.92	0.00517361	**	0.021979975
9_TxReg	Hyper-	0.83	0.60	1.10	0.221215472		0.343858247
9_TxReg	Hypo-	0.64	0.44	0.90	0.008773419	**	0.032273232
13_EnhA1	All	0.72	0.56	0.91	0.006185254	**	0.024450979
13_EnhA1	Hyper-	0.83	0.59	1.13	0.274129188		0.395378636
13_EnhA1	Hypo-	0.60	0.40	0.88	0.006256427	**	0.024450979
18_EnhAc	All	0.96	0.51	1.64	1		1

18_EnhAc	Hyper-	0.14	0.00	0.79	0.012817173	*	0.04471107
18_EnhAc	Hypo-	1.85	0.95	3.24	0.045213374	*	0.116324521
20_ZNF/Rpts	All	1.09	0.54	1.96	0.749665372		0.819625185
20_ZNF/Rpts	Hyper-	1.34	0.54	2.77	0.377698175		0.508114136
20_ZNF/Rpts	Hypo-	0.83	0.22	2.12	1		1
11_TxEnh3'	All	0.79	0.60	1.01	0.059738097	.	0.13878684
11_TxEnh3'	Hyper-	0.39	0.22	0.63	1.69136E-05	***	0.000140947
11_TxEnh3'	Hypo-	1.23	0.89	1.64	0.187471368		0.30565984

Supplementary Table ST6. Details of sample description indicating the diagnosis, age, sex, race, postmortem interval, and sample source repository.

Sentrix_ID	Sentrix Position	Sample Name	Age	Sex	PMI	Repository	Race	Group	Row
202226400120	R05C01	21-UR-14901-Normal	71	F	16	Miami	White	Control	5
202226400120	R06C01	22-UR-14902-Normal	75	M	14	Miami	White	Control	6
202226400120	R07C01	23-UR-14903-Normal	76	M	27	Miami	White	Control	7
202226400120	R08C01	24-UR-14904-Normal	77	M	14	Miami	White	Control	8
202226400158	R05C01	25-UR-14905-Normal	79	F	17	Miami	White	Control	5
202226400158	R06C01	26-UR-14906-Normal	82	F	14	Miami	White	Control	6
202226400158	R07C01	27-UR-14907-Normal	83	F	6	Miami	White	Control	7
202226400158	R08C01	28-UR-14908-Normal	84	M	15	Miami	White	Control	8
202226400181	R05C01	29-UR-14909-Normal	86	F	20	Miami	White	Control	5
202226400181	R06C01	30-UR-14910-Normal	87	F	13	Miami	White	Control	6
202226400181	R07C01	31-UR-14911-Normal	71	M	19	Miami	White	Control	7
202226400181	R08C01	32-UR-14912-Normal	86	F	25	Miami	White	Control	8
202226400144	R01C01	1-UR-14914-PD	78	M	14.83	Harvard	White	PD	1
202226400144	R02C01	2-UR-14915-PD	73	M	18.45	Harvard	White	PD	2
202226400144	R03C01	3-UR-14916-PD	71	M	12.8	Harvard	White	PD	3
202226400144	R05C01	1-UR-14917-Normal	80	M	15.5	Harvard	Unknown	Control	5
202226400144	R04C01	4-UR-14918-PD	71	F	19.83	Harvard	White	PD	4
202226400144	R07C01	3-UR-14920-Normal	82	F	15.7	Harvard	Unknown	Control	7
202242400035	R01C01	5-UR-14921-PD	70	F	19.58	Harvard	White	PD	1
202242400035	R02C01	6-UR-14922-PD	85	F	10.6	Harvard	White	PD	2
202226400144	R08C01	4-UR-14923-Normal	76	M	18.25	Harvard	White	Control	8
202242400035	R03C01	7-UR-14925-PD	79	F	18.5	Harvard	White	PD	3
202242400035	R04C01	8-UR-14926-PD	80	F	15.62	Harvard	Unknown	PD	4
202242400129	R01C01	9-UR-14927-PD	72	F	9.67	Harvard	White	PD	1

202242400129	R02C01	10-UR-14928-PD	76	M	11	Harvard	Unknown	PD	2
202242400035	R05C01	5-UR-14929-Normal	72	M	19.28	Harvard	Unknown	Control	5
202242400035	R06C01	6-UR-14930-Normal	73	M	19.3	Harvard	Unknown	Control	6
202242400035	R07C01	7-UR-14931-Normal	74	M	18.58	Harvard	Unknown	Control	7
202242400035	R08C01	8-UR-14932-Normal	83	M	13	Harvard	White	Control	8
202242400129	R03C01	11-UR-14933-PD	83	F	13.95	Harvard	Unknown	PD	3
202242400129	R04C01	12-UR-14934-PD	72	M	7.75	Harvard	White	PD	4
202242410050	R01C01	13-UR-14935-PD	73	F	20.97	Harvard	Unknown	PD	1
202242400129	R05C01	9-UR-14936-Normal	79	F	17.58	Harvard	Unknown	Control	5
202242400129	R06C01	10-UR-14937-Normal	83	F	8.86	Harvard	White	Control	6
202242400129	R07C01	11-UR-14938-Normal	77	F	20.61	Harvard	White	Control	7
202242410050	R02C01	14-UR-14939-PD	74	M	15.15	Harvard	White	PD	2
202242410050	R03C01	15-UR-14940-PD	82	M	18.08	Harvard	White	PD	3
202242410050	R04C01	16-UR-14941-PD	79	F	4.95	Harvard	Unknown	PD	4
202242410112	R01C01	17-UR-14942-PD	70	F	20.92	Harvard	Unknown	PD	1
202242410112	R02C01	18-UR-14943-PD	79	M	11.77	Harvard	White	PD	2
202242400129	R08C01	12-UR-14944-Normal	85	M	20.83	Harvard	White	Control	8
202242410112	R03C01	19-UR-14945-PD	71	M	20.92	Harvard	Unknown	PD	3
202242410050	R05C01	13-UR-14946-Normal	84	F	18.37	Harvard	White	Control	5
202242410112	R04C01	20-UR-14947-PD	74	M	12.75	Harvard	White	PD	4
202242410050	R06C01	14-UR-14948-Normal	74	M	20.53	Harvard	Unknown	Control	6
202226400120	R01C01	21-UR-14949-PD	83	M	17.25	Harvard	White	PD	1
202226400120	R02C01	22-UR-14950-PD	87	M	12.3	Harvard	White	PD	2
202226400120	R03C01	23-UR-14951-PD	84	F	18.92	Harvard	Unknown	PD	3
202226400120	R04C01	24-UR-14952-PD	78	F	20.17	Harvard	White	PD	4
202226400158	R01C01	25-UR-14953-PD	72	M	6	Harvard	White	PD	1
202226400158	R02C01	26-UR-14954-PD	70	M	20.38	Harvard	White	PD	2
202226400158	R03C01	27-UR-14955-PD	77	F	11.25	Harvard	White	PD	3
202226400158	R04C01	28-UR-14956-PD	87	F	5.58	Harvard	Unknown	PD	4
202226400181	R01C01	29-UR-14957-PD	83	M	15.03	Harvard	White	PD	1
202242410050	R07C01	15-UR-14958-Normal	79	F	20.6	Harvard	Unknown	Control	7
202226400181	R02C01	30-UR-14959-PD	84	M	13.16	Harvard	Unknown	PD	2
202226400181	R03C01	31-UR-14960-PD	79	M	8.16	Harvard	White	PD	3
202226400181	R04C01	32-UR-14961-PD	84	M	19.9	Harvard	White	PD	4
202242410050	R08C01	16-UR-14962-Normal	69	F	18.65	Harvard	Unknown	Control	8
202226400182	R01C01	33-UR-14964-PD	83	F	18.3	Harvard	White	PD	1
202226400182	R02C01	34-UR-14965-PD	73	F	19.25	Harvard	White	PD	2
202226400182	R03C01	35-UR-14966-PD	81	F	13.8	Harvard	White	PD	3
202226400182	R04C01	36-UR-14967-PD	77	M	6.62	Harvard	White	PD	4
202242410112	R05C01	17-UR-14968-Normal	78	M	19.18	Harvard	Unknown	Control	5
202229250195	R01C01	37-UR-14969-PD	80	M	12.83	Harvard	White	PD	1
202229250195	R02C01	38-UR-14970-PD	87	F	11.5	Harvard	White	PD	2
202242410112	R06C01	18-UR-14971-Normal	70	F	17.18	Harvard	White	Control	6

202242410112	R07C01	19-UR-14972-Normal	72	M	18.85	Harvard	White	Control	7
202242410112	R08C01	20-UR-14973-Normal	77	M	14.55	Harvard	Unknown	Control	8
202226400182	R05C01	33-UR-14974-Normal	86	F	20.33	Harvard	Unknown	Control	5
202229250195	R03C01	39-UR-14975-PD	81	M	19.17	Harvard	White	PD	3
202229250195	R04C01	40-UR-14976-PD	74	F	19.67	Harvard	Unknown	PD	4
202226400182	R06C01	34-UR-14977-Normal	74	F	12.5	Harvard	White	Control	6
202226400182	R07C01	35-UR-14979-Normal	59	F	26.58	Mount Sinai	Black	Control	7
202226400182	R08C01	36-UR-14980-Normal	79	M	13	Mount Sinai	White	Control	8
202229250195	R05C01	37-UR-14981-Normal	73	M	14.92	Mount Sinai	Asian	Control	5
202229250195	R06C01	38-UR-14982-Normal	74	M	19.17	Mount Sinai	White	Control	6
202229250195	R07C01	39-UR-14983-Normal	83	M	13.9	Mount Sinai	Hispanic	Control	7

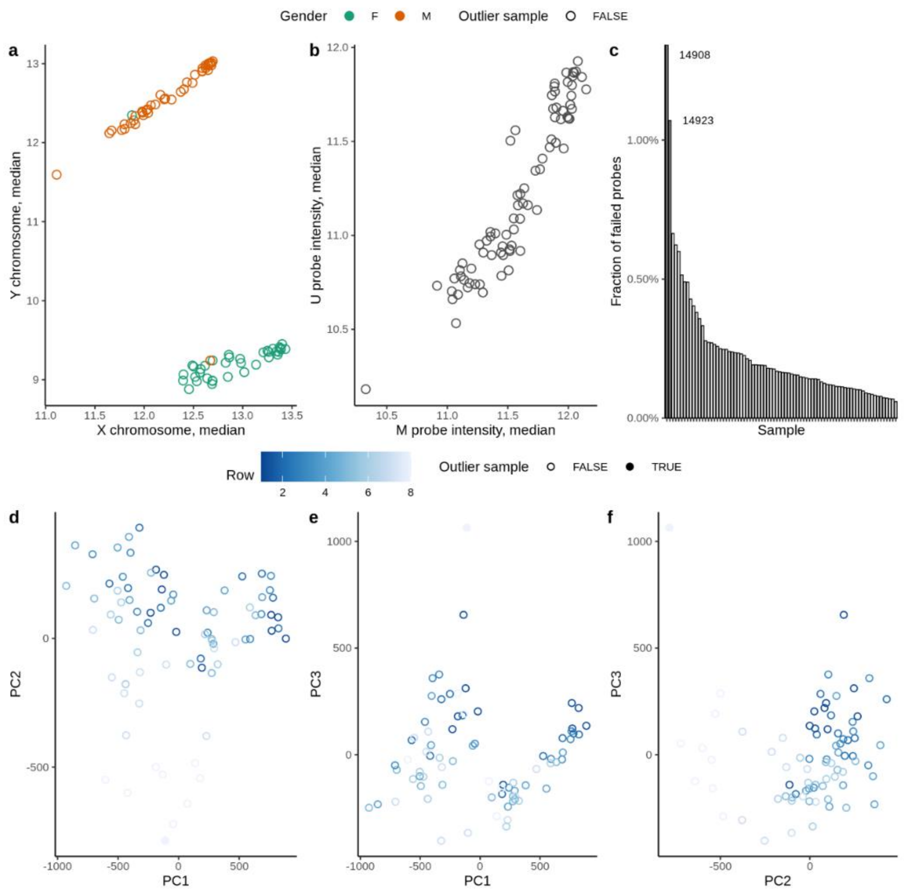

Supplementary Figure S1: Detection of outliers in EPIC array methylation data. (a) Median signal intensity in sex chromosomes indicating the sex of all samples. (b) Median overall probe intensity. (c) Fraction of failed probes. Samples that deviate by more than 2 SD from average fraction of failed probes are considered outliers. Principal component analysis, sample projections on the first three principal components (d, e, f).

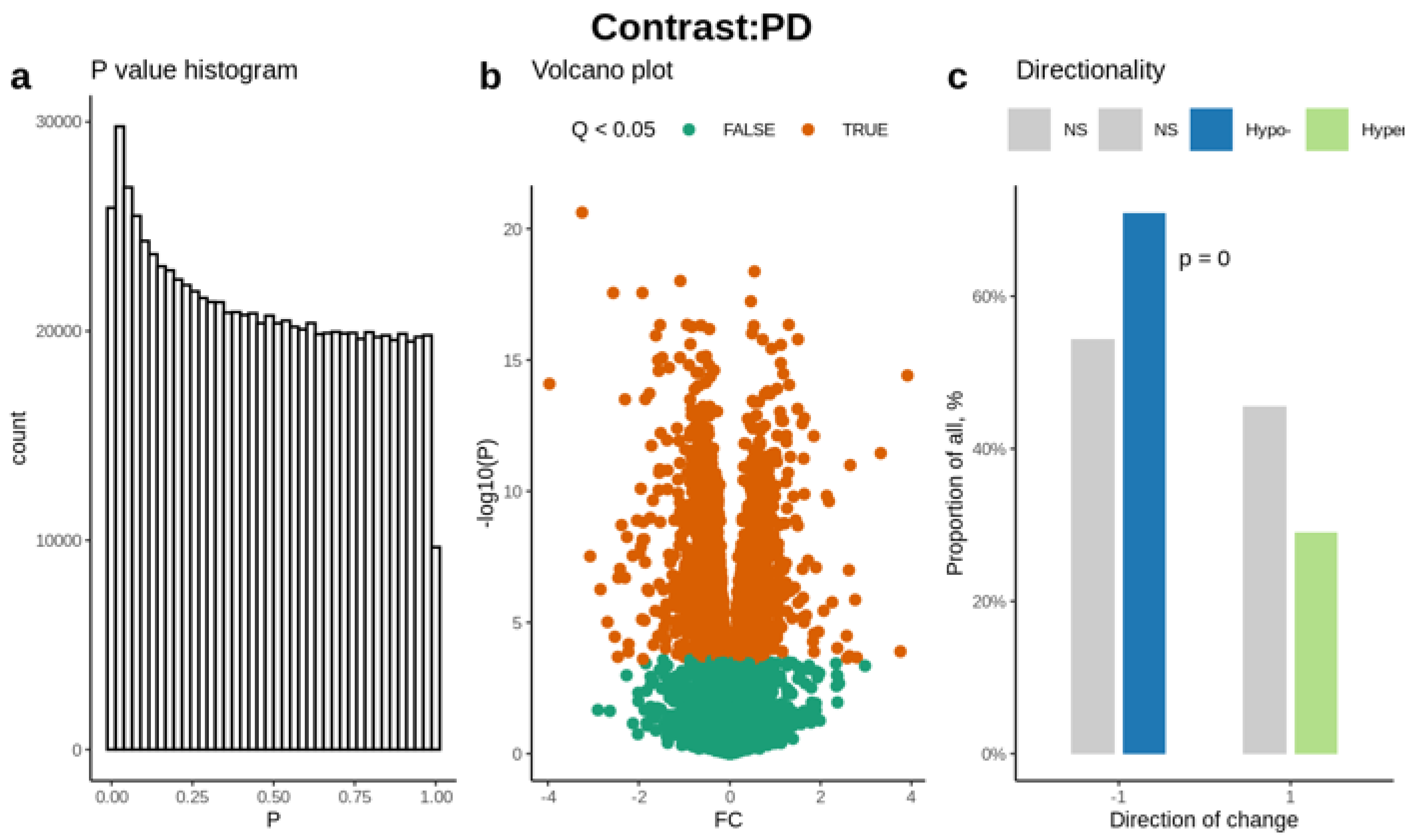

Supplementary Figure S2: Linear model of methylome data with all the samples: Robust linear models fitted to the methylation data using Age, Sex, Race, PMI, Repository, NeuN proportion and Sentrix ID as covariates. (a) Histogram indicating the distribution of p values. (b) Volcano plot showing the fold change and p values for each CpG. Significant differentially modified cytosines are shown in red. (c) Proportion of hyper- and hypo- modified CpGs. Non-significant CpGs are shown in grey. Significant hyper-modified CpGs are shown in green. Significant hypo modified CpGs are shown in blue. Fisher's exact test was used to obtain the p value.

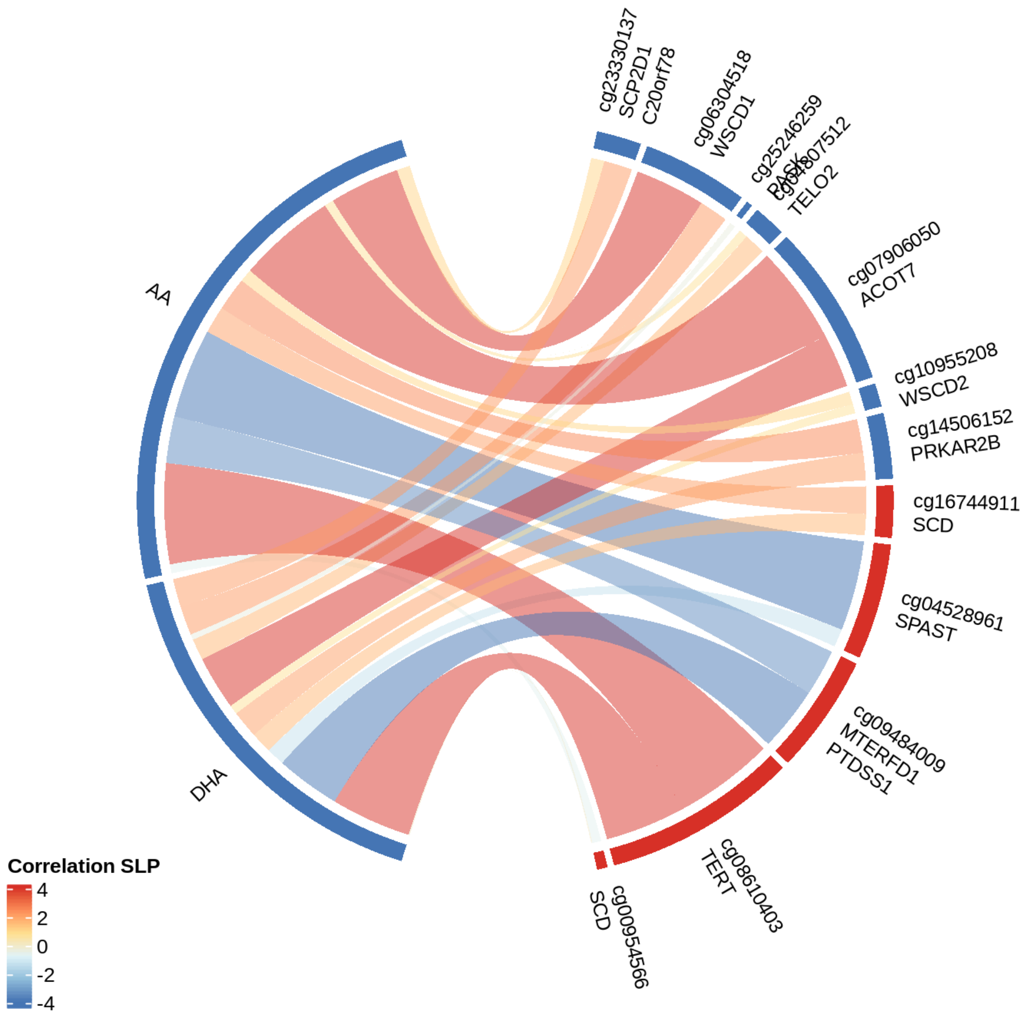

Supplementary Figure S3: Correlation between significantly differentially methylated cytosines (genes) with metabolites of Biosynthesis of unsaturated fatty acids: The red strip around the "circos plot" shows the positive correlation and the blue

strip shows the negative correlation. The metabolite correlated with CpGs and the genes encompassed under CpGs are shown.

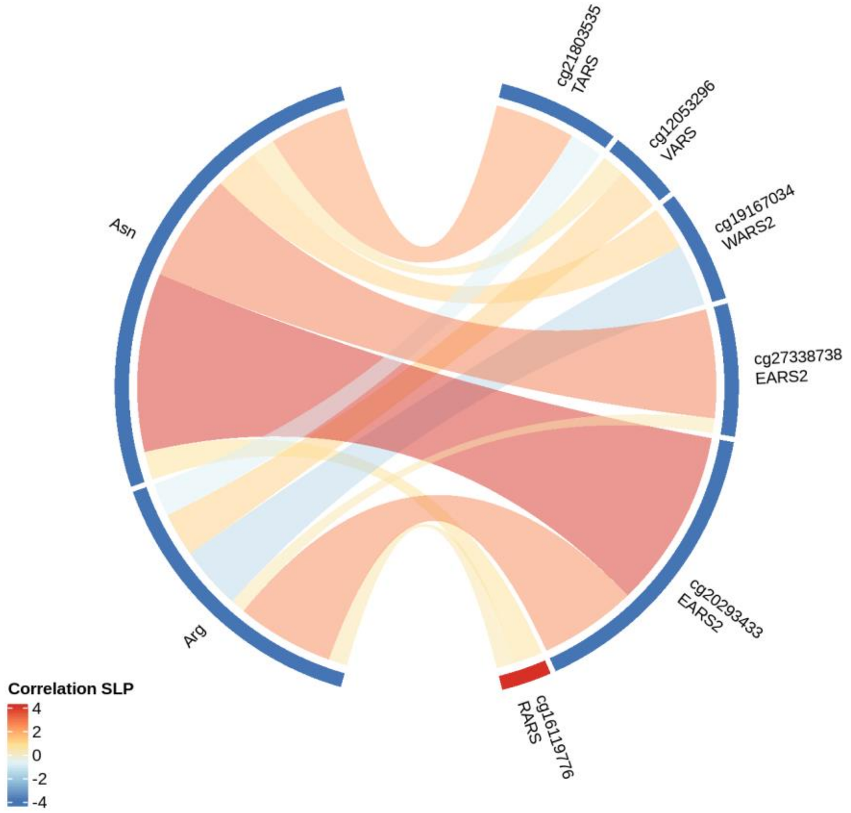

Supplementary Figure S4: Correlation between significantly differentially methylated cytosines (genes) with metabolites of Aminoacyl t-RNA biosynthesis: The red strip around the “circos plot” shows the positive correlation and the blue strip shows the negative correlation. The metabolite correlated with CpGs and the genes encompassed under CpGs are shown.

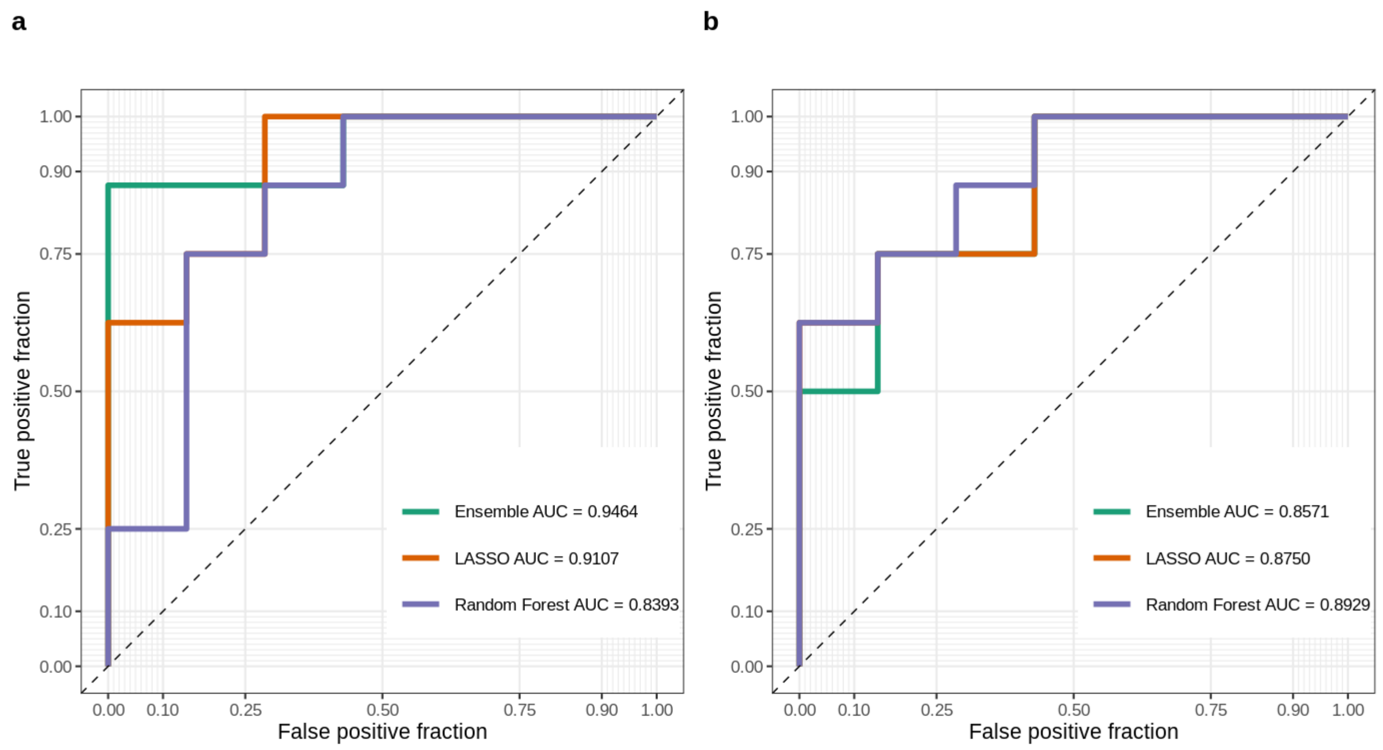

Supplementary Figure S5: Diagnostic models to discriminate between PD cases and normal controls using the brain tissues based on (a) Epigenetic (b) Metabolomic. Three types are predictions (Ensemble, LASSO and Random Forest) are detailed with the "Area Under the receiver operating characteristic Curve" (AUC) values.
